# Exploration of Nirmatrelvir Derivatives as Optimized SARS‐CoV‐2 Antivirals

**DOI:** 10.1002/cmdc.70424

**Published:** 2026-08-11

**Authors:** Yugendar R. Alugubelli, Veerabhadra R. Vulupala, Kai S. Yang, Kaustav Khatua, Lauren R. Blankenship, Sathish Kumar, Demonta Coleman, Xuejiao Guo, Zhi Zachery Geng, Chia‐Chuan D. Cho, Banumathi Sankaran, Benjamin W. Neuman, Shiqing Xu, Wenshe Ray Liu

**Affiliations:** ^1^ Texas A&M Drug Discovery Center Department of Chemistry College of Arts and Sciences Texas A&M University College Station Texas USA; ^2^ Department of Biology College of Arts and Sciences Texas A&M University College Station Texas USA; ^3^ Molecular Biophysics and Integrated Bioimaging Berkeley Center for Structural Biology Laurence Berkeley National Laboratory Berkeley California USA; ^4^ Texas A&M Global Health Research Complex Texas A&M University College Station Texas USA; ^5^ Department of Molecular Pathogenesis and Immunology College of Medicine Bryan Texas USA; ^6^ Department of Pharmaceutical Sciences Irma Lerma Rangel College of Pharmacy Texas A&M University College Station Texas USA; ^7^ Institute of Biosciences and Technology Department of Translational Medical Sciences School of Medicine Texas A&M University Houston Texas USA; ^8^ Department of Biochemistry and Biophysics College of Agriculture and Life Sciences Texas A&M University College Station Texas USA; ^9^ Department of Cell Biology and Genetics College of Medicine Texas A&M University College Station Texas USA

**Keywords:** covalent inhibitor, COVID‐19, main protease, nirmatrelvir, SARS‐CoV‐2

## Abstract

Nirmatrelvir (NMV) is a SARS‐CoV‐2 antiviral component of the approved COVID‐19 therapeutic Paxlovid. It is a reversible covalent inhibitor of SARS‐CoV‐2 main protease (M^Pro^) that is effluxed from human cells by P‐glycoprotein (P‐gp). To identify NMV analogs with improved potency and reduced P‐gp efflux, a structure–activity relationship campaign was conducted. Warheads alternative to nitrile for engaging the active site cysteine were tested showing aldehyde and dichloroacetamide with better enzyme inhibition potency. Crystal structure of MPI‐136−M^Pro^ shows its aldehyde warhead forming a thiohemiacetal with active Cys145 of M^Pro^. Several S4 binders were explored revealing that an O‐to‐S shift at the *N*‐terminal amide leads to better enzyme inhibition. By exploring different combinations of S2, S3, and S4 binders, two inhibitors with better enzyme inhibition potency than NMV were found. Crystal structure of MPI‐148, with (*S*)‐2‐azaspiro[4,5]decane‐3‐carboxylate as an alternative S2 binder, shows extensive hydrogen‐bond networks for locking the inhibitor in active site, explaining high affinity of NMV analogs. Further characterization of cellular M^Pro^ engagement and antiviral potency against SARS‐CoV‐2 revealed four inhibitors with greater potency than NMV in P‐gp‐expressing cells. Studies with the P‐gp inhibitor CP‐100356 showed that these compounds were less sensitive to P‐gp inhibition than NMV, consistent with reduced P‐gp‐mediated efflux.

## Graphical abstract

Structure‐guided optimization of nirmatrelvir identifies aldehyde and dichloroacetamide warheads and a potency‐enhancing S4 thioamide. Crystal structures reveal an S2 rearrangement extending toward Cys44, suggesting a new covalent‐design opportunity. Four analogs outperform nirmatrelvir in cellular and antiviral assays, with reduced P‐gp‐mediated efflux contributing to their enhanced activity.

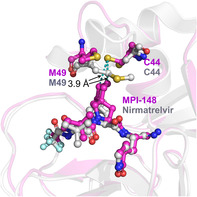

**Abbreviations**
COVID‐19: Coronavirus disease 2019
SARS‐CoV‐2: Severe Acute Respiratory Syndrome Coronavirus 2
M^Pro^
: Main Protease

## 1 Introduction

SARS‐CoV‐2, the etiologic agent of COVID‐19, carries a positive‐sense single‐stranded RNA genome [1, 4]. Upon entry into its host cells, the genomic RNA is released into the cytoplasm and immediately translated into two large polyproteins, pp1a and pp1ab.

Proteolytic processing of these polyproteins into nonstructural proteins (nsps) that assemble the replication–transcription complex requires two viral cysteine proteases: nsp3 (papain‐like protease or PL^Pro^) and nsp5, the main protease (M^Pro^, also called 3C‐like protease or 3CL^Pro^) [1, 5, 7]. M^Pro^ contains a His41‐Cys145 catalytic dyad that carries out peptide‐bond hydrolysis. By Schechter–Berger nomenclature, M^Pro^ recognizes substrates with a conserved Gln at P1 and a large hydrophobic residue, often Leu or Phe, at P2. Binding is organized across subsites S1, S2, S3, S4, and S1’. S3 is solvent‐exposed and relatively permissive [8]. S4 is shallow and favors small hydrophobic residues such as Val. S1’ is comparatively spacious but contributes less to sequence selectivity. Targeting M^Pro^ is a validated antiviral strategy for treating COVID‐19. Two oral inhibitors, nirmatrelvir (a reversible covalent nitrile, approved in the United States for combined use with ritonavir in Paxlovid) and ensitrelvir (noncovalent, approved in Japan), are clinically approved and act by occupying the M^Pro^ active site and engaging these subsites to block polyprotein processing [9, 13]. Several nirmatrelvir (NMV) analogs have also been approved for clinical use in China as well [11, 18].

**FIGURE 1:**
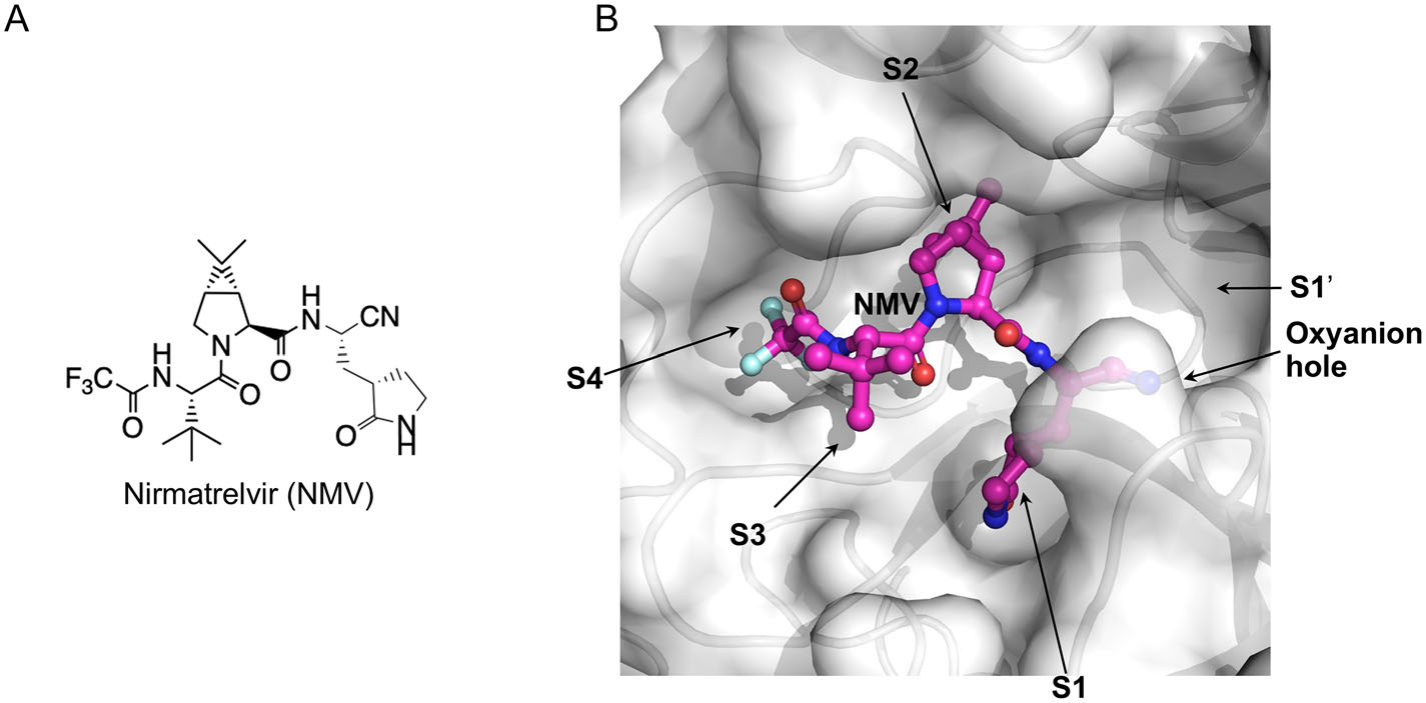
Nirmatrelvir (NMV) and its complex with M^Pro^. (A) Structures of NMV. (B) The binding mode of NMV at the active site of M^Pro^ (PDB: 7RFS). M^Pro^ surface is colored in white. NMV is depicted in a ball‐and‐stick representation and with its carbon atoms colored in purple. All other atoms are shown in a standard color system (oxygen: red; nitrogen: blue; fluorine, pale cyan).

NMV contains an activated nitrile that reacts with Cys145 to generate a thioimidate adduct [19]. As shown in its M^Pro^ complex crystal structure (Figure 1), this thioimidate adduct positions its imidine nitrogen at the enzyme's oxyanion hole. Its P1 residue is 3‐(S‐2‐oxopyrrolidin‐3‐yl)‐Ala, which both binds more tightly at S1 and is more metabolically stable than Gln. In the M^Pro^‐NMV crystal structure [20], the S1 subsite is close to complete occupation by this residue. NMV's P2 residue is dimethylcycloproylproline for tight binding to S2. The same residue has been used in other clinically approved peptidomimetics such as boceprevir [21, 23]. NMV's P3 is *tert*‐butyl‐glycine. Although part of its side chain touches on the groove forming S3, the *tert*‐butyl side chain is largely exposed to solvent [24]. The same residue exists in boceprevir for high metabolic stability. NMV has an *N*‐terminal trifluoroacetamide that fits S4 perfectly well. Compared to a regular amide, trifluoroacetamide is more labile toward base‐catalyzed hydrolysis. Although there is no report about this as a significant concern, it is good to keep in mind from a chemistry perspective. NMV has strong enzyme inhibition potency. In an assay setup we adopted for M^Pro^ inhibitor analyses, it shows an IC_50_ of 66 nM. However, its antiviral potency varies in different tested cells. It is a substrate of the P‐glycoprotein (P‐gp) multidrug transporter and is effluxed from cells expressing P‐gp. In Vero E6 cells, it exhibits antiviral potency with an EC_50_ of 74.5 nM when ritonavir is provided as a P‐gp inhibitor. When ritonavir is absent, this EC_50_ turns to 1.30 μM. In HEK293T, a human cell with strong basal‐level expression of P‐gp, NMV displays potency with an EC_50_ of 3.4 μM to engage ectopically expressed M^Pro^. Considering that NMV is susceptible to P‐gp‐mediated efflux, we sought to identify analogs with improved enzyme inhibition potency and cellular antiviral activity while reducing sensitivity to P‐gp‐mediated transport. We decided to conduct its structure–activity relationship (SAR) campaign. Here, we wish to report our progress on this research front.

We started by searching for alternative covalent warheads [25, 28]. Alkyne, as a popular click chemistry functionality, was found to irreversibly react with deubiquitinases when it was conjugated to ubiquitin's *C*‐terminus [29, 30]. The involved reaction is a thiol‐yne reaction to form a vinylthioether adduct. This reactivity has also been explored to develop peptidomimetic inhibitors for cysteine proteases [31]. We replaced the nitrile in NMV with an alkyne to afford MPI‐134 (Table 1), which shows about 60‐fold reduced potency with an enzyme inhibition IC_50_ of 3.9 μM. In our assay conditions, M^Pro^ was incubated with an inhibitor for 30 min before its fluorogenic substrate was provided to detect residual enzyme activity. Prolonging this incubation time for an irreversible inhibitor such as MPI‐134 will definitely lead to better potency. But due to its significantly reduced potency, we decided not to explore further. In a previous study [19], we showed peptidomimetic inhibitors with an aza‐dichloroacetyl warhead and P1 propanamide side chain displaying high inhibition potency against M^Pro^; we integrated both components into our design to generate MPI‐135. MPI‐135 has a detected IC_50_ of 26 nM that is more than twofold less than that for NMV. Aldehyde is also a commonly used warhead for M^Pro^ inhibitor designs. We integrated it into our design as well to synthesize MPI‐136, that contains also a *N*‐terminal *O*‐isopropylcarbamate. We incorporated both aldehyde warhead and isopropyl as a strong S4 binder for achieving better potency than NMV. MPI‐136 does show about twofold better potency than NMV with an IC_50_ of 28 nM. Although MPI‐135 and MPI‐136 show better potency than NMV, this mild potency improvement doesn’t compensate for concerns related to metabolic stability and toxicity of the two alternative warheads. Therefore, we decided to keep the nitrile warhead and explore alternative binders at S2, S3, and S4. 3‐(S‐2‐oxopyrrolidin‐3‐yl)‐Ala is maintained as a S1 binder for its validated optimized interaction features at S1 [32].

**TABLE 1:** Structures and enzymatic IC_50_ of group 1 inhibitors.

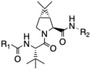			
ID	***R*** **_1_**	***R*** **_2_**	**IC** **_50_** **, μM**
Nirmatrelvir	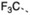	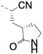	0.066
MPI‐134	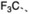	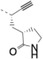	3.9
MPI‐135	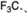	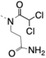	0.026
MPI‐136	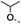	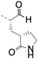	0.028

**FIGURE 2:**
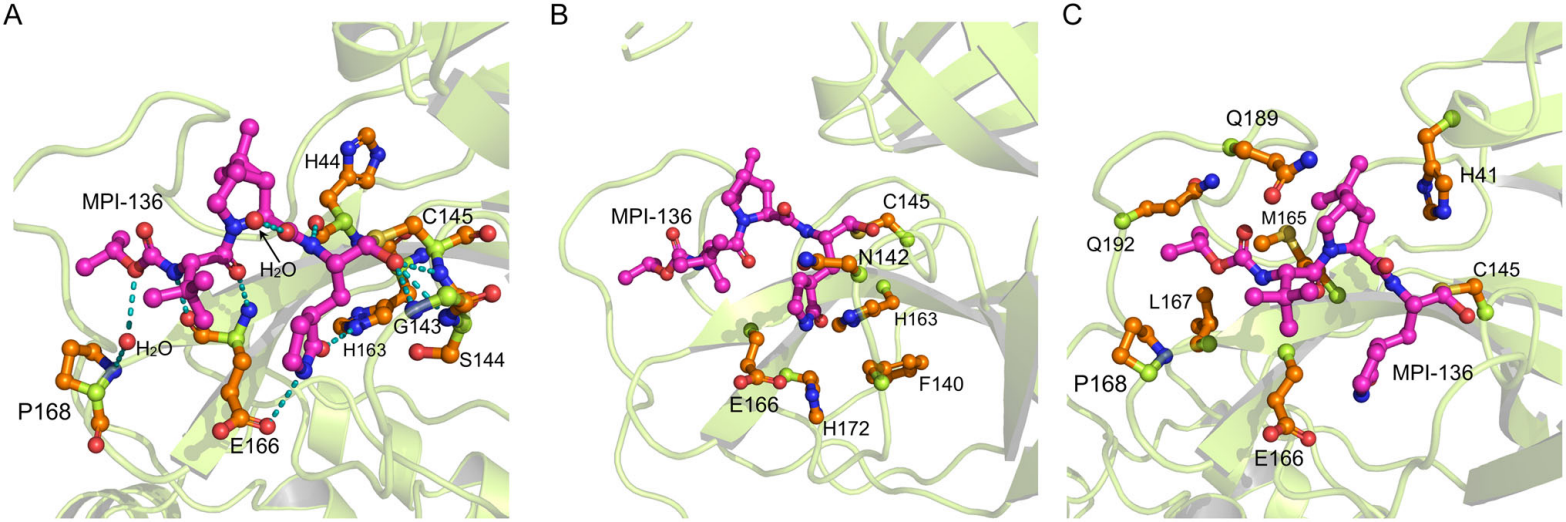
Crystal structure of M^Pro^ bound with MPI‐136. (A) Hydrogen bonds involving MPI‐136 in the active site of M^Pro^. (B) Interactions at the S1 subsite. (C) Interactions at S2, S3, and S4 subsites. M^Pro^ secondary structures are presented in the limon color. M^Pro^ residues are presented in ball‐and‐stick representations with their non‐Cα carbon atoms colored in orange. MPI‐136 is depicted in a ball‐and‐stick representation with its carbon atoms colored in purple. All other atoms are shown in a standard color system (oxygen: red; nitrogen: blue; sulfur, brown).

We determined the structure of M^Pro^ bound with MPI‐136 using X‐ray crystallography. The crystal diffracted X‐rays to 2.0 Å resolution. In the active site, 2F_o_‐F_c_ electron density maps delineate unambiguously the structure of MPI‐136 that forms a covalent bond with the thiol group of Cys145. The generated thiohemiacetal hydroxide poises at exactly the oxyanion hole and is within hydrogen‐bond distance to three backbone NH groups from aa 143–145 (Figure 2A). Figure 2A presents several other key hydrogen bonds for locking MPI‐136 in the active site. The P1 side chain lactam amide forms two hydrogen bonds with His163 and Glu166. The inhibitor's three backbone amides form three hydrogen bonds with His44 and Glu166. There is a water molecule that can be clearly resolved and forms a hydrogen bond with the inhibitor backbone amide oxygen between P1 and P2 residues. MPI‐136 has an *N*‐terminal carbamate. Its carbamate oxygen forms a hydrogen bond with a water molecule that connects with the Pro166 amine through a hydrogen bond as well. At S1 (Figure 2B), MPI‐136's P1 3‐(S‐2‐oxopyrrolidin‐3‐yl)‐Ala packs tightly against residues Phe140, Asn142, His163, Glu166, and His172, leaving little vacancy for further chemistry maneuvers. At S2 (Figure 2C), MPI‐136's P2 dimethylcycloproylproline interacts with His41, Met165, Gln189, and the loop backbone containing aa 187–189. A small α‐helix containing Met49 that caps S2 in apo‐M^Pro^ and some other M^Pro^‐inhibitor complexes is unresolvable in this structure, indicating a flexible conformation for this region. This conformational flexibility allows Cys44 to adopt about a 180° flip to form a covalent S—O—N—O—S crosslink with Lys61 and Cys22. This novel posttranslational crosslink has been observed in many other M^Pro^‐inhibitor complexes and other proteins as well. The conformational flexibility of this Met49‐containing region indicates that S2 may adopt a flexible conformation for binding different hydrophobic moieties, and therefore many additional S2 binder designs can be tested. MPI‐136's P3 *tert*‐butylglycine binds M^Pro^ with no difference from NMV. Its *N*‐terminal *O*‐isopropylcarbamate, besides that unique hydrogen bond with a water molecule, points the isopropyl moiety for hydrophobic interactions for tight packing against side chains of Leu167, Pro168, and Glu192 and the backbone of aa 189–192. However, S4 is not fully occupied. Superimposing the resolved M^Pro^‐MPI‐136 complex structure over M^Pro^‐NMV (PDB: 7RFS) as shown in Figure S2 reveals a space for two fluorides in M^Pro^‐NMV not occupied in M^Pro^‐MPI‐136, implying that further designs may take advantage of this small unoccupied space. Figure S2 also reveals that, compared to M^Pro^‐NMV, the aa 187–192 loop region in M^Pro^‐MPI‐136 moves more toward the solvent to accommodate the isopropyl functional group at the *N*‐terminus of MPI‐136, indicating that S4 has flexibility to expand its size to accommodate chemical moieties larger than trifluoromethyl.

Both M^Pro^‐NMV and M^Pro^‐MPI‐136 structures show an *N*‐terminal amide or carbamate oxygen that forms only van der Waals interactions with the side chain of Gln189. Since no hydrogen bond is involved and the Gln189 side chain has been found to adopt different conformations for binding inhibitors, we then decided to explore alternative *N*‐terminal functional groups in NMV analogs for engaging S4. The *N*‐terminal oxygen of NMV was first removed to afford MPI‐137, which has an *N*‐terminal secondary amine (Table 2). Although this amine is likely protonated at pH 7.4 and consequently requires significant desolvation energy, MPI‐137 has an IC_50_ of 280 nM that is only fourfold weaker than NMV. A sulfonate derivative, MPI‐138, was also synthesized, showing an IC_50_ of 280 nM. Although both MPI‐137 and MPI‐138 are weaker inhibitors than NMV, they are still potent inhibitors, indicating a flexible recognition mode for S4. Suspecting that replacing the *N*‐terminal oxygen with sulfur may introduce more favorable hydrophobic interactions with M^Pro^, we synthesized three sulfur‐containing inhibitors, MPI‐139, MPI‐140, and MPI‐142. MPI‐142 has a *tert‐*butylthiourea component that is structurally aligned with boceprevir. However, it shows weak potency with an IC_50_ of 330 nM. MPI‐139's *N*‐terminus is structurally aligned with that of MPI‐136. Its IC_50_ of 70 nM is equivalent to that of NMV. A surprising result was found with MPI‐142. MPI‐142 is a close NMV homolog with only the *N*‐terminal amide oxygen switched to sulfur. Its IC_50_ of 11 nM is one‐sixth of that of NMV, showing much better enzyme inhibition potency for this inhibitor. Phthalimide and *tert*‐butoxycarbonyl (Boc) have been used as *N*‐terminal caps in other peptidomimetic inhibitors for M^Pro^. We integrated them with NMV to afford MPI‐141 and MPI‐143 that shows IC_50_s of 210 and 320 nM, respectively. To merge S4 binding features of NMV and MPI‐136, we developed MPI‐144 that has an *N*‐terminal 1,1′‐bi(cyclopropane)‐1‐carbonyl group. However, MPI‐144 shows significantly weaker potency than NMV, with an IC_50_ of 510 nM. Although MPI‐142 is the only one in this series of inhibitors with potency better than NMV, all other inhibitors still show high inhibition potency, implying that all these alternative S4 binders may be tuned for better binding at S4.

**TABLE 2:** Structures and enzymatic IC_50_ of group 2 inhibitors.

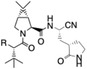		
ID	***R***	**IC** **_50_,** **μM**
MPI‐137	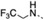	0.28
MPI‐138	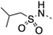	0.28
MPI‐139	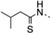	0.070
MPI‐140	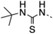	0.33
MPI‐141	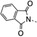	0.21
MPI‐142	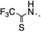	0.011
MPI‐143	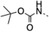	0.32
MPI‐144	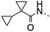	0.51

*O*‐*tert*‐butyl‐threonine is an S3 binder whose incorporation in M^Pro^ inhibitors leads to favorable cell penetration and antiviral potency [24]. We integrated it with NMV to generate two inhibitors, MPI‐145 and MPI‐146, that have an *N*‐terminal trifluorothioacetyl and trifluoroacetyl group, respectively. As shown in Table 3, MPI‐146 has weaker potency than NMV, but MPI‐145 shows equivalent potency. NMV has a bicyclic S2 binder. Alternative spiro binders with favorable binding to S2 include (*S*)‐2‐azaspiro[4,4]nonane‐3‐carbonyl, (*S*)‐2‐azaspiro[4,5]‐decane‐3‐carbonyl, (*S*)‐1,4‐dithia‐7‐azaspiro[4,4]nonane‐8‐carbonyl. We integrated them with NMV to generate MPI‐147, MPI‐148, and MPI‐149. MPI‐149 shows better potency than NMV with an IC_50_ of 25 nM. The other two inhibitors have weaker potency, but they may still be considered with potency at a similar level to NMV. By integrating both *O*‐*tert*‐butyl‐threonine as an alternative S2 binder and spiro S3 binders with NMV, we synthesized three more inhibitors, MPI‐150, MPI‐151, and MPI‐152. MPI‐150 shows slightly better potency than NMV. On the contrary, both MPI‐151 and MPI‐152 show about twofold weaker potency than NMV.

**TABLE 3:** Structures and enzymatic IC_50_ of group 3 inhibitors.

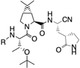		
ID	***R***	**IC** **_50_,** **μM**
MPI‐145	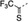	0.065
MPI‐146	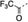	0.25
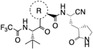		
MPI‐147	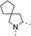	0.39
MPI‐148	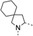	0.16
MPI‐149	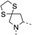	0.025
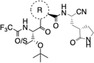		
MPI‐150	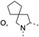	0.047
MPI‐151	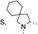	0.12
MPI‐152	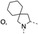	0.12

**FIGURE 3:**
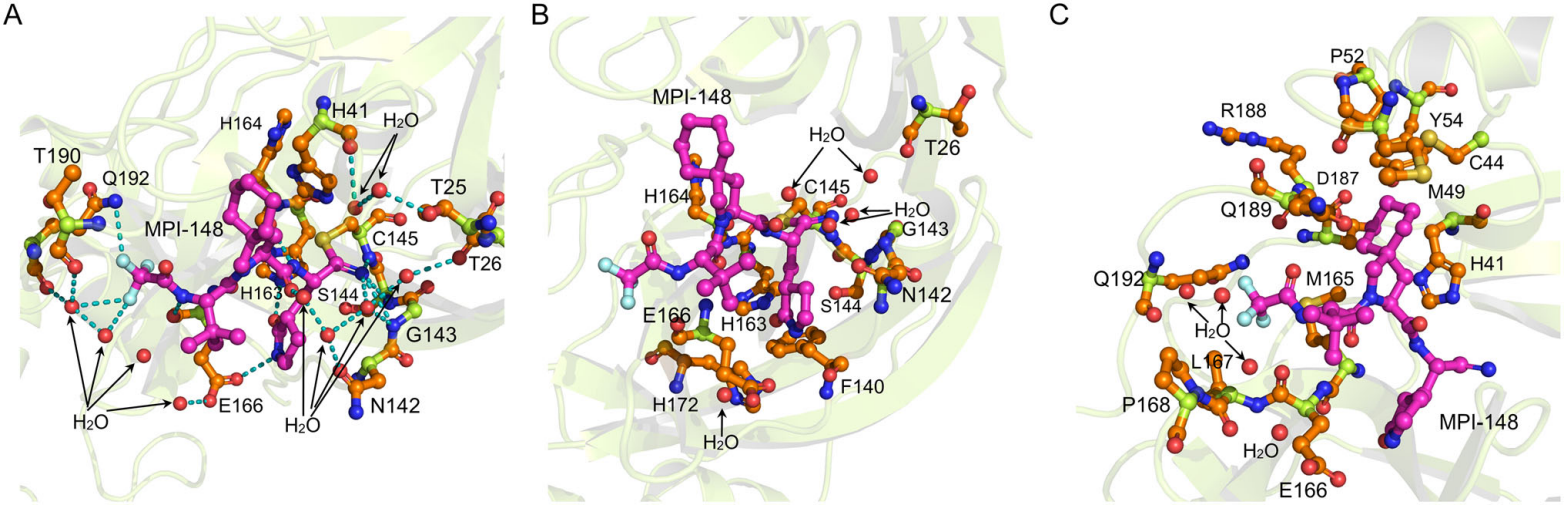
Crystal structure of M^Pro^ bound with MPI‐148. (A) Extensive hydrogen bond network involving MPI‐148 in the active site of M^Pro^. (B) Interactions at the S1 and S1’ subsites. (C) Interactions at S2, S3, and S4 subsites. M^Pro^ secondary structures are presented in the limon color. M^Pro^ residues are presented in ball‐and‐stick representations with their non‐Cα carbon atoms colored in orange. MPI‐148 is depicted in a ball‐and‐stick representation with its carbon atoms colored in purple. All other atoms are shown in a standard color system (oxygen: red; nitrogen: blue; sulfur; brown).

Crystal structure of M^Pro^ bound with MPI‐148 was successfully determined. The protein crystal used for this study diffracted X‐ray to 1.85 Å resolution. This resolution and high quality of the data allowed unambiguously delineating precise positions of all non‐hydrogen atoms in MPI‐148 bound at the M^Pro^ active site (Figure S3). One additional advantage of this determined M^Pro^‐MPI‐148 complex structure is its clearly defined water molecule positions around MPI‐148 at the M^Pro^ active site (Figure S4), allowing the clarification of water‐mediated hydrogen bonding networks in facilitating the binding of MPI‐148. As shown in Figure 3A, MPI‐148 forms all necessary hydrogen bonds with M^Pro^ that were observed in M^Pro^‐NMV. Its nitrile reacts with Cys145 to form a thioimidate. The generated imidine nitrogen poises at the oxyanion hole and is within hydrogen bond distance to three backbone NH groups from aa 143–145. Two hydrogen bonds are formed between its P1 lactam amide and two M^Pro^ residues, His166 and Glu166. And three more hydrogen bonds are formed between three MPI‐148 backbone amides and backbone oxygen or nitrogen from M^Pro^ residues, including His164 and Glu166. Around S1, S1’, and the S1–S2 bridging space, there are four water molecules connecting the covalent adduct's imidine nitrogen, Asn142 side chain amide, Thr26 backbone oxygen, and MPI‐148's P2 carbonyl oxygen through a network of hydrogen bonds. Although not directly interacting with MPI‐148, two water molecules fill the open space of S1’ by connecting Thr26 side chain hydroxide and His41 backbone oxygen via a hydrogen bond network. At S4, two water molecules are within hydrogen‐bond distance to a fluoride atom in the M148's *N*‐terminal trifluoroacetyl group and connect with Thr190 backbone oxygen and Gln192 backbone oxygen. The final refined structure also shows that one of MPI‐148's fluoride atoms is 3.2 Å to Gln192 side chain amide, indicating a possible weak hydrogen bond. There are two more water molecules that nestle at vacant space formed by Glu166, Pro168, and MPI‐148's P3 *tert*‐butylglycine. Although just one of them forms a hydrogen bond with Gln166 side chain, both water molecules likely contribute to stabilizing the complex formation.

Around S1, MPI‐148's P1 lactam fits neatly at the pocket and is surrounded by Phe140, Asn142, Ser144, His163, Glu166, and His172 (Figure 3B). This seamless binding leaves very little space for chemical maneuver for improved binding. Besides forming a hydrogen bond network, the four water molecules around S1, S1’, and the S1–S2 bridging space also fill the vacancy left by the inhibitor. At S2, the spirocyclohexyl group is surrounded by side chains from residues His41, Cys44, Met49, Pro52, Tyr54, and Gln189 and backbones of aa 187–189. There is 5.0 Å distance between Pro52 and the spirocyclohexyl group, indicating a small vacancy left in M^Pro^ for possible development of improved potency S2 binders by taking advantage of this leftover hydrophobic pocket. Chemical innovation will be needed for this purpose. An interesting structural feature that has so far been only observed in M^Pro^‐MPI‐148 is the close distance of 3.9 Å between an inhibitor's S2 binder and Cys44. MPI‐148 is structurally similar to NMV, with the only difference at the P2 position. Its spiro P2 residue is significantly bigger than NMV's P2 bicycle. Compared to NMV, MPI‐148 pushes Met49 toward the solvent, which consequently changes the small aa 46–51 helix to adopt a loop structure to leave space to accommodate MPI‐148's spiro cyclohexyl group and a rotated thiol group by about 60° from Cys44 (Figure S6). This structural rearrangement allows direct van der Waals interactions between Cys44 and MPI‐148, making the design of new inhibitors with covalent access to Cys44 possible. How exactly this can be achieved will require significant chemical innovation and is beyond the scope of the current work. Except for the significant difference observed at S2, MPI‐148 binds at S3 and S4 almost identically to NMV by using identical chemical moieties. Its *N*‐terminal trifluoroacetyl group nestles at S4 by forming van der Waals interactions with side chains of Met165, Leu167, Pro168, and Gln192. Its binding at S4 is not as deep as what was observed for MPI‐136, leaving space for designing new inhibitors.

Transient expression of M^Pro^ in HEK293T cells induces cell death, an effect that can be counteracted by cell‐permeable inhibitors [33]. Leveraging this observation, we previously developed a live‐cell assay to quantify intracellular M^Pro^ inhibitors [34]. In this assay, HEK293T cells are transiently transfected to express M^Pro^‐eGFP and then incubated with test inhibitors. Potent and cellularly permeable inhibitors suppress M^Pro^‐induced cell death, leading to cell survival and increased overall eGFP expression. The eGFP fluorescence is then quantified to determine cellular EC_50_ values. Seven developed nitrile‐containing NMV analogs with enzyme inhibition IC_50_ around or below 100 nM were subjected to this assay to evaluate their cellular potency. Determined EC_50_ values are summarized in Table 4. MPI‐139, MPI‐150, and MPI‐151 showed cellular potency worse than NMV, indicating either poor cellular permeability and/or serving as P‐gp substrates for efflux from cells. Four inhibitors showed cellular potency better than nirmatrelvir. They were subjected to antiviral potency tests in Vero E6 cells using the SARS‐CoV‐2 strain USA_WA1/2020. Determined antiviral EC_50_ values are listed in Table 4 as well. All four inhibitors displayed stronger antiviral effects than NMV, with MPI‐152 as the most potent, showing an EC_50_ of 96 nM. Although information from enzyme inhibition potency can be used to delineate what chemical components favor M^Pro^ binding and assist in further designs, cellular and antiviral activities do not directly correlate with the chemical components these compounds have. For example, MPI‐151 differs from MPI‐152 only at its *N*‐terminal thioamide but showed much lower cellular potency. But MPI‐145 with this thioamide exhibited strong cellular and antiviral potency. Thus, the MPI‐151/MPI‐152 comparison does not support a simple one‐parameter explanation. Instead, it illustrates the complex, multifactorial determinants governing the translation of enzymatic potency into cellular and antiviral efficacy, including cellular permeability, efflux susceptibility, and intracellular stability.

**TABLE 4:** Cellular and antiviral EC_50_ values of selected inhibitors.

ID	**Cellular EC** **_50_** **, μM**	**Antiviral EC** **_50_** **, μM** a
NMV	3.4	1.3
MPI‐139	7.6	—
MPI‐142	0.33	0.38
MPI‐145	0.78	0.17
MPI‐149	1.06	0.13
MPI‐150	9.8	—
MPI‐151	>10	—
MPI‐152	1.03	0.096

^a^Tested in Vero E6 cells using the SARS‐CoV‐2 strain USA_WA1/2020.

To investigate whether the improved cellular potency of selected NMV analogs was associated with reduced susceptibility to P‐gp‐mediated efflux, we evaluated NMV, MPI‐142, and MPI‐145 in the cellular MPro inhibition assay in the presence and absence of the selective P‐gp inhibitor CP‐100356 (2 μM). NMV exhibited a pronounced improvement in potency upon P‐gp inhibition, with its EC_50_ improving approximately fivefold. In contrast, MPI‐142 and MPI‐145 showed substantially smaller improvements of only about 2.2‐fold (Figure S8). These observations indicate that MPI‐142 and MPI‐145 are less sensitive to P‐gp‐mediated transport than NMV and support the notion that reduced efflux contributes to their improved cellular activity.

To obtain preliminary information regarding the developability of the most promising compounds, MPI‐145 and MPI‐152 were evaluated in human liver microsomes. Both compounds displayed comparable metabolic stability with half‐lives of 26.5 and 26.4 min, respectively (Figure S9). These results indicate that the improved cellular and antiviral activities of these compounds are achieved without obvious loss of microsomal stability.

In summary, we conducted an SAR campaign to optimize NMV for improved potency in cells expressing P‐gp. Warheads alternative to nitrile that show better potency are aldehyde and aza‐dichloroacetyl groups. The search for favorable S4 binders led to the identification of a thioamide that leads to better enzyme inhibition potency. Searching through alternative S2 and S3 binders and their combination with alternative S4 binders resulted in inhibitors with better potency than NMV as well. Two M^Pro^‐inhibitor structures were determined by X‐ray crystallography. One structure reveals a rearranged S2 that allows the inhibitor to reach deep to form close contacts with Cys44. This new structural observation indicates that it is possible to design covalent inhibitors by directly reacting with Cys44. Several inhibitors with strong enzyme inhibition potency were tested for their cellular potency in engaging ectopically expressed M^Pro^ and antiviral potency in Vero E6 cells. Results showed that four inhibitors possessed better cellular and antiviral potency than NMV. Additional studies with the selective P‐gp inhibitor CP‐100356 demonstrated that representative optimized analogs were less sensitive to P‐gp inhibition than NMV, supporting reduced P‐gp‐mediated efflux as an important contributor to their improved cellular activity.

## Author Contributions

**Benjamin W.** **Neuman**, **Shiqing Xu**, and **Wenshe Ray Liu**: designed the project. **Yugendar R. Alugubelli**, **Veerabhadra R. Vulupala**, **Kai S. Yang**, **Kaustav Khatua**, **Lauren R. Blankenship**, **Sathish Kumar**, **Demonta Coleman**, **Xuejiao Guo**, **Zhi Zachery Geng**, **Chia‐Chuan D. Cho**, and **Banumathi Sankaran**: conducted experiments. The manuscript was written through contributions of all authors. All authors have approved to the final version of the manuscript.

## Funding

This study was supported by the Office of Extramural Research, National Institutes of Health (R35GM145351, R21AI164088, R21EB032983, F31CA26883, P30GM124169), Welch Foundation (A‐1715, A‐2174), U.S. Department of Defense (DE‐AC02‐05CH11231), Cancer Prevention and Research Institute of Texas (RP210043).

## Conflicts of Interest

The authors declare no conflicts of interest.

## Supporting information

The expression and crystallization of M^Pro^; the structural determination of M^Pro^ bound with all inhibitors; the synthesis and characterization of all newly developed inhibitors; the determination of enzymatic IC_50_, and cellular EC_50_ to inhibit ectopically expressed M^Pro^ in 293T cells; in vitro antiviral tests for SARS‐CoV‐2; Tables S1 and S2 show all parameters for the X‐ray crystallography analysis of M^Pro^ bound with nine inhibitors; Figures S1–S6 show electron density maps of M^Pro^‐MPI‐136 and M^Pro^‐MPI‐148 crystal structures at active sites, their analysis, and comparison with M^Pro^‐NMV; Finally determined structures have been deposited into the Protein Data Bank with the entry codes as 9BQP (MPI‐136), 9BQN (MPI‐148) (pdf).

## Data Availability

The data that support the findings of this study are openly available in the Protein Data Bank at https://www.rcsb.org/, reference numbers 9BQP and 9BQN.
