# Supplementary material for: Exploration of Nirmatrelvir Derivatives as Optimized SARS‐CoV‐2 Antivirals
Source: ChemMedChem. 2026 Aug 11;21(15):e70424. doi: 10.1002/cmdc.70424 (PMC13461302; doi:10.1002/cmdc.70424)
Supplement: Supplementary file 1 [file CMDC-21-e70424-s001.pdf]

Supplementary Information for

Exploration of Nirmatrelvir Derivatives as Optimized SARS-CoV-2 Antivirals

Yugendar R. Alugubelli[a‡], Veerabhadra R. Vulupala[a,‡], Kai S. Yang[a], Kaustav Khatua[a, ‡], Lauren R. Blankenship[a], Sathish Kumar[b], Demonta Coleman[a], Xuejiao Guo[a], Zhi Zachary Geng[a], Chia-Chuan D. Cho[a], Banumathi Sankaran[c], Benjamin W. Neuman[b,d,e,*], Shiqing Xu[a,e,*], and Wenshe Ray Liu[a,e,f,g,h,*]

[a]Texas A&M Drug Discovery Center and Department of Chemistry, College of Arts and Sciences, Texas A&M University, College Station, TX 77843, United States
[b] Department of Biology, College of Arts and Sciences, Texas A&M University, College Station, TX 77843, TX, USA
[c]Molecular Biophysics and Integrated Bioimaging, Berkeley Center for Structural Biology, Laurence Berkeley National Laboratory, Berkeley, CA 94720, United States
[d]Texas A&M Global Health Research Complex, Texas A&M University, College Station, TX 77843, USA
[e]Department of Molecular Pathogenesis and Immunology, College of Medicine, Bryan, TX 77807, USA
[e]Department of Pharmaceutical Sciences, Irma Lerma Rangel School of Pharmacy, Texas A&M University, College Station, TX 77843, United States
[f]Institute of Biosciences and Technology and Department of Translational Medical Sciences, School of Medicine, Texas A&M University, Houston, TX 77030, United States
[g]Department of Biochemistry and Biophysics, College of Agriculture and Life Sciences, Texas A&M University, College Station, TX 77843, United States
[h]Department of Cell Biology and Genetics, College of Medicine, Texas A&M University, College Station, TX 77843, United States
[‡]Contributed equally to the paper
*Corresponding authors: Benjamin W. Neuman: bneuman@tamu.edu; Shiqing Xu: shiqing.xu@tamu.edu; Wenshe Ray Liu: wsliu2007@tamu.edu.

Supplementary Materials and Methods

Expression and Purification of SARS-CoV-2 M^{Pro}

The expression plasmid pET28a-His-SUMO-CoV-2 M^{Pro} was constructed in a previous study (*43*). We used this construct to transform *E. coli* BL21(DE3) cells. A single colony grown on an LB plate with 25 µg/mL kanamycin was picked and grown in 5 mL LB media supplemented with 25 µg/mL kanamycin overnight. We inoculated this overnight culture to 6 L 2YT media with 25 µg/mL kanamycin. Cells were grown to OD_{600} as 0.8. At this point, we added 1 mM IPTG to induce the expression of His-SUMO-CoV-2 M^{Pro}. Induced cells were let grown for 3 h and then harvested by centrifugation at 12,000 rpm, 4 °C for 30 min. We resuspended cell pellets in 150 mL lysis buffer (20 mM Tris-HCl, 100 mM NaCl, 10 mM imidazole, pH 8.0) and lysed the cells by sonication on ice. We clarified the lysate by centrifugation at 16,000 rpm, 4 °C for 30 min. We decanted the supernatant and mixed with Ni-NTA resins (GenScript). We loaded the resins to a column, washed the resins with 10 volumes of lysis buffer, and eluted the bound protein using elution buffer (20 mM Tris-HCl, 100 mM NaCl, 250 mM imidazole, pH 8.0). We exchanged buffer of the elute to another buffer (20 mM Tris-HCl, 100 mM NaCl, 10 mM imidazole, 1 mM DTT, pH 8.0) using a HiPrep 26/10 desalting column (Cytiva) and digested the elute using 10 units SUMO protease overnight at 4 °C. The digested elute was subjected to Ni-NTA resins in a column to remove His-tagged SUMO protease, His-tagged SUMO tag, and undigested His-SUMO-CoV-2 M^{Pro}. We loaded the flow-through onto a Q-Sepharose column and purified CoV-2 M^{Pro} using FPLC by running a linear gradient from 0 to 500 mM NaCl in a buffer (20 mM Tris-HCl, 1 mM DTT, pH 8.0). Fractions eluted from the Q-Sepharose column was concentrated and loaded onto a HiPrep 16/60 Sephacryl S-100 HR column and purified using a buffer containing 20 mM Tris-HCl, 100 mM NaCl, 1 mM DTT, and 1 mM EDTA at pH 7.8. The final purified protein was concentrated and stored in a -80 °C freezer.

Characterization of M^{Pro} Inhibition by All Synthesized Inhibitors

For all inhibitors, we conducted the assay using 20 nM M^{Pro} and 10 µM Sub3 (DABCYL-Lys-Thr- Ser-Ala-Val-Leu-Gln-Ser-Gly-Phe-Arg-Lys-Met-Glu-EDANS). We dissolved all inhibitors in DMSO as 10 mM stock solutions. Sub3 was dissolved in DMSO as a 1 mM stock solution and diluted 100 times in the final assay buffer containing 10 mM $Na_xH_yPO_4$, 10 mM NaCl, 0.5 mM EDTA, and 1.25% DMSO at pH 7.6. We incubated M^{Pro} and an inhibitor in the final assay buffer for 30 min before adding the substrate to initiate the reaction catalyzed by M^{Pro}. The production format was monitored in a fluorescence plate reader with excitation at 336 nm and emission at 490 nm. We calculated the initial rate according to the fluorescent intensity in the first 5 min by linear regression, which was then normalized according to the initial rate of positive and negative controls. We calculated the initial rate according to the fluorescent intensity in the first 5 min by linear regression, which was then normalized according to the initial rate of positive and negative controls.

Cellular Potency Tests for Selected Inhibitors

HEK293T cells were maintained in high-glucose DMEM supplemented with GlutaMAX and 10% fetal bovine serum in 10 cm culture plates at 37 °C and 5% CO_2 until reaching 80%–90% confluency. Cells were transfected with the pLVX-MPro-eGFP-2 plasmid (43) using 8 µg of plasmid DNA in 500 µL opti-MEM per transfection. Transfections were performed using polyethyleneimine (30 µg/mL) for standard cellular assays, whereas Lipofectamine 2000 was utilized for the P-gp inhibition experiments. The transfection mixture was incubated with cells overnight. On the following day, the medium was removed, and cells were washed with PBS before enzymatic detachment using 0.05% trypsin-EDTA.

The dissociated cells were resuspended in their original growth medium, and the density was adjusted to 5×10^5 cells/mL.

For standard cellular EC_{50} determinations, 500 µL of the cell suspension was seeded into each well of a 48-well plate, followed by the addition of 100 µL of drug solution prepared in growth media. To evaluate the influence of P-glycoprotein (P-gp) efflux on intracellular potency, an additional set of experiments was performed using the P-gp inhibitor CP-100356. For these inhibition studies, cells were pre-treated with CP-100356 (2 µM final concentration) for 45 minutes prior to compound addition. Subsequently, test inhibitors were co-administered in media containing 2 µM CP-100356 to maintain continuous transporter blockade.

Cells were incubated under standard culture conditions for 72 hours with varying concentrations of inhibitors (with or without 2 µM CP-100356) before flow cytometry analysis. Post-incubation, cells were resuspended in 500 µL PBS and centrifuged at 800 rpm for 5 minutes. The supernatant was discarded, and cell pellets were resuspended in 200 µL PBS. eGFP fluorescence was analyzed using a Cytoflex Beckman Flow Cytometer, with cells sorted based on side scatter (SSC-A, SSC-H) and forward scatter (FSC-A). Gating was performed sequentially using SSC-A/FSC-A followed by SSC-A/SSC-H. eGFP fluorescence was excited using a 488 nm blue laser, and emissions were recorded at FITC-A (525 nm).

All processed data were plotted and fitted to a four-parameter Hill equation using GraphPad Prism 9.0 to determine EC_{50} values. To quantify P-gp recognition, the relative shift in cellular potency was calculated as the ratio of the EC_{50} value obtained in the absence of CP-100356 to the EC_{50} value obtained in the presence of CP-100356.

Antiviral Potency Tests for Selected Inhibitors

A549-hACE2 cells (BEI Resources, NR53821) were cultured in a 37ºC incubator with 5% CO_2 in DMEM supplemented with 10% fetal bovine serum, 1× antibiotic/antimycotic and puromycin dihydrochloride. SARS-CoV-2 strain WA1 (BEI Resources; USA-WA1/2020; NR52281) was cultured on Vero E6-ACE2-TMPRSS2. Infectious titer was determined by tissue culture infectious dose 50% ($TCID_{50}$) on Vero E6-ACE2-TMPRSS2 cells. Cells were inoculated at a multiplicity of 0.1 $TCID_{50}$ unit per cell, incubated for 1h at 37ºC to allow for virus adsorption, rinsed three times with phosphate buffered saline pH 7 to remove unbound inoculum, and treated by addition of DMEM with 10% fetal bovine serum and 1× antibiotic/antimycotic containing various concentrations of potential antivirals. Small aliquots of medium were collected at 48h and 72 h after inoculation, and the amount of virus growth was titrated using a Luna One-Step RT175 qPCR (NEB, Ipswich, MA, US) with the N1 primer set targeting the nucleoprotein gene of the virus, calibrated to a standard curve of inactivated viral RNA (BEI Resources, NR-52285).

X-Ray Crystallography Analysis of M^{Pro}-Inhibitor Complexes

The production of crystals of M^{pro}-inhibitor complexes was following the previous protocols (*32*). Data were collected on a Bruker Photon II detector or the Advanced Light Source (ALS) beamline 5.0.2 using a Pilatus3 S 6M detector. The diffraction data were indexed, integrated and scaled with iMosflm or PROTEUM3. All the structures were determined by molecular replacement using the structure model of the free enzyme of the SARS-CoV-2 M^{pro} [Protein Data Bank (PDB) ID code 7JPY] as the search model using Phaser in the Phenix package. *JLigand* and *Sketcher* from the CCP4 suite were employed for the generation of PDB and geometric restraints for the inhibitors. The inhibitors were built into the Fo-Fc

density by using *Coot*. Refinement of all the structures was performed with Real-space Refinement in Phenix. Details of data quality and structure refinement are summarized in Tables S1-S2.

Supplementary Figures

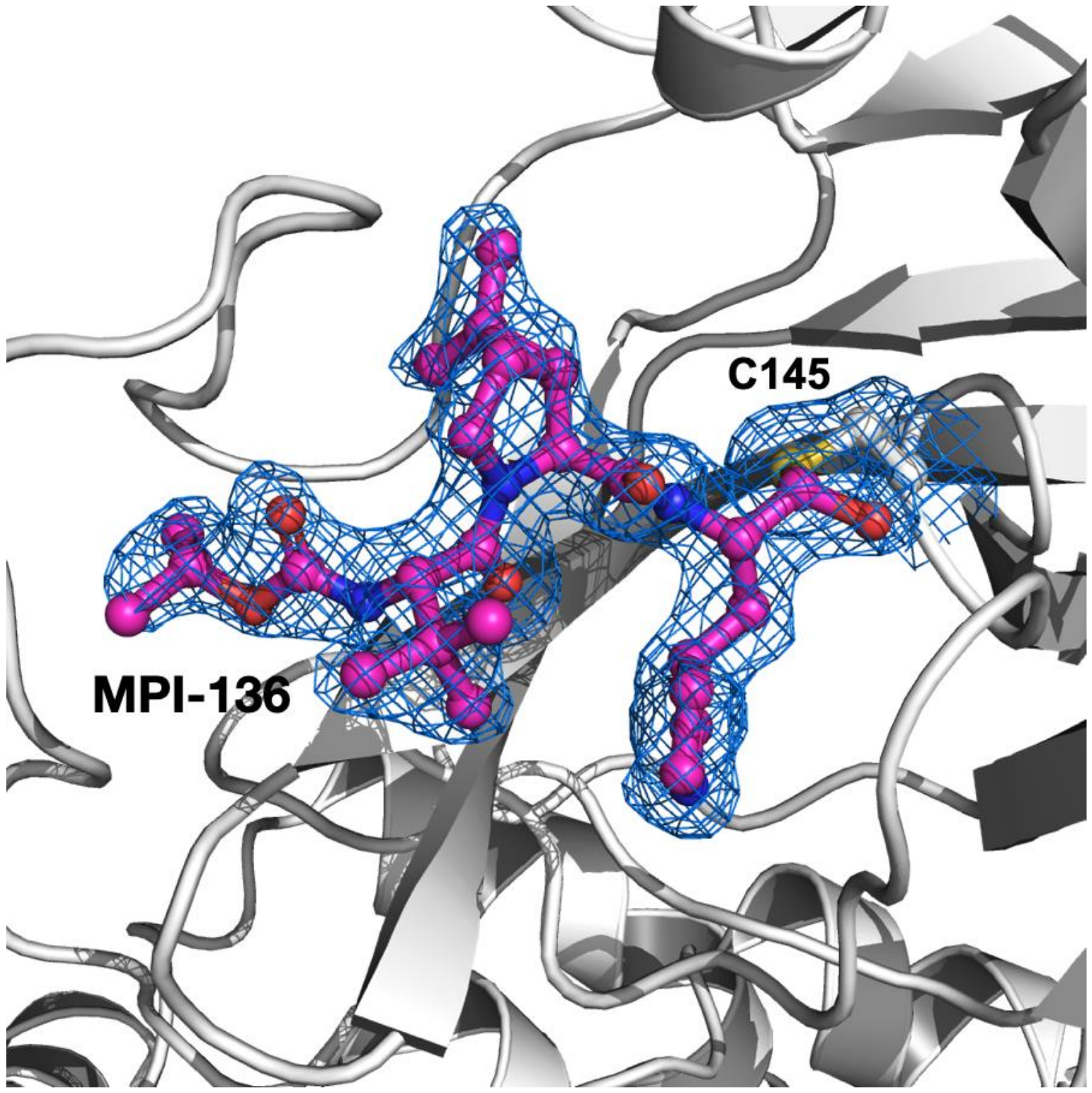

Figure S1: 2Fo-Fc electron density maps around MPI-136 and Cys145 side chain in the M^{Pro}-MPI-136 complex that is contoured at the 1σ level.

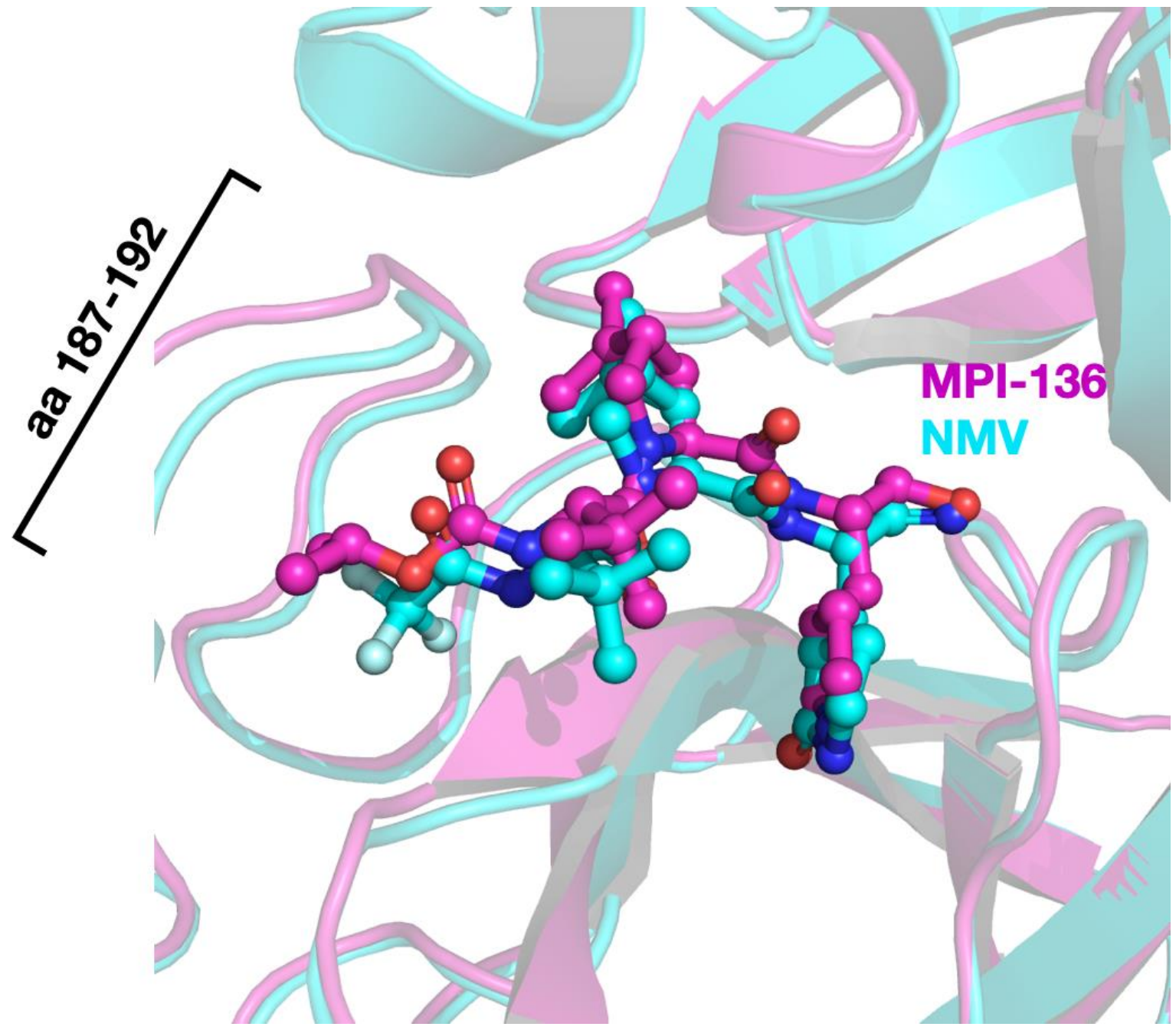

Figure S2: Superimposition of M[Pro]-MPI-136 (PDB: 9BQP) over M[Pro]-NMV (PDB: 7RFS) at the active site.

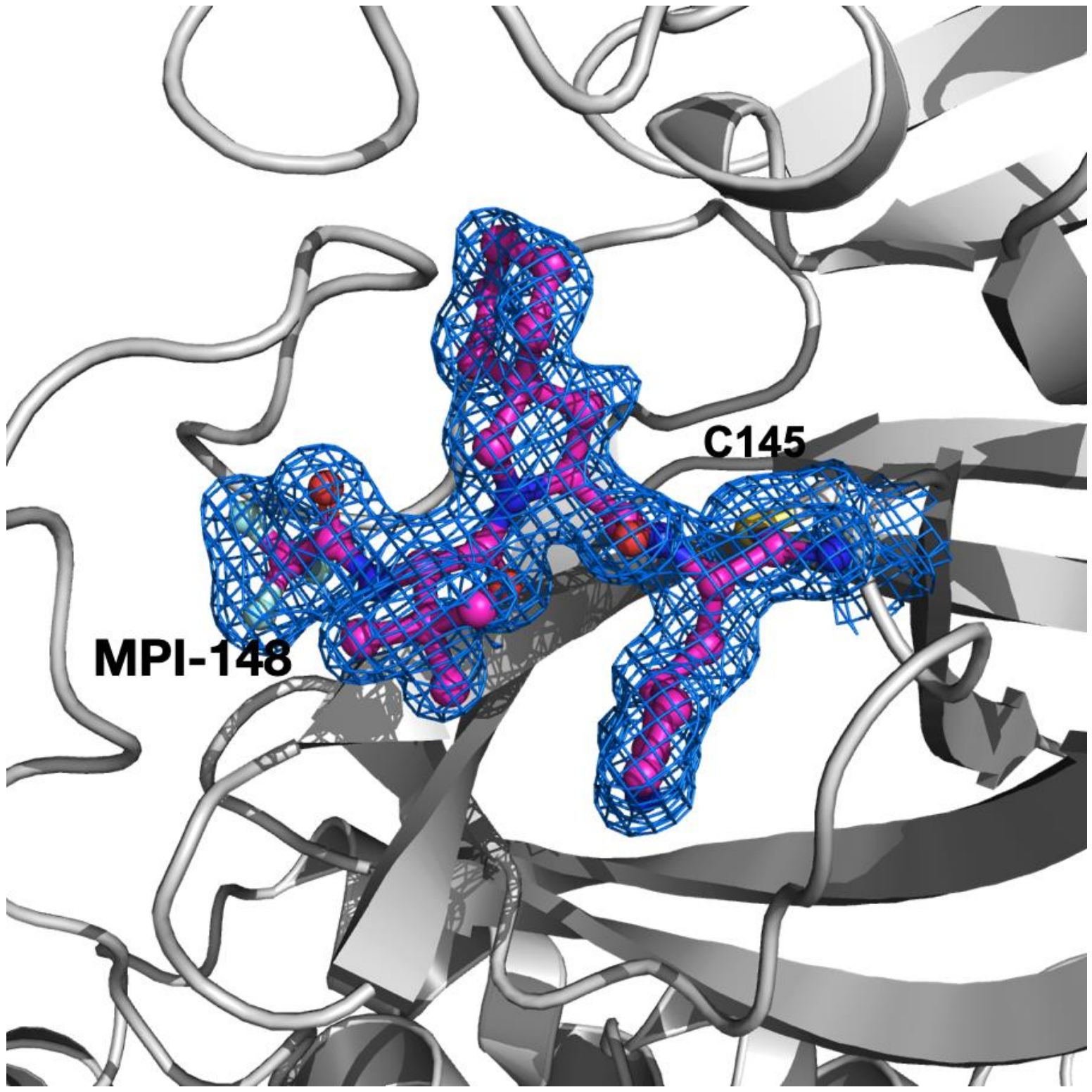

Figure S3: 2Fo-Fc electron density maps around MPI-148 and Cys145 side chain in the M^{Pro}-MPI-148 complex that is contoured at the 1σ level.

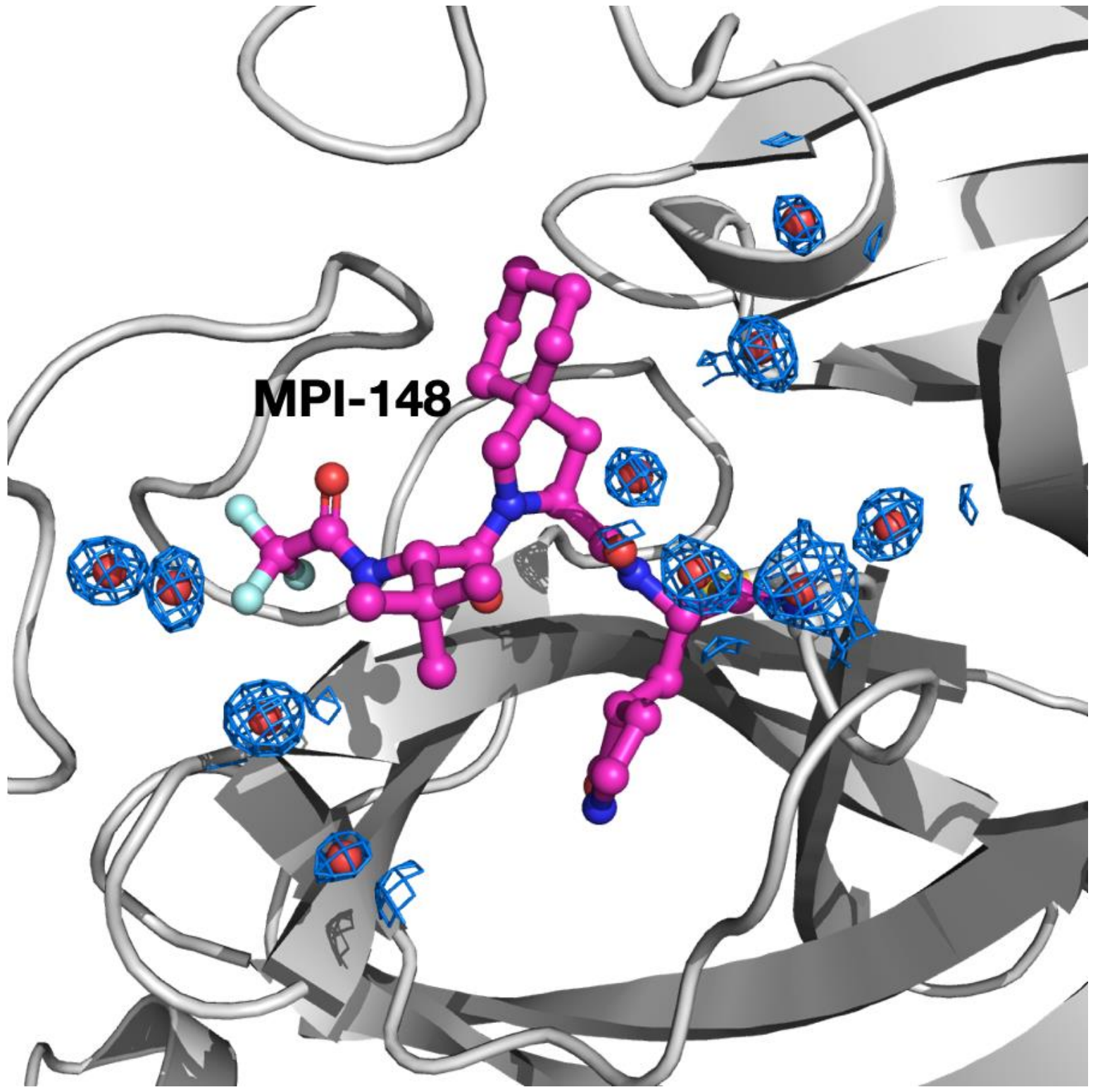

Figure S4: 2Fo-Fc electron density maps around water molecules in the M^{Pro}-MPI-148 complex that is contoured at the 1σ level.

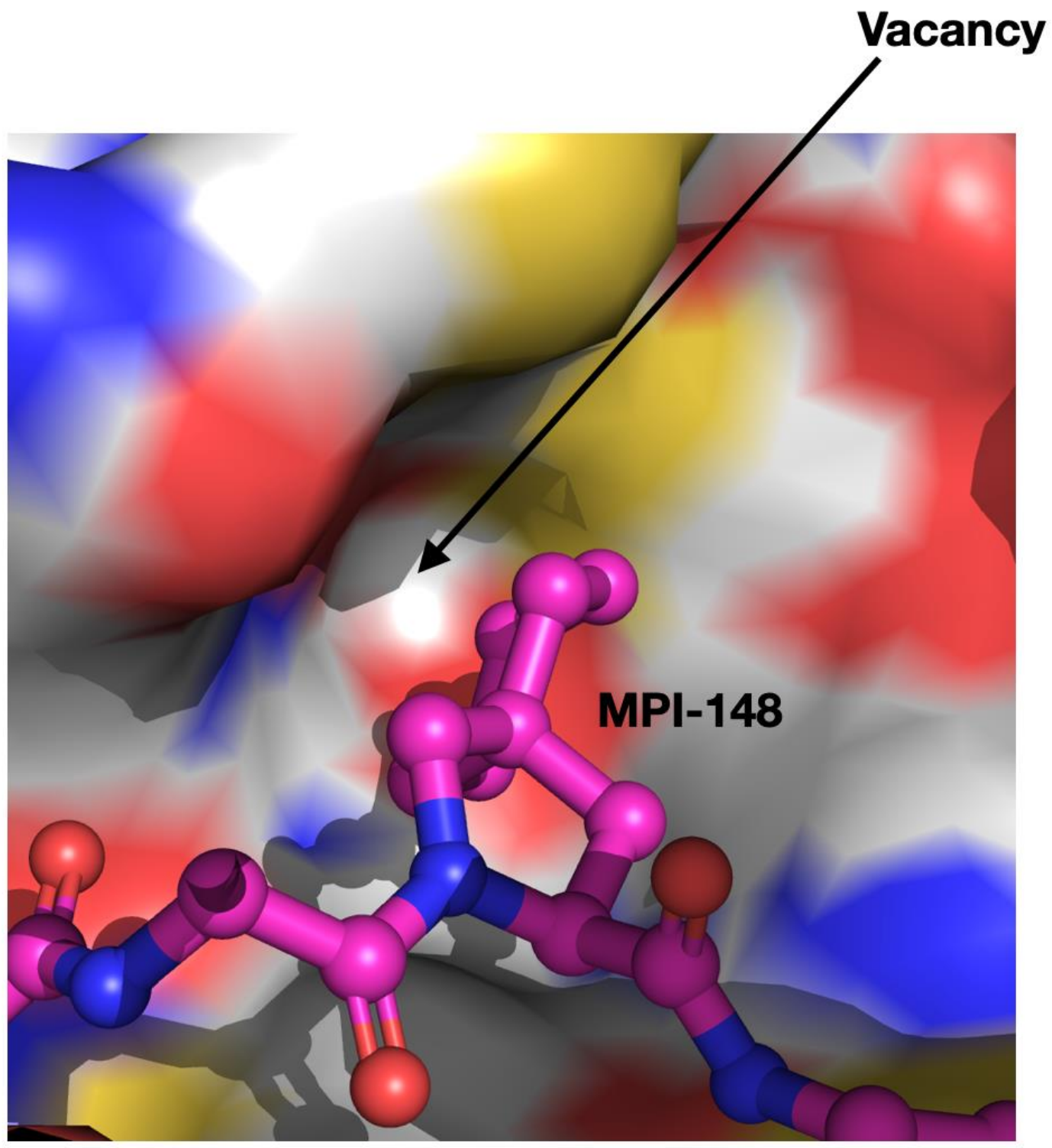

Figure S5: Small vacancy left in M^{Pro} after MPI-148 binding.

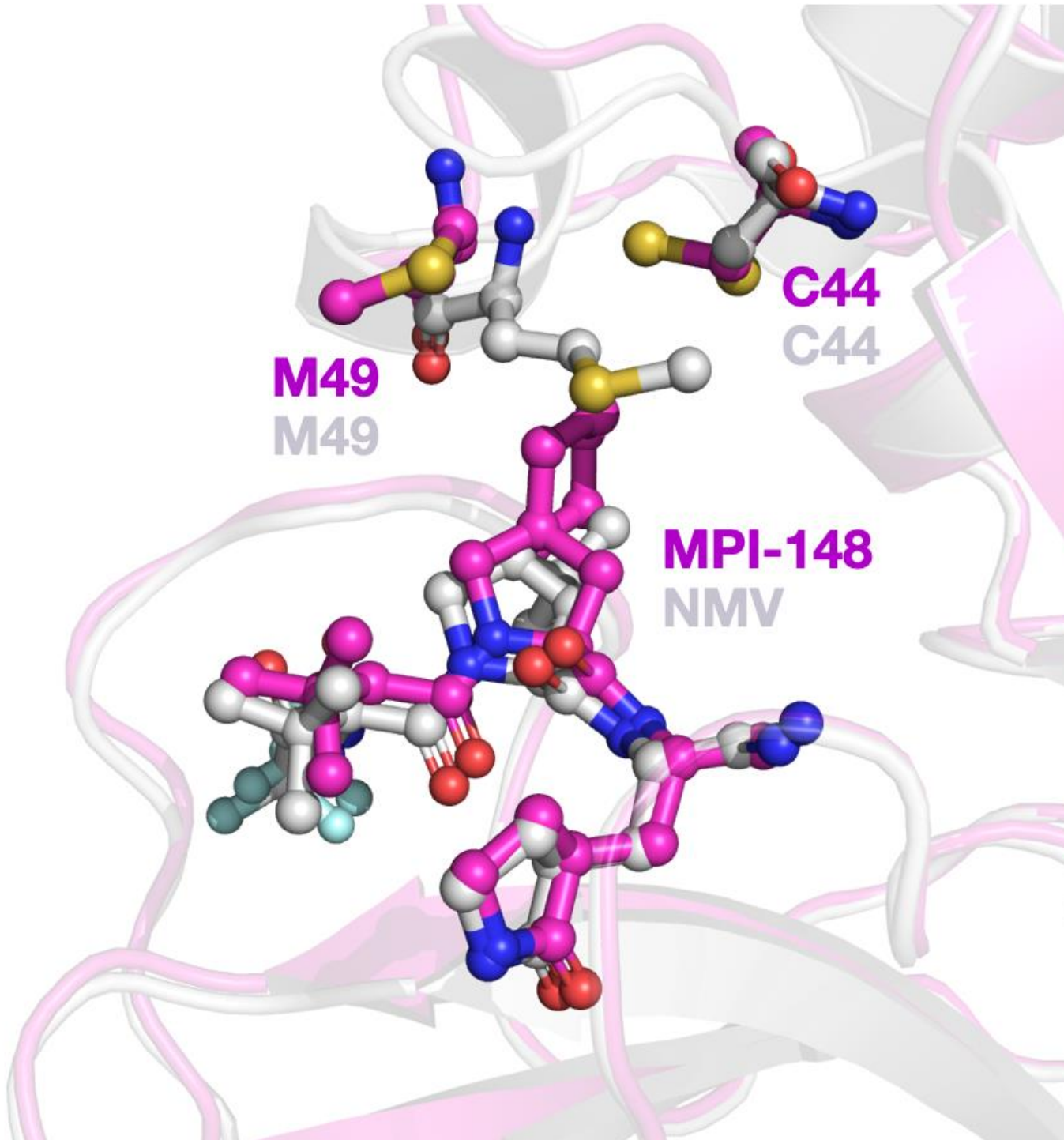

Figure S6: Superimposition of M^{Pro}-MPI-148 over M^{Pro}-NMV at the active site.

Supplementary Tables

Table S1: Data collection and refinement statistics for the M^{Pro}-MPI-136 complex

PDB Entry	9BQP
Resolution range	24.24 - 2.0 (2.071 - 2.0)
Space group	I 1 2 1
Unit cell	55.4764 83.277 88.2668 90 97.3338 90
Total reflections	150954 (12445)
Unique reflections	26225 (2660)
Multiplicity	5.8 (4.7)
Completeness (%)	97.17 (98.70)
Mean I/sigma(I)	12.42 (2.68)
Wilson B-factor	21.46
R-merge	0.08221 (0.4973)
R-meas	0.08915 (0.559)
R-pim	0.03279 (0.2473)
CC1/2	0.997 (0.937)
CC*	0.999 (0.984)
Reflections used in refinement	26194 (2655)
Reflections used for R-free	1273 (132)
R-work	0.2229 (0.3276)
R-free	0.2625 (0.4057)
CC(work)	0.954 (0.839)
CC(free)	0.928 (0.737)
Number of non-hydrogen atoms	2636
macromolecules	2363
ligands	35
solvent	238
Protein residues	306
RMS(bonds)	0.010
RMS(angles)	1.08
Ramachandran favored (%)	98.36
Ramachandran allowed (%)	1.64
Ramachandran outliers (%)	0.00
Rotamer outliers (%)	0.00
Clashscore	3.82
Average B-factor	29.56
macromolecules	29.22
ligands	27.59
solvent	33.27
Number of TLS groups	1

Table S2: Data collection and refinement statistics for the M^{Pro}-MPI-117 complex

PDB Entry	9BQN
Resolution range	24.34 - 1.85 (1.916 - 1.85)
Space group	I 1 2 1
Unit cell	51.6357 80.7727 89.2751 90 96.9992 90
Total reflections	218688 (15811)
Unique reflections	31070 (3118)
Multiplicity	7.0 (5.1)
Completeness (%)	99.14 (99.17)
Mean I/sigma(I)	8.79 (1.08)
Wilson B-factor	22.32
R-merge	0.119 (1.082)
R-meas	0.1277 (1.208)
R-pim	0.04561 (0.5318)
CC1/2	0.997 (0.728)
CC*	0.999 (0.918)
Reflections used in refinement	30832 (3095)
Reflections used for R-free	1557 (175)
R-work	0.2157 (0.3167)
R-free	0.2382 (0.3518)
CC(work)	0.948 (0.791)
CC(free)	0.928 (0.692)
Number of non-hydrogen atoms	2628
macromolecules	2367
ligands	37
solvent	224
Protein residues	306
RMS(bonds)	0.008
RMS(angles)	1.04
Ramachandran favored (%)	97.37
Ramachandran allowed (%)	2.63
Ramachandran outliers (%)	0.00
Rotamer outliers (%)	0.38
Clashscore	4.24
Average B-factor	28.27
macromolecules	27.85
ligands	26.14
solvent	33.14
Number of TLS groups	1

Supplementary Synthesis Details

The Synthesis of MPIs

All reagents and solvents for the synthesis were purchased from commercial sources and used without purification. All glassware was flame-dried prior to use. Thin-layer chromatography (TLC) was carried out on aluminum plates coated with 60 F254 silica gel. TLC plates were visualized under UV light (254 or 365 nm) or stained with 5% phosphomolybdic acid. Normal phase column chromatography was carried out using a Yamazen Small Flash AKROS system. NMR spectra were recorded on a Bruker AVANCE Neo 400 MHz spectrometer in specified deuterated solvents.

Supplementary Figures

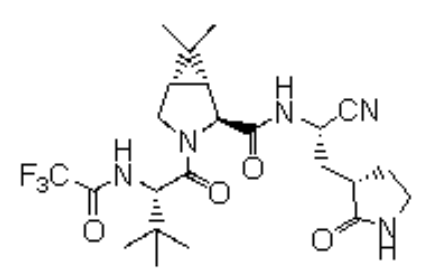

Nirmatrelvir

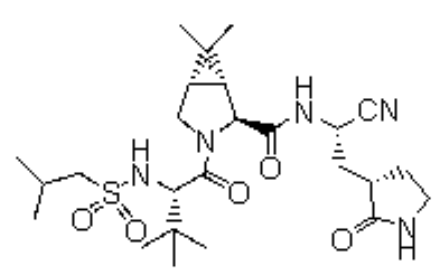

MPI138

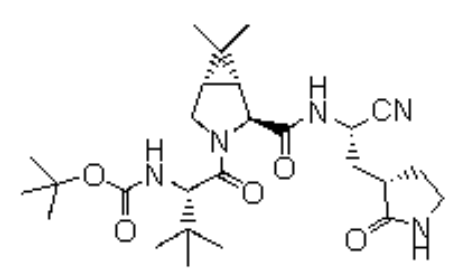

MPI143

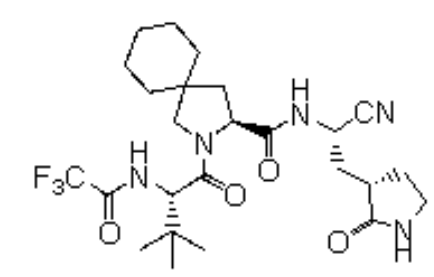

MPI148

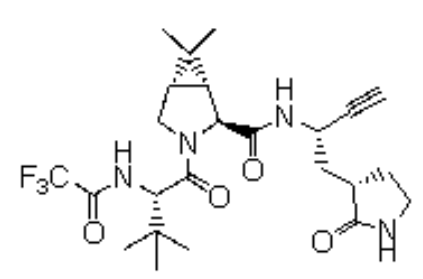

MPI134

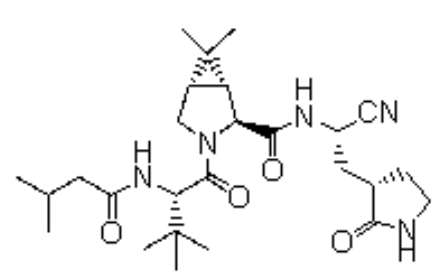

MPI139

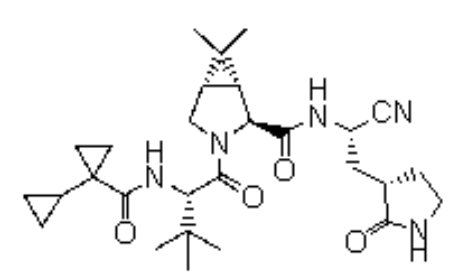

MPI144

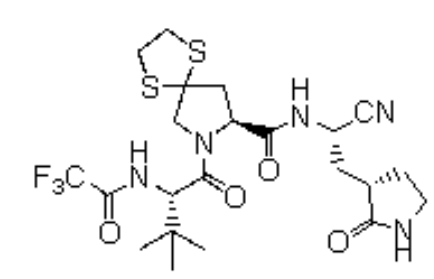

MPI149

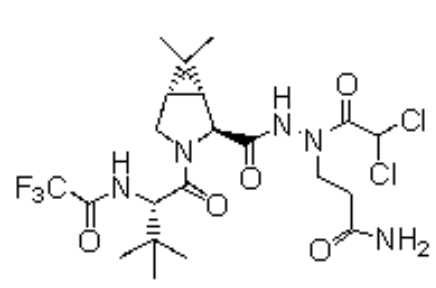

MPI135

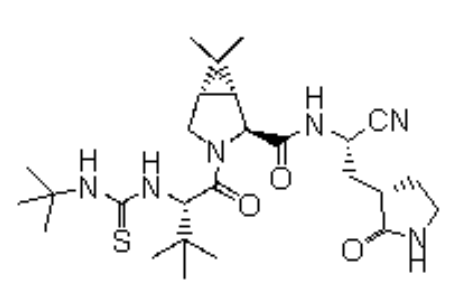

MPI140

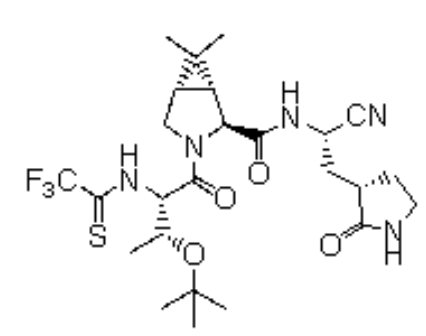

MPI145

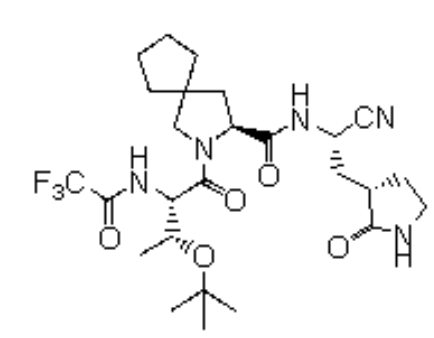

MPI150

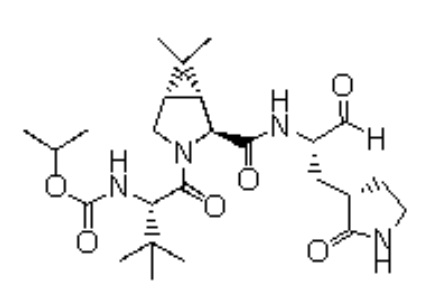

MPI136

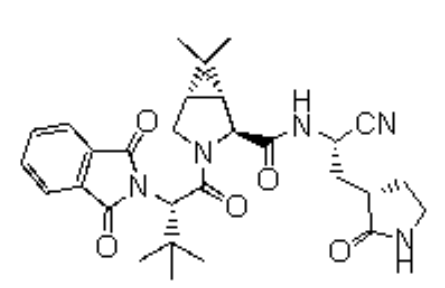

MPI141

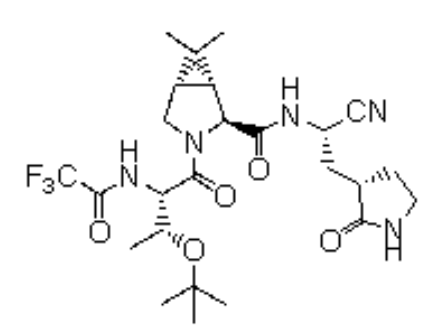

MPI146

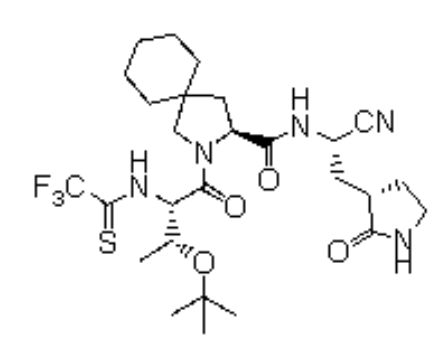

MPI151

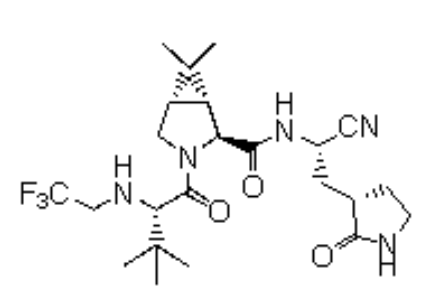

MPI137

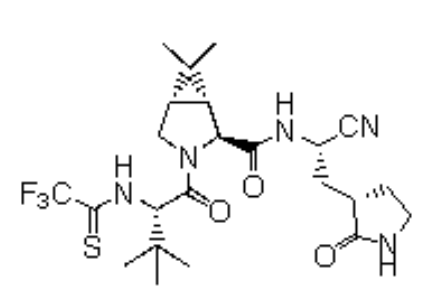

MPI142

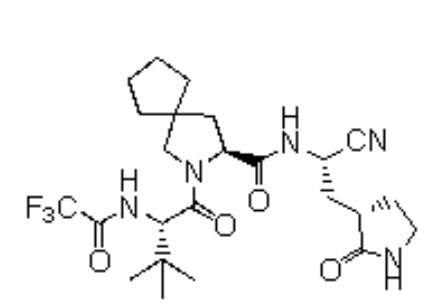

MPI147

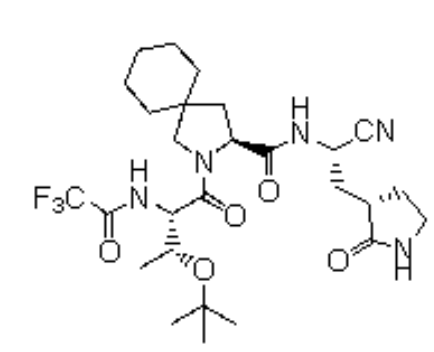

MPI152

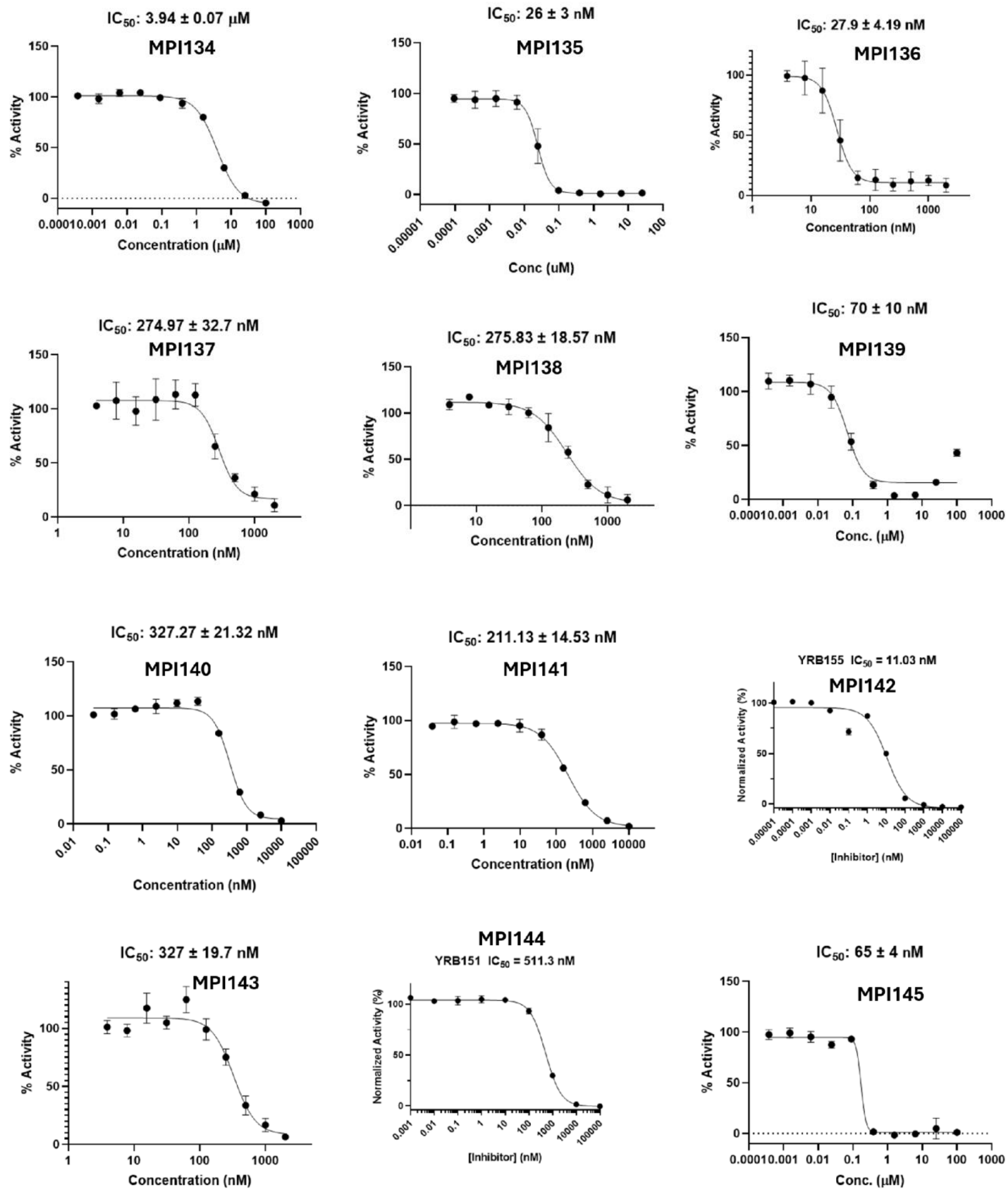
IC50: 3.94 ± 0.07 μM
MPI134
% Activity
Concentration (μM)
IC50: 26 ± 3 nM
MPI135
% Activity
Conc (uM)
IC50: 27.9 ± 4.19 nM
MPI136
% Activity
Concentration (nM)
IC50: 274.97 ± 32.7 nM
MPI137
% Activity
Concentration (nM)
IC50: 275.83 ± 18.57 nM
MPI138
% Activity
Concentration (nM)
IC50: 70 ± 10 nM
MPI139
% Activity
Conc. (μM)
IC50: 327.27 ± 21.32 nM
MPI140
% Activity
Concentration (nM)
IC50: 211.13 ± 14.53 nM
MPI141
% Activity
Concentration (nM)
YRB155 IC50 = 11.03 nM
MPI142
Normalized Activity (%)
[Inhibitor] (nM)
IC50: 327 ± 19.7 nM
MPI143
% Activity
Concentration (nM)
MPI144
YRB151 IC50 = 511.3 nM
Normalized Activity (%)
[Inhibitor] (nM)
IC50: 65 ± 4 nM
MPI145
% Activity
Conc. (μM)

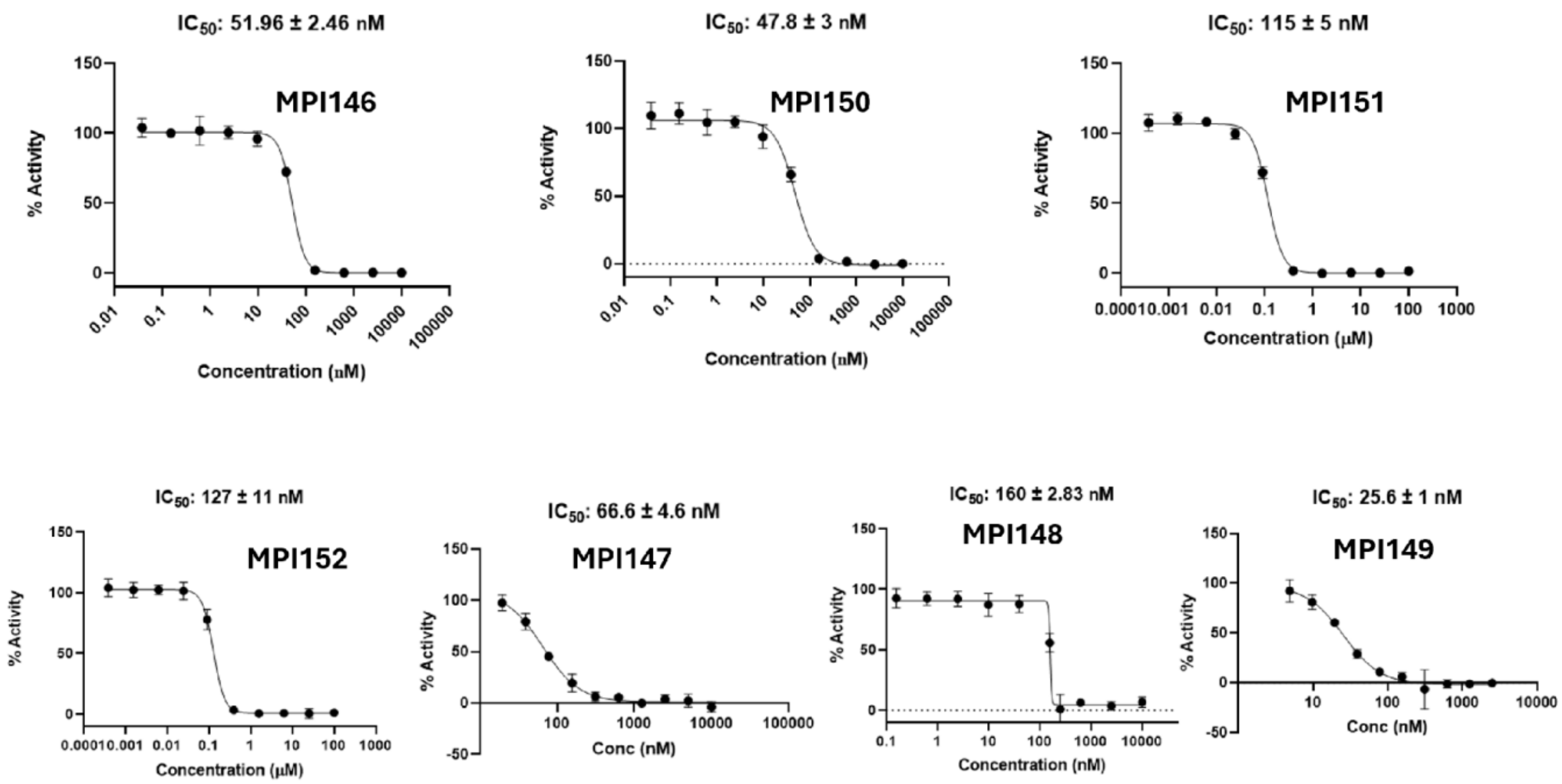

Figure S7. Inhibition curves of compounds on MPro. Triplicate experiments were performed for each compound. For all experiments, 20 or 10 nM MPro was incubated with an inhibitor for 30 min before 10 μM Sub3 was added. The MPro-catalyzed Sub3 hydrolysis rate was determined by measuring linear increase of product fluorescence (Ex: 336 nm/Em: 455 nm) for 5 min.

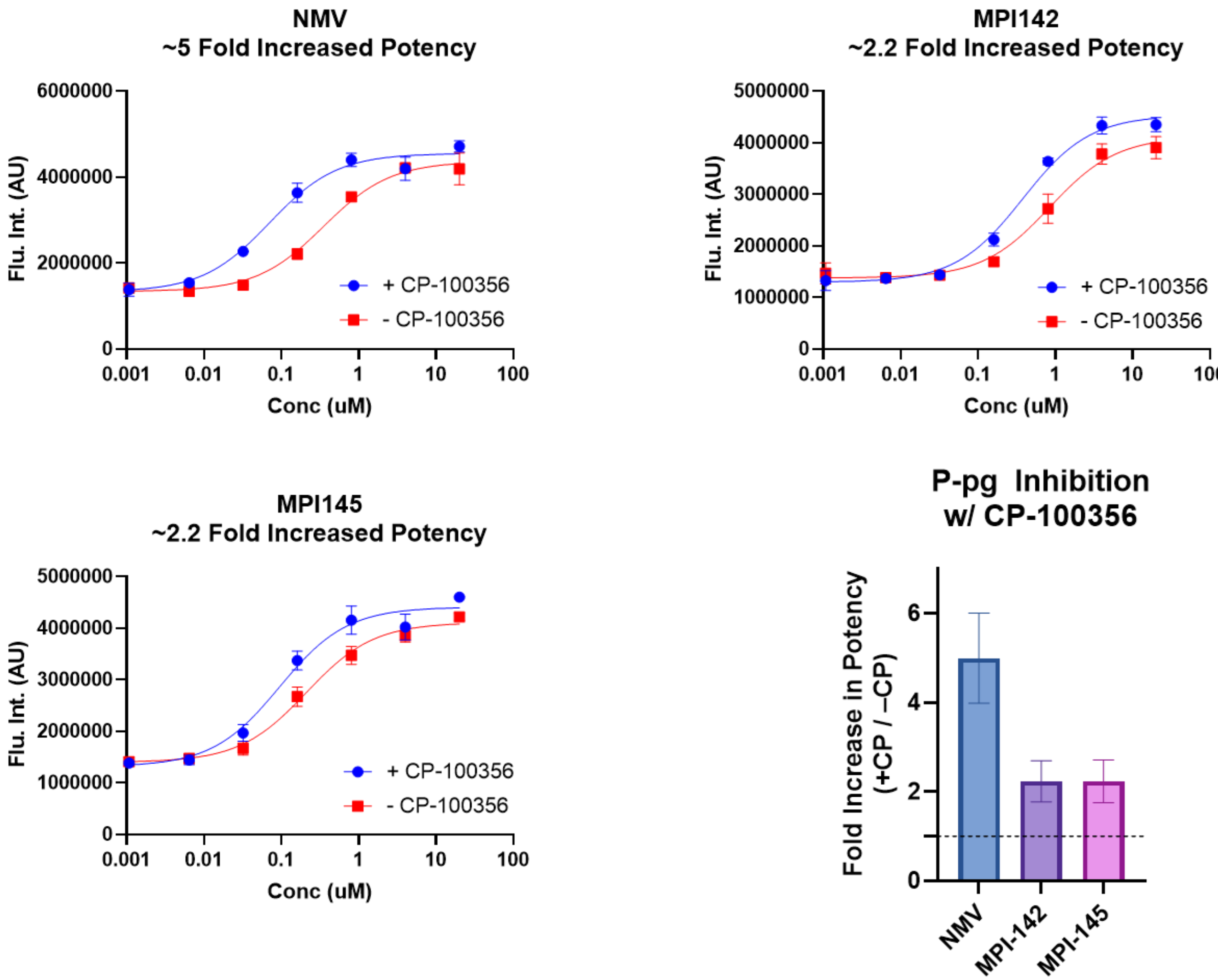

Figure S8. Evaluation of P-glycoprotein (P-gp) mediated efflux on intracellular potency.

(A) Cellular dose-response curves for NMV (control), MPI-142, and MPI-145 in HEK293T cells expressing MPro-eGFP, treated with varying concentrations of inhibitor in the absence (red squares) or presence (blue circles) of the P-gp inhibitor CP-100356 (2 µM). Co-administration of CP-100356 induced a leftward shift in the curves, indicating increased intracellular potency due to transporter blockade. (B) Tabulated EC_{50} values for each condition and the corresponding calculated fold change (ΔEC_{50}), defined as the ratio of EC_{50} without CP-100356 to EC_{50} with CP-100356. (C) Bar graph quantifying the relative fold increase in potency upon P-gp inhibition. The reference line (y = 1.0) represents no shift in potency.

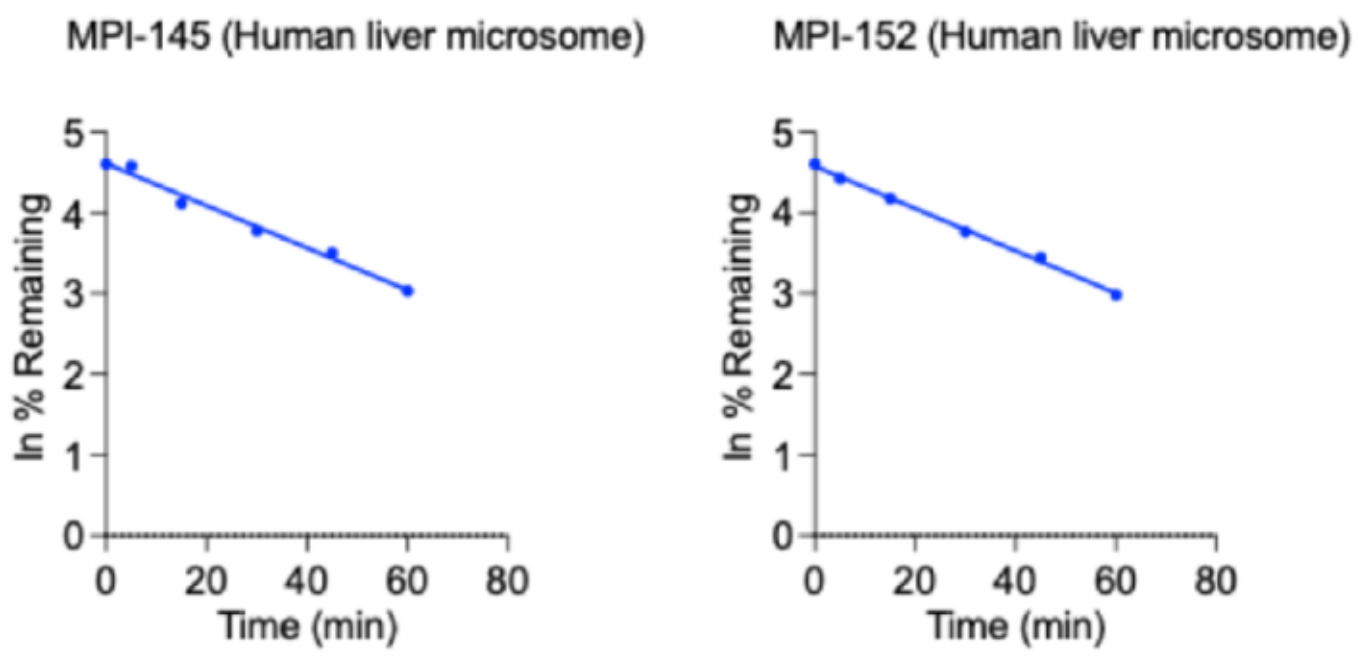

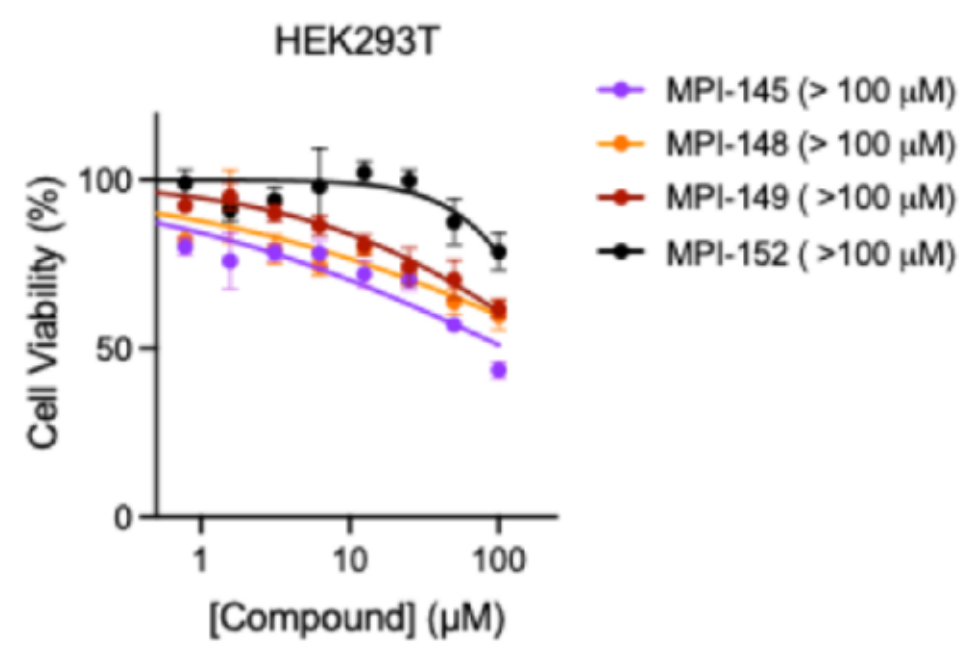

Compound	Half life $t_{1/2}$ (min) in Human liver microsome
MPI-145	26.5
MPI-152	26.4

Figure S9. Determination of human liver microsomal stability for MPI-145 and MPI-152. Representative microsomal stability profiles of MPI-145 and MPI-152 in pooled human liver microsomes. Compounds were incubated with microsomes under metabolic turnover conditions, and the remaining parent compound was quantified by LC–MS/MS at designated time points. First-order kinetic analysis afforded half-life (t½) values of 26.5 min for MPI-145 and 26.4 min for MPI-152.

General procedures:

General procedure A: To a solution of **alcohol** (1 eq.,) in CH_2Cl_2 (12 mL) was added $NaHCO_3$ (4 eq.,) and the Dess-Martin reagent (3 eq.,) was added at 0 °C. The resulting mixture was stirred at rt for 2-3 h. Then the reaction was quenched with a saturated $NaHCO_3$ solution containing 10 % $Na_2S_2O_3$. The layers were separated. The organic layer was then washed with saturated brine solution, dried over anhydrous Na_2SO_4 and concentrated *on vacuum*. The residue was then purified with flash chromatography afford **aldehyde** as solid.

General Procedure B

Boc protected compound was stirred in Trifluoroethanol (5 mL for 1 g) at 0 °C was added TMSCl (1.5 ml for 1 g). The reaction mixture was stirred at rt for 3 h. After completion of the reaction, solvent was concentrated in a vacuum. The residue was used in the next step without further purification.

General Procedure C

To a solution of **d** (1 eq) and **Int.1** (1 eq) in anhydrous DMF was added NMM (4 eq), and the mixture was cooled to 0 °C. HATU (1.2 eq) was added to the solution under 0 °C and then stirred at rt overnight. The reaction mixture was then diluted with ethyl acetate and washed with saturated $NaHCO_3$ solution (two times), 1 M HCl solution (two times), and saturated brine solution (two times) sequentially. The organic layer was dried over anhydrous Na_2SO_4 and then concentrated *in vacuo*. The residue was then purified with flash chromatography (0–10% MeOH in dichloromethane as the eluent) to afford the **compound** as a white solid/gummy solid.

General Procedure D

To a stirred solution of **Amide** (1 eq) in DCM (10 mL) at 0 °C was added Burgess reagent (2.5 eq), and the mixture was stirred at rt for 2 h. The reaction was quenched with saturated $NaHCO_3$ solution (5 mL) and extracted with DCM (2 × 10 mL). The combined organic layer was washed with brine, dried over $MgSO_4$, and concentrated in a vacuum. The residue was then purified with flash chromatography (0–10% MeOH in dichloromethane as the eluent) to afford Nitrile compound as a white solid.

General Procedure E

To a stirred solution of compound **Ester** (1 eq) in THF was added $LiBH_4$ (2.0 M in THF, 5 eq) in several portions at 0 °C under a nitrogen atmosphere. The reaction mixture was stirred at 0 °C for 1 h, allowed to warm up to rt, and then stirred for an additional 2 h. The reaction was quenched by the dropwise addition of 1.0 M HCl (aq.) (1.2 mL) with cooling in an ice bath. The solution was diluted with ethyl acetate and H_2O. The phases were separated, and the aqueous layer was extracted with ethyl acetate (three times). The organic phases were combined, dried over $MgSO_4$, filtered, and concentrated on a rotorvap to give a yellow oily residue. Column chromatographic purification of the residue (2–10% MeOH in CH_2Cl_2 as the eluent) afforded **primary alcohol** as a white solid/gummy solid.

General Procedure F

To a stirred solution of **Boc compound** (1 eq) in 1,4-dioxane at 0 °C was added 4 N HCl (10 eq). The reaction mixture was stirred at rt for 3 h. After completion of the reaction, solvent was concentrated in a vacuum. The residue **compound** was used in the next step without further purification.

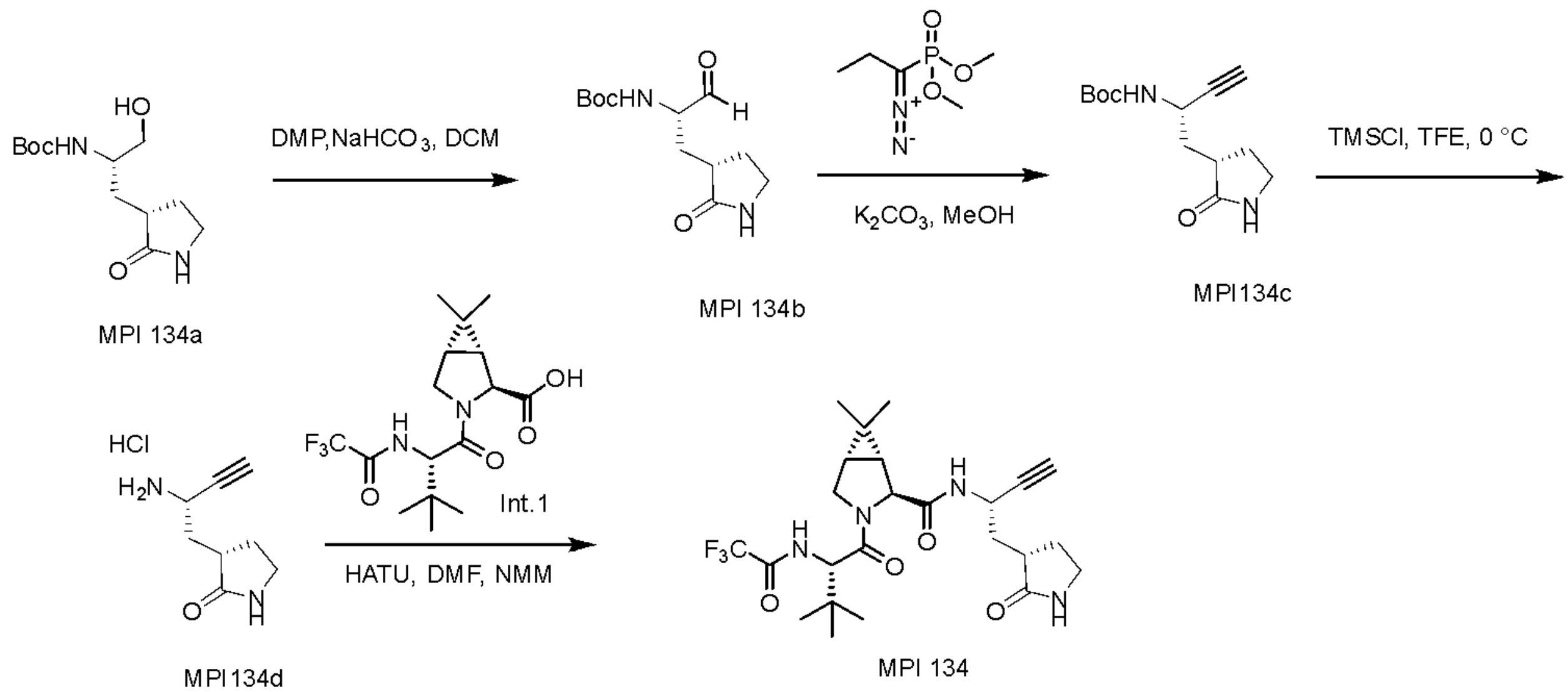

Scheme 1. The synthesis of **MPI134**

tert-butyl ((S)-1-oxo-3-((S)-2-oxopyrrolidin-3-yl)propan-2-yl)carbamate (MPI134b): MPI134b was prepared by using **MPI134a** as a white solid following a general procedure A (yield 70%). ^{1}H NMR (400 MHz, Chloroform-*d*) δ 9.59 (s, 1H), 6.11 (s, 1H), 5.49 (s, 1H), 4.22 (s, 1H), 3.39 (dd, *J* = 8.8, 6.4 Hz, 2H), 2.50 (d, *J* = 6.6 Hz, 1H), 2.08 – 1.82 (m, 2H), 1.57 (s, 9H).

tert-butyl ((S)-1-((S)-2-oxopyrrolidin-3-yl)but-3-yn-2-yl)carbamate (MPI134C): Charge a reaction flask with the aldehyde (1.0 eq) and K_2CO_3 (3 eq) in anhydrous methanol under an argon atmosphere, then add Bestmann–Ohira reagent (1.5 eq). The reaction mixture is stirred at room temperature for 24 h and monitored by TLC. Upon completion, the reaction is quenched with water and extracted with diethyl ether. The combined organic layers are washed with aqueous $NaHCO_3$, dried over anhydrous Na_2SO_4, filtered, and concentrated under reduced pressure to afford the crude product. Purified by flash (0 to 7 % of MeOH in DCM) get the pure compound (60%). ^{1}H NMR (400 MHz, Chloroform-*d*) δ 5.74 (s, 1H), 4.92 (d, *J* = 8.4 Hz, 1H), 4.52 – 4.38 (m, 1H), 3.90 – 3.64 (m, 1H), 3.33 – 3.19 (m, 2H), 2.46 – 2.31 (m, 2H), 2.25 – 2.12 (m, 2H), 1.88 – 1.75 (m, 1H), 1.75 – 1.64 (m, 1H), 1.38 (s, 9H).

(S)-3-((S)-2-aminobut-3-yn-1-yl)pyrrolidin-2-one hydrogen chloride (MPI134d): MPI134d was prepared by using **MPI134c** as a white solid following a general procedure B (Crude). Used next step without further purification.

(1R,2S,5S)-3-((S)-3,3-dimethyl-2-(2,2,2-trifluoroacetamido)butanoyl)-6,6-dimethyl-N-((S)-1-((S)-2-oxopyrrolidin-3-yl)but-3-yn-2-yl)-3-azabicyclo[3.1.0]hexane-2-carboxamide (MPI134): MPI134 was prepared as a white solid following general procedure C (yield 45%). ^{1}H NMR (400 MHz, Chloroform-*d*) δ 8.33 (s, 1H), 7.04 (d, *J* = 8.5 Hz, 1H), 6.65 – 6.16 (m, 1H), 6.06 (s, 1H), 4.90 – 4.51 (m, 2H), 4.28 – 4.14 (m, 1H), 3.99 – 3.64 (m, 2H), 3.38 – 3.10 (m, 1H), 2.48 – 2.30 (m, 1H), 2.30 – 2.13 (m, 1H), 1.96 – 1.88 (m, 1H), 1.88 – 1.62 (m,

1H), 1.49 – 1.29 (m, 2H), 1.26 – 1.11 (m, 1H), 1.01 – 0.73 (m, 15H). ^{13}C NMR (100 MHz, $CDCl_3$) δ 179.95, 171.96, 170.91, 170.71, 83.21, 70.92, 61.93, 60.41, 58.67, 54.40, 48.34, 40.51, 39.30, 38.24, 37.08, 34.15, 31.77, 29.69, 28.22, 27.13, 26.69, 26.37, 26.13, 23.05, 21.05, 19.59, 19.24, 14.20, 13.19. HRMS (ESI+) m/z calculated for $C_{24}H_{34}F_3N_4O_4^+$ $(M+H)^+$ 499.2533, found 499.2529.

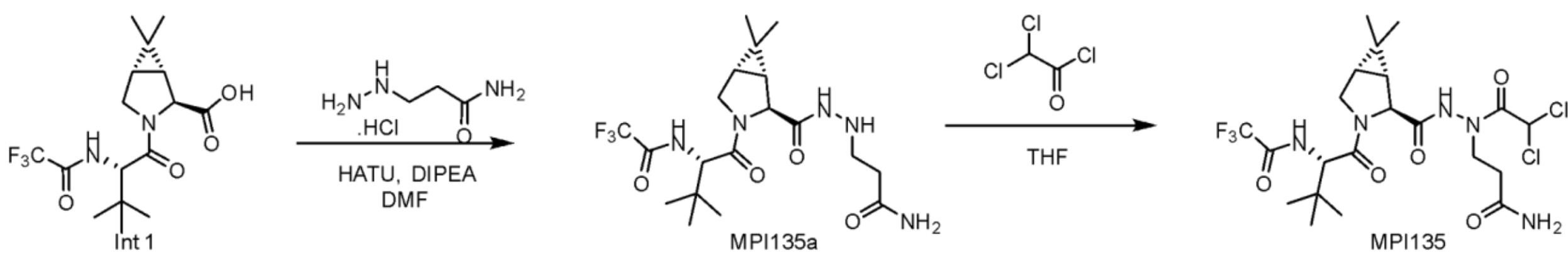

Scheme 2. The synthesis of **MPI135**

Synthesis of 3-(2-((1R,2S,5S)-3-((S)-3,3-dimethyl-2-(2,2,2-trifluoroacetamido)butanoyl)-6,6-dimethyl-3-azabicyclo[3.1.0]hexane-2-carbonyl)hydrazineyl)propenamide (MPI135a). To a solution of **Int 1** (70 mg, 0.2 mmol) in DMF (1 mL) was added compound **3-hydrazineylpropanamide** (27 mg, 0.25 mmol) and DIPEA (26 mg, 0.2 mmol). The solution was cooled at 0 °C and HATU (95 mg, 0.25 mmol) was added to the solution. The reaction mixture was then allowed to warm up to room temperature and stirred at room temperature overnight. The reaction mixture was the diluted with ethyl acetate (10 mL), washed with saturated $NaHCO_3$ solution (2×10 mL), 1 M HCl (2×10 mL) and saturated brine (10 mL) sequentially. The organic layers were then dried over anhydrous Na_2SO_4, concentrated *in vacuo* and the residue was purified with flash chromatography (1-10% methanol in dichloromethane as the eluent) to afford **MPI135a** as white solid. ^{1}H NMR (400 MHz, DMSO) δ 9.41 (dd, *J* = 27.6, 7.2 Hz, 2H), 7.31 (s, 1H), 6.76 (s, 1H), 4.97 (q, *J* = 6.1 Hz, 1H), 4.43 (t, *J* = 8.9 Hz, 1H), 4.16 (s, 1H), 3.90 (dd, *J* = 10.3, 5.4 Hz, 1H), 3.68 (d, *J* = 10.3 Hz, 1H), 2.83 (dp, *J* = 12.1, 6.5 Hz, 2H), 2.18 (t, *J* = 7.0 Hz, 2H), 1.52 (dd, *J* = 7.6, 5.2 Hz, 1H), 1.27 (s, 1H), 1.01 (d, *J* = 7.5 Hz, 12H), 0.83 (s, 3H).

Synthesis of 3-(1-(2,2-dichloroacetyl)-2-((1R,2S,5S)-3-((S)-3,3-dimethyl-2-(2,2,2-trifluoroacetamido)butanoyl)-6,6-dimethyl-3-azabicyclo[3.1.0]hexane-2-carbonyl)hydrazineyl)propenamide (MPI135). To a stirred solution of **MPI135a** (30 mg, 0.066 mmol) in THF (5 mL) at 0 °C, dichloroacetyl chloride (7 μL, 0.08) was added dropwise, and the mixture was stirred for a further 30 min at the same temperature. After completion of the reaction, the reaction mixture concentrated in a vacuum. The residue was purified by flash column chromatography (0 to 4% MeOH in ethyl acetate as the eluent)) to give the title compound **MPI135** as white solid (15 mg). ^{1}H NMR (400 MHz, $CDCl_3$) δ 9.36 (s, 1H), 7.27 (m, 5H), 5.84 (d, *J* = 79.9 Hz, 2H), 5.05 (s, 2H), 4.10 (s, 1H), 3.77 (m, 2H), 2.43 (m, 2H), 2.24 (s, 3H), 1.45 (dd, *J* = 18.9, 9.8 Hz, 2H), 1.24 (d, *J* = 19.4 Hz, 1H), 0.86 (d, *J* = 6.6 Hz, 6H). ^{13}C NMR (125 MHz, MeOD) δ 174.16, 172.07, 169.21, 158.24, 157.94, 157.64, 157.34, 119.42, 117.14, 114.86, 112.58, 63.90, 59.44, 58.20, 45.79, 37.49, 34.87, 32.39, 29.84, 28.26, 25.50, 25.46, 24.81, 19.31, 19.25, 11.48. HRMS (ESI+) m/z calculated for $C_{21}H_{30}Cl_2F_3N_5O_5^+$ $(M+H)^+$ 560.1610, found 560.1636.

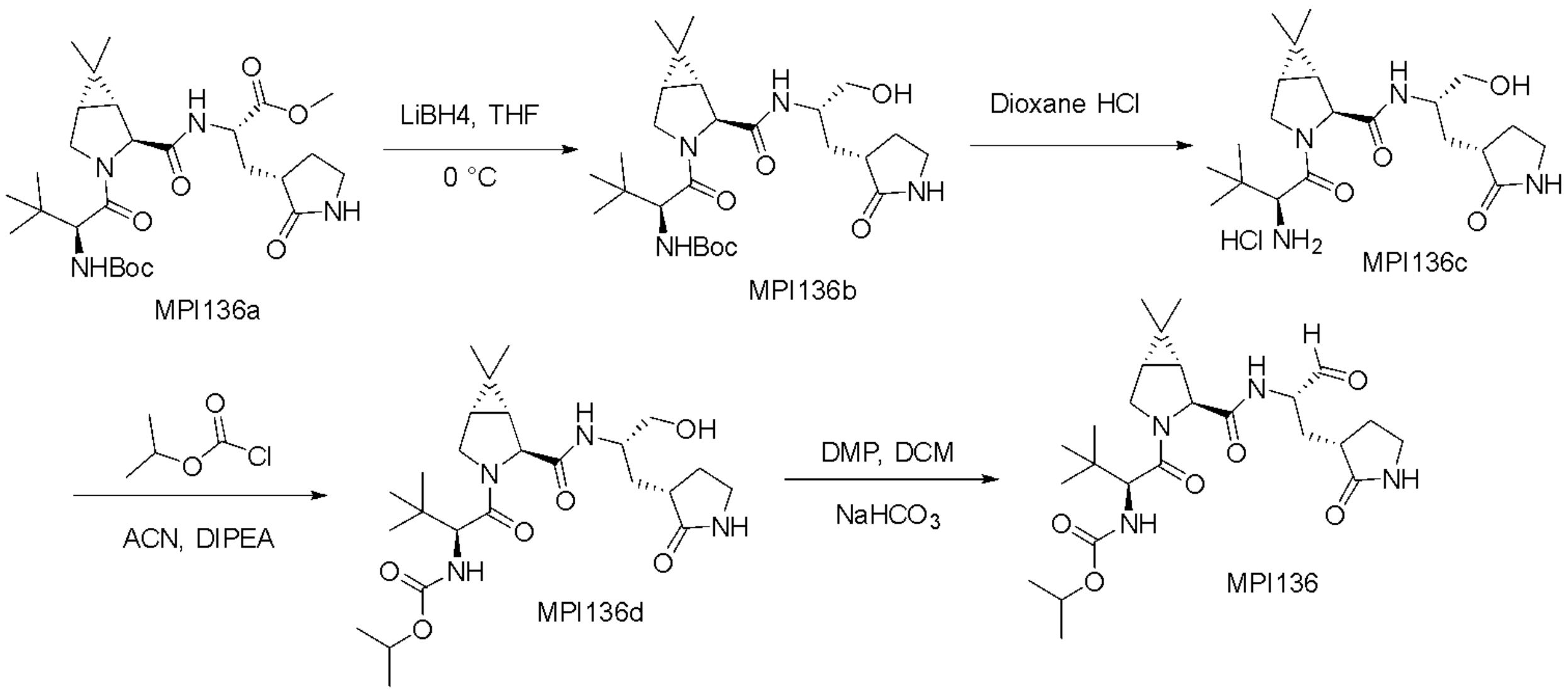

Scheme 3. The synthesis of **MPI136**

tert-butyl ((S)-1-((1R,2S,5S)-2-(((S)-1-hydroxy-3-((S)-2-oxopyrrolidin-3-yl)propan-2-yl)carbamoyl)-6,6-dimethyl-3-azabicyclo[3.1.0]hexan-3-yl)-3,3-dimethyl-1-oxobutan-2-yl)carbamate (MPI136b): MPI136b was prepared by using **MPI136a** as a white solid following a general procedure E (yield 68%). ^{1}H NMR (400 MHz, $CDCl_3$) δ 7.34 (d, J = 6.1 Hz, 1H), 5.86 (s, 1H), 5.14 (d, J = 10.2 Hz, 1H), 4.27 (s, 1H), 4.21 (d, J = 10.2 Hz, 1H), 4.08 – 3.88 (m, 3H), 3.77 – 3.51 (m, 2H), 3.42 – 3.24 (m, 2H), 2.60 – 2.47 (m, 1H), 2.41 (ddt, J = 12.7, 8.8, 4.4 Hz, 1H), 1.99 (ddd, J = 14.3, 10.2, 5.9 Hz, 1H), 1.82 (ddt, J = 12.5, 10.5, 9.0 Hz, 1H), 1.64 (ddd, J = 14.4, 6.6, 4.6 Hz, 1H), 1.49 (s, 2H), 1.39 (s, 9H), 1.03 (s, 3H), 0.99 (s, 9H), 0.87 (s, 3H). ^{13}C NMR (101 MHz, $CDCl_3$) δ 180.91, 172.08, 171.58, 155.94, 79.69, 65.57, 61.26, 58.71, 50.69, 48.33, 40.46, 37.93, 34.92, 32.12, 30.75, 28.85, 28.23, 27.74, 26.38, 26.21, 19.25, 12.55.

(1R,2S,5S)-3-((S)-2-amino-3,3-dimethylbutanoyl)-N-((S)-1-hydroxy-3-((S)-2-oxopyrrolidin-3-yl)propan-2-yl)-6,6-dimethyl-3-azabicyclo[3.1.0]hexane-2-carboxamide hydrogen chloride (MPI136c): MPI136c was prepared by using **MPI136b** as a white solid following a general procedure F (Crude). Used next step without further purification.

Isopropyl ((S)-1-((1R,2S,5S)-2-(((S)-1-hydroxy-3-((S)-2-oxopyrrolidin-3-yl)propan-2-yl)carbamoyl)-6,6-dimethyl-3-azabicyclo[3.1.0]hexan-3-yl)-3,3-dimethyl-1-oxobutan-2-yl)carbamate (MPI136d): To a stirred solution of corresponding Amino alcohol (1.0 eq,) in acetonitrile (10 mL) cooled to 0 °C was added DIPEA(1.5 eq), isopropyl chloroformate (1.3 eq.,) and allowed to stir at this temperature about 3h. Solvent was removed by rotavapor at lower temperatures. The residue was diluted with water and ethyl acetate (20 mL) and the layers were separated and extracted with ethyl acetate (10 mL x 2). The filtrate was washed with brine (2 x 25 mL), dried, filtered and concentrated in vacuum to afford **MPI136d** as a yellow syrup. ^{1}H NMR (400 MHz, $CDCl_3$) δ 7.42 –

7.33 (m, 1H), 6.08 (s, 1H), 5.32 – 5.23 (m, 1H), 4.82 (p, *J* = 6.3 Hz, 0H), 4.34 – 4.21 (m, 2H), 4.06 – 3.89 (m, 2H), 3.81 – 3.55 (m, 2H), 3.38 – 3.28 (m, 2H), 2.62 – 2.49 (m, 1H), 2.47 – 2.35 (m, 1H), 2.03 – 1.96 (m, 1H), 1.87 – 1.79 (m, 1H), 1.68 – 1.58 (m, 1H), 1.54 – 1.47 (m, 2H), 1.21 (d, *J* = 6.2 Hz, 3H), 1.18 – 1.14 (m, 2H), 1.03 (s, 3H), 0.99 (s, 9H), 0.88 (s, 3H).

Isopropyl ((S)-1-((1R,2S,5S)-6,6-dimethyl-2-(((S)-1-oxo-3-((S)-2-oxopyrrolidin-3-yl)propan-2-yl)carbamoyl)-3-azabicyclo[3.1.0]hexan-3-yl)-3,3-dimethyl-1-oxobutan-2-yl)carbamate (MPI136): **MPI136** was prepared as a white solid following general procedure A (yield 62%). ^{1}H NMR (400 MHz, $CDCl_3$) δ 9.52 (s, 1H), 7.91 (d, *J* = 6.9 Hz, 1H), 6.29 (s, 1H), 5.30 (d, *J* = 9.7 Hz, 1H), 4.81 (p, *J* = 6.2 Hz, 1H), 4.50 – 4.38 (m, 1H), 4.36 (s, 1H), 4.29 – 4.15 (m, 1H), 3.99 – 3.84 (m, 2H), 3.39 – 3.23 (m, 3H), 2.62 – 2.50 (m, 1H), 2.41 – 2.33 (m, 1H), 2.07 – 1.88 (m, 2H), 1.85 – 1.71 (m, 1H), 1.60 – 1.44 (m, 2H), 1.19 (d, *J* = 6.3 Hz, 3H), 1.15 (d, 3H), 1.03 (s, 3H), 0.97 (s, 9H), 0.88 (s, 3H). ^{13}C NMR (101 MHz, $CDCl_3$) δ 199.53, 180.20, 172.55, 172.16, 171.06, 156.30, 68.49, 60.83, 59.01, 57.33, 53.44, 48.29, 40.51, 37.57, 35.18, 30.81, 29.95, 28.47, 27.86, 26.36, 26.23, 22.01, 19.28, 12.60. HRMS (ESI+) m/z calculated for $C_{25}H_{41}N_4O_6^+$ $(M+H)^+$ 493.3021, found 493.3013.

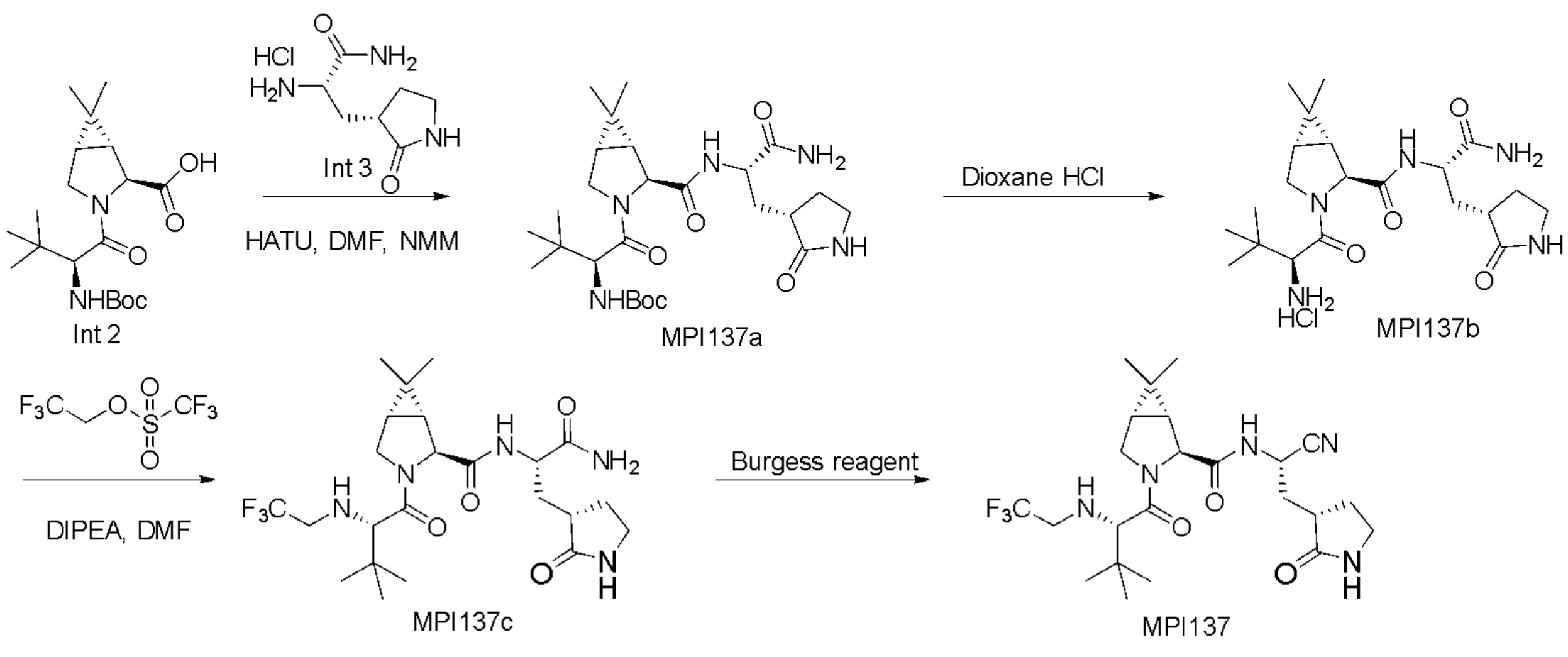

Scheme 4. The synthesis of **MPI137**

tert-butyl ((S)-1-((1R,2S,5S)-2-(((S)-1-amino-1-oxo-3-((S)-2-oxopyrrolidin-3-yl)propan-2-yl)carbamoyl)-6,6-dimethyl-3-azabicyclo[3.1.0]hexan-3-yl)-3,3-dimethyl-1-oxobutan-2-yl)carbamate (MPI137a): MPI137a was prepared by using Int 2 and Int 3 as a white solid following general procedure C (yield 65%). ^{1}H NMR (400 MHz, DMSO) δ 8.24 (d, *J* = 8.7 Hz, 1H), 7.54 (s, 1H), 7.27 (s, 1H), 7.02 (s, 1H), 6.62 (d, *J* = 9.3 Hz, 1H), 4.32 – 4.22 (m, 2H), 4.02 (d, *J* = 9.3 Hz, 1H), 3.90 – 3.77 (m, 2H), 3.17 – 2.96 (m, 2H), 2.46 – 2.34 (m, 1H), 2.13 (dt, *J* = 14.7, 8.0 Hz, 1H), 1.94 (td, *J* = 13.1, 3.7 Hz, 1H), 1.71 – 1.58 (m, 1H), 1.54 – 1.42 (m, 2H), 1.35 (s, 9H), 1.02 (s,

3H), 0.92 (s, 9H), 0.87 (s, 3H). ^{13}C NMR (101 MHz, DMSO) δ 179.09, 174.04, 171.39, 170.23, 156.41, 78.57, 60.44, 59.13, 50.85, 48.31, 37.78, 34.49, 30.99, 28.53, 27.91, 27.72, 26.87, 26.46, 19.08, 12.96.

(1R,2S,5S)-N-((S)-1-amino-1-oxo-3-((S)-2-oxopyrrolidin-3-yl)propan-2-yl)-3-((S)-2-amino-3,3-dimethylbutanoyl)-6,6-dimethyl-3-azabicyclo[3.1.0]hexane-2-carboxamide(MPI137b): MPI137b was prepared by using **MPI137a** as a white solid following a general procedure F (Crude). Used next step without further purification. ^{1}H NMR (400 MHz, DMSO) δ 8.37 (d, *J* = 9.0 Hz, 1H), 8.11 (s, 3H), 7.55 (s, 1H), 7.39 (s, 1H), 7.04 (s, 1H), 4.38 – 4.27 (m, 2H), 3.81 (dt, *J* = 10.6, 5.4 Hz, 2H), 3.65 (d, *J* = 10.7 Hz, 1H), 3.20 – 3.09 (m, 1H), 3.01 (td, *J* = 9.3, 7.1 Hz, 1H), 2.37 (q, *J* = 8.7 Hz, 1H), 2.13 (dt, *J* = 14.3, 8.0 Hz, 1H), 1.95 (td, *J* = 13.1, 3.5 Hz, 1H), 1.71 – 1.56 (m, 1H), 1.56 – 1.45 (m, 2H), 1.40 (d, *J* = 7.7 Hz, 1H), 1.03 (d, *J* = 5.2 Hz, 12H), 0.97 (s, 3H).

(1R,2S,5S)-N-((S)-1-amino-1-oxo-3-((S)-2-oxopyrrolidin-3-yl)propan-2-yl)-3-((S)-3,3-dimethyl-2-((2,2,2-trifluoroethyl)amino)butanoyl)-6,6-dimethyl-3-azabicyclo[3.1.0]hexane-2-carboxamide (3): 2,2,2-Trifluoroethyl trifluoromethanesulfonate (1.5 eq) was added dropwise to a solution of the corresponding amine (1.0 equiv) and DIPEA (3.0 eq) in anhydrous DMF under an argon atmosphere at 0 °C. The reaction mixture was then allowed to warm to room temperature and stirred for 36 h, with progress monitored by TLC. Upon completion, the reaction was quenched with water and extracted with ethyl acetate. The combined organic layers were washed with water and brine, dried over anhydrous Na_2SO_4, filtered, and concentrated under reduced pressure to afford the crude product. Directly proceeded for next step.

(1R,2S,5S)-N-((S)-1-cyano-2-((S)-2-oxopyrrolidin-3-yl)ethyl)-3-((S)-3,3-dimethyl-2-((2,2,2-trifluoroethyl)amino)butanoyl)-6,6-dimethyl-3-azabicyclo[3.1.0]hexane-2-carboxamide (MPI137): MPI137 was prepared as a white solid following general procedure D (yield 15% from 2 steps). ^{1}H NMR (400 MHz, DMSO) δ 9.05 (d, *J* = 8.6 Hz, 1H), 7.66 (s, 1H), 4.98 (ddd, *J* = 10.9, 8.6, 5.1 Hz, 1H), 4.23 (s, 1H), 3.82 (dd, *J* = 10.5, 5.4 Hz, 1H), 3.74 – 3.61 (m, 1H), 3.14 (t, *J* = 9.2 Hz, 1H), 3.03 (td, *J* = 9.3, 6.9 Hz, 1H), 2.96 – 2.83 (m, 1H), 2.46 – 2.36 (m, 1H), 2.22 – 2.02 (m, 2H), 1.80 – 1.62 (m, 2H), 1.57 (dd, *J* = 7.7, 5.2 Hz, 1H), 1.33 (t, *J* = 7.6 Hz, 1H), 1.05 (s, 3H), 0.92 (d, *J* = 1.3 Hz, 12H). HRMS (ESI+) m/z calculated for $C_{23}H_{35}F_3N_5O_3^+$ (M+H)$^+$ 486.2692, found 486.2686.

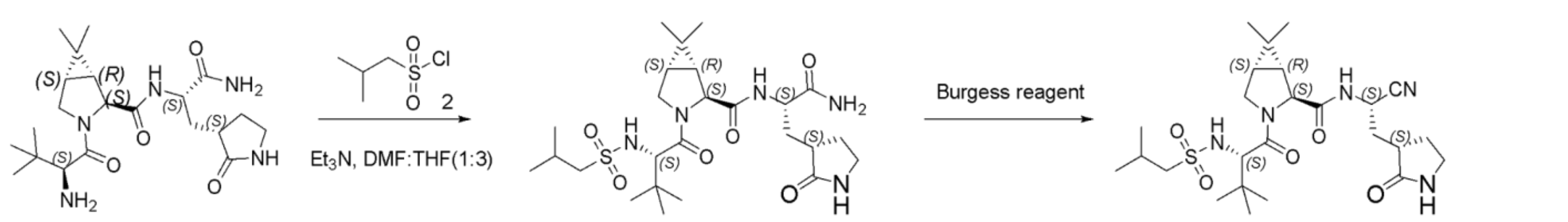

MPI137b MPI138a MPI138

Scheme 5. The synthesis of **MPI138**

(1R,2S,5S)-N-((S)-1-amino-1-oxo-3-((S)-2-oxopyrrolidin-3-yl)propan-2-yl)-3-((S)-3,3-dimethyl-2-((2-methylpropyl)sulfonamido)butanoyl)-6,6-dimethyl-3-azabicyclo[3.1.0]hexane-2-carboxamide (3): 2-methylpropane-1-sulfonyl chloride (1.1 eq) was added dropwise to a solution of the corresponding amine MPI137b (1.0 equiv) and TEA (3.0 eq) in anhydrous DMF: THF (1:3) under an argon atmosphere at 0 °C. The reaction mixture was then allowed to warm to room temperature and stirred for 36 h, with progress monitored by TLC. Upon completion, the reaction was quenched with water and extracted with ethyl acetate. The combined organic layers were washed with water and brine, dried over anhydrous Na_2SO_4, filtered, and concentrated under reduced pressure to afford the crude product. Directly proceeded for next step.

(1R,2S,5S)-N-((S)-1-cyano-2-((S)-2-oxopyrrolidin-3-yl)ethyl)-3-((S)-3,3-dimethyl-2-((2-methylpropyl)sulfonamido)butanoyl)-6,6-dimethyl-3-azabicyclo[3.1.0]hexane-2-carboxamide (MPI138): **MPI138** was prepared as a white solid following general procedure D (yield 20% from 2 steps). ^{1}H NMR (400 MHz, DMSO) δ 9.06 (d, *J* = 8.7 Hz, 1H), 7.66 (s, 1H), 7.08 (d, *J* = 9.7 Hz, 1H), 4.98 (ddd, *J* = 11.1, 8.6, 4.8 Hz, 1H), 4.17 (s, 1H), 3.87 (dd, *J* = 10.3, 5.6 Hz, 1H), 3.80 (d, *J* = 9.8 Hz, 1H), 3.65 (d, *J* = 10.4 Hz, 1H), 3.14 (t, *J* = 9.2 Hz, 1H), 3.03 (td, *J* = 9.3, 7.0 Hz, 1H), 2.92 – 2.82 (m, 1H), 2.77 – 2.67 (m, 1H), 2.42 (td, *J* = 9.6, 4.0 Hz, 1H), 2.26 – 1.96 (m, 3H), 1.79 – 1.63 (m, 2H), 1.58 (dd, *J* = 7.7, 5.5 Hz, 1H), 1.31 (d, *J* = 7.8 Hz, 1H), 1.04 (s, 3H), 1.00 – 0.83 (m, 18H). HRMS (ESI+) m/z calculated for $C_{25}H_{42}N_5O_5S^+$ $(M+H)^+$ 524.2907, found 524.2901.

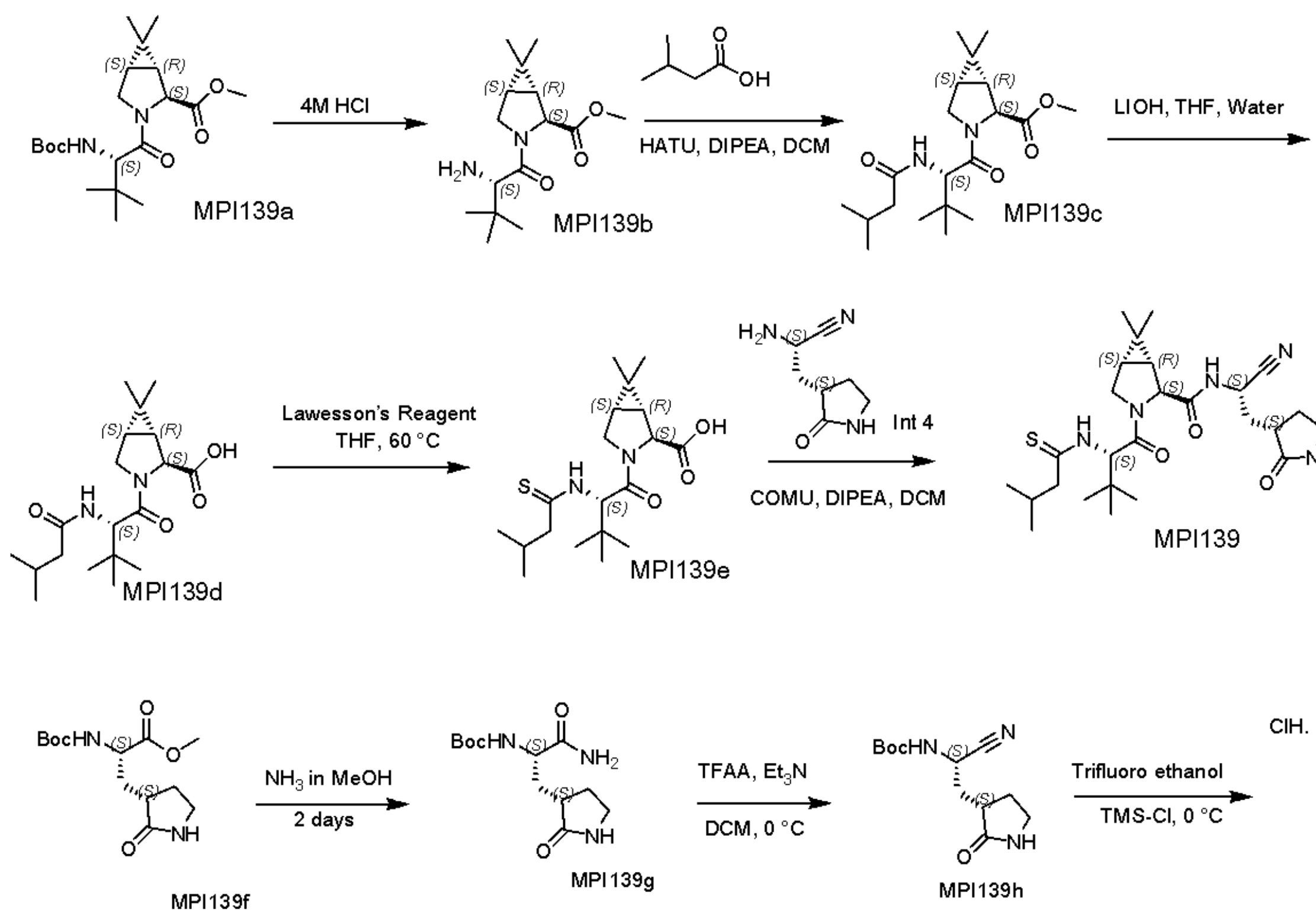

Scheme 6. The synthesis of **MPI139**

***tert*-butyl ((*S*)-1-cyano-2-((*S*)-2-oxopyrrolidin-3-yl)ethyl)carbamate(MPI139h):**
MPI139f (1.90 g, 6.59 mmol, 1.0 eq.) was stirred with NH_3 in MeOH (100 mL, 7 M NH_3 in MeOH). The mixture was stirred overnight at 80°C and then concentrated under reduced pressure to afford **MPI139g** (1.80 g, crude) as a colorless solid. Crude compound 2 (1.5 g, 5.5 mmol), ethyl acetate (25 mL), and Et_3N (3.35 g, 33.2 mmol) into a 100 mL three-neck reaction flask. Under nitrogen protection, cool to 0~20 °C, and add TFAA (2.55 g, 12.1 mmol).When the reaction is complete, add 10% sodium chloride aqueous solution (25 mL), stir and separate the liquids, extract the aqueous phase once with ethyl acetate (25 mL), combine the organic phases, wash once with 1M HCl (25 mL), and saturated brine solution (2×20 mL) sequentially. The organic layer was dried over anhydrous Na_2SO_4 and then concentrated *on vacuo*. The residue was then purified with flash chromatography (0-10% Methanol in dichloromethane as the eluent) to afford **MPI139h** as white solid (1.0 g, 72%).^{1}H NMR (400 MHz, DMSO) δ 7.81 (d, *J* = 8.3 Hz, 1H), 7.72 (s, 1H), 4.73 (q, *J* = 8.1 Hz, 1H), 3.16 (dd, *J* = 8.8, 6.5 Hz, 2H), 2.34 – 2.27 (m, 1H), 2.19 (dtd, *J* = 11.7, 5.7, 3.0 Hz, 1H), 2.07 – 2.00 (m, 1H), 1.73 (dtd, *J* = 18.1, 8.4, 4.3 Hz, 2H), 1.41 (s, 10H). ^{13}C NMR (101 MHz, DMSO) δ 177.99, 155.19, 120.39, 79.76, 37.62, 34.06, 28.49, 27.62.

(*S*)-2-amino-3-((*S*)-2-oxopyrrolidin-3-yl)propanenitrile hydrochloride (Int 4): To a stirred solution of **MPI139h** (500 mg, 1.97 mmol) in 1,4-Dioxane (1 mL) at 0 °C was added 4N HCl (2.0 mL). Reaction mixture was stirred at rt for 3 h. After completion of reaction, solvent was concentrated in a vacuum. The residue was used in the next step without further purification. (450 mg). ^{1}H NMR (400 MHz, DMSO) δ 9.30 (s, 2H), 7.87 (s, 1H),

7.50 – 7.06 (m, 1H), 4.90 – 4.68 (m, 1H), 3.19 – 3.06 (m, 2H), 2.47 (s, 1H), 2.34 – 2.11 (m, 2H), 2.01 – 1.83 (m, 1H), 1.75 – 1.63 (m, 1H). ^{13}C NMR (101 MHz, DMSO) δ 177.81, 117.39, 59.80, 59.47, 38.15, 32.67, 28.01.

Methyl (1*R*,2*S*,5*S*)-3-((*S*)-3,3-dimethyl-2-(3-methylbutanamido)butanoyl)-6,6-dimethyl-3-azabicyclo[3.1.0]hexane-2-carboxylate (139c): To a solution of **MPI139b** (1.57 mmol, 0.5 g) and **3-methylbutanoic acid** (2.35 mmol, 0.240 g) in anhydrous DCM (20 mL) was added DIPEA (4.71 mmol, 0.60 g) and was cooled to 0 °C. HATU (2.35 mmol, 0.896 g) was added to the solution under 0 °C and then stirred at room temperature overnight. The reaction mixture was then diluted with ethyl acetate (50 mL) and washed with saturated $NaHCO_3$ solution (2×20 mL), 1 M HCl solution (2×20 mL), and saturated brine solution (2×20 mL) sequentially. The organic layer was dried over anhydrous Na_2SO_4 and then concentrated *on vacuo*. The residue was then purified with flash chromatography (50-100% EtOAc in hexanes as the eluent) to afford compound **MPI139c** as a colorless solid (460 mg, 80%).^{1}H NMR (400 MHz, $CDCl_3$) δ 5.94 (d, *J* = 9.8 Hz, 1H), 4.54 (d, *J* = 9.7 Hz, 1H), 4.38 (s, 1H), 3.93 – 3.79 (m, 2H), 3.68 (s, 3H), 2.02 – 1.98 (m, 2H), 1.41 – 1.36 (m, 2H), 0.96 (d, *J* = 1.5 Hz, 12H), 0.91 – 0.82 (m, 7H), 0.81 (s, 3H). ^{13}C NMR (101 MHz, $CDCl_3$) δ 172.17, 171.91, 170.49, 60.39, 59.26, 56.77, 52.26, 47.77, 45.97, 35.29, 30.30, 27.39, 26.38, 26.23, 26.20, 22.38, 22.33, 21.05, 19.41, 14.20, 12.41.

(1*R*,2*S*,5*S*)-*N*-((*S*)-1-cyano-2-((*S*)-2-oxopyrrolidin-3-yl)ethyl)-3-((*S*)-3,3-dimethyl-2-(3-methylbutanethioamido)butanoyl)-6,6-dimethyl-3-azabicyclo[3.1.0]hexane-2-carboxamide (MPI139): The peptide **MPI139c** (450 mg, 1.22 mmol) was dissolved in THF/H_2O (1:1, 20 mL). LiOH (136 mg, 1.84 mmol) was added at 0 °C. The mixture was stirred at room temperature overnight. Then THF was removed *on vacuum* and the aqueous layer was acidified with 1 M HCl and extracted with dichloromethane (3 x 20 mL). The organic layer was dried over anhydrous Na_2SO_4 and concentrated to yield **MPI139d** as white solid (410 mg). A mixture of the **MPI139d** (400 mg, 1.13 mmol) and Lawesson's reagent (235 mg, 0.56 mmol) in dry THF was stirred for 2 h at room temperature and then refluxed for 0.5 h under argon atmosphere, Then the reaction mixture was filtered and concentrated under reduced pressure to provide the title compound, the resulting residue was purified by flash chromatography (hexane/EtOAc) to afford compound **MPI139e** (188 mg, yield 45%, white solid). **MPI139e** (100 mg, 0.27 mmol) and **Int 4** (77 mg, 0.40 mmol) were dissolved in DCM (6.0 ml) and COMU (174 mg, 0.40 mmol) was added at 0 °C. The reaction mixture was stirred at room temperature for 5 min and Hunig's base (0.14 mL 0.81 mmol) was added. The solution was stirred for 2 hours. The mixture was poured onto an aqueous sodium hydrogen carbonate (saturated, 20 ml) and water (20 ml). The mixture was stirred in an Erlenmeyer flask for 10 min. The aqueous layer was extracted with DCM. The organic layer was washed with brine and dried on $MgSO_4$. It was concentrated under reduced pressure. The crude product was purified by flash (eluted with 0:100 to 5:95 Methanol/ DCM) to give the title compound **MPI139** as a colorless solid (30 mg, 22%). ^{1}H NMR (400 MHz, DMSO) δ 9.64 (d, *J* = 8.9 Hz, 1H), 8.96 (d, *J* = 8.7 Hz, 1H), 7.59 (s, 1H), 5.19 (d, *J* = 8.9 Hz, 1H), 4.91 (ddd, *J* = 11.1, 8.6, 4.8 Hz, 1H), 4.16 – 4.03 (m, 2H), 3.89 – 3.63 (m, 2H), 3.08 (t, *J* = 9.1 Hz, 1H), 2.48 (d, *J* = 7.3 Hz, 2H),

2.34 (dq, J = 14.3, 5.3 Hz, 1H), 2.16 – 1.97 (m, 3H), 1.64 (ddd, J = 12.6, 10.4, 4.5 Hz, 2H), 1.49 (dd, J = 7.7, 5.3 Hz, 1H), 1.23 (d, J = 7.7 Hz, 1H), 0.94 (d, J = 9.4 Hz, 11H), 0.81 – 0.71 (m, 9H). ^{13}C NMR (101 MHz, $CDCl_3$) δ 171.26, 169.87, 164.77, 77.24, 66.69, 47.32, 40.53, 38.44, 36.04, 36.03, 29.38, 28.14, 27.32, 26.73, 26.22, 22.01, 21.84, 19.67, 13.21. **HRMS- ESI** (m/z) [M+H]+ calc'd for $C_{26}H_{42}N_5O_3S^+$, 504.3008, found 504.2994.

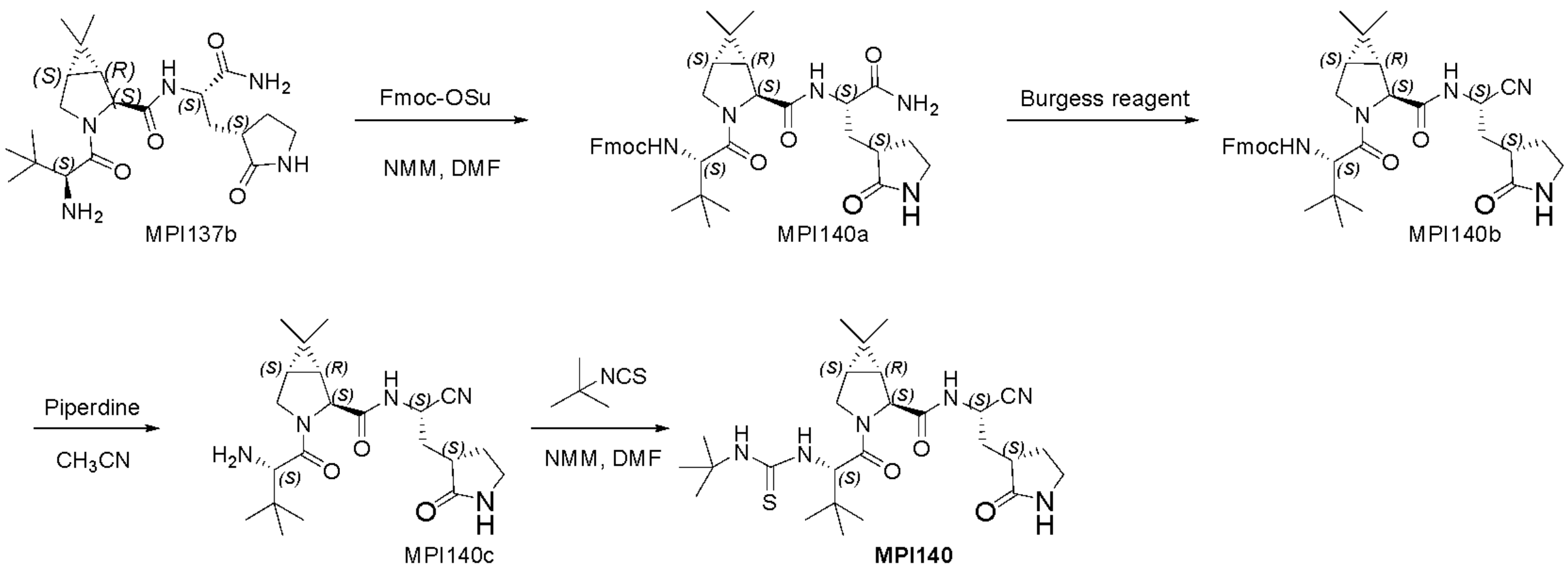

Scheme 7. The synthesis of **MPI140**

(9H-fluoren-9-yl)methyl ((S)-1-((1R,2S,5S)-2-(((S)-1-amino-1-oxo-3-((S)-2-oxopyrrolidin-3-yl)propan-2-yl)carbamoyl)-6,6-dimethyl-3-azabicyclo[3.1.0]hexan-3-yl)-3,3-dimethyl-1-oxobutan-2-yl)carbamate (MPI140a): To a stirred solution of **MPI137b** (1.0 eq) in dry DMF (10 mL), N-methyl morpholine (1.5 eq) was added dropwise. Next Fmoc-Osu was added under argon atmosphere at 0 °C. The reaction mixture was then allowed to warm to room temperature and stirred for 6 h, with progress monitored by TLC. Upon completion, the reaction was quenched with water and extracted with ethyl acetate. The combined organic layers were washed with water and brine, dried over anhydrous Na_2SO_4, filtered, and concentrated under reduced pressure and purified by flash chromatography (0 to 7% MeOH in DCM) to afford the desired product. ^{1}H NMR (400 MHz, $CDCl_3$) δ 8.30 (s, 1H), 7.76 (t, J = 7.9 Hz, 2H), 7.56 (dd, J = 29.6, 8.0 Hz, 2H), 7.45 – 7.15 (m, 5H), 6.27 (s, 1H), 6.00 – 5.70 (m, 2H), 4.42 (d, J = 26.7 Hz, 2H), 4.32 – 3.92 (m, 4H), 3.42 – 3.23 (m, 2H), 2.46 (d, J = 39.0 Hz, 2H), 2.18 – 1.84 (m, 3H), 1.63 – 1.43 (m, 2H), 1.15 – 0.43 (m, 15H). ^{13}C NMR (101 MHz, $CDCl_3$) δ 180.62, 174.14, 172.03, 171.58, 143.69, 141.25, 127.71, 127.08, 125.09, 119.97, 67.14, 59.12, 53.44, 47.06, 40.77, 35.23, 32.47, 30.72, 27.78, 26.48, 25.96, 19.40, 12.63.

(9H-fluoren-9-yl)methyl ((S)-1-((1R,2S,5S)-2-(((S)-1-cyano-2-((S)-2-oxopyrrolidin-3-yl)ethyl)carbamoyl)-6,6-dimethyl-3-azabicyclo[3.1.0]hexan-3-yl)-3,3-dimethyl-1-oxobutan-2-yl)carbamate (MPI140b): **MPI140b** was prepared as a white solid following general procedure D (yield 50%). ^{1}H NMR (400 MHz, DMSO) δ 9.05 – 8.78 (m, 1H), 7.89 (d, J = 7.6 Hz, 2H), 7.81 – 7.65 (m, 2H), 7.63 – 7.50 (m, 1H), 7.37 (dt, J = 36.2, 7.2 Hz, 4H), 4.93 (d, J = 38.7 Hz, 1H), 4.29 – 4.08 (m, 4H), 3.86 (d, J = 6.6 Hz, 1H), 3.77 (d, J = 10.2 Hz, 1H), 3.10

(dd, *J* = 31.8, 9.1 Hz, 2H), 2.44 (dd, *J* = 9.0, 4.1 Hz, 1H), 2.13 (t, *J* = 15.6 Hz, 2H), 1.79 – 1.60 (m, 2H), 1.53 (d, *J* = 8.6 Hz, 1H), 1.28 (d, *J* = 7.5 Hz, 1H), 1.08 – 0.42 (m, 15H).

(1R,2S,5S)-3-((S)-2-amino-3,3-dimethylbutanoyl)-N-((S)-1-cyano-2-((S)-2-oxopyrrolidin-3-yl)ethyl)-6,6-dimethyl-3-azabicyclo[3.1.0]hexane-2-carboxamide (MPI140c): To a stirred solution of **MPI140b** in acetonitrile, 20 % piperidine in acetonitrile was added then stirred reaction until finish the starting material (by TLC). Remove the solvent through rotorvap and wash with ether. Crude material directly used for the next step without further purification.

(1R,2S,5S)-3-((S)-2-(3-(tert-butyl)thioureido)-3,3-dimethylbutanoyl)-N-((S)-1-cyano-2-((S)-2-oxopyrrolidin-3-yl)ethyl)-6,6-dimethyl-3-azabicyclo[3.1.0]hexane-2-carboxamide (MPI140): 2-isothiocyanato-2-methylpropane (1.2 eq)) was added dropwise to a solution of the corresponding amine (1.0 equiv) and NMM (3.0 eq) in anhydrous DMF under an argon atmosphere at 0 °C. The reaction mixture was then allowed to warm to room temperature and stirred for 12 h, with progress monitored by TLC. Upon completion, the reaction was quenched with water and extracted with ethyl acetate. The combined organic layers were washed with water and brine, dried over anhydrous Na_2SO_4, filtered, and concentrated under reduced pressure to afford the crude product. Crude products were purified by flash chromatography (0 to 7% MeOH in DCM) to afford the desired product. (30% yield). ^{1}H NMR (400 MHz, DMSO) δ 8.99 (d, *J* = 8.6 Hz, 1H), 7.65 (s, 1H), 7.54 (s, 1H), 7.30 (d, *J* = 9.2 Hz, 1H), 5.04 – 4.91 (m, 2H), 4.23 (d, *J* = 10.3 Hz, 1H), 4.13 (s, 1H), 3.87 (dd, *J* = 10.2, 5.6 Hz, 1H), 3.15 (t, *J* = 9.1 Hz, 1H), 3.03 (td, *J* = 9.3, 7.0 Hz, 1H), 2.45 (dd, *J* = 8.9, 4.0 Hz, 1H), 2.22 – 2.03 (m, 2H), 1.80 – 1.65 (m, 2H), 1.55 (dd, *J* = 7.7, 5.4 Hz, 1H), 1.39 (s, 9H), 1.28 (d, *J* = 7.7 Hz, 1H), 1.02 (s, 3H), 0.93 (s, 9H), 0.90 (s, 3H). **HRMS- ESI** (m/z) [M+H]+ calc'd for $C_{26}H_{42}N_6O_3S^+$, 519.3117, found 519.3105.

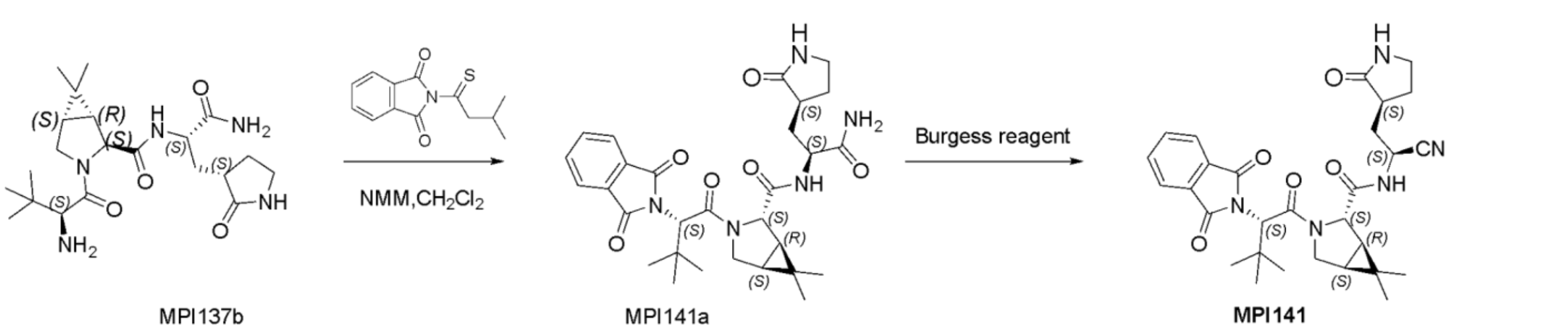

Scheme 8. The synthesis of **MPI141**

(1R,2S,5S)-N-((S)-1-amino-1-oxo-3-((S)-2-oxopyrrolidin-3-yl)propan-2-yl)-3-((S)-2-(1,3-dioxoisoindolin-2-yl)-3,3-dimethylbutanoyl)-6,6-dimethyl-3-azabicyclo[3.1.0]hexane-2-carboxamide (3): 2-(3-methylbutanethioyl)isoindoline-1,3-dione (1.2 eq)) was added dropwise to a solution of the corresponding amine

MPI137b (1.0 eq) and NMM (3.0 eq) in anhydrous DCM under an argon atmosphere at 0 °C. The reaction mixture was then allowed to warm to room temperature and stirred for 36 h, with progress monitored by TLC. Upon completion, the reaction was quenched with water and extracted with DCM. The combined organic layers were washed with water and brine, dried over anhydrous Na_2SO_4, filtered, and concentrated under reduced pressure to afford the crude product. Crude products were purified by flash chromatography (0 to 7% MeOH in DCM) to afford the desired product. (30% yield).

(1R,2S,5S)-N-((S)-1-cyano-2-((S)-2-oxopyrrolidin-3-yl)ethyl)-3-((S)-2-(1,3-dioxoisoindolin-2-yl)-3,3-dimethylbutanoyl)-6,6-dimethyl-3-azabicyclo[3.1.0]hexane-2-carboxamide (MPI141) (Isomers):
MPI141 was prepared as a white solid following general procedure D (yield 50%). ^{1}H NMR (400 MHz, DMSO) δ 8.97 (d, J = 8.4 Hz, 1H), 8.42 (dd, J = 19.9, 8.3 Hz, 1H), 7.97 – 7.83 (m, 2H), 7.68 – 7.49 (m, 2H), 7.41 – 7.33 (m, 1H), 6.32 (dd, J = 37.1, 10.2 Hz, 1H), 5.02 – 4.91 (m, 1H), 4.27 (dd, J = 21.4, 8.3 Hz, 1H), 4.12 (d, J = 2.7 Hz, 1H), 3.75 (tt, J = 12.4, 6.3 Hz, 1H), 3.68 – 3.53 (m, 1H), 3.14 (t, J = 9.0 Hz, 1H), 3.03 (q, J = 8.8 Hz, 1H), 2.95 – 2.83 (m, 1H), 2.45 – 2.35 (m, 1H), 2.20 – 2.03 (m, 2H), 1.79 – 1.65 (m, 2H), 1.55 (q, J = 7.0 Hz, 1H), 1.27 (d, J = 7.5 Hz, 1H), 1.14 – 0.87 (m, 14H), 0.79 (d, J = 10.8 Hz, 2H). **HRMS- ESI** (m/z) [M+H]+ calc'd for $C_{29}H_{36}N_5O_5^+$, 534.2716, found 534.2703.

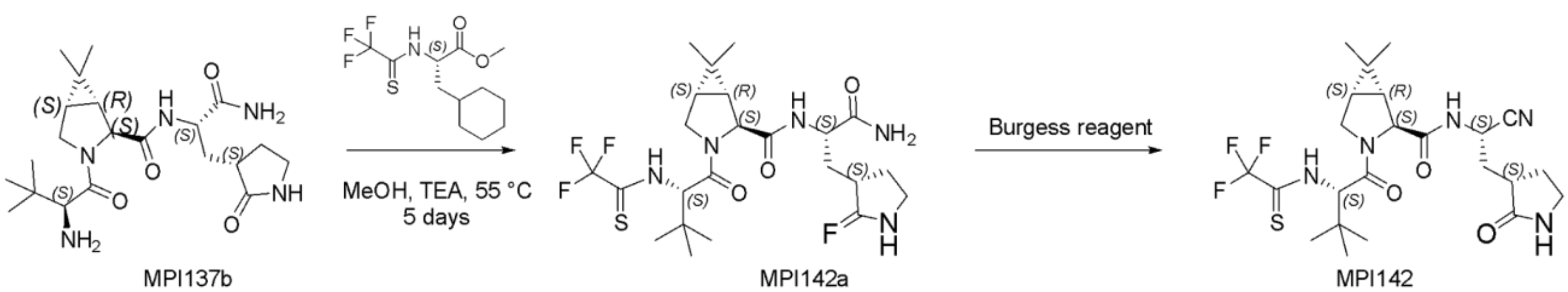

Scheme 9. The synthesis of **MPI142**

(1R,2S,5S)-N-((S)-1-amino-1-oxo-3-((S)-2-oxopyrrolidin-3-yl)propan-2-yl)-3-((S)-3,3-dimethyl-2-(2,2,2-trifluoroethanethioamido)butanoyl)-6,6-dimethyl-3-azabicyclo[3.1.0]hexane-2-carboxamide (MPI142a): A solution of **MPI137b** (1 eq) in TEA (3 eq) was added methyl (S)-3-cyclohexyl-2-(2,2,2-trifluoroethanethioamido)propanoate (1.5 eq) and stirred at 55 °C for 5 days. Upon completion, the reaction mixture was concentrated in vacuum to remove MeOH and afford a residue. The combined residue was diluted with DCM (20 mL) and washed with 1 M aqueous HCl (20mL). The organic layer was separated and the aqueous layer was extracted with DCM (20 ml * 2). The combined organic layer was dried over Na_2SO_4, filtered and concentrated under reduced pressure to give a residue. The residue products were purified by flash chromatography (0 to 10% MeOH in DCM) to afford the desired product. (20% yield). ^{1}H NMR (400 MHz, DMSO) δ 11.01 (s, 1H), 8.30 (d, J = 8.9 Hz, 1H), 7.55 (s, 1H), 7.34 (s, 1H), 7.04 (s, 1H), 5.17 (s, 1H), 4.37 – 4.28 (m, 2H), 3.94 (dd, J = 13.7, 8.5 Hz, 2H), 3.15 (t, J = 9.1 Hz, 1H), 3.03 (q, J = 8.6 Hz, 1H), 2.42 – 2.33 (m, 1H),

2.18 – 2.12 (m, 1H), 1.97 – 1.93 (m, 1H), 1.68 (tt, J = 21.5, 10.7 Hz, 1H), 1.59 – 1.45 (m, 2H), 1.39 (d, J = 7.6 Hz, 1H), 1.03 (d, J = 10.8 Hz, 12H), 0.85 (s, 3H).

(1R,2S,5S)-N-((S)-1-cyano-2-((S)-2-oxopyrrolidin-3-yl)ethyl)-3-((S)-3,3-dimethyl-2-(2,2,2-trifluoroethanethioamido)butanoyl)-6,6-dimethyl-3-azabicyclo[3.1.0]hexane-2-carboxamide (MPI142): MPI142 was prepared as a white solid following general procedure D (yield 60%). ^{1}H NMR (400 MHz, DMSO) δ 11.01 (d, J = 8.4 Hz, 1H), 9.07 (d, J = 8.6 Hz, 1H), 7.68 (s, 1H), 5.14 (d, J = 8.4 Hz, 1H), 4.99 (ddd, J = 10.9, 8.6, 5.0 Hz, 1H), 4.19 (s, 1H), 3.97 (d, J = 5.7 Hz, 2H), 3.19 – 2.99 (m, 3H), 2.44 – 2.33 (m, 1H), 2.21 – 2.04 (m, 2H), 1.73 (td, J = 12.3, 5.8 Hz, 2H), 1.64 – 1.55 (m, 1H), 1.34 (d, J = 7.7 Hz, 1H), 1.04 (d, J = 4.6 Hz, 12H), 0.85 (s, 3H). **HRMS- ESI** (m/z) [M+H]+ calc'd for $C_{23}H_{33}F_3N_5O_3S^+$, 516.2256, found 516.2241.

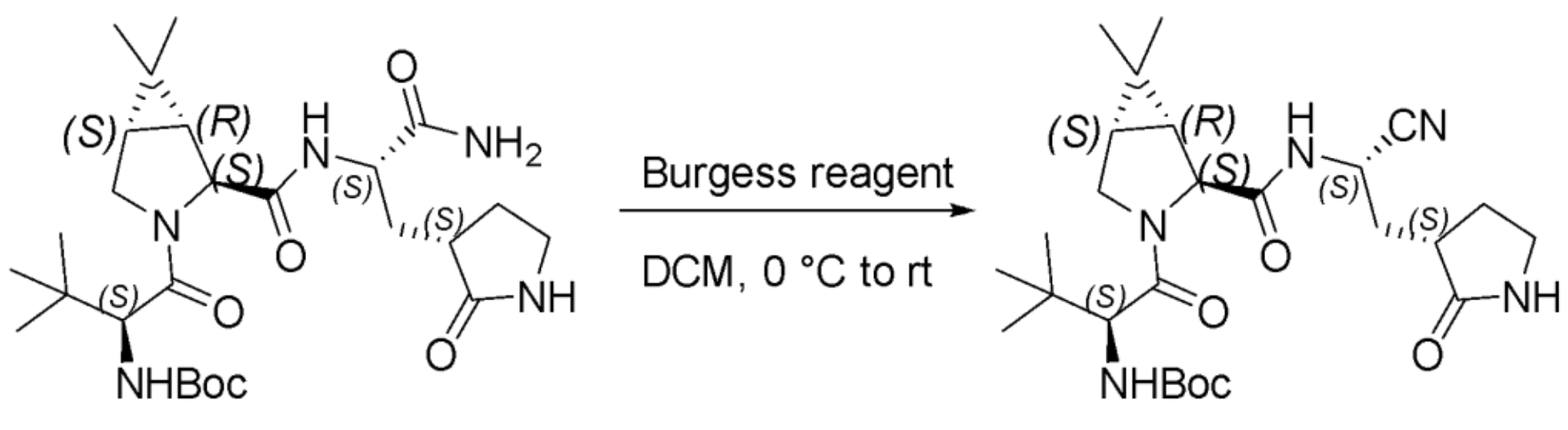

MPI137a YR-B-76/MPI143

Scheme 10. The synthesis of **MPI143**

tert-butyl ((S)-1-((1R,2S,5S)-2-(((S)-1-cyano-2-((S)-2-oxopyrrolidin-3-yl)ethyl)carbamoyl)-6,6-dimethyl-3-azabicyclo[3.1.0]hexan-3-yl)-3,3-dimethyl-1-oxobutan-2-yl)carbamate (MPI143): MPI143 was prepared as a white solid following general procedure D (yield 60%). ^{1}H NMR (400 MHz, DMSO) δ 8.99 (d, J = 8.5 Hz, 1H), 7.66 (s, 1H), 6.68 (d, J = 9.1 Hz, 1H), 4.96 (ddd, J = 10.9, 8.5, 5.1 Hz, 1H), 4.16 (s, 1H), 4.09 – 3.98 (m, 1H), 3.93 – 3.79 (m, 2H), 3.19 – 3.09 (m, 1H), 3.04 (td, J = 9.4, 7.0 Hz, 1H), 2.41 (qt, J = 8.5, 4.4 Hz, 1H), 2.22 – 2.03 (m, 2H), 1.82 – 1.62 (m, 2H), 1.54 (dd, J = 7.6, 5.0 Hz, 1H), 1.35 (s, 9H), 1.28 (d, J = 7.6 Hz, 1H), 1.18 (t, J = 7.1 Hz, 1H), 1.03 (s, 3H), 0.90 (d, J = 13.9 Hz, 12H).

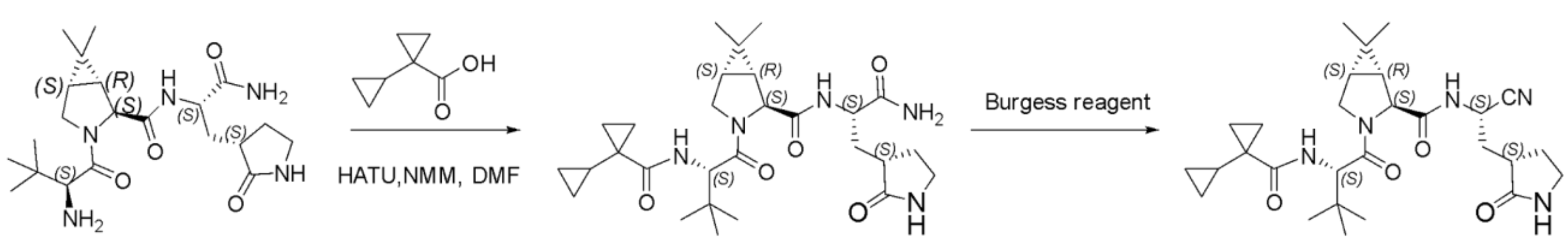

MPI137b MPI144a MPI144

Scheme 11. The synthesis of **MPI144**

(1R,2S,5S)-3-((S)-2-([1,1'-bi(cyclopropane)]-1-carboxamido)-3,3-dimethylbutanoyl)-N-((S)-1-amino-1-oxo-3-((S)-2-oxopyrrolidin-3-yl)propan-2-yl)-6,6-dimethyl-3-azabicyclo[3.1.0]hexane-2-carboxamide

(MPI144a): MPI144a was prepared by using MPI137b and [1,1'-bi(cyclopropane)]-1-carboxylic acid followed by general procedure C. (Yield 60%),^{1}H NMR (400 MHz, DMSO) δ 8.16 (d, *J* = 8.7 Hz, 1H), 7.48 (s, 1H), 7.22 (s, 1H), 7.00 – 6.92 (m, 2H), 4.31 (d, *J* = 9.3 Hz, 1H), 4.25 – 4.13 (m, 1H), 3.76 (dd, *J* = 10.3, 5.2 Hz, 1H), 3.67 (d, *J* = 10.3 Hz, 1H), 3.21 – 3.01 (m, 1H), 2.95 (q, *J* = 9.1 Hz, 1H), 2.36 – 2.24 (m, 1H), 2.06 (dt, *J* = 14.0, 7.4 Hz, 1H), 1.93 – 1.79 (m, 1H), 1.68 – 1.37 (m, 3H), 1.28 (d, *J* = 7.7 Hz, 1H), 1.25 – 1.19 (m, 1H), 0.96 – 0.82 (m, 12H), 0.78 – 0.68 (m, 4H), 0.66 – 0.56 (m, 1H), 0.47 – 0.30 (m, 4H), 0.01 (dq, *J* = 9.0, 4.6 Hz, 2H).

(1R,2S,5S)-3-((S)-2-([1,1'-bi(cyclopropane)]-1-carboxamido)-3,3-dimethylbutanoyl)-N-((S)-1-cyano-2-((S)-2-oxopyrrolidin-3-yl)ethyl)-6,6-dimethyl-3-azabicyclo[3.1.0]hexane-2-carboxamide (MPI144): MPI144 was prepared as a white solid following general procedure D (yield 60%). ^{1}H NMR (400 MHz, $CDCl_3$) δ 8.02 (dd, *J* = 13.5, 7.4 Hz, 1H), 7.09 (dd, *J* = 22.5, 9.2 Hz, 1H), 6.25 (s, 1H), 5.02 – 4.92 (m, 1H), 4.70 – 4.47 (m, 1H), 4.31 (s, 1H), 4.00 (d, *J* = 10.3 Hz, 1H), 3.89 (dd, *J* = 10.3, 4.9 Hz, 1H), 3.42 – 3.26 (m, 2H), 2.56 (ddd, *J* = 16.6, 10.2, 7.2 Hz, 1H), 2.47 – 2.25 (m, 2H), 1.94 (dt, *J* = 14.0, 6.9 Hz, 1H), 1.87 – 1.75 (m, 1H), 1.62 – 1.45 (m, 2H), 1.38 – 1.21 (m, 1H), 1.09 – 0.96 (m, 12H), 0.95 – 0.77 (m, 5H), 0.66 – 0.39 (m, 4H), 0.17 (dd, *J* = 9.0, 5.6 Hz, 2H). ^{13}C NMR (101 MHz, $CDCl_3$) δ 181.33, 178.97, 174.69, 171.19, 170.59, 118.37, 60.41, 57.38, 48.34, 40.44, 39.18, 37.66, 36.59, 36.04, 35.26, 34.19, 29.93, 28.29, 27.85, 26.52, 26.49, 26.22, 25.03, 24.91, 12.71, 12.58, 12.25, 11.34, 3.53, 3.31. **HRMS- ESI** (m/z) [M+H]+ calc'd for $C_{28}H_{42}N_5O_4^+$, 512.3231 , found 512.3229.

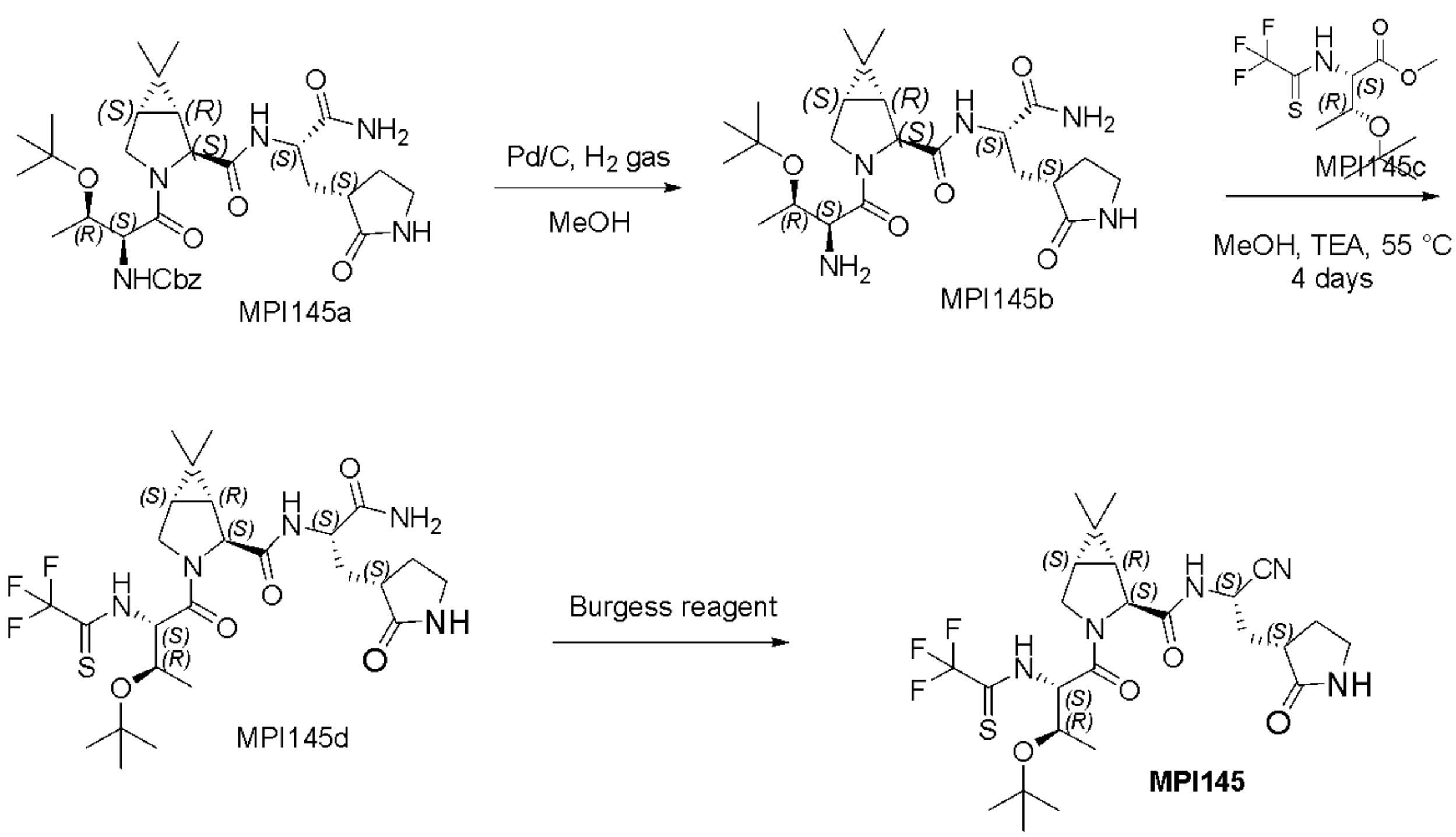

Scheme 12. The synthesis of **MPI145**

(1R,2S,5S)-N-((S)-1-amino-1-oxo-3-((S)-2-oxopyrrolidin-3-yl)propan-2-yl)-3-(O-(tert-butyl)-N-(2,2,2-trifluoroethanethioyl)-L-threonyl)-6,6-dimethyl-3-azabicyclo[3.1.0]hexane-2-carboxamide (MPI145d): Stir MPI145a at room temperature overnight under a hydrogen atmosphere in the presence of Pd/C (10 wt%). Filter the reaction mixture and concentrate under reduced pressure and used the compound next step without further purification. Next same as the MPI142a procedure, get the gummy solid. (Yield 45%). ^{1}H NMR (400 MHz, DMSO) δ 11.39 (d, *J* = 7.9 Hz, 1H), 8.31 (d, *J* = 8.8 Hz, 1H), 7.58 (s, 1H), 7.33 (s, 1H), 7.04 (s, 1H), 5.00 (t, *J* = 7.7 Hz, 1H), 4.36 – 4.27 (m, 2H), 4.18 – 4.02 (m, 1H), 3.98 (d, *J* = 3.2 Hz, 2H), 3.17 (t, *J* = 9.0 Hz, 1H), 3.05 (td, *J* = 9.3, 6.9 Hz, 1H), 2.42 – 2.31 (m, 1H), 2.14 (dq, *J* = 15.3, 7.7 Hz, 1H), 2.02 – 1.88 (m, 1H), 1.76 – 1.57 (m, 1H), 1.56 – 1.45 (m, 2H), 1.40 (d, *J* = 7.7 Hz, 1H), 1.19 (d, *J* = 6.2 Hz, 3H), 1.12 (s, 9H), 1.02 (s, 3H), 0.87 (s, 3H).

(1R,2S,5S)-3-(O-(tert-butyl)-N-(2,2,2-trifluoroethanethioyl)-L-threonyl)-N-((S)-1-cyano-2-((S)-2-oxopyrrolidin-3-yl)ethyl)-6,6-dimethyl-3-azabicyclo[3.1.0]hexane-2-carboxamide (MPI145): MPI145 was prepared as a white solid following general procedure D (yield 55%). ^{1}H NMR (400 MHz, DMSO) δ 11.35 (d, *J* = 7.7 Hz, 1H), 9.06 – 8.98 (m, 2H), 7.70 (s, 1H), 4.99 (dt, *J* = 10.8, 6.6 Hz, 2H), 4.15 (s, 1H), 4.12 – 4.06 (m, 1H), 4.05 – 3.94 (m, 2H), 3.17 (t, *J* = 8.9 Hz, 1H), 3.09 – 3.03 (m, 2H), 2.44 – 2.34 (m, 1H), 2.12 (ddd, *J* = 14.0, 10.7, 4.9 Hz, 2H), 1.80 – 1.65 (m, 2H), 1.61 (dd, *J* = 7.6, 5.0 Hz, 1H), 1.35 (d, *J* = 7.7 Hz, 1H), 1.17 (d, *J* = 2.8 Hz, 3H), 1.12 (s, 9H), 1.03 (s, 3H), 0.88 (s, 3H). **HRMS- ESI** (m/z) [M-H$^-$] calc'd for C25H35F3N5O4S$^-$, 558.2362, found 558.2367.

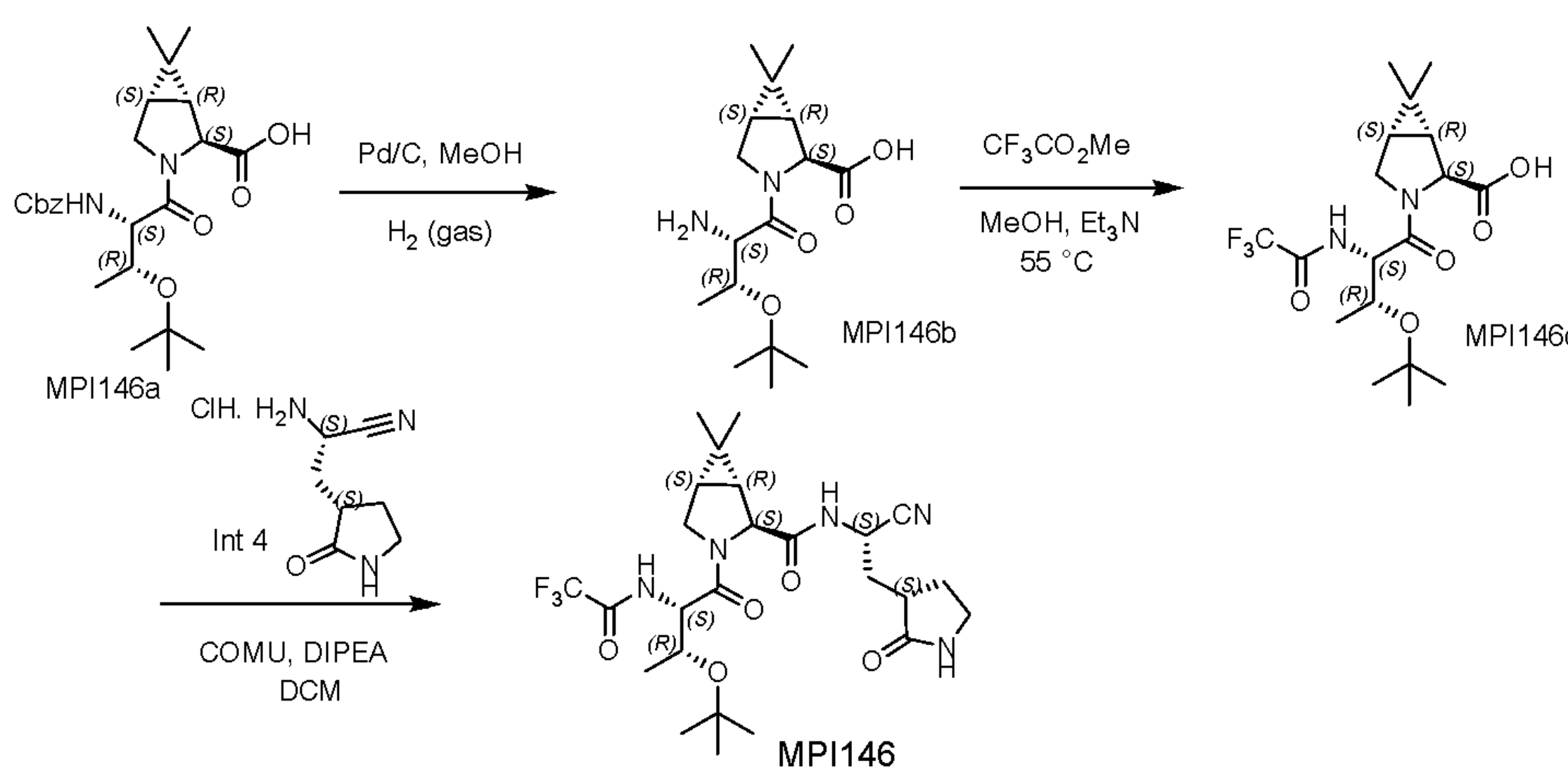

Scheme 13. The synthesis of **MPI146**

(1*R*,2*S*,5*S*)-3-(*O*-(*tert*-butyl)-*L*-threonyl)-6,6-dimethyl-3-azabicyclo[3.1.0]hexane-2-carboxylic acid (MPI146b): The compound **MPI146a** (0.9 g, 2.01 mmol) in methanol (30 mL) was added palladium on carbon (0.2 g) and the mixture was allowed to react in the presence of hydrogen (P = 50 psi) at 25°C for 12 hours. Then

the reaction mixture was filtered and concentrated under reduced pressure to provide the title compound which was used in the next step without further purification.^{1}H NMR (400 MHz, DMSO) δ 4.36 (s, 1H), 3.85 (ddd, *J* = 14.5, 7.8, 5.6 Hz, 1H), 3.49 – 3.36 (m, 3H), 1.70 (d, *J* = 7.6 Hz, 1H), 1.25 (dd, *J* = 7.9, 4.6 Hz, 1H), 1.16 (s, 10H), 1.07 – 0.86 (m, 10H). ^{13}C NMR (101 MHz, DMSO) δ 173.37, 167.44, 74.76, 67.61, 63.21, 56.70, 32.69, 28.99, 26.75, 24.75, 19.54, 18.72, 13.34.

(1*R*,2*S*,5*S*)-3-(*O*-(*tert*-butyl)-*N*-(2,2,2-trifluoroacetyl)-*L*-threonyl)-6,6-dimethyl-3-azabicyclo[3.1.0]hexane-2-carboxylic acid (MPI146c):A solution of **MPI146b** (0.5 g, 2.40 mmol, 1 eq) in TEA (0.72 g, 7.2 mmol, 3 eq) was added methyl 2,2,2-trifluoroacetate (0.30 g, 3.6 mmol,1.5 eq) and stirred at 25 °C for 6 h. Upon completion, the reaction mixture was concentrated in vacuum to remove most of methyl 2,2,2-trifluoroacetate and afford a residue. The combined residue was diluted with EtOAc (20 mL) and washed with 1 M aqueous HCl (20mL). The organic layer was seprated and the aqueous layer was extracted with EtOAc (20 ml * 2). The combined organic layer was dried over Na_2SO_4, filtered and concentrated under reduced pressure to give a residue. ^{1}H NMR (400 MHz, DMSO) δ 9.69 (d, *J* = 8.7 Hz, 1H), 4.43 (dd, *J* = 8.5, 4.1 Hz, 1H), 4.12 (s, 1H), 3.92 (ddd, *J* = 10.6, 5.5, 3.0 Hz, 2H), 3.67 (d, *J* = 10.6 Hz, 1H), 3.56 – 3.40 (m, 1H), 1.59 – 1.54 (m, 1H), 1.45 (d, *J* = 7.6 Hz, 1H), 1.17 (s, 3H), 1.12 (s, 9H), 1.00 (d, *J* = 6.8 Hz, 3H), 0.82 (s, 3H). ^{19}F NMR (377 MHz, DMSO) δ -73.74. ^{13}C NMR (101 MHz, DMSO) δ 172.56, 167.17, 156.97, 156.62, 74.10, 67.91, 60.22, 59.60, 56.84, 30.21, 28.89, 26.95, 26.16, 20.55, 19.24, 14.55, 12.46.

(1*R*,2*S*,5*S*)-3-(*O*-(*tert*-butyl)-*N*-(2,2,2-trifluoroacetyl)-*L*-threonyl)-*N*-((*S*)-1-cyano-2-((*S*)-2-oxopyrrolidin-3-yl)ethyl)-6,6-dimethyl-3-azabicyclo[3.1.0]hexane-2-carboxamide (MPI146): MPI146c (100 mg, 0.24 mmol) and Int **4** (70 mg, 0.36 mmol) were dissolved in DCM (6.0 ml) and COMU (157 mg, 0.36 mmol) was added at 0 °C. The reaction mixture was stirred at room temperature for 5 min and Hunig's base (0.12 mL 0.72 mmol) was added. The solution was stirred for 2 hours. The mixture was poured onto an aqueous sodium hydrogen carbonate (saturated, 20 ml) and water (20 ml). The mixture was stirred in an Erlenmeyer flask for 10 min. The aqueous layer was extracted with DCM. The organic layer was washed with brine and dried on $MgSO_4$. It was concentrated under reduced pressure. The crude product was purified by flash (eluted with 0:100 to 5:95 Methanol/ DCM) to give the title compound **MPI146** as a colorless solid (66 mg, 50%). ^{1}H NMR (400 MHz, DMSO) δ 9.64 (s, 1H), 8.91 (s, 1H), 7.64 (s, 1H), 4.91 – 4.87 (m, 1H), 4.74 – 4.68 (m, 1H), 4.32 (s, 1H), 4.06 (s, 1H), 3.94 – 3.90 (m, 1H), 3.81 (dd, *J* = 7.0, 5.0 Hz, 1H), 3.59 (s, 1H), 3.10 (s, 1H), 2.35 – 2.31 (m, 1H), 2.05 – 2.00 (m, 2H), 1.70 – 1.64 (m, 2H), 1.52 – 1.50 (m, 1H), 1.28 (s, 1H), 1.09 (s, 3H), 1.04 (s, 9H), 0.96 (s, 3H), 0.77 (s, 3H). ^{19}F NMR (376 MHz, $CDCl_3$) δ -75.67. ^{13}C NMR (101 MHz, $CDCl_3$) δ 179.21, 171.22, 167.40, 156.96, 156.59, 118.24, 75.86, 66.65, 61.02, 56.23, 53.45, 47.30, 40.62, 39.09, 38.41, 28.12, 25.97, 19.60, 18.83, 12.65. **HRMS- ESI** (m/z) [M+Na] calc'd for $C_{25}H_{36}F_3N_5O_5Na^+$, 566.2566, found 566.2550.

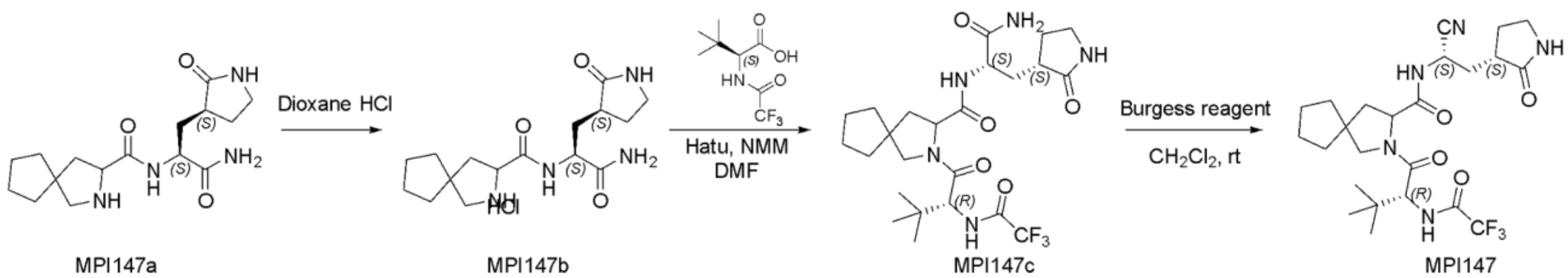

Scheme 14. The synthesis of **MPI147**

tert-butyl 3-(((S)-1-amino-1-oxo-3-((S)-2-oxopyrrolidin-3-yl)propan-2-yl)carbamoyl)-2-azaspiro[4.4]nonane-2-carboxylate (MPI147b): MPI147b was prepared as a white solid following general procedure F (Directly used for next step).

N-((S)-1-amino-1-oxo-3-((S)-2-oxopyrrolidin-3-yl)propan-2-yl)-2-((R)-3,3-dimethyl-2-(2,2,2-trifluoroacetamido)butanoyl)-2-azaspiro[4.4]nonane-3-carboxamide (MPI147c): MPI147c was prepared by using MPI147b and (S)-3,3-dimethyl-2-(2,2,2-trifluoroacetamido)butanoic acid as a white solid following general procedure C.

N-((S)-1-cyano-2-((S)-2-oxopyrrolidin-3-yl)ethyl)-2-((R)-3,3-dimethyl-2-(2,2,2-trifluoroacetamido)butanoyl)-2-azaspiro[4.4]nonane-3-carboxamide (MPI-147): (1:1 isomers): MPI147 was prepared as a white solid following general procedure D (yield 60%). ^{1}H NMR (400 MHz, DMSO) δ 9.24 (dd, *J* = 28.8, 8.1 Hz, 1H), 8.77 (dd, *J* = 45.9, 8.4 Hz, 1H), 7.61 (d, *J* = 19.4 Hz, 1H), 4.97 – 4.83 (m, 1H), 4.41 (dd, *J* = 8.1, 3.0 Hz, 1H), 4.22 – 4.09 (m, 1H), 3.63 (t, *J* = 11.0 Hz, 1H), 3.29 (d, *J* = 9.9 Hz, 1H), 3.12 – 2.92 (m, 2H), 2.25 – 2.14 (m, 1H), 2.02 (dddd, *J* = 36.4, 19.6, 9.3, 4.1 Hz, 3H), 1.74 – 1.32 (m, 12H), 1.26 (d, *J* = 14.8 Hz, 1H), 0.97 – 0.82 (m, 9H). **HRMS- ESI** (m/z) [M+H] calc'd for $C_{24}H_{35}F_3N_5O_4^+$, 514.2641, found 514.2637.

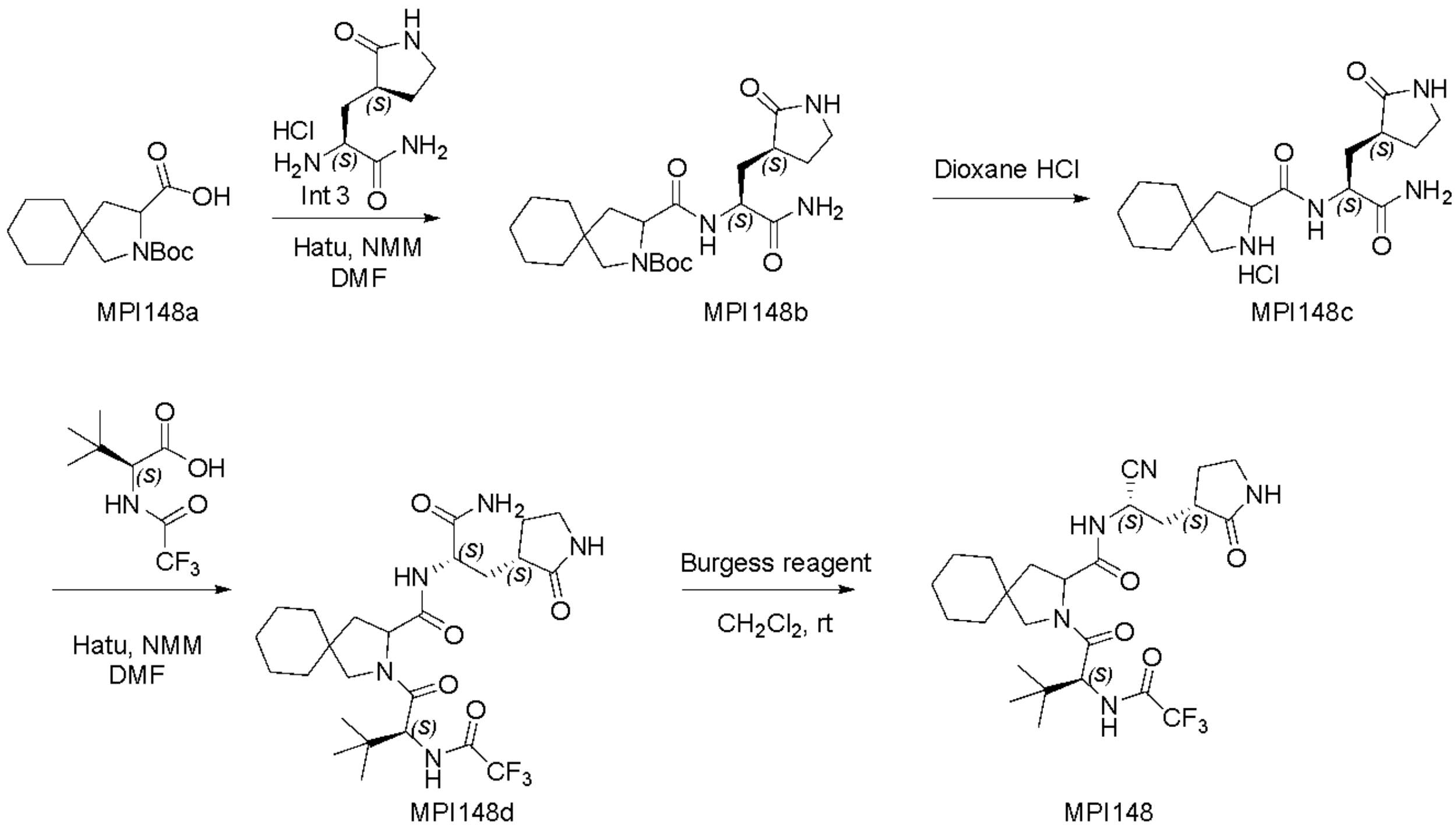

Scheme 15. The synthesis of **MPI148**

tert-butyl 3-(((S)-1-amino-1-oxo-3-((S)-2-oxopyrrolidin-3-yl)propan-2-yl)carbamoyl)-2-azaspiro[4.5]decane-2-carboxylate (MPI148b): MPI148b was prepared by using MPI148a and int.3 as a white solid following general procedure C. ^{1}H NMR (400 MHz, DMSO) δ 8.19 (t, J = 9.1 Hz, 0.5H), 7.94 (t, J = 7.7 Hz, 0.5H), 7.62 (d, J = 9.8 Hz, 1H), 7.28 (dd, J = 46.2, 25.0 Hz, 1H), 7.08 (d, J = 24.9 Hz, 1H), 4.41 – 4.07 (m, 2.5H), 3.20 – 2.96 (m, 3.5H), 2.26 – 1.89 (m, 4H), 1.77 – 1.47 (m, 3H), 1.47 – 1.23 (m, 19H).

N-((S)-1-amino-1-oxo-3-((S)-2-oxopyrrolidin-3-yl)propan-2-yl)-2-azaspiro[4.5]decane-3-carboxamide hydrogen chloride (MPI148c): MPI148c was prepared as a white solid following general procedure F (Directly used for next step).

N-((S)-1-amino-1-oxo-3-((S)-2-oxopyrrolidin-3-yl)propan-2-yl)-2-((S)-3,3-dimethyl-2-(2,2,2-trifluoroacetamido)butanoyl)-2-azaspiro[4.5]decane-3-carboxamide (MPI148d): MPI148d was prepared by using MPI148c and (S)-3,3-dimethyl-2-(2,2,2-trifluoroacetamido)butanoic acid as a white solid following general procedure C. (Yield 65%).^{1}H NMR (400 MHz, DMSO) δ 8.42 (d, J = 8.6 Hz, 1H), 8.16 – 8.06 (m, 1H), 7.58 (d, J = 26.1 Hz, 1H), 7.29 (s, 0H), 7.18 (s, 1H), 7.03 (d, J = 8.6 Hz, 1H), 4.53 (d, J = 20.9 Hz, 1H), 4.33 (dq, J = 17.3, 8.3 Hz, 1H), 4.20 – 4.08 (m, 1H), 3.85 (dd, J = 28.4, 10.1 Hz, 1H), 3.26 – 3.00 (m, 3H), 2.25 – 1.83 (m, 4H), 1.83 – 1.18 (m, 14H), 1.03 – 0.95 (m, 9H).

N-((S)-1-cyano-2-((S)-2-oxopyrrolidin-3-yl)ethyl)-2-((S)-3,3-dimethyl-2-(2,2,2-trifluoroacetamido)butanoyl)-2-azaspiro[4.5]decane-3-carboxamide (MPI148): (1:1 isomers): MPI148 was prepared as a white solid following general procedure D (yield 60%). ^{1}H NMR (400 MHz, DMSO) δ 9.30 (dd, *J* = 34.3, 8.1 Hz, 1H), 8.84 (dd, *J* = 44.4, 8.4 Hz, 1H), 7.69 (d, *J* = 20.0 Hz, 1H), 4.95 (dt, *J* = 11.7, 8.8 Hz, 1H), 4.54 (dd, *J* = 8.2, 4.3 Hz, 1H), 4.25 (dt, *J* = 15.8, 8.6 Hz, 1H), 3.84 (dd, *J* = 14.3, 10.2 Hz, 1H), 3.14 (ddd, *J* = 41.8, 31.4, 8.7 Hz, 3H), 2.31 – 2.02 (m, 3H), 1.73 (ddd, *J* = 21.5, 16.9, 9.4 Hz, 2H), 1.55 – 1.16 (m, 11H), 1.05 – 0.88 (m, 9H). **HRMS- ESI** (m/z) $[M+Na]^+$ calc'd for C25H36F3N5ONa+, 550.2617, found 550.2610.

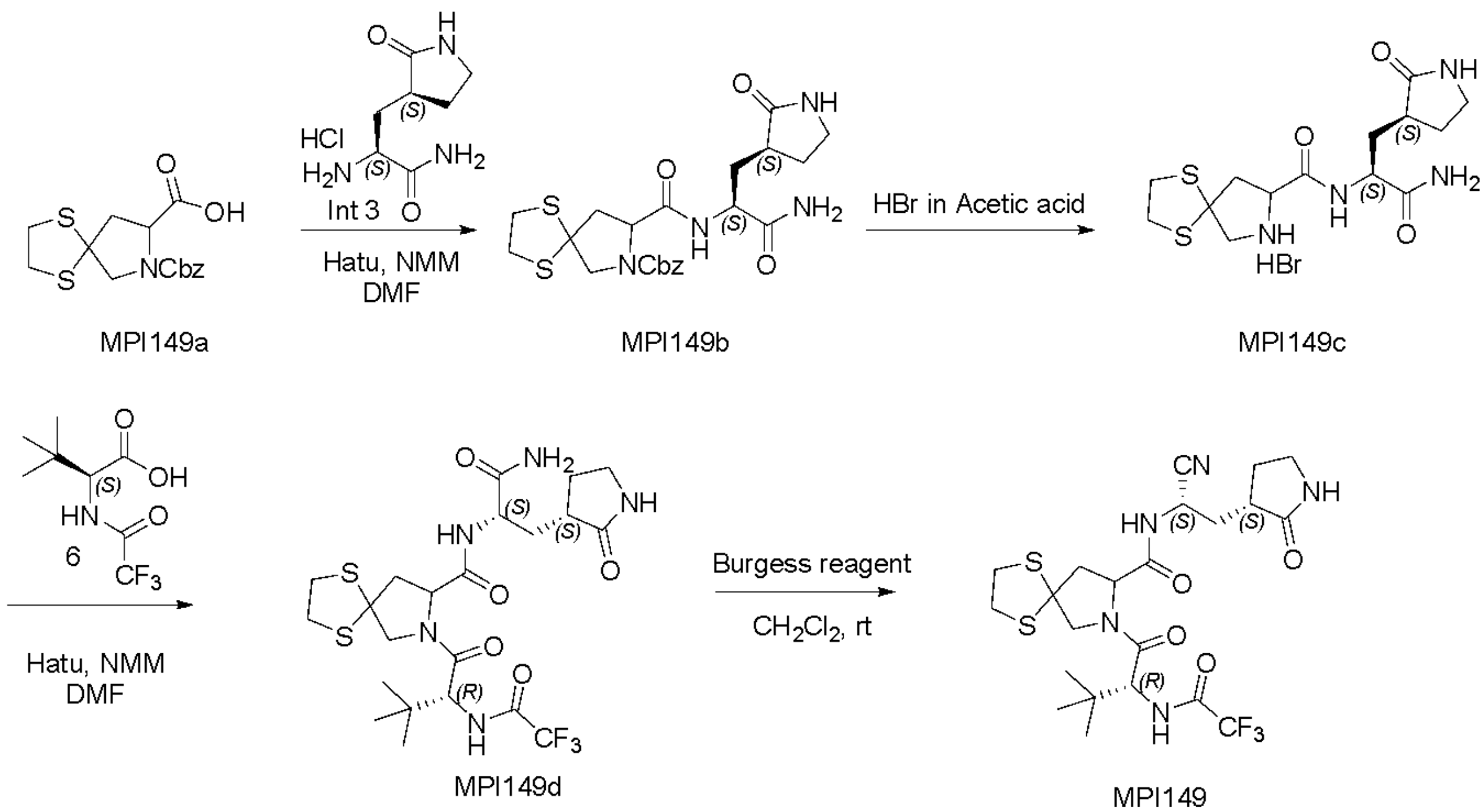

Scheme 16. The synthesis of **MPI149**

benzyl 8-(((S)-1-amino-1-oxo-3-((S)-2-oxopyrrolidin-3-yl)propan-2-yl)carbamoyl)-1,4-dithia-7-azaspiro[4.4]nonane-7-carboxylate (MPI149b):): MPI149b was prepared by using MPI149a and int.3 as a white solid following general procedure C. Directly proceeded for next step.

N-((S)-1-amino-1-oxo-3-((S)-2-oxopyrrolidin-3-yl)propan-2-yl)-1,4-dithia-7-azaspiro[4.4]nonane-8-carboxamide (MPI149c): The Cbz-protected amine was dissolved in glacial acetic acid (0.1 M) and cooled to 0 °C under an inert atmosphere. A solution of HBr in acetic acid (30–33 wt%, 3 eq) is added dropwise with stirring, and the reaction mixture is allowed to warm to room temperature and stirred for 1 h, monitoring completion by TLC. Upon complete consumption of the starting material, the solvent is removed under reduced pressure at low temperature, and the residue is triturated with cold diethyl ether to precipitate the amine hydrobromide salt. The solid is collected by filtration, washed with ether, and dried under vacuum. ^{1}H NMR (400 MHz, DMSO) δ 9.83 (s, 1H), 9.14 – 8.85 (m, 1H), 8.75 (d, *J* = 8.1 Hz, 1H), 7.66 (s, 1H), 7.51 (s, 1H), 7.12 (s, 1H), 4.42 (t, *J* = 8.0 Hz,

1H), 4.33 – 4.23 (m, 1H), 3.64 (p, *J* = 5.9 Hz, 2H), 3.38 (dqd, *J* = 9.5, 6.6, 3.9 Hz, 4H), 3.22 – 3.05 (m, 2H), 2.87 (dd, *J* = 13.5, 7.7 Hz, 1H), 2.48 – 2.38 (m, 1H), 2.32 – 2.10 (m, 2H), 1.98 (ddd, *J* = 14.2, 10.9, 3.7 Hz, 1H), 1.71 (dq, *J* = 12.0, 8.9 Hz, 1H), 1.62 – 1.49 (m, 1H). ^{13}C NMR (101 MHz, DMSO) δ 178.57, 173.07, 167.04, 66.87, 59.84, 58.76, 51.96, 45.28, 38.16, 34.24, 27.68.

N-((S)-1-amino-1-oxo-3-((S)-2-oxopyrrolidin-3-yl)propan-2-yl)-7-((R)-3,3-dimethyl-2-(2,2,2-trifluoroacetamido)butanoyl)-1,4-dithia-7-azaspiro[4.4]nonane-8-carboxamide (MPI149d): MPI149d was prepared by using MPI148c and (S)-3,3-dimethyl-2-(2,2,2-trifluoroacetamido)butanoic acid as a white solid following general procedure C. (Yield 58%). ^{1}H NMR (400 MHz, DMSO) δ 9.48 (d, *J* = 8.8 Hz, 1H), 8.33 (d, *J* = 8.9 Hz, 1H), 7.56 (s, 1H), 7.31 (s, 1H), 7.04 (s, 1H), 4.55 (d, *J* = 8.9 Hz, 1H), 4.47 (dd, *J* = 9.8, 7.2 Hz, 1H), 4.30 (ddd, *J* = 12.2, 8.6, 3.4 Hz, 1H), 4.18 (d, *J* = 10.9 Hz, 1H), 3.86 (d, *J* = 10.9 Hz, 1H), 3.47 – 3.27 (m, 4H), 3.14 (t, *J* = 9.1 Hz, 1H), 3.05 (td, *J* = 9.3, 7.1 Hz, 1H), 2.65 – 2.56 (m, 1H), 2.45 (d, *J* = 11.7 Hz, 1H), 2.29 (dd, *J* = 13.0, 9.9 Hz, 1H), 2.24 – 2.13 (m, 1H), 2.02 – 1.89 (m, 1H), 1.65 (dq, *J* = 12.1, 9.2 Hz, 1H), 1.49 (ddd, *J* = 14.1, 11.6, 3.5 Hz, 1H), 0.99 (s, 9H). ^{13}C NMR (101 MHz, DMSO) δ 179.19, 173.80, 170.44, 167.98, 157.35, 156.98, 117.77, 114.91, 67.46, 62.90, 60.52, 58.34, 55.38, 50.80, 44.83, 37.73, 35.70, 34.78, 27.93, 26.57.

N-((S)-1-cyano-2-((S)-2-oxopyrrolidin-3-yl)ethyl)-7-((R)-3,3-dimethyl-2-(2,2,2-trifluoroacetamido)butanoyl)-1,4-dithia-7-azaspiro[4.4]nonane-8-carboxamide (YR-D-18): MPI149 was prepared as a white solid following general procedure D (yield 60%). ^{1}H NMR (400 MHz, DMSO) δ 9.47 (d, *J* = 8.7 Hz, 1H), 9.05 (d, *J* = 8.6 Hz, 1H), 7.68 (s, 1H), 4.97 (ddd, *J* = 11.0, 8.5, 4.9 Hz, 1H), 4.53 (d, *J* = 8.7 Hz, 1H), 4.33 (dd, *J* = 9.9, 7.1 Hz, 1H), 4.20 (d, *J* = 11.0 Hz, 1H), 3.91 (d, *J* = 10.9 Hz, 1H), 3.53 – 3.35 (m, 3H), 3.15 (t, *J* = 9.1 Hz, 1H), 3.06 (td, *J* = 9.3, 7.0 Hz, 1H), 2.66 – 2.57 (m, 1H), 2.35 – 2.25 (m, 1H), 2.21 – 2.06 (m, 2H), 1.78 – 1.64 (m, 2H), 0.98 (s, 9H). **HRMS- ESI** (m/z) $[M+H]^+$ calc'd for $C_{22}H_{31}F_3N_5O_4S_2^+$, 550.1770, found 550.1766.

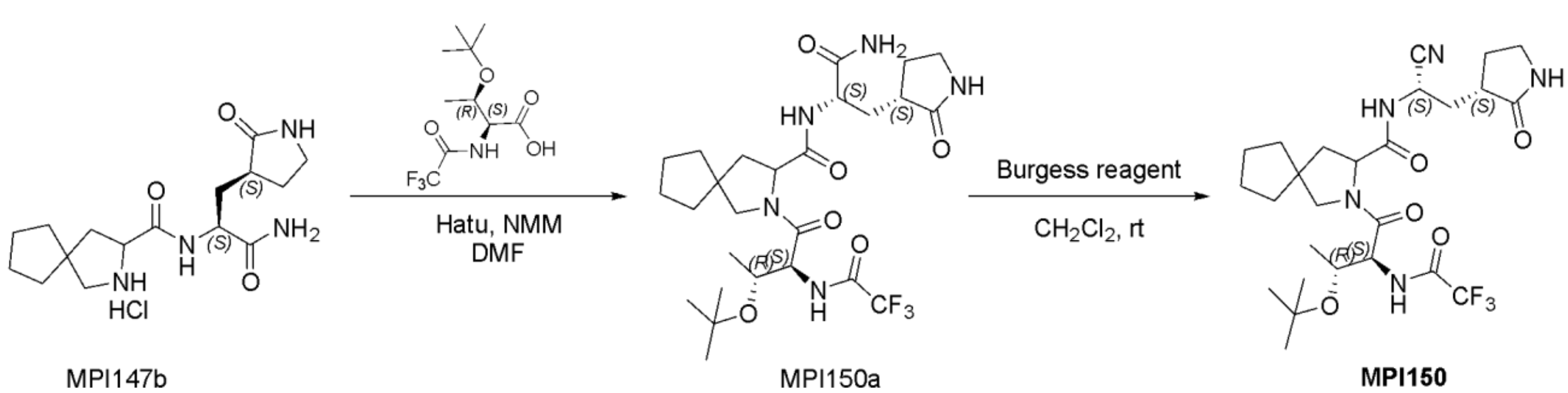

Scheme 17. The synthesis of **MPI150**

N-((S)-1-amino-1-oxo-3-((S)-2-oxopyrrolidin-3-yl)propan-2-yl)-2-(O-(tert-butyl)-N-(2,2,2-trifluoroacetyl)-L-threonyl)-2-azaspiro[4.4]nonane-3-carboxamide (6): To a stirred solution of compound MPI147b (100 mg, 0.28 mmol), O-(tert-butyl)-N-(2,2,2-trifluoroacetyl)-L-threonine (91 mg, 336 ml) in anhydrous DMF (10 mL) was

added NMM (6.46 mmol, 0.123 mL) and was cooled to 0 °C. HATU (1.93 mmol, 149 mg) was added to the solution under 0 °C and then stirred at room temperature overnight. The reaction mixture was then diluted with ethyl acetate (20 mL) and washed with saturated $NaHCO_3$ solution (2×10 mL), 1 M HCl solution (2×10 mL), and saturated brine solution (2×10 mL) sequentially. The organic layer was dried over anhydrous Na_2SO_4 and then concentrated *on vacuo*. The residue was then purified with flash chromatography (0-10% MeOH in DCM as the eluent) to afford **MPI150a** as gummy solid (70 mg, 44%).

2-(O-(tert-butyl)-N-(2,2,2-trifluoroacetyl)-L-threonyl)-N-((S)-1-cyano-2-((S)-2-oxopyrrolidin-3-yl)ethyl)-2-azaspiro[4.4]nonane-3-carboxamide (MPI150) (isomers): MPI150 was prepared as a white solid following general procedure D (yield 60%).^{1}H NMR (400 MHz, DMSO) δ 9.68 (dd, *J* = 18.4, 8.5 Hz, 1H), 8.86 (d, *J* = 8.3 Hz, 1H), 7.69 (d, *J* = 8.9 Hz, 1H), 5.02 – 4.89 (m, 1H), 4.46 (t, *J* = 8.2 Hz, 1H), 4.24 – 4.12 (m, 1H), 3.93 – 3.85 (m, 1H), 3.68 – 3.50 (m, 2H), 3.22 – 3.10 (m, 2H), 2.22 – 1.99 (m, 3H), 1.84 – 1.32 (m, 12H), 1.17 – 0.99 (m, 12H).). **HRMS- ESI** (m/z) $[M+H]^+$ calc'd for $C_{22}H_{31}F_3N_5O_4S_2$+, 558.2903, found 558.2880.

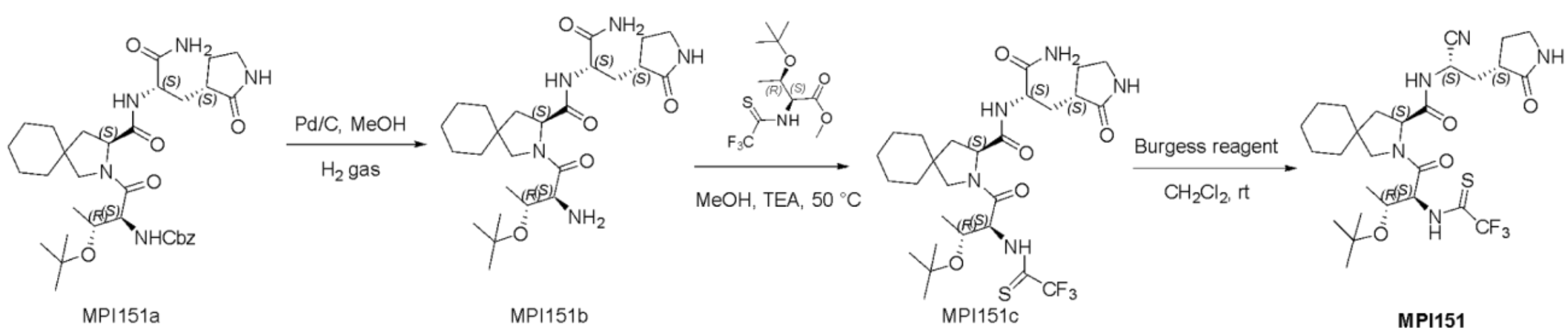

Scheme 18. The synthesis of **MPI151**

2-(O-(tert-butyl)-N-(2,2,2-trifluoroethanethioyl)-L-threonyl)-N-((S)-1-cyano-2-((S)-2-oxopyrrolidin-3-yl)ethyl)-2-azaspiro[4.5]decane-3-carboxamide(MPI-151): MPI151 was prepared as a white solid following procedures MPI145d and general procedure D (overall yield 35%).^{1}H NMR (400 MHz, DMSO) δ 11.32 (d, *J* = 7.5 Hz, 1H), 8.90 (d, *J* = 8.3 Hz, 1H), 7.76 – 7.68 (m, 1H), 5.06 – 4.88 (m, 1H), 4.23 (dt, *J* = 9.8, 6.4 Hz, 1H), 4.17 – 4.04 (m, 2H), 3.22 – 3.04 (m, 2H), 2.47 – 2.36 (m, 1H), 2.21 – 1.97 (m, 3H), 1.81 – 1.63 (m, 2H), 1.58 – 1.30 (m, 11H), 1.14 (s, 9H). **HRMS- ESI** (m/z) $[M+H]^+$ calc'd for $C_{27}H_{41}F_3N_5O_4S$+, 588.2831, found 588.2810.

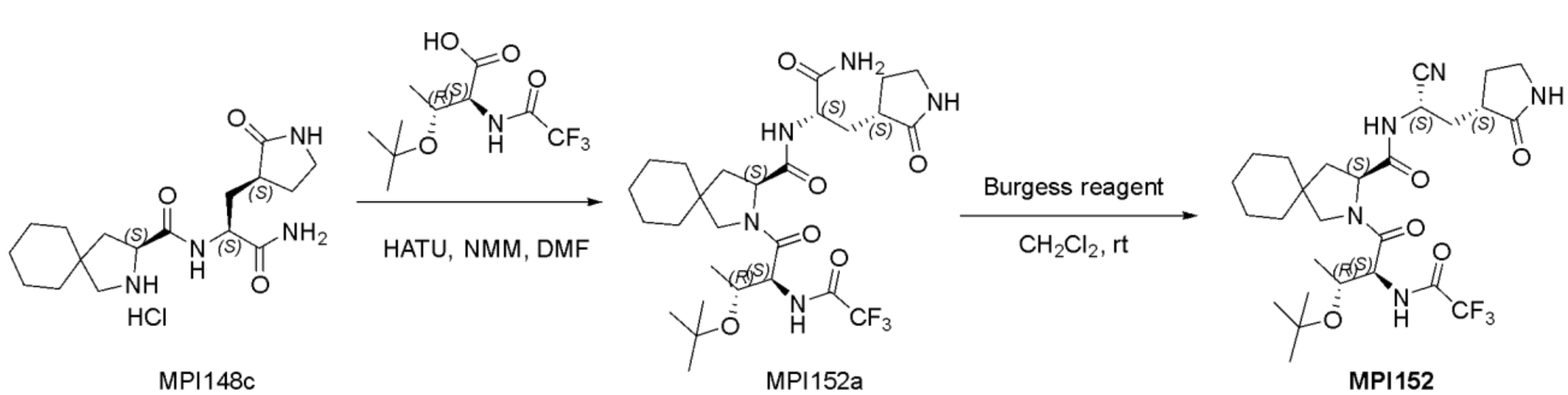

Scheme 19. The synthesis of **MPI152**

N-((S)-1-amino-1-oxo-3-((S)-2-oxopyrrolidin-3-yl)propan-2-yl)-2-(O-(tert-butyl)-N-(2,2,2-trifluoroacetyl)-L-threonyl)-2-azaspiro[4.5]decane-3-carboxamide (MPI152a): MPI152a was prepared as a white solid following same procedure of MPI150a (yield 40%).

2-(O-(tert-butyl)-N-(2,2,2-trifluoroacetyl)-L-threonyl)-N-((S)-1-cyano-2-((S)-2-oxopyrrolidin-3-yl)ethyl)-2-azaspiro[4.5]decane-3-carboxamide (MPI152):): MPI152 was prepared as a white solid following general procedure D (yield 50%).^{1}H NMR (400 MHz, DMSO) δ 9.67 (d, J = 8.5 Hz, 1H), 8.87 (d, J = 8.4 Hz, 1H), 7.71 (d, J = 7.8 Hz, 1H), 5.01 – 4.84 (m, 1H), 4.50 (t, J = 8.3 Hz, 1H), 4.21 (dd, J = 9.6, 7.6 Hz, 1H), 3.94 – 3.83 (m, 1H), 3.78 (d, J = 10.3 Hz, 1H), 3.30 – 3.18 (m, 3H), 2.12 (dq, J = 13.1, 6.4 Hz, 3H), 1.81 – 1.62 (m, 2H), 1.54 – 1.23 (m, 12H), 1.16 (d, J = 6.7 Hz, 3H), 1.14 – 1.10 (m, 9H). **HRMS- ESI** (m/z) $[M+H]^+$ calc'd for $C_{27}H_{41}F_3N_5O_5$+, 572.3060, found 572.3036.

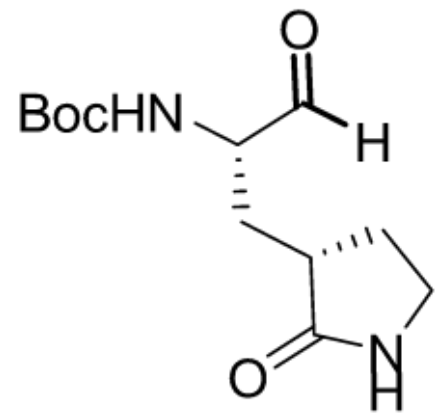
BocHN
O
H
O
N
H

MPI 134b

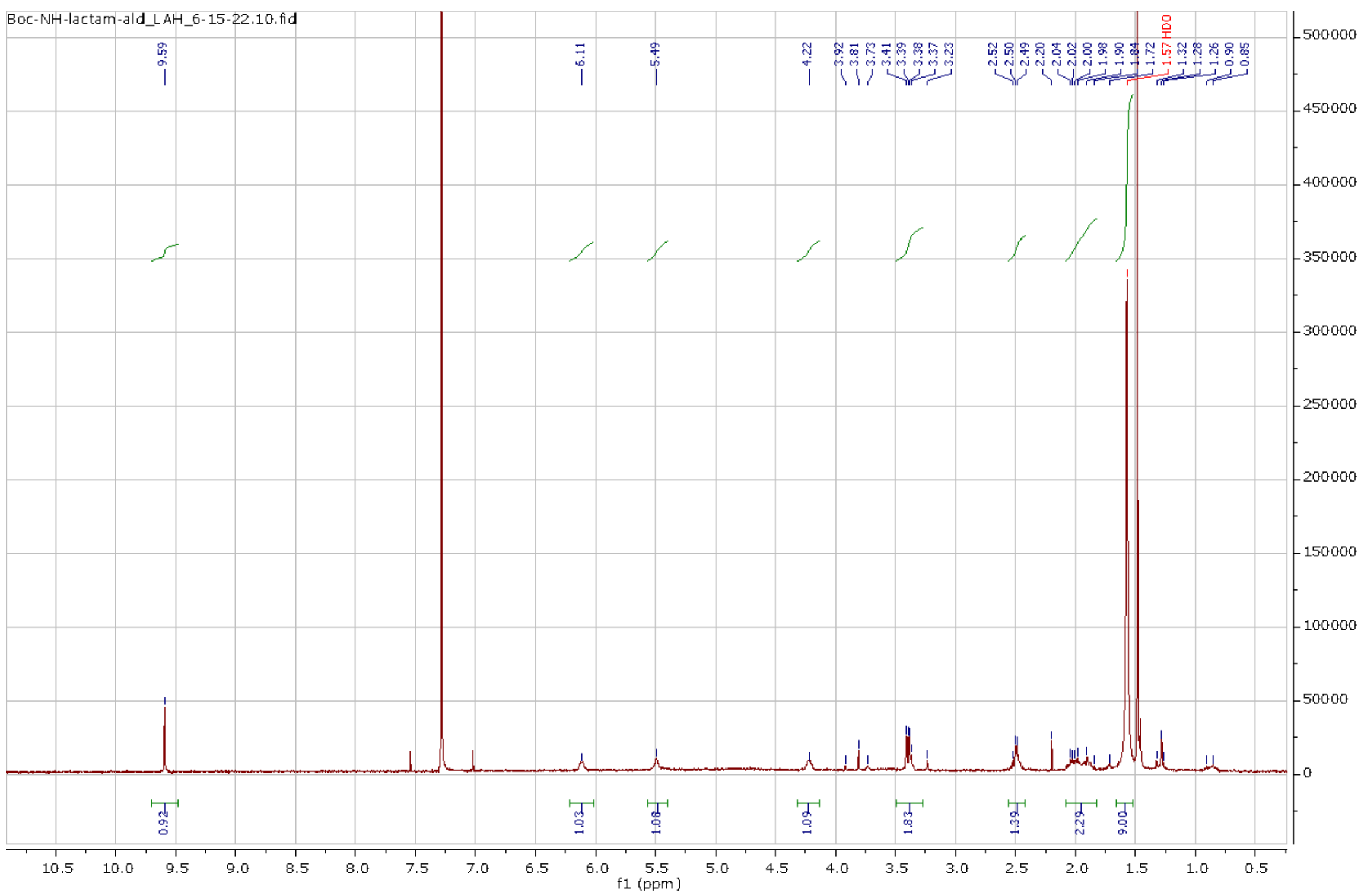
Boc-NH-lactam-ald_LAH_6-15-22.10.fid
9.59
6.11
5.49
4.22
1.57 HDO
0.92
1.03
1.08
1.09
1.83
1.39
2.29
9.00
f1 (ppm)

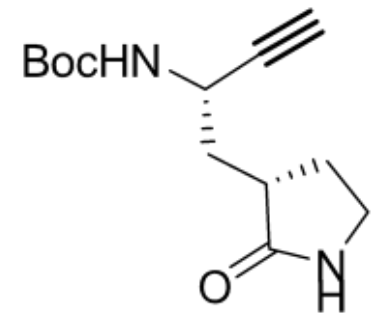

MPI134c

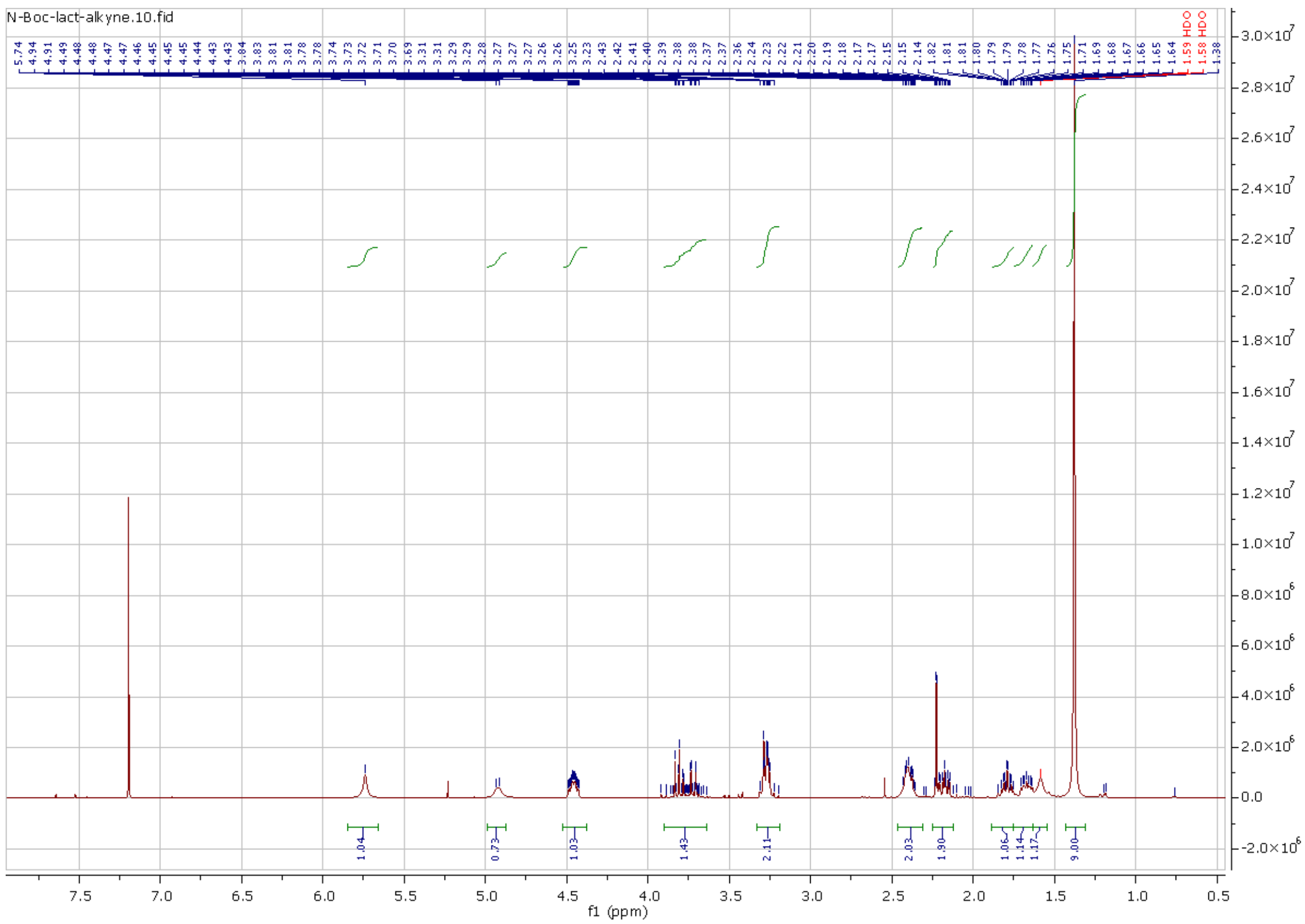

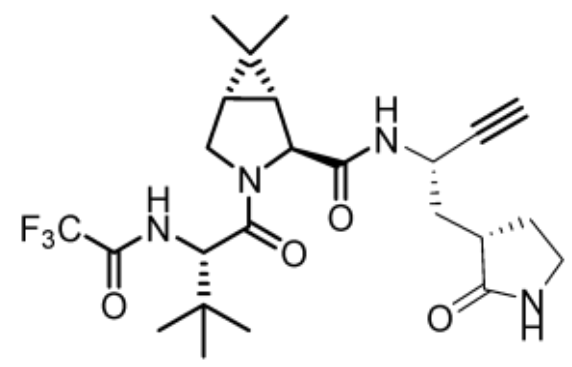

MPI 134

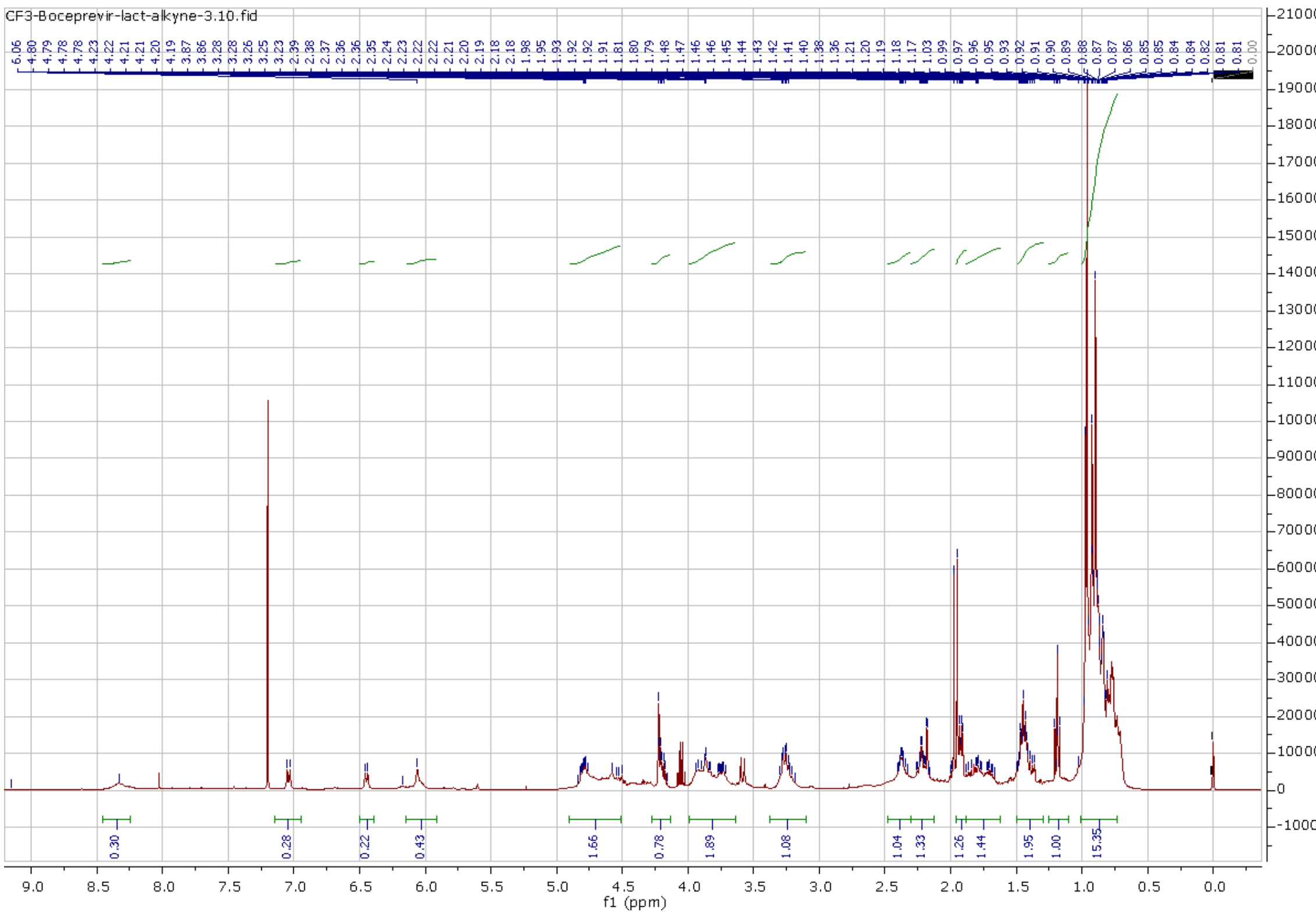

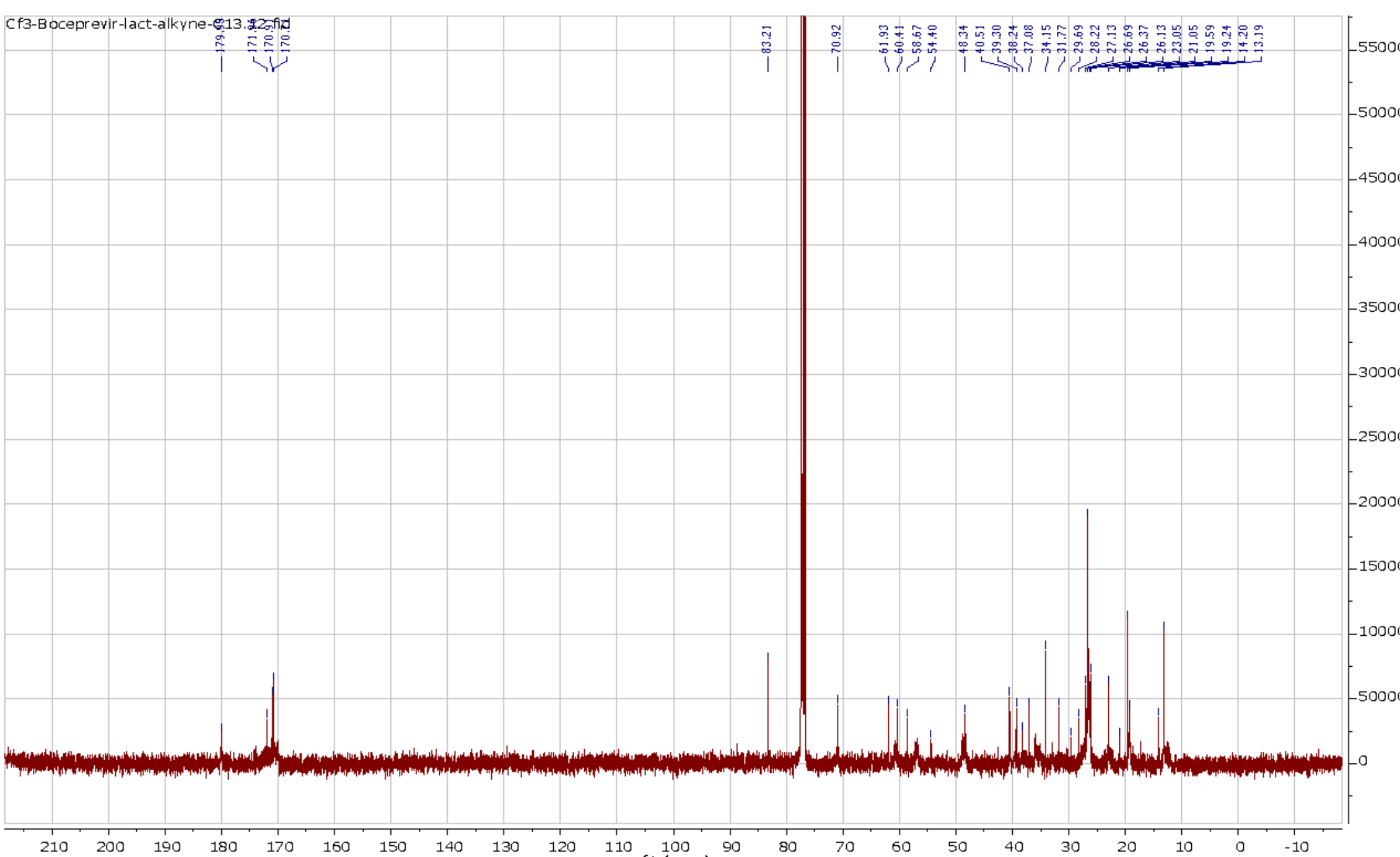

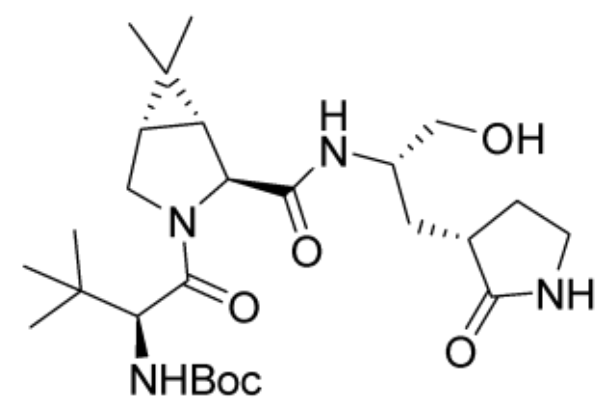

MPI136b

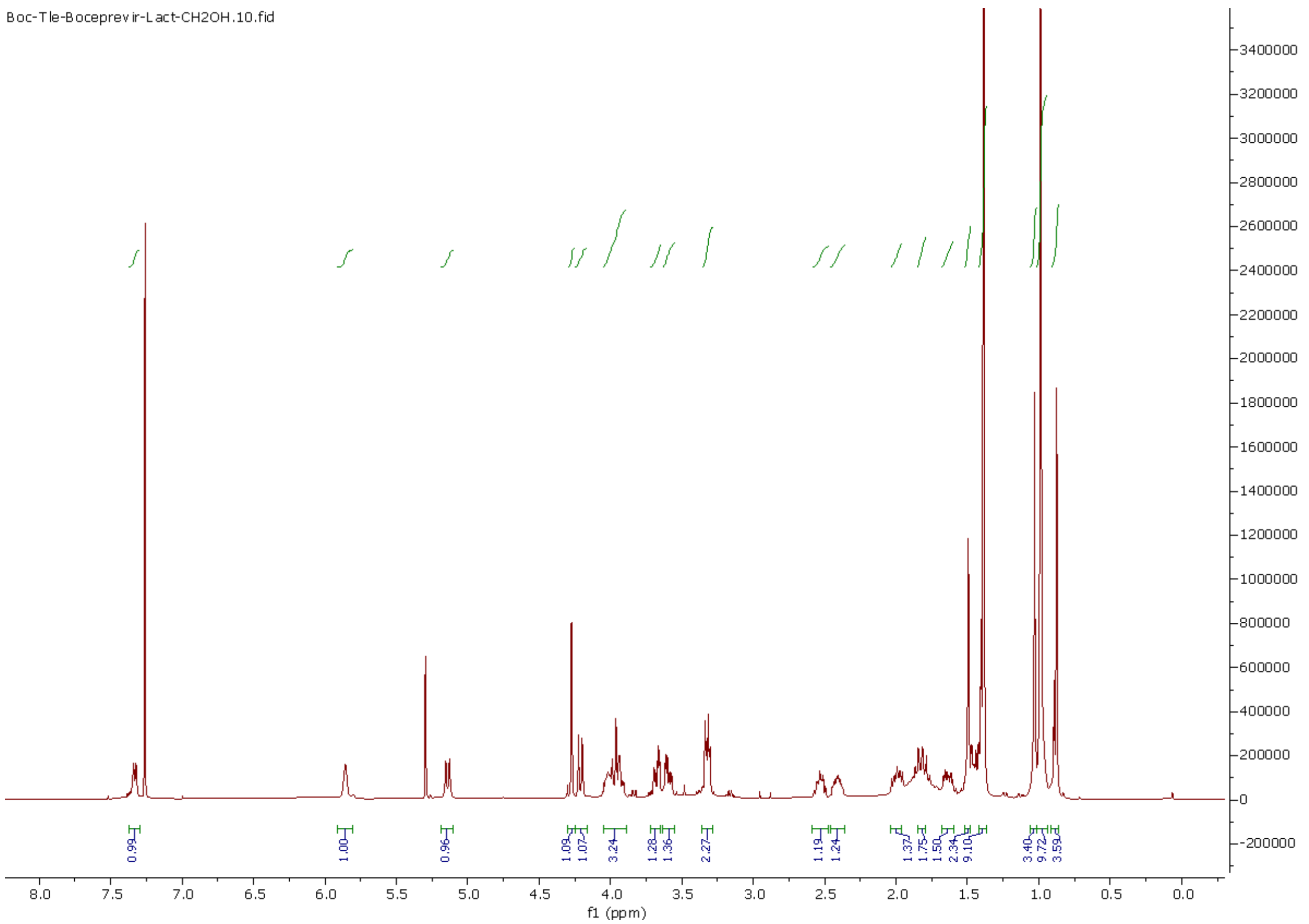

Boc-Tle-Boceprevir-Lact-CH2OH.10.fid
0.99
1.00
0.96
1.09
1.07
3.24
1.28
1.36
2.27
1.19
1.24
1.37
1.75
1.50
2.34
9.10
3.40
9.72
3.59
f1 (ppm)

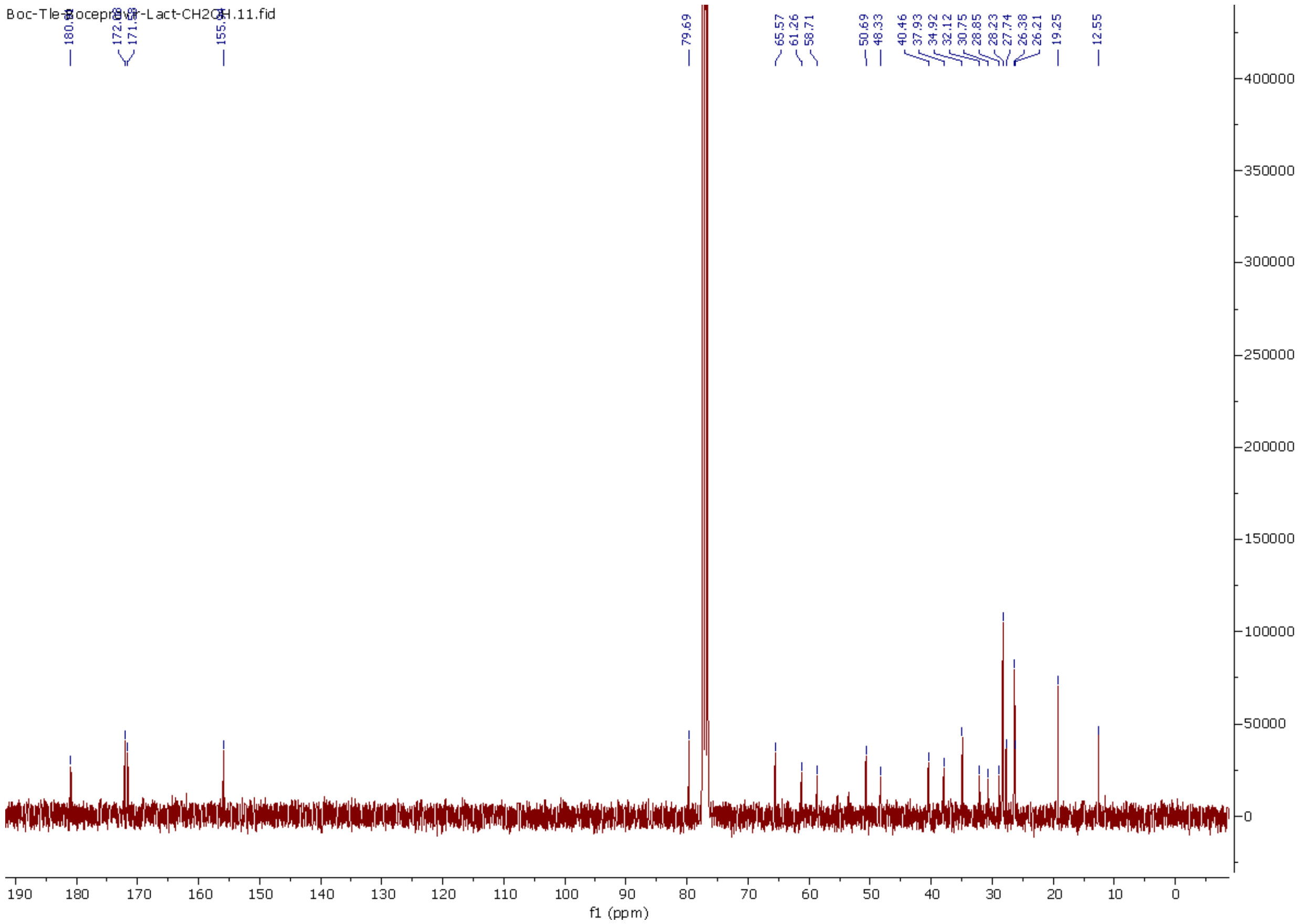

Boc-Tle-Boceprevir-Lact-CH2OH.11.fid
180.9
172.08
171.59
155.94
79.69
65.57
61.26
58.71
50.69
48.33
40.46
37.93
34.92
32.12
30.75
28.85
28.23
27.74
26.38
26.21
19.25
12.55
400000
350000
300000
250000
200000
150000
100000
50000
0
190 180 170 160 150 140 130 120 110 100 90 80 70 60 50 40 30 20 10 0
f1 (ppm)

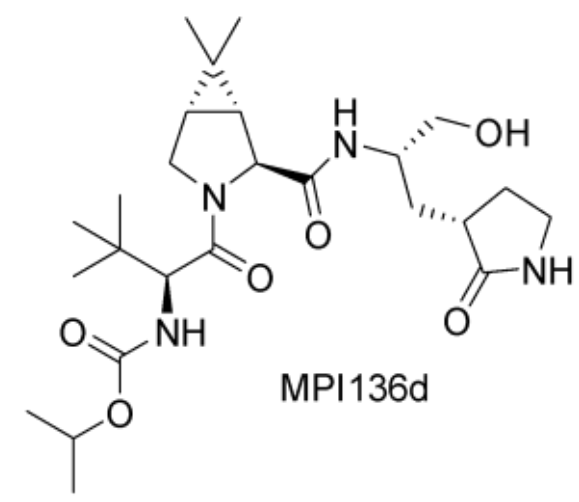

MPI136d

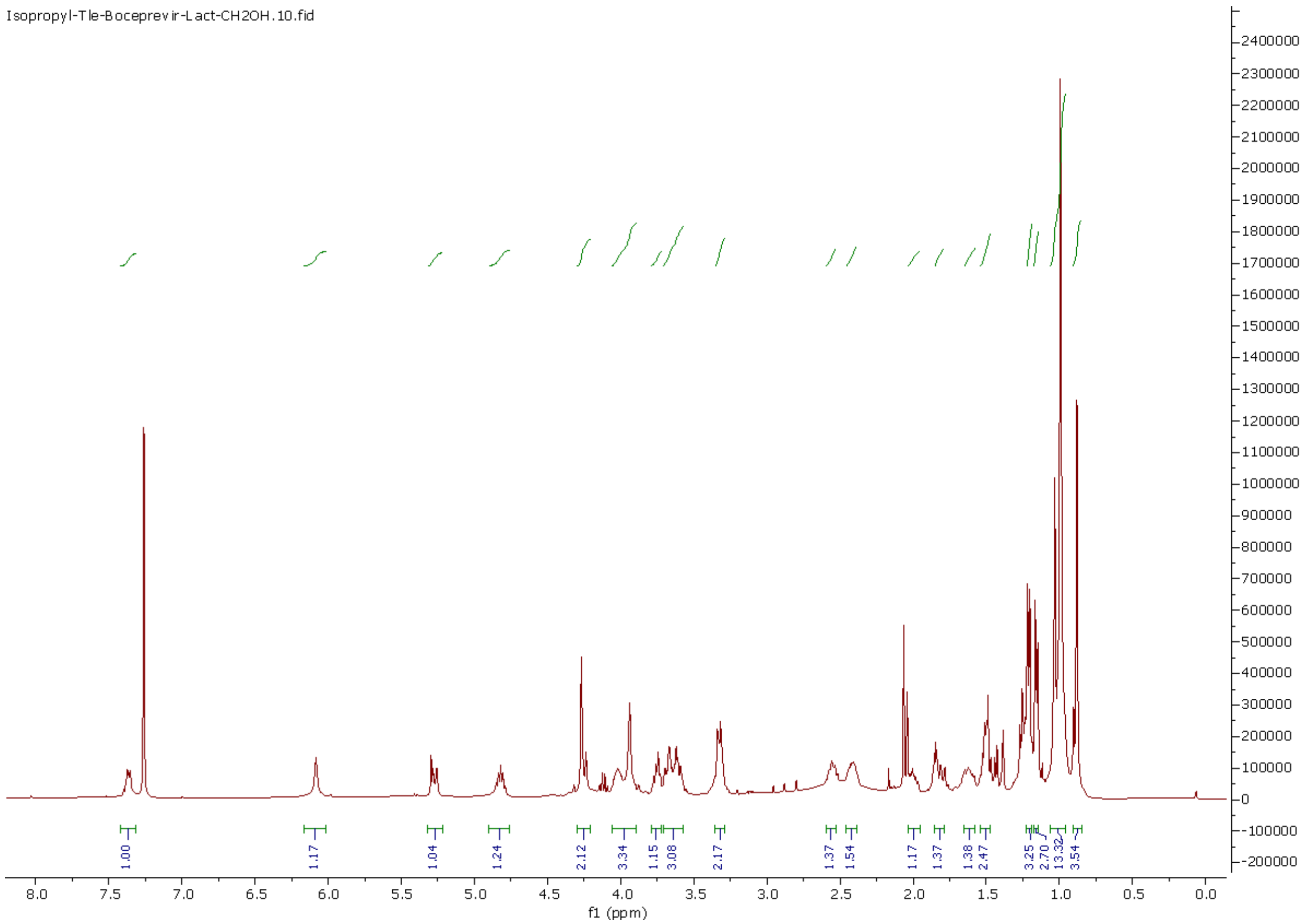
Isopropyl-Tle-Boceprevir-Lact-CH2OH.10.fid
1.00
1.17
1.04
1.24
2.12
3.34
1.15
3.08
2.17
1.37
1.54
1.17
1.37
1.38
2.47
3.25
2.70
13.32
3.54
f1 (ppm)

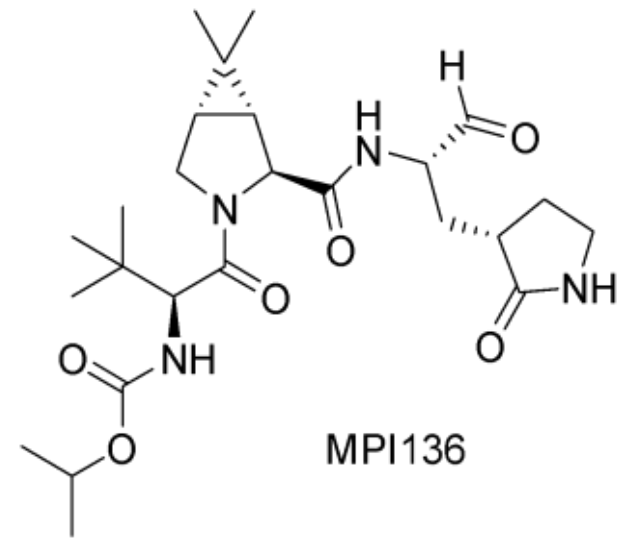

MPI136

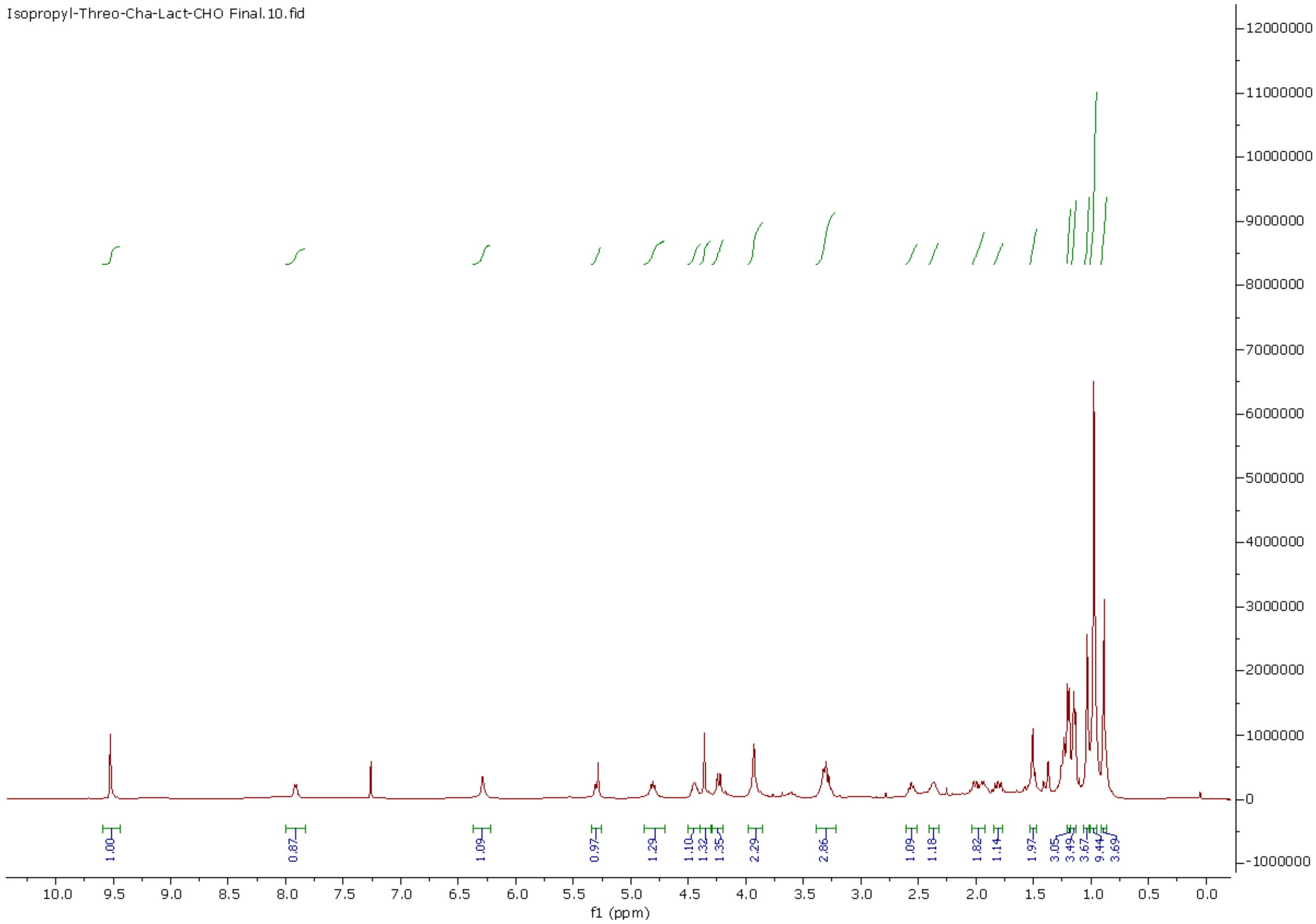

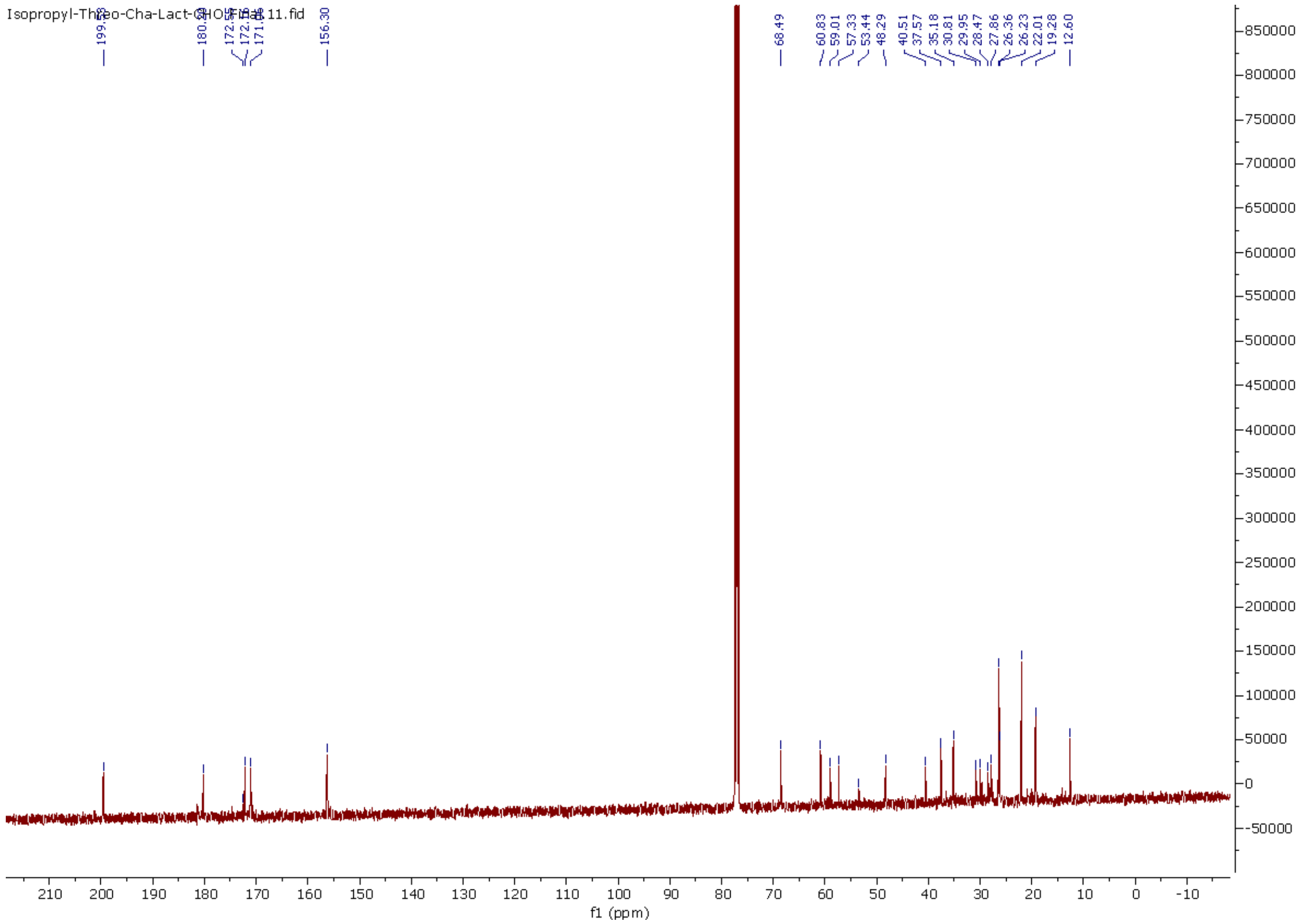

Isopropyl-Threo-Cha-Lact-CHO.Final.11.fid
156.30
68.49
60.83
59.01
57.33
53.44
48.29
40.51
37.57
35.18
30.81
29.95
28.47
27.86
26.36
26.23
22.01
19.28
12.60
f1 (ppm)

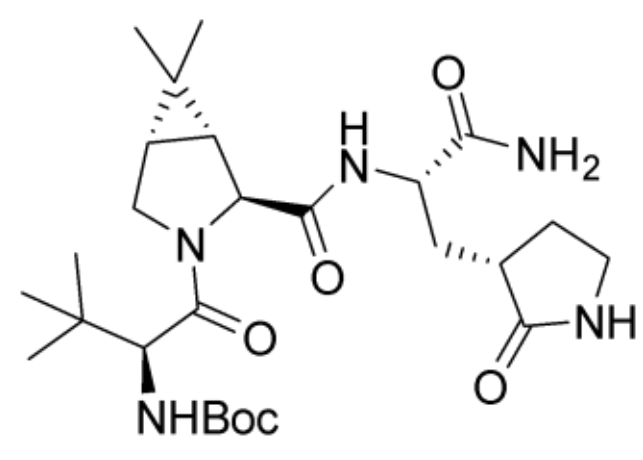

O
H
N
NH2
N
O
O
NH
O
NHBoc

MPI137a

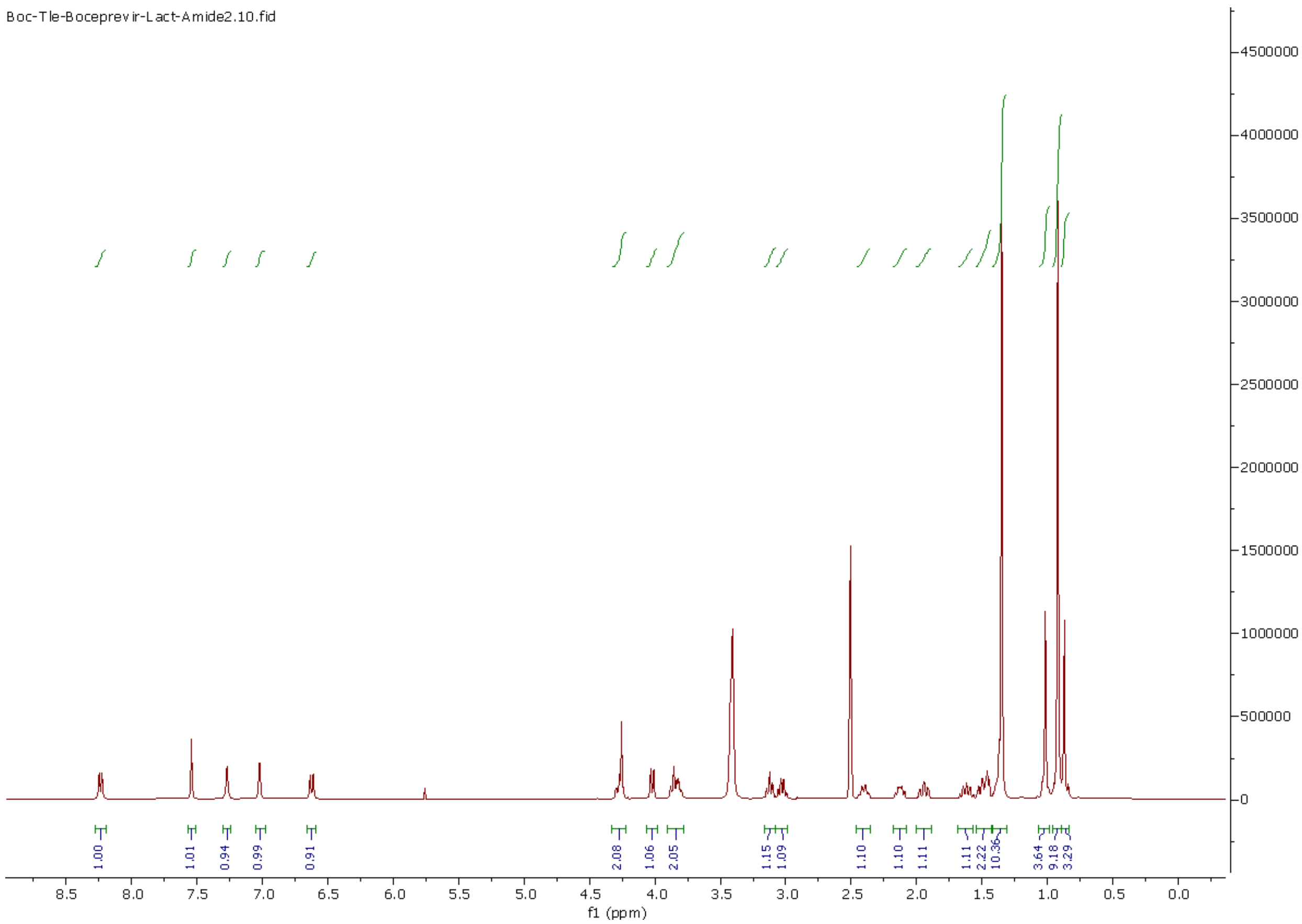

Boc-Tle-Boceprevir-Lact-Amide2.10.fid
4500000
4000000
3500000
3000000
2500000
2000000
1500000
1000000
500000
0
1.00
1.01
0.94
0.99
0.91
2.08
1.06
2.05
1.15
1.09
1.10
1.10
1.11
1.11
2.22
10.36
3.64
9.18
3.29
8.5
8.0
7.5
7.0
6.5
6.0
5.5
5.0
4.5
4.0
3.5
3.0
2.5
2.0
1.5
1.0
0.5
0.0
f1 (ppm)

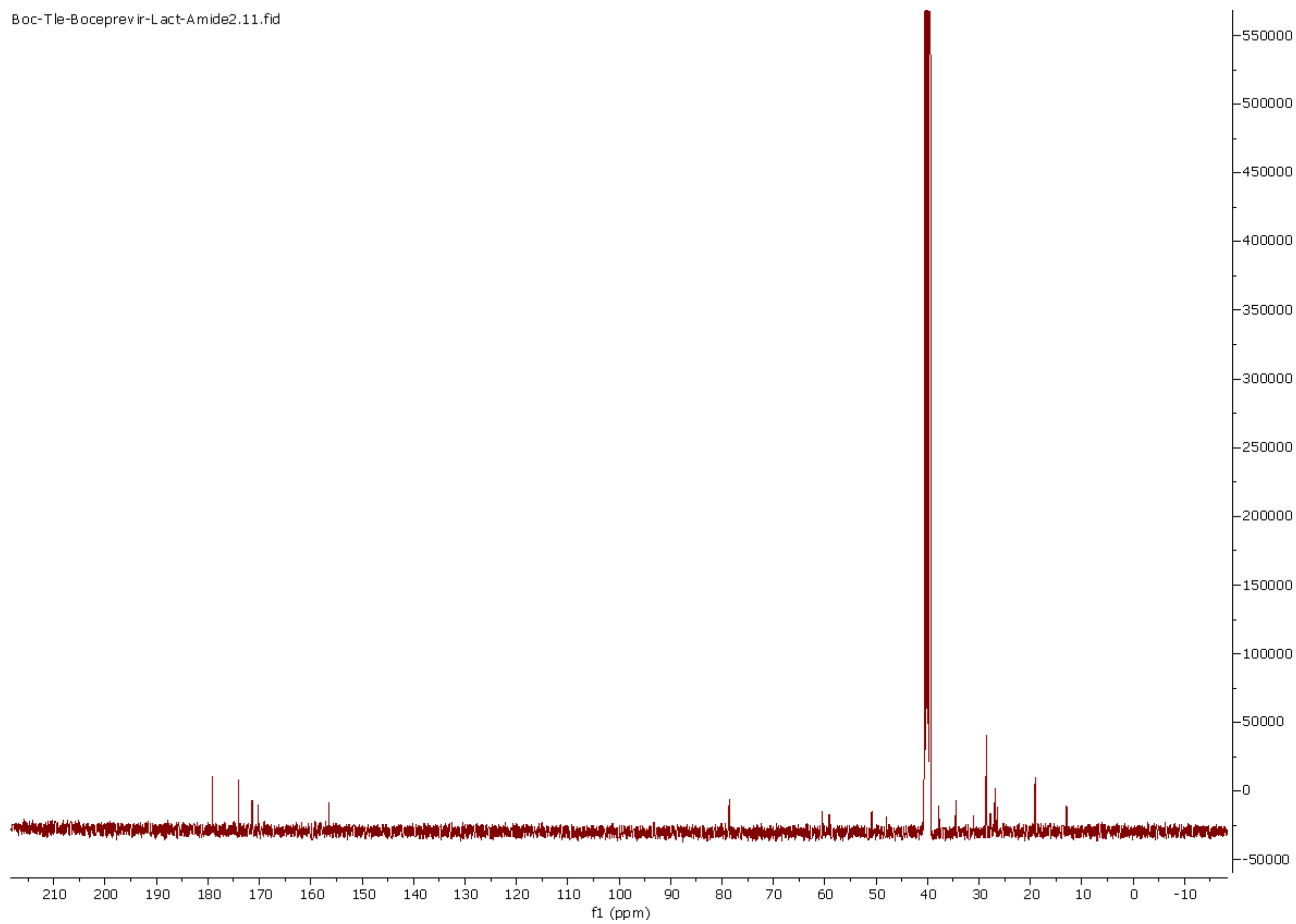

Boc-Tle-Boceprevir-Lact-Amide2.11.fid
550000
500000
450000
400000
350000
300000
250000
200000
150000
100000
50000
0
-50000
210 200 190 180 170 160 150 140 130 120 110 100 90 80 70 60 50 40 30 20 10 0 -10
f1 (ppm)

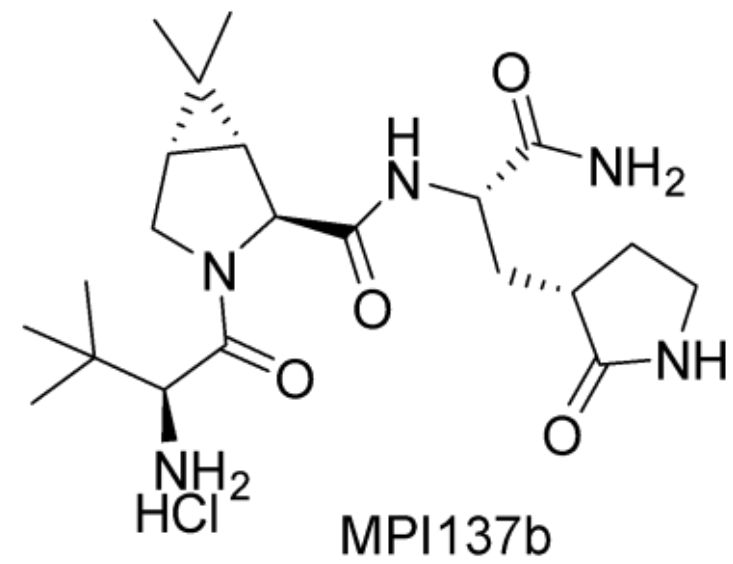

MPI137b

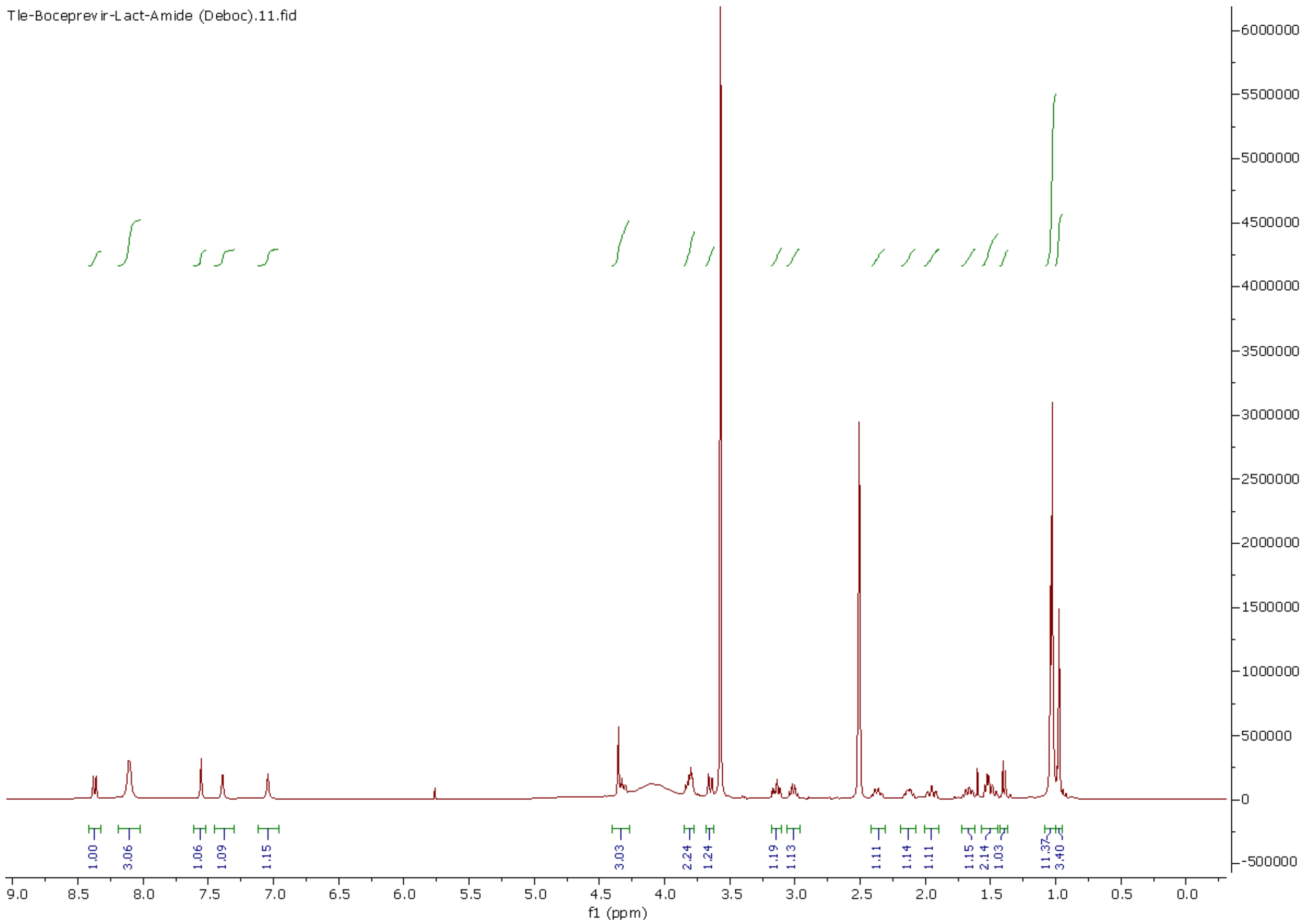

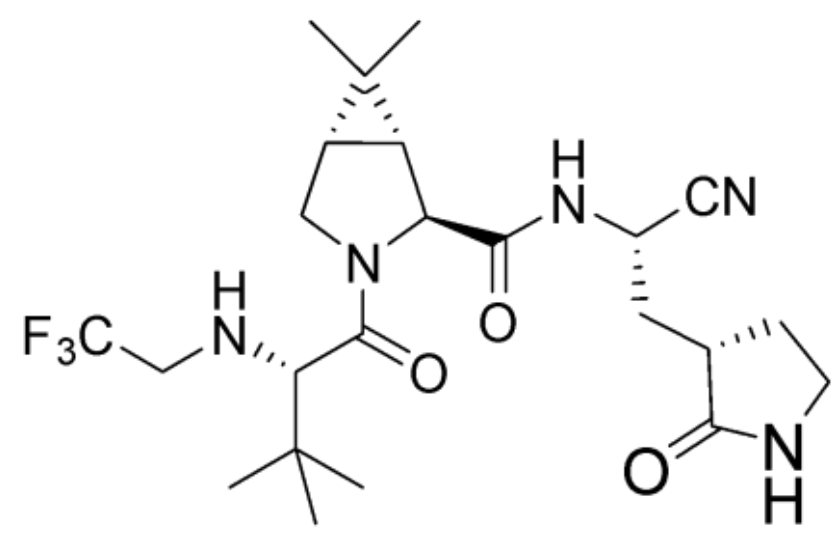
F3C
H
N
O
N
O
H
N
CN
O
N
H

MPI137

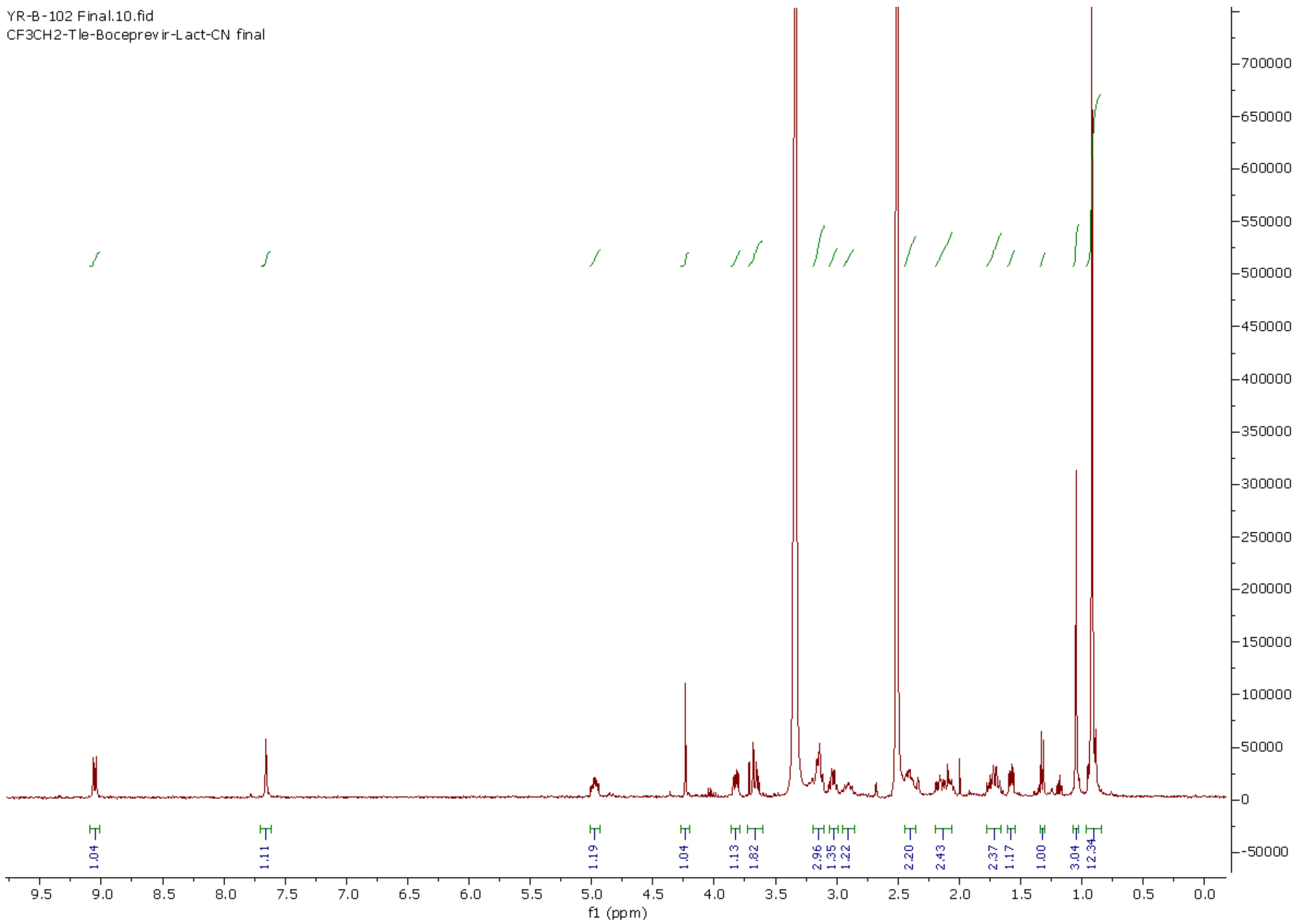
YR-B-102 Final.10.fid
CF3CH2-Tle-Boceprevir-Lact-CN final
1.04
1.11
1.19
1.04
1.13
1.82
2.96
1.35
1.22
2.20
2.43
2.37
1.17
1.00
3.04
12.34
f1 (ppm)

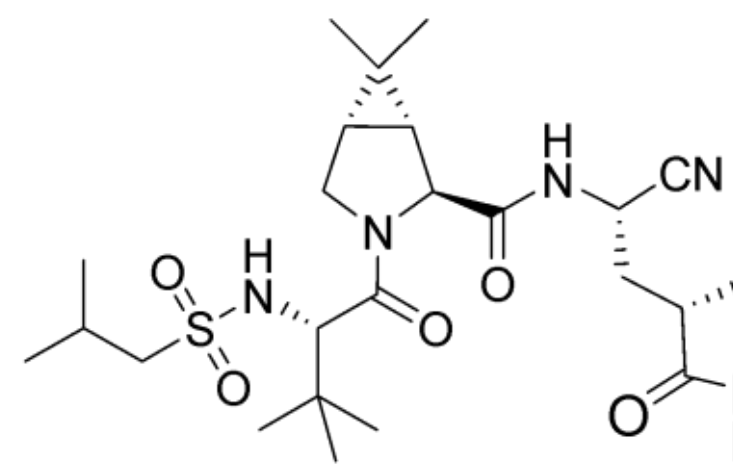

MPI138

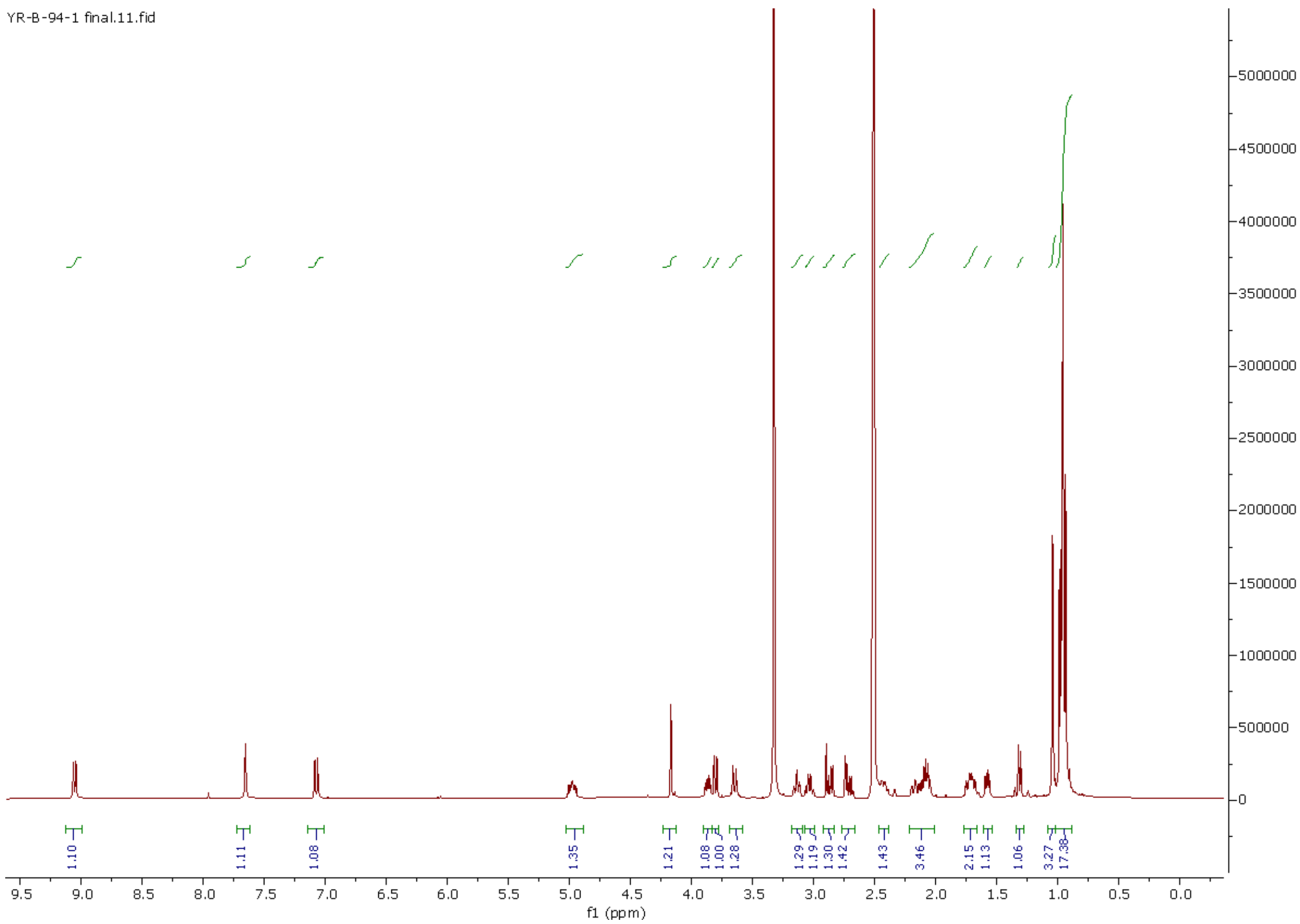

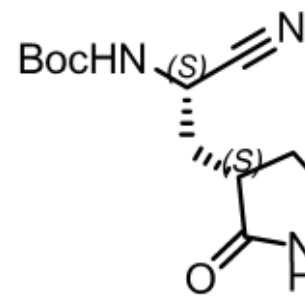

MPI139h

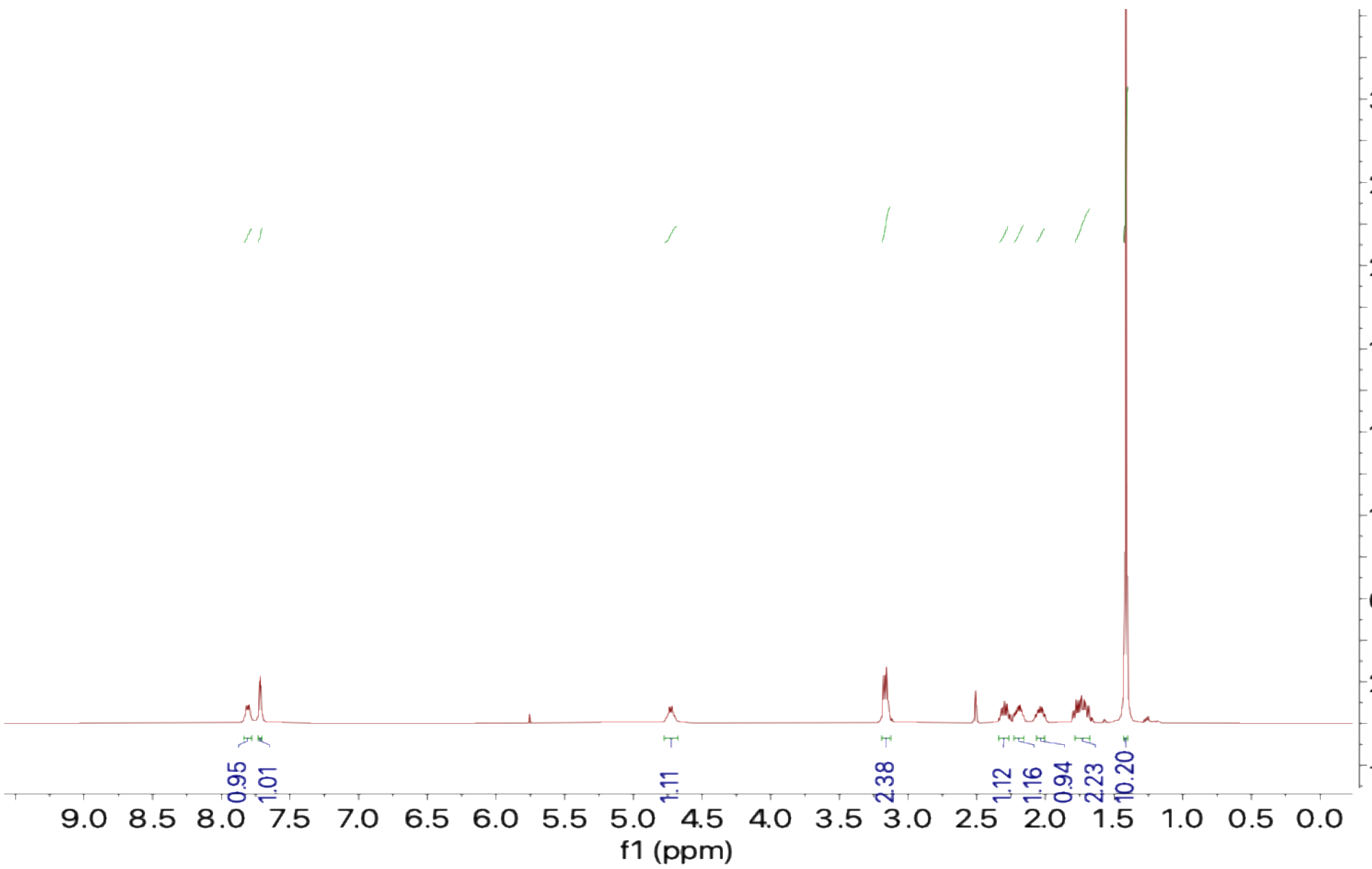
0.95
1.01
1.11
2.38
1.12
1.16
0.94
2.23
10.20
9.0 8.5 8.0 7.5 7.0 6.5 6.0 5.5 5.0 4.5 4.0 3.5 3.0 2.5 2.0 1.5 1.0 0.5 0.0
f1 (ppm)

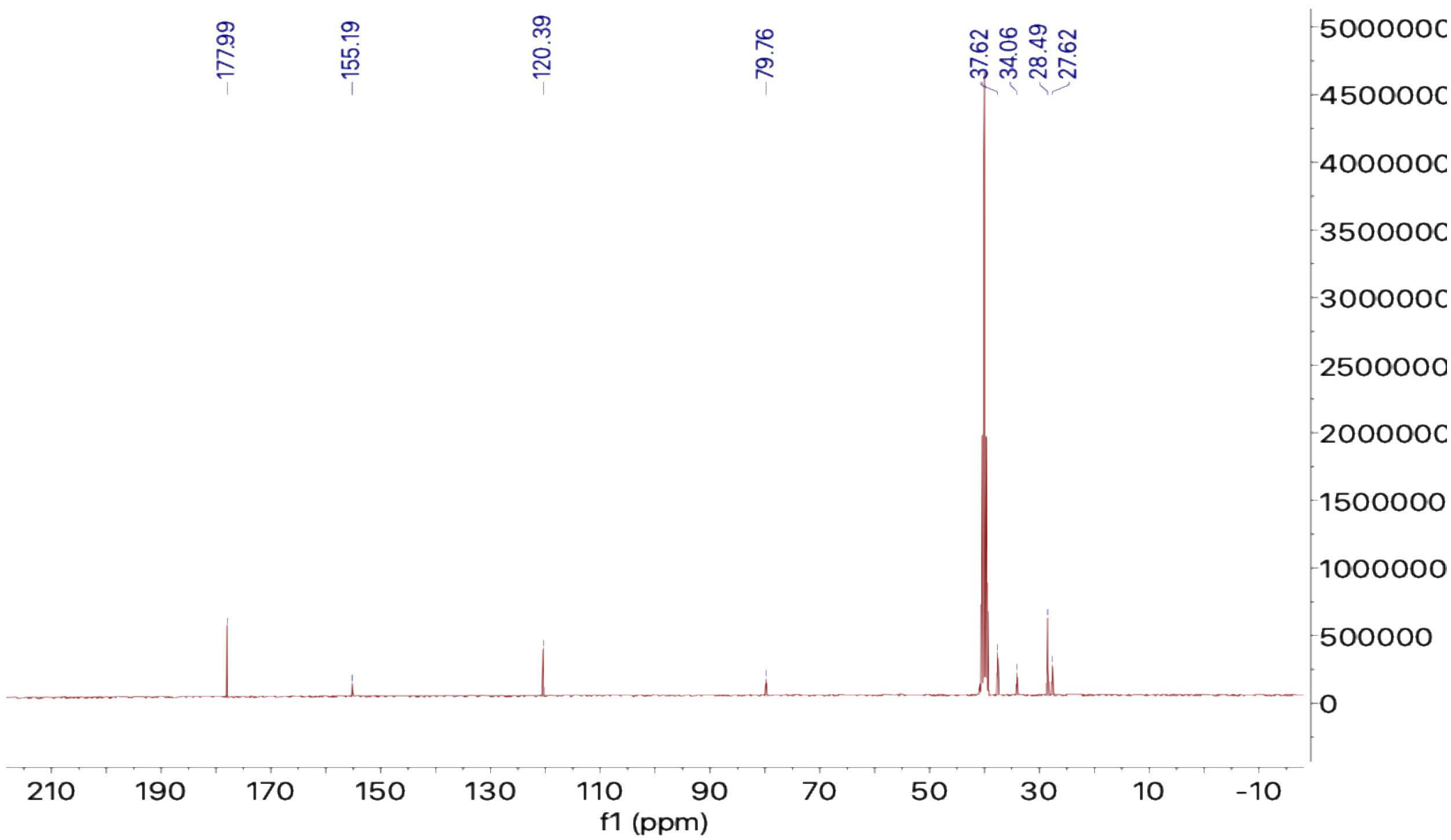
177.99
155.19
120.39
79.76
37.62
34.06
28.49
27.62
5000000
4500000
4000000
3500000
3000000
2500000
2000000
1500000
1000000
500000
0
210 190 170 150 130 110 90 70 50 30 10 -10
f1 (ppm)

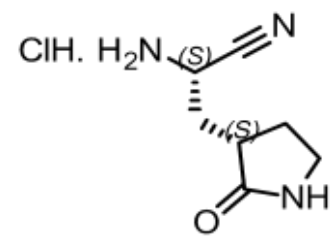
ClH. H2N
(S)
N
(S)
NH
O

Int 4

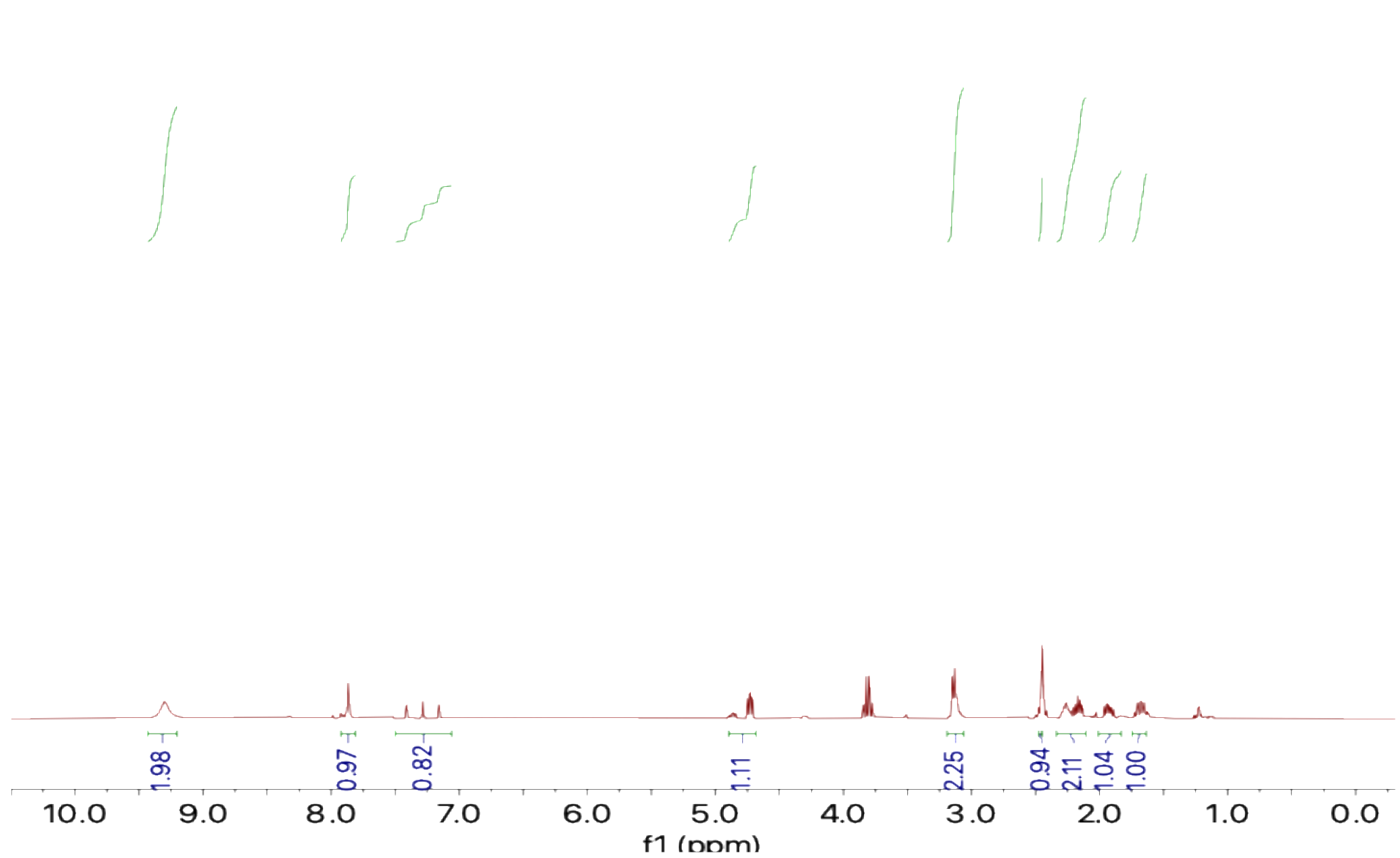
1.98
0.97
0.82
1.11
2.25
0.94
2.11
1.04
1.00
10.0
9.0
8.0
7.0
6.0
5.0
4.0
3.0
2.0
1.0
0.0
f1 (ppm)

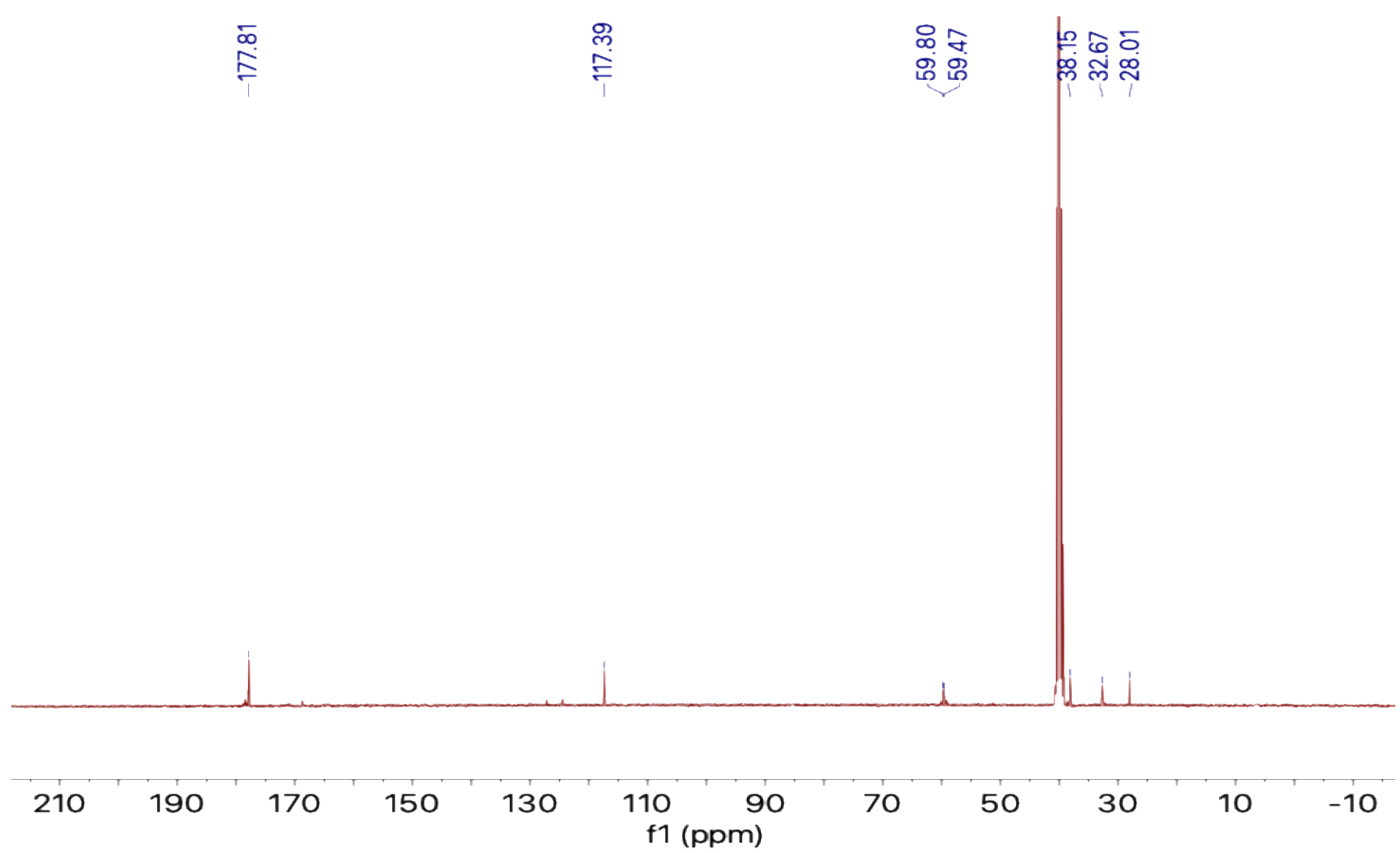
177.81
117.39
59.80
59.47
38.15
32.67
28.01
210
190
170
150
130
110
90
70
50
30
10
-10
f1 (ppm)

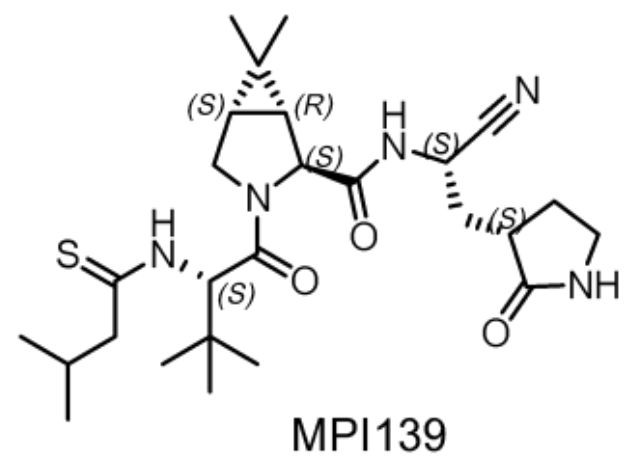
(S)
(R)
(S)
(S)
(S)
(S)
N
H
N
O
O
S
N
H
O
NH
N
O

MPI139

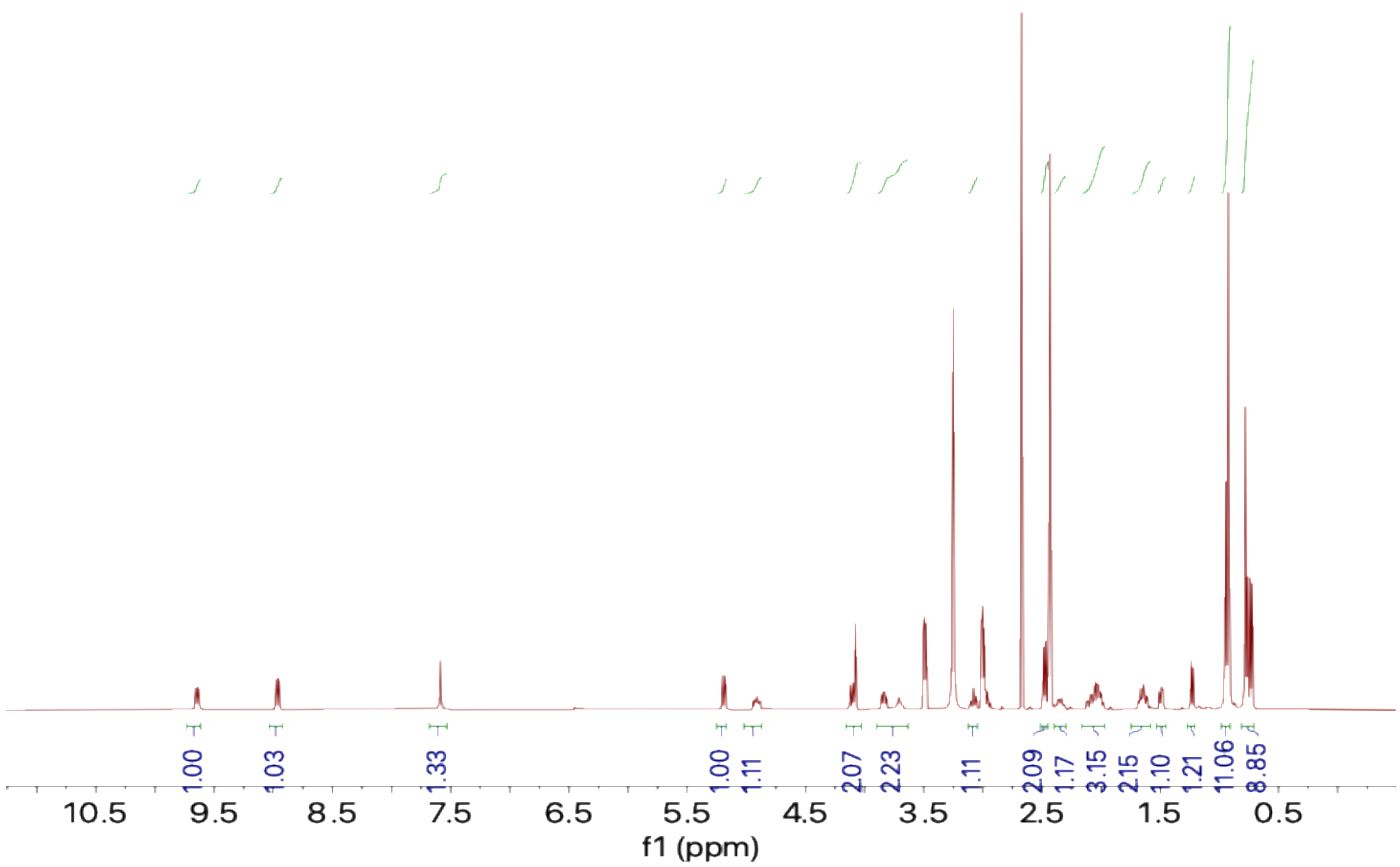
1.00
1.03
1.33
1.00
1.11
2.07
2.23
1.11
2.09
1.17
3.15
2.15
1.10
1.21
11.06
8.85
10.5
9.5
8.5
7.5
6.5
5.5
4.5
3.5
2.5
1.5
0.5
f1 (ppm)

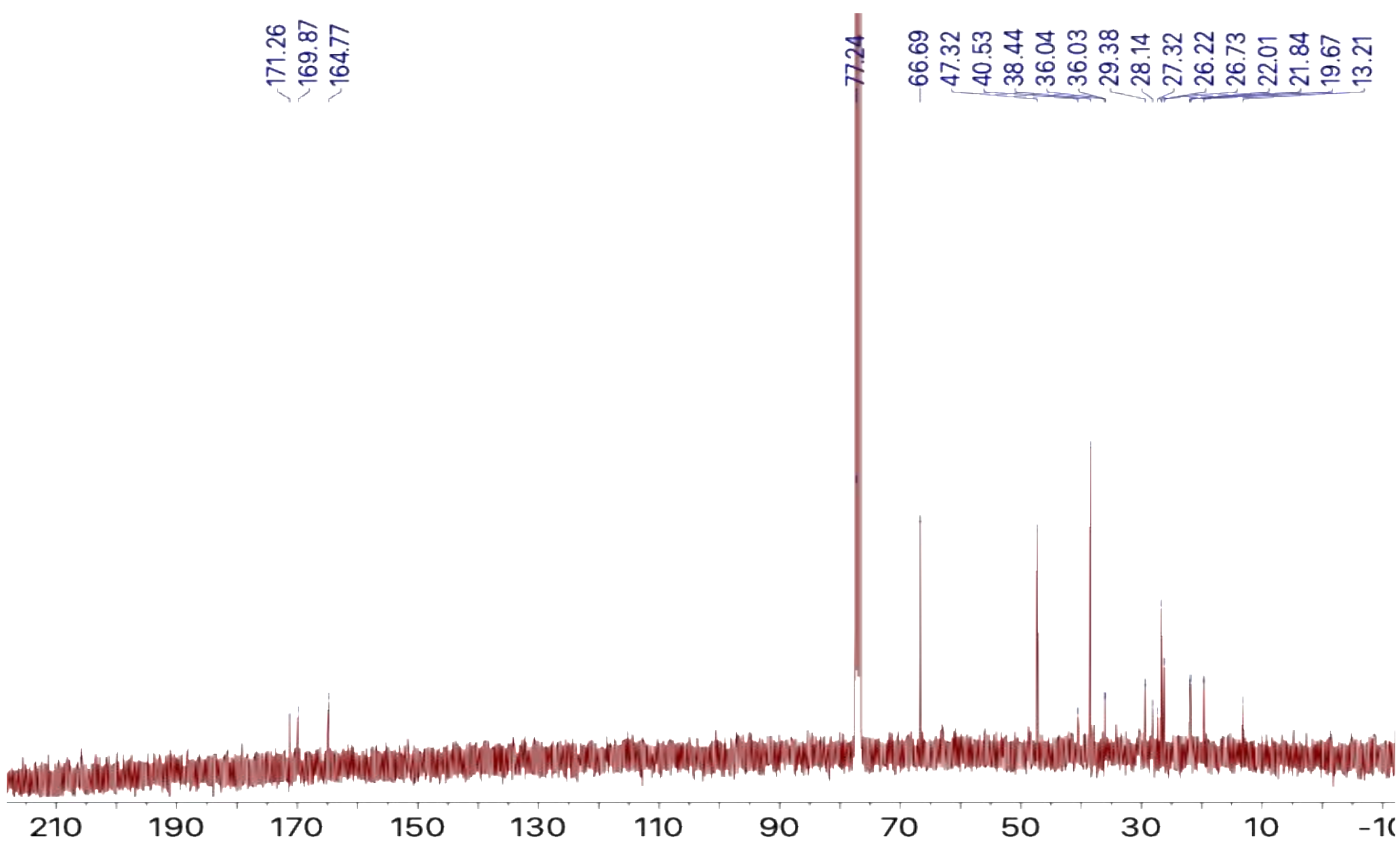
171.26
169.87
164.77
77.24
66.69
47.32
40.53
38.44
36.04
36.03
29.38
28.14
27.32
26.22
26.73
22.01
21.84
19.67
13.21
210
190
170
150
130
110
90
70
50
30
10

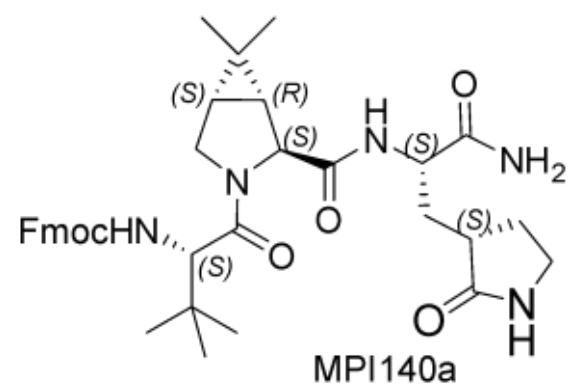
FmocHN
NH2
MPI140a

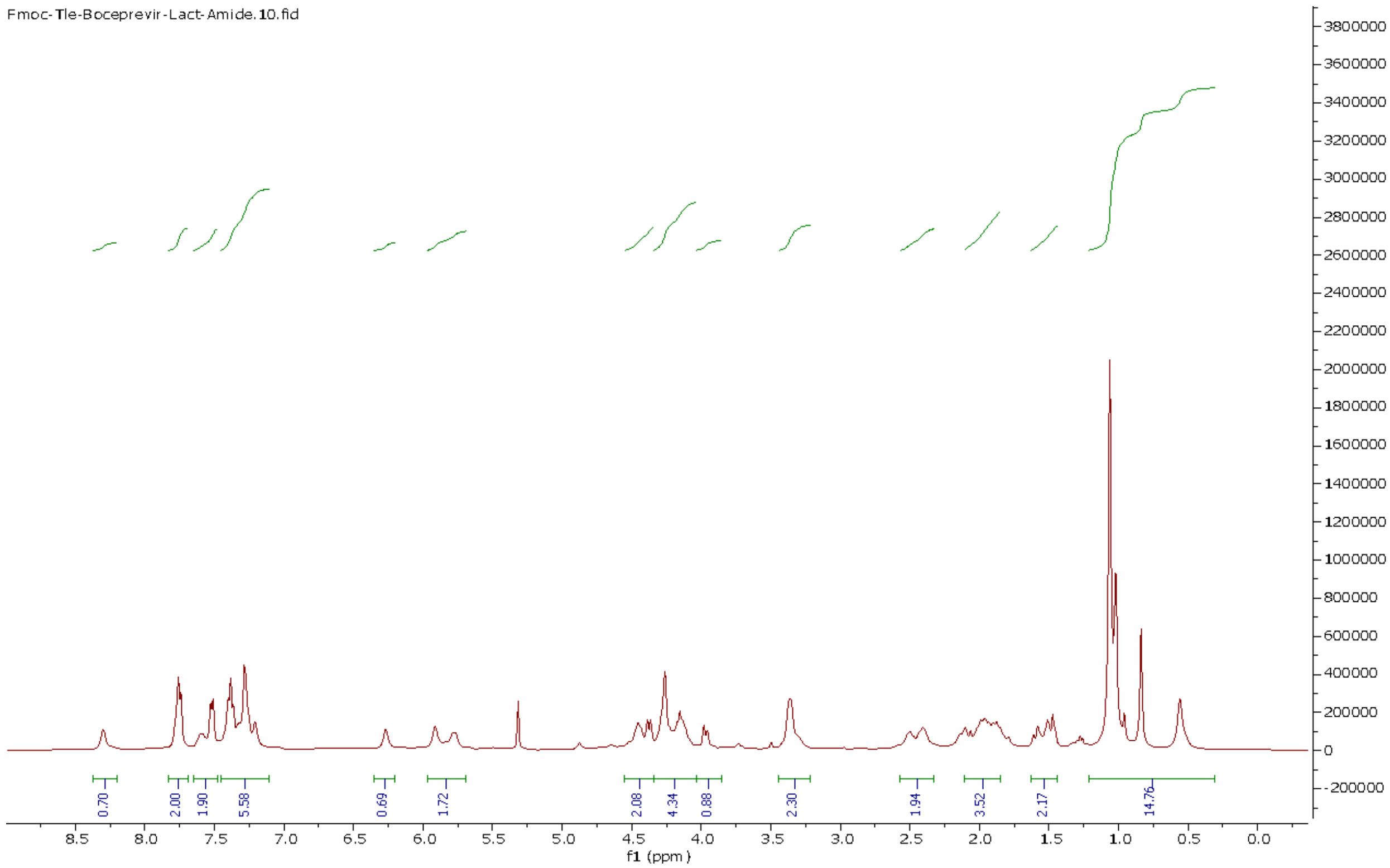
Fmoc-Tle-Boceprevir-Lact-Amide.10.fid
f1 (ppm)

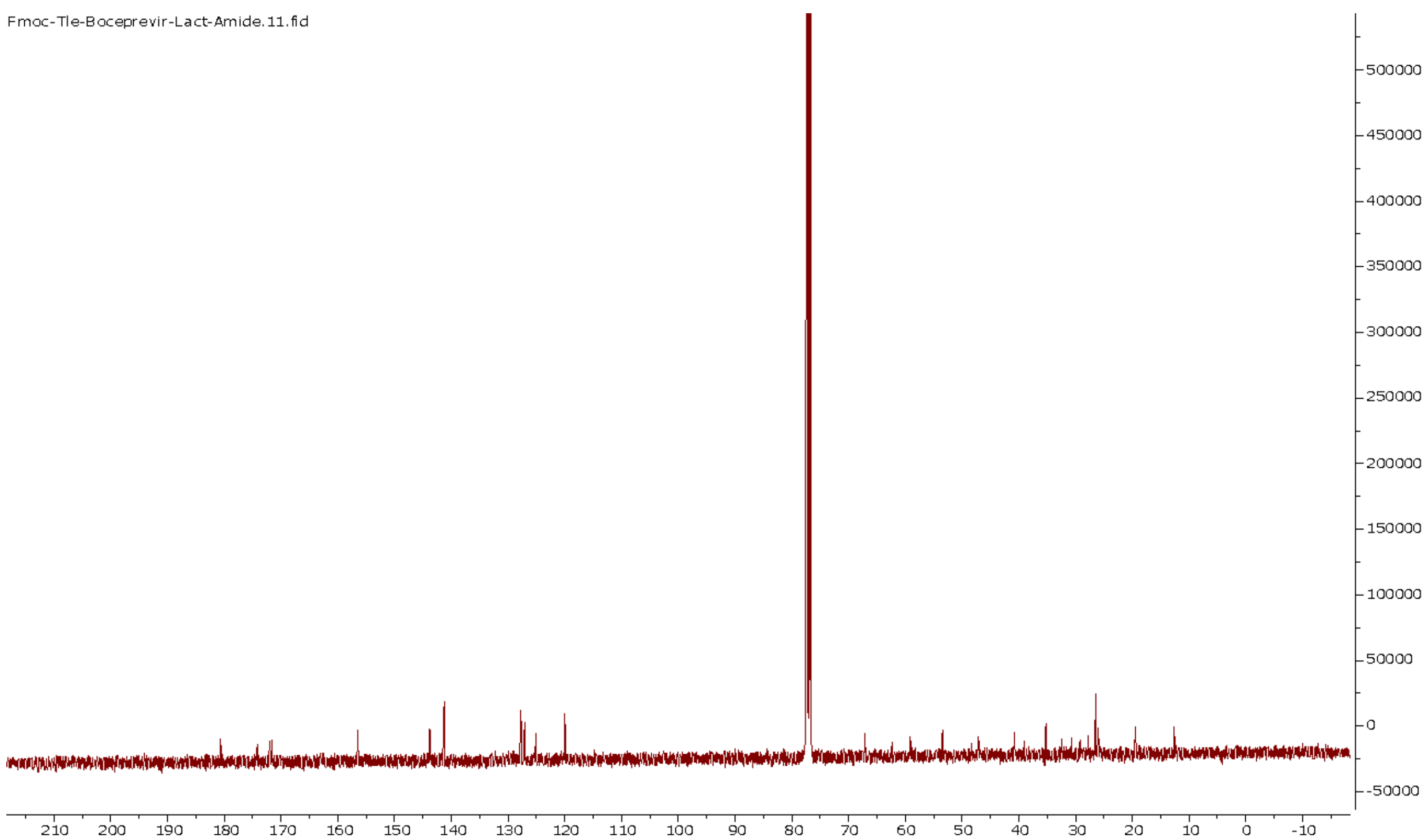
Fmoc-Tle-Boceprevir-Lact-Amide.11.fid
f1 (ppm)

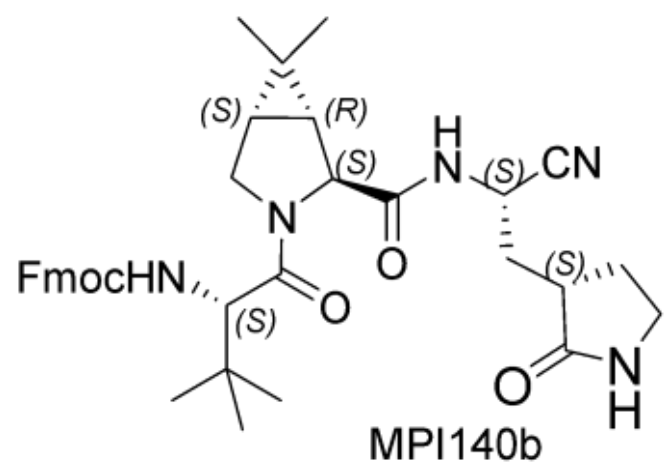
FmocHN
CN
MPI140b

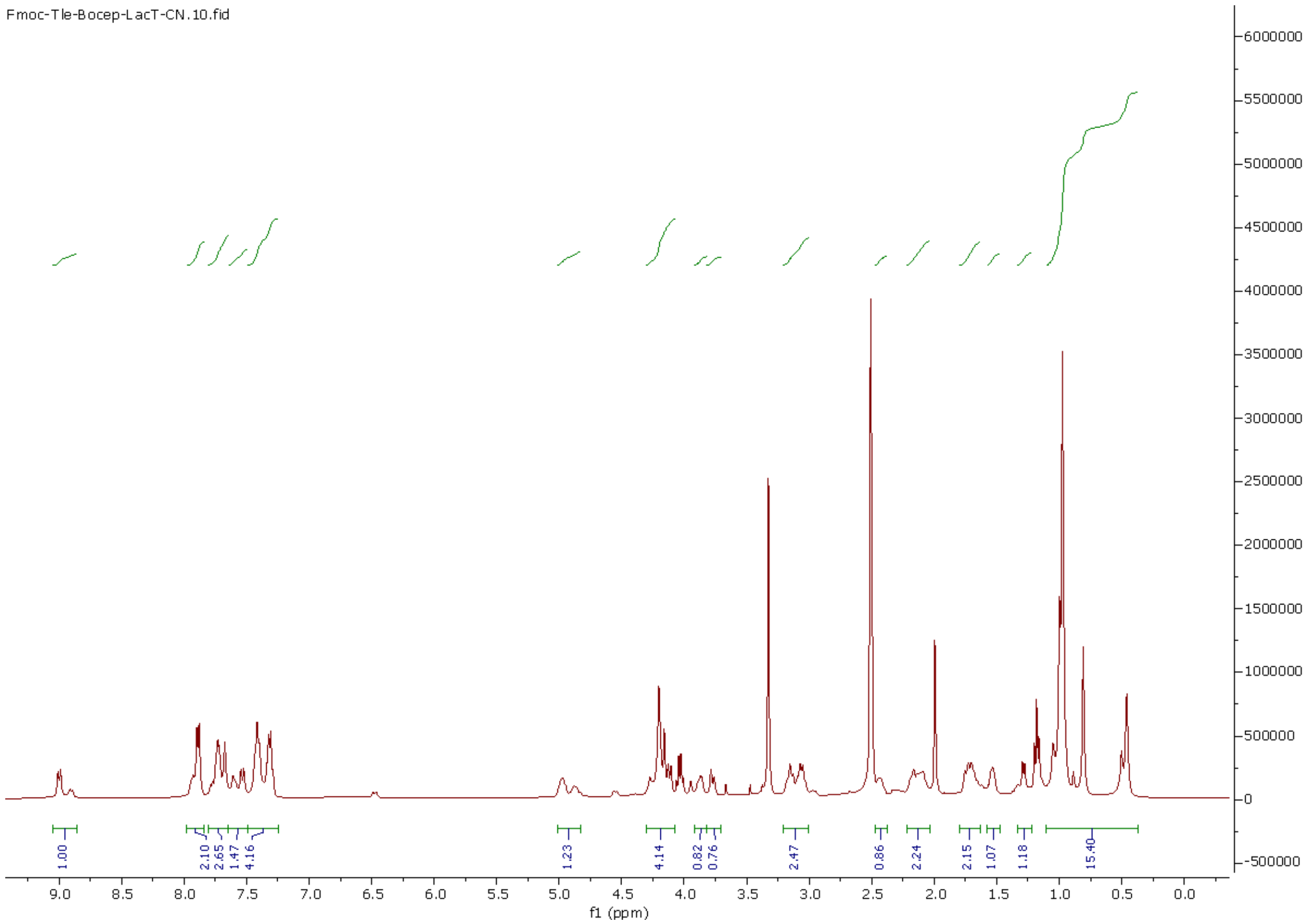
Fmoc-Tle-Bocep-LacT-CN.10.fid
1.00
2.10
2.65
1.47
4.16
1.23
4.14
0.82
0.76
2.47
0.86
2.24
2.15
1.07
1.18
15.40
f1 (ppm)

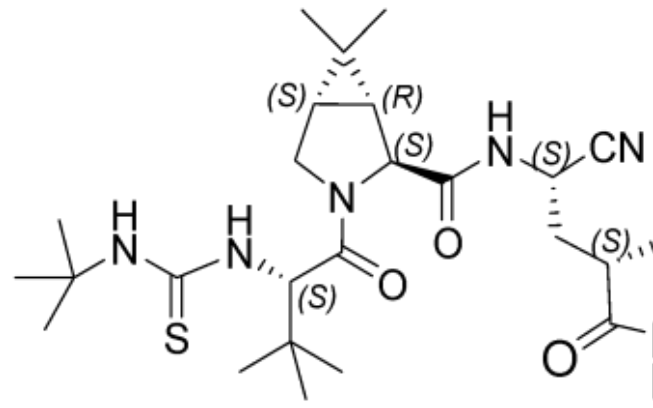

MPI140

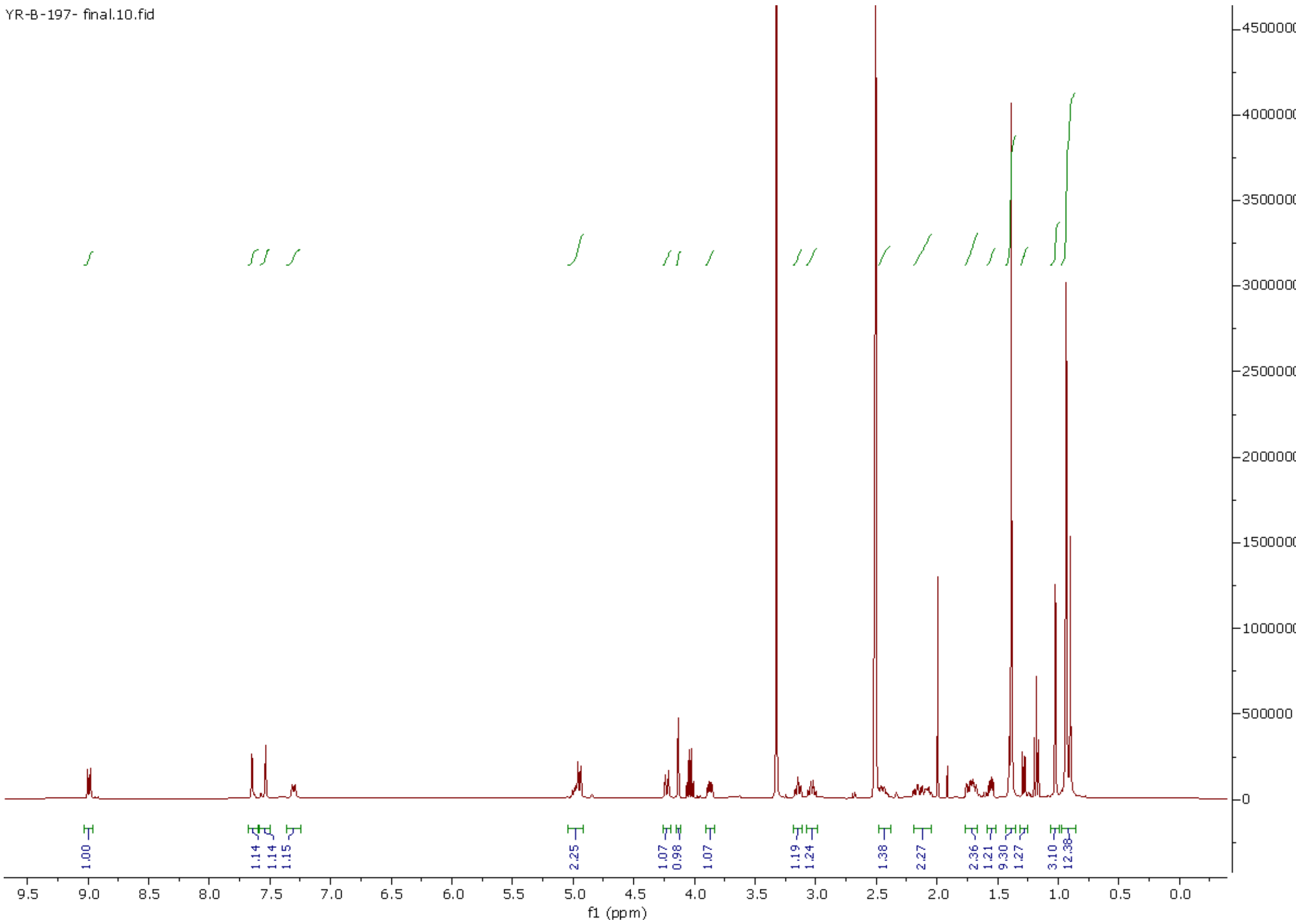

YR-B-197- final.10.fid
1.00
1.14
1.14
1.15
2.25
1.07
0.98
1.07
1.19
1.24
1.38
2.27
2.36
1.21
9.30
1.27
3.10
12.38
f1 (ppm)

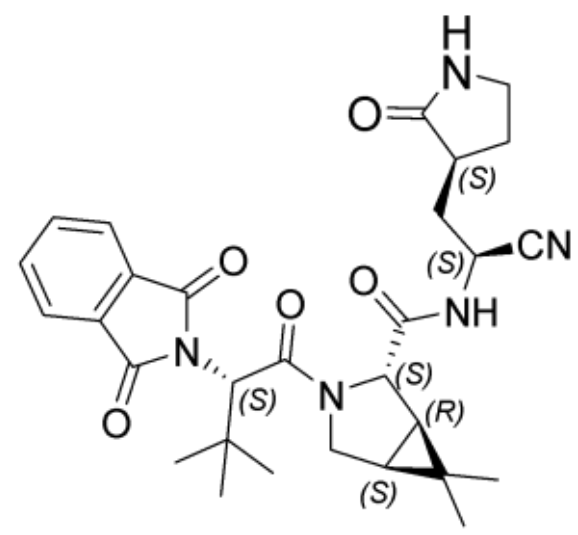

MPI141

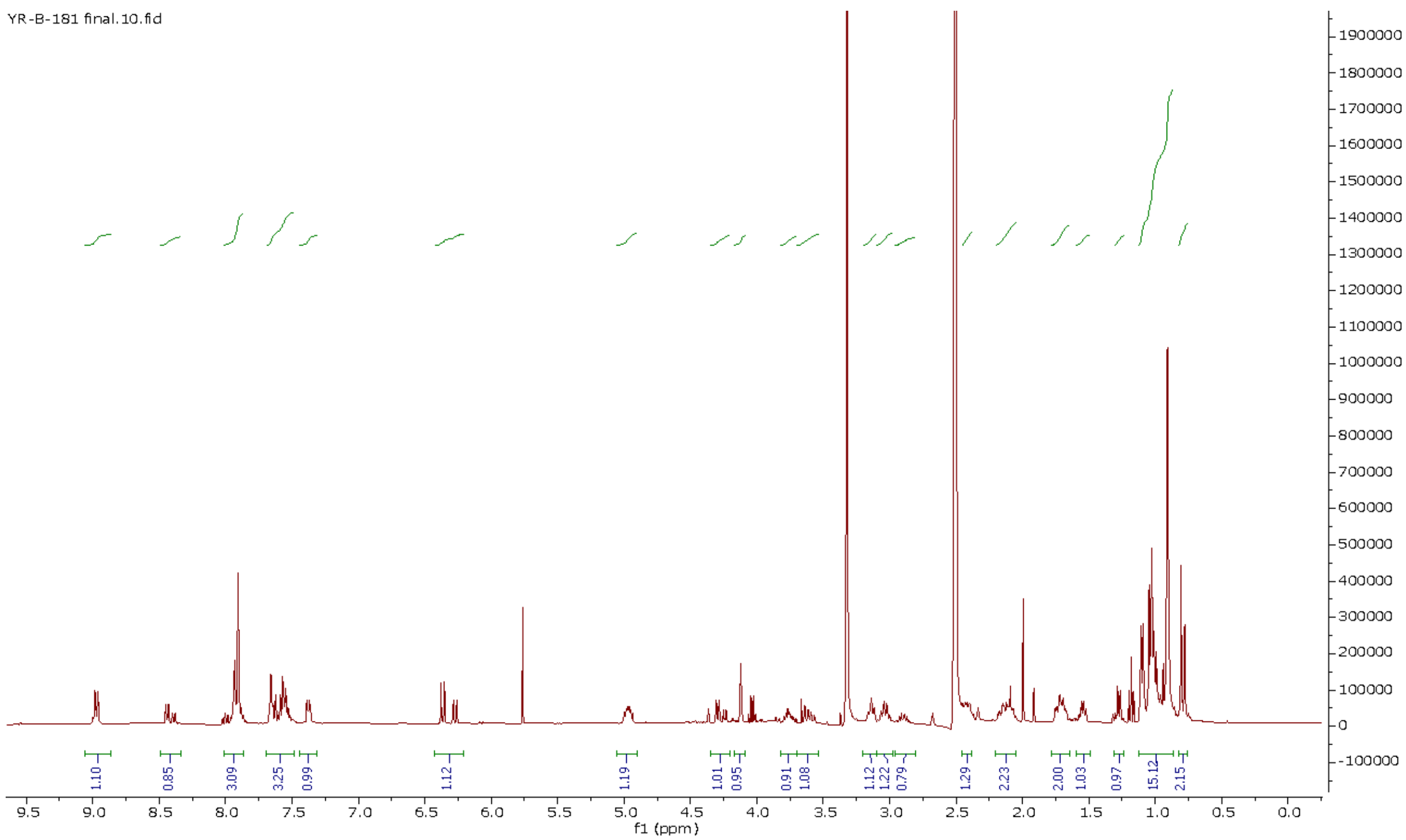

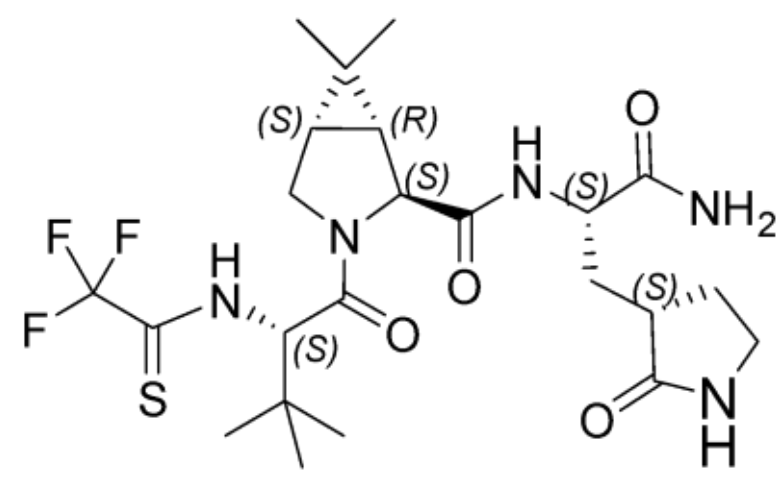

MPI142a

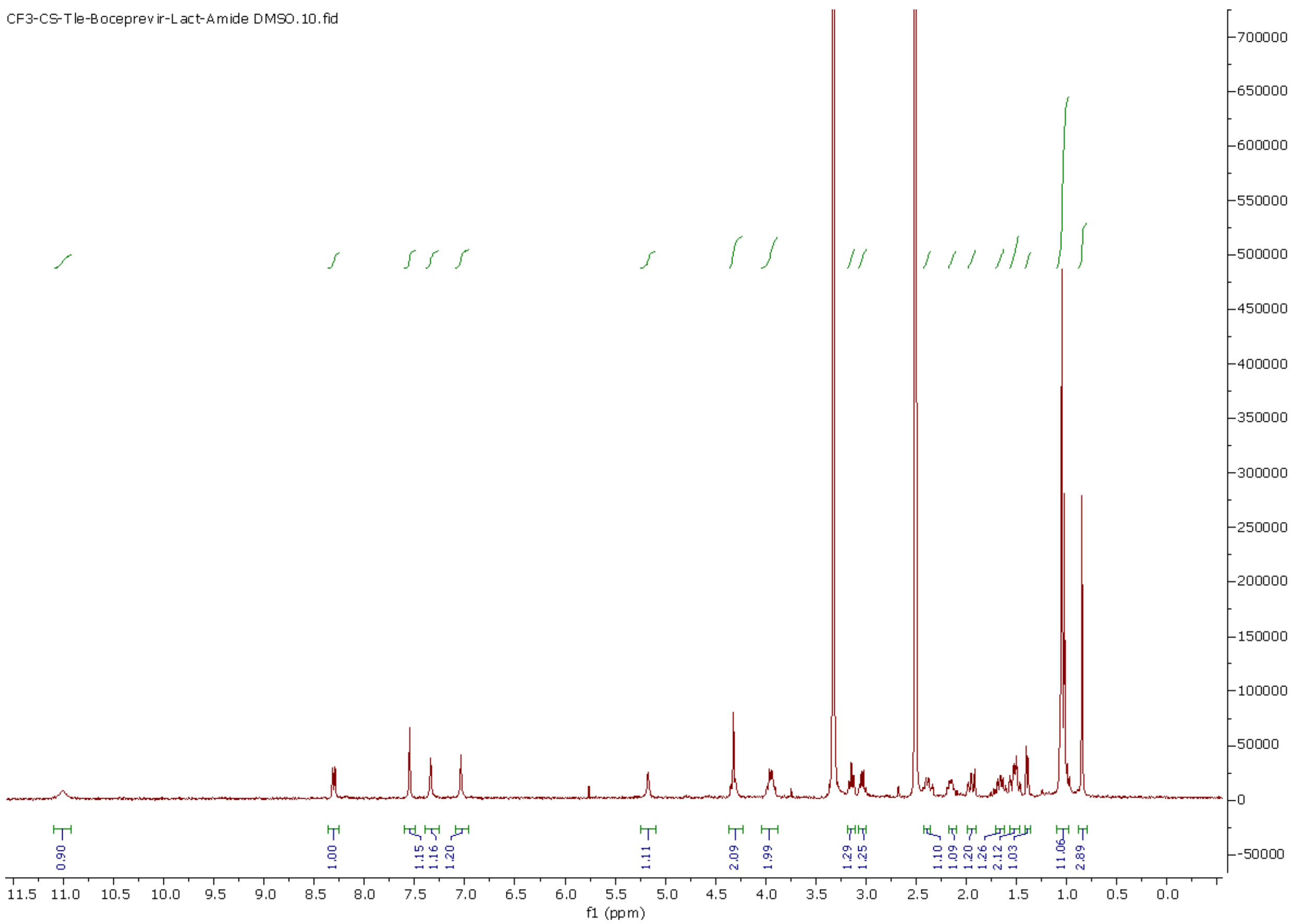

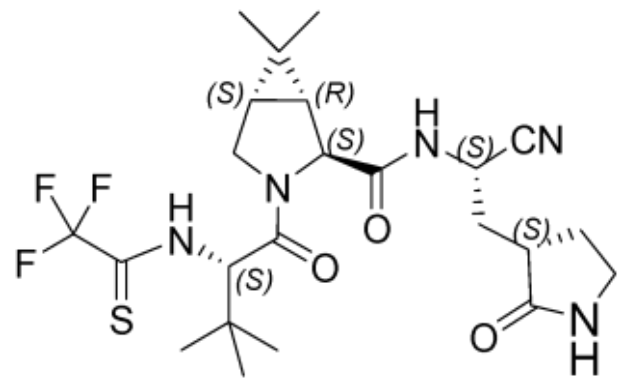
F
F
F
H
N
S
(S)
O
(S)
(R)
(S)
N
H
N
(S)
CN
O
(S)
O
N
H

MPI142

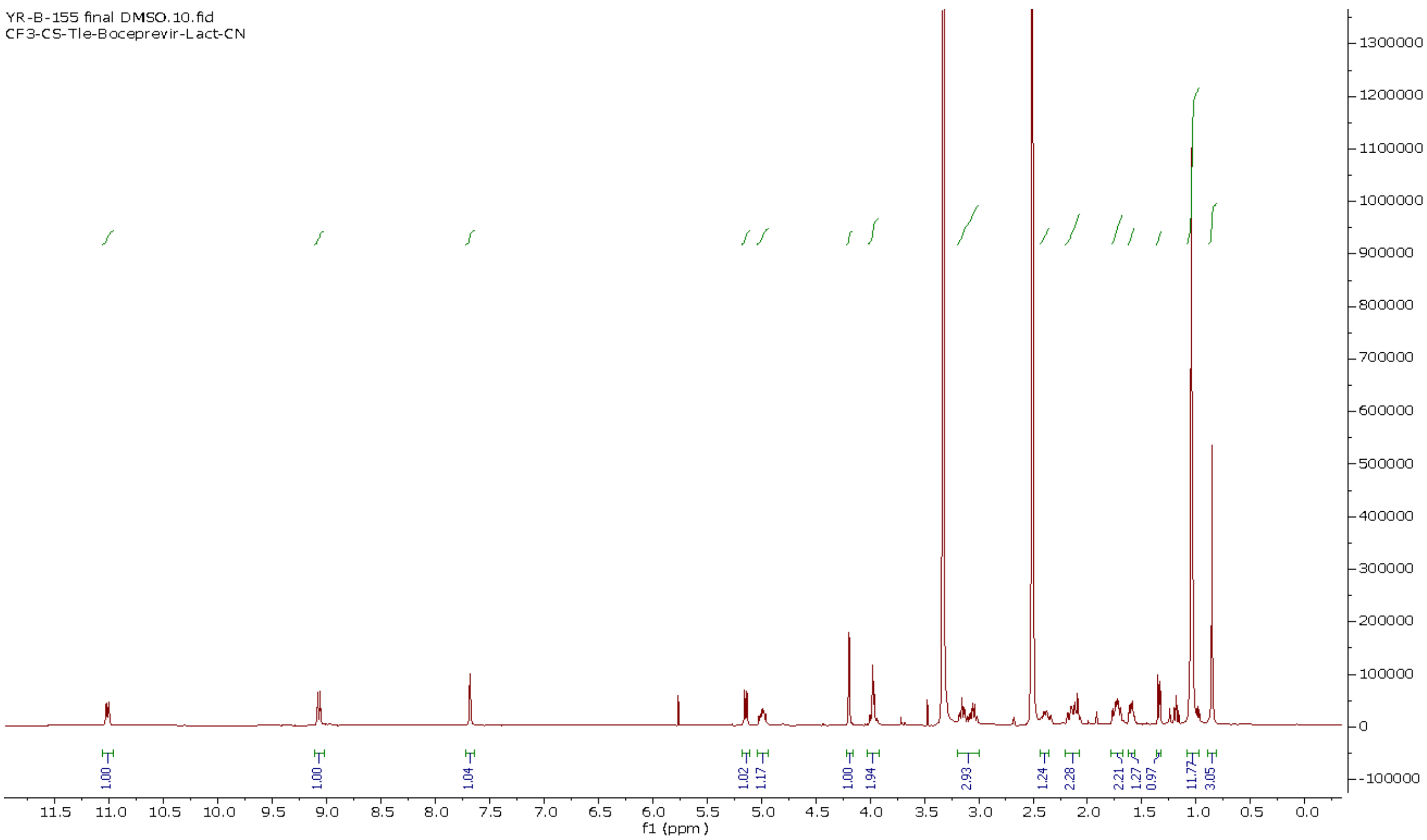
YR-B-155 final DMSO.10.fid
CF3-CS-Tle-Boceprevir-Lact-CN
1.00
1.00
1.04
1.02
1.17
1.00
1.94
2.93
1.24
2.28
2.21
1.27
0.97
11.72
3.05
11.5 11.0 10.5 10.0 9.5 9.0 8.5 8.0 7.5 7.0 6.5 6.0 5.5 5.0 4.5 4.0 3.5 3.0 2.5 2.0 1.5 1.0 0.5 0.0
f1 (ppm)
1300000
1200000
1100000
1000000
900000
800000
700000
600000
500000
400000
300000
200000
100000
0
-100000

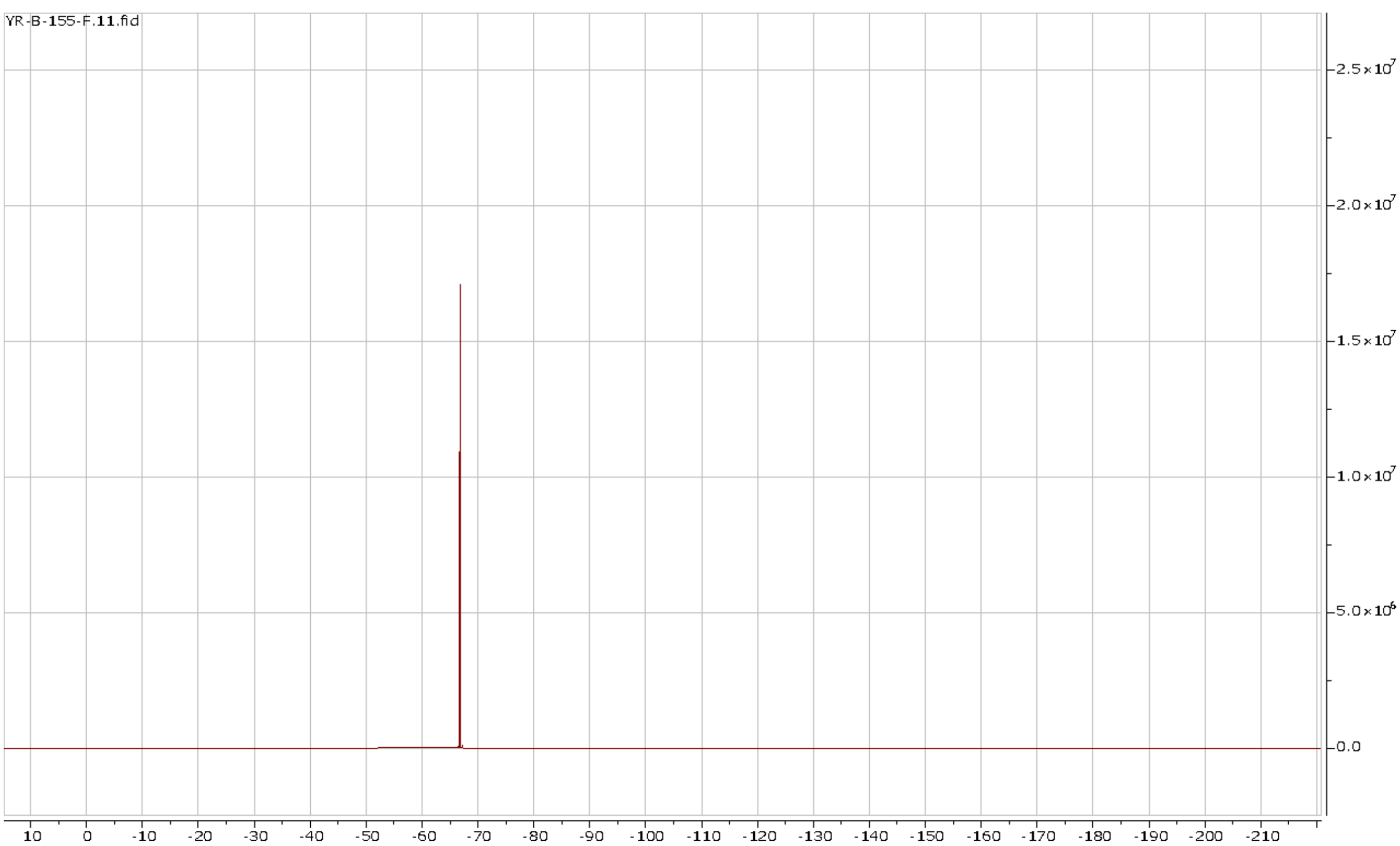
YR-B-155-F.11.fid
10 0 -10 -20 -30 -40 -50 -60 -70 -80 -90 -100 -110 -120 -130 -140 -150 -160 -170 -180 -190 -200 -210
f1 (ppm)
2.5×10^7
2.0×10^7
1.5×10^7
1.0×10^7
5.0×10^6
0.0

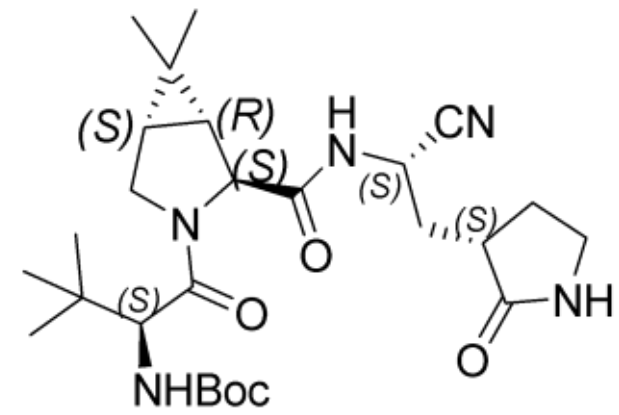

MPI143

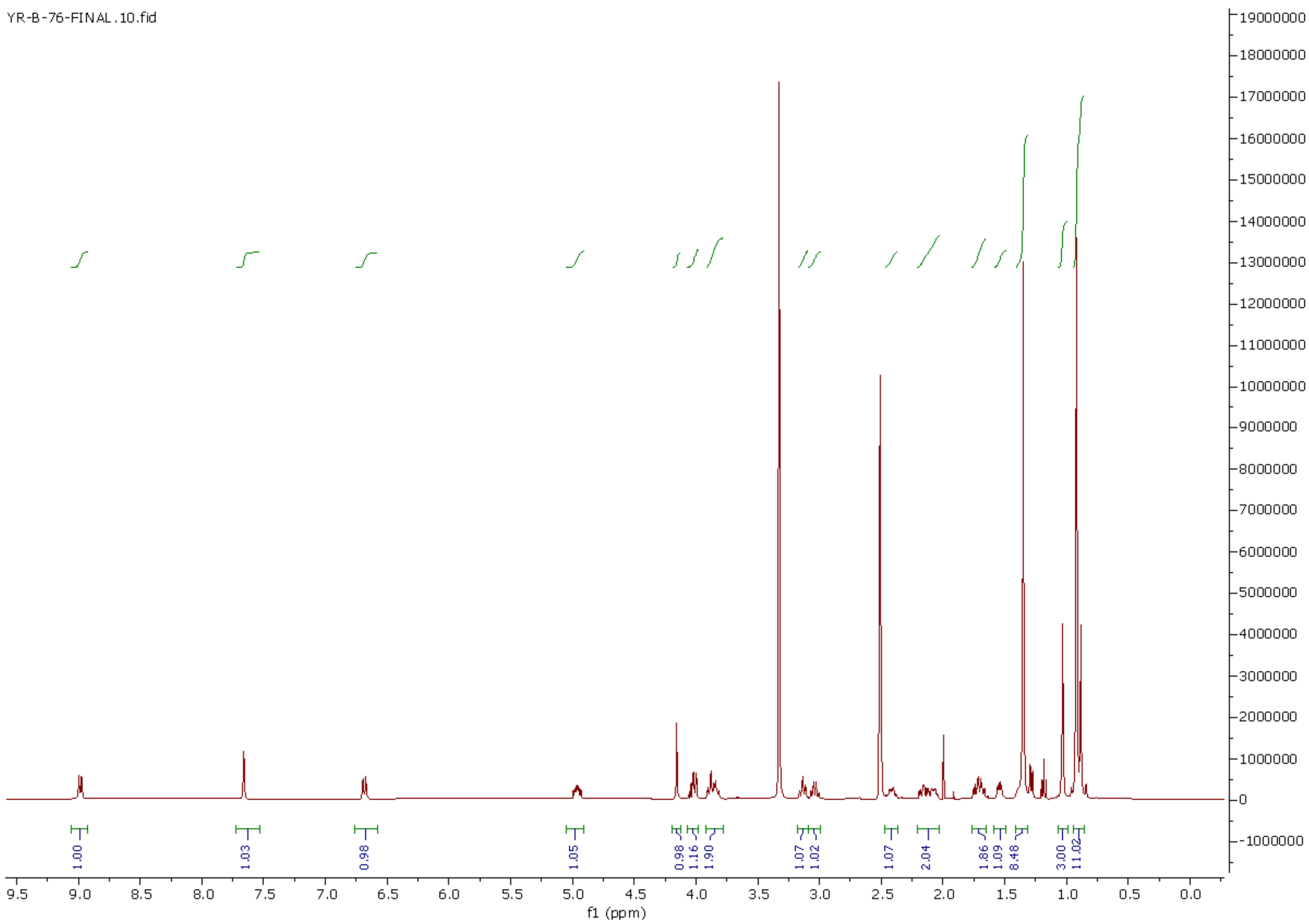

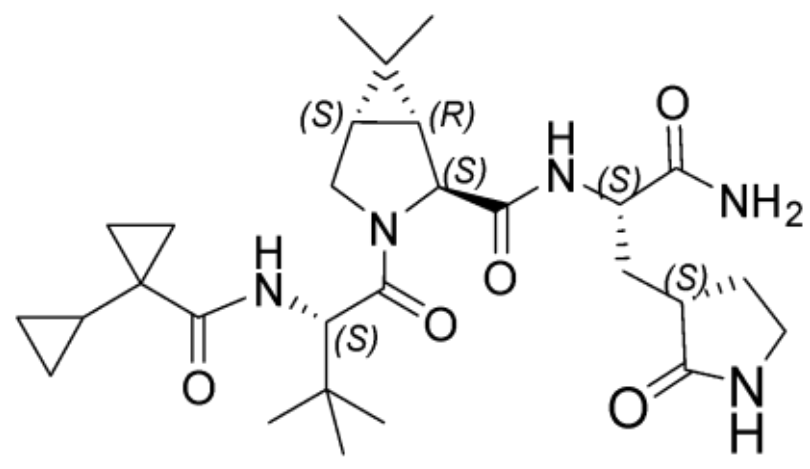

MPI144a

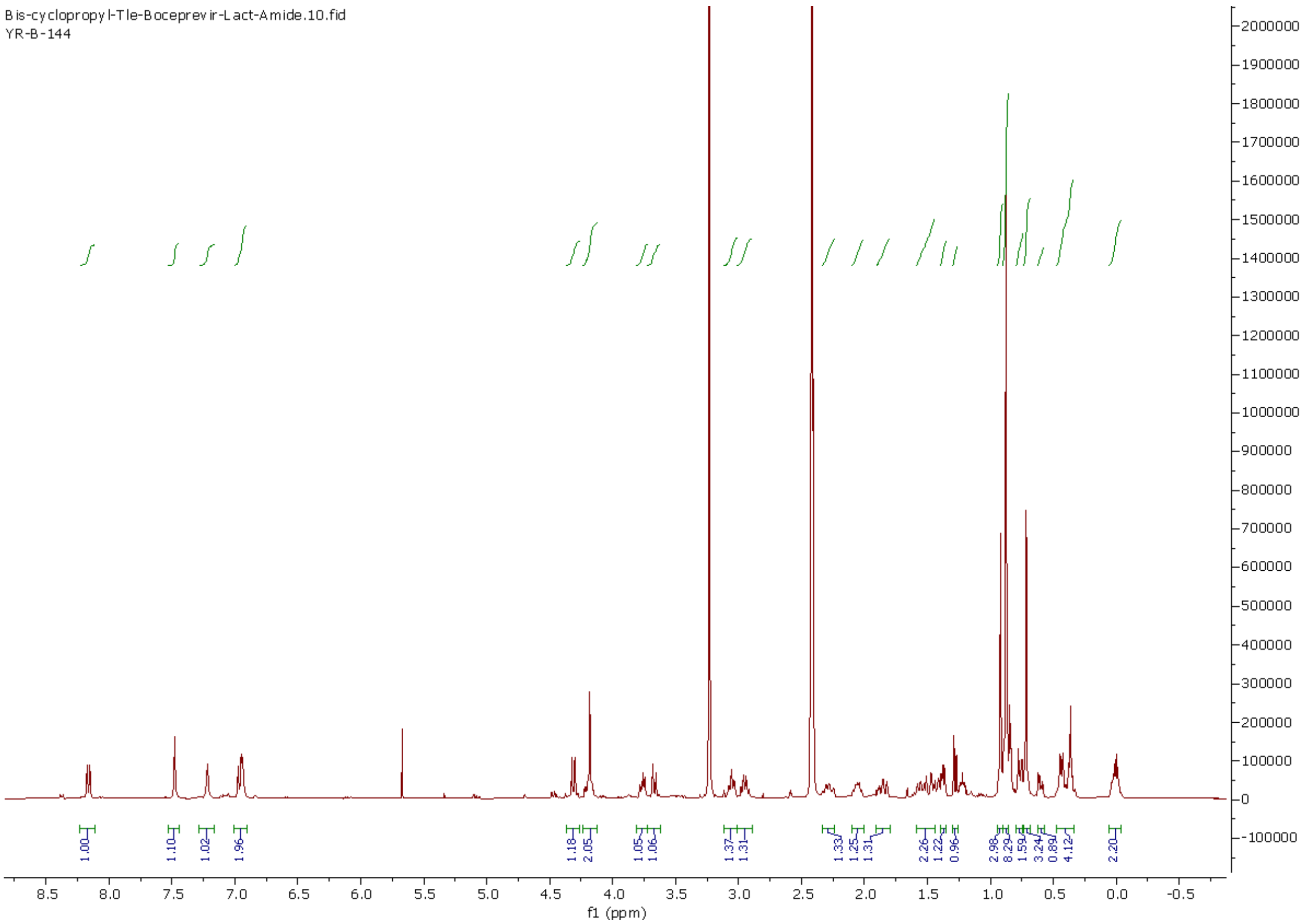
Bis-cyclopropyl-Tle-Boceprevir-Lact-Amide.10.fid
YR-B-144
f1 (ppm)

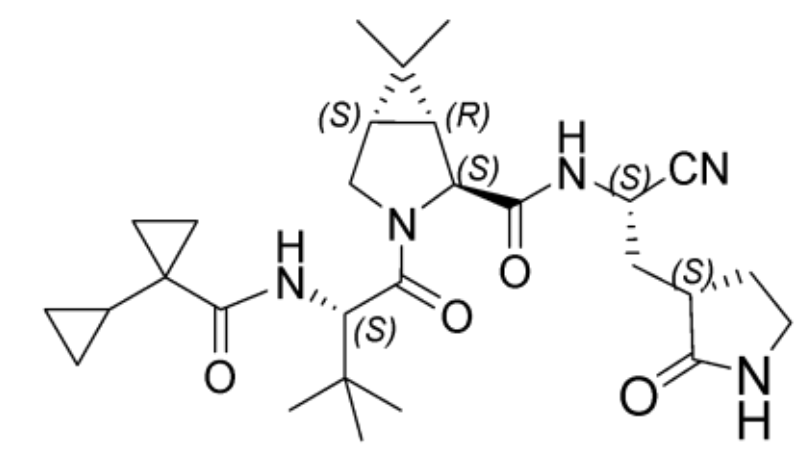

(S)
(R)
(S)
H
N
(S)
CN
H
N
N
(S)
O
O
(S)
O
O
N
H

MPI144

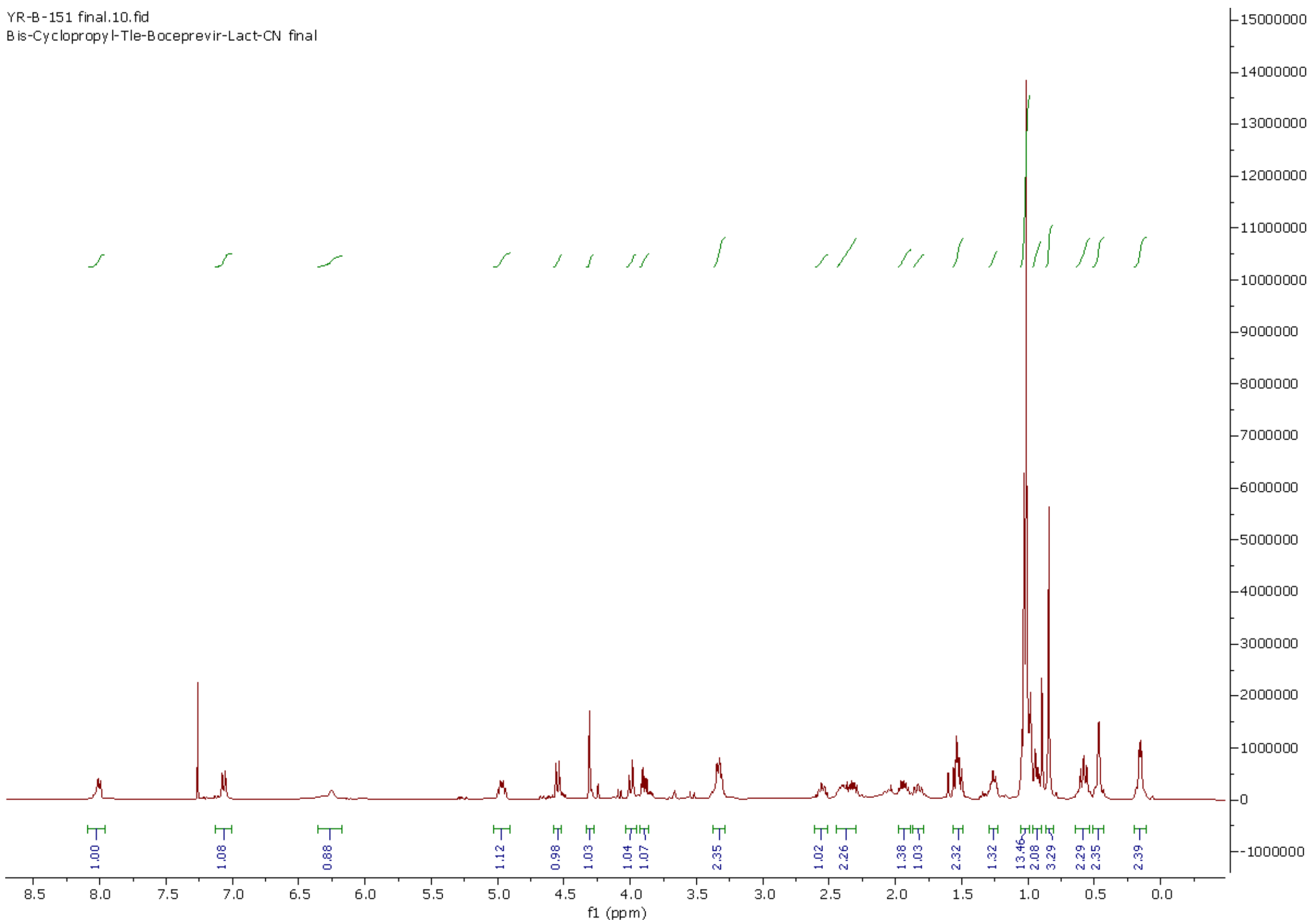

YR-B-151 final.10.fid
Bis-Cyclopropyl-Tle-Boceprevir-Lact-CN final
1.00
1.08
0.88
1.12
0.98
1.03
1.04
1.07
2.35
1.02
2.26
1.38
1.03
2.32
1.32
13.46
2.08
3.29
2.29
2.35
2.39
f1 (ppm)

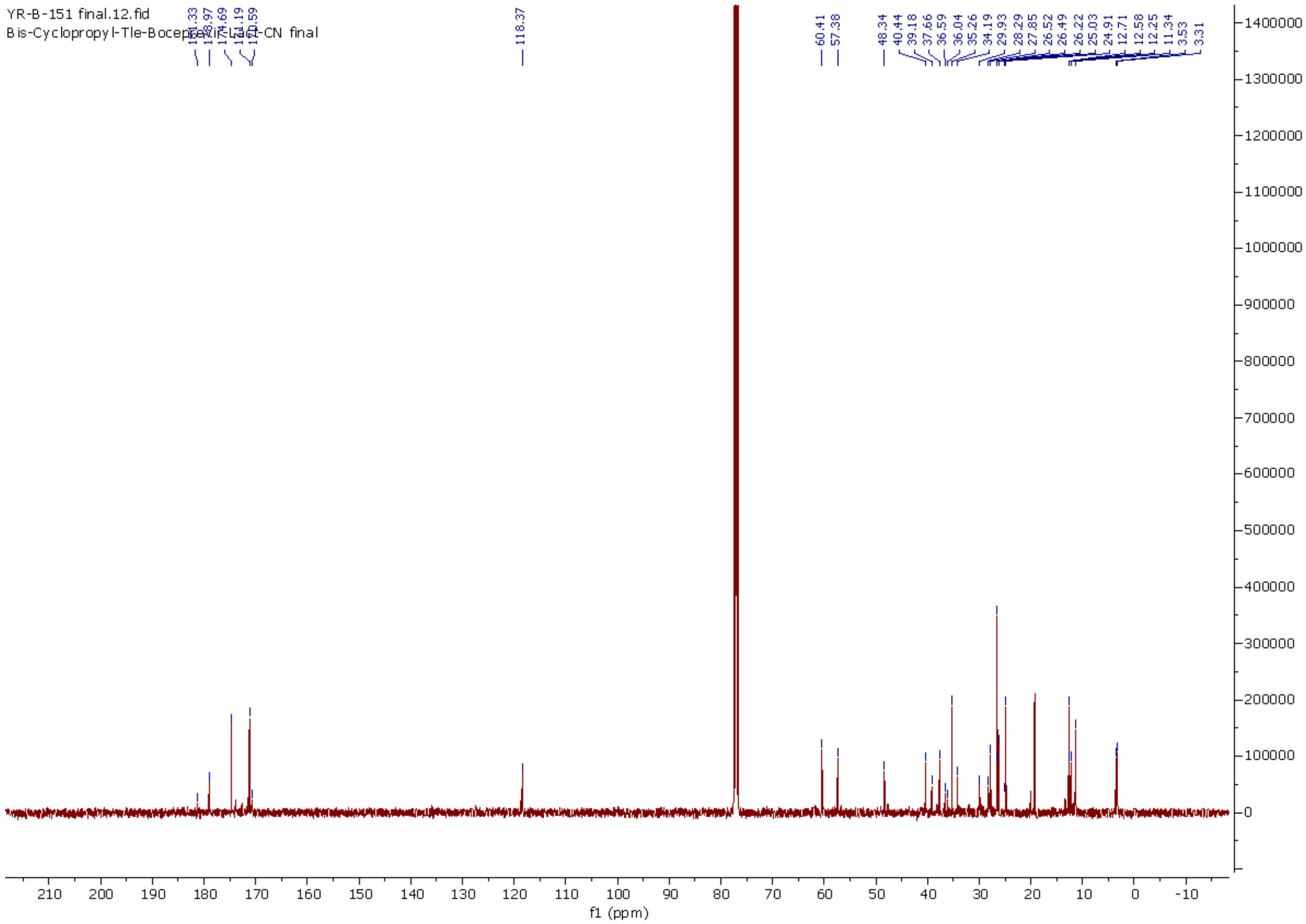

YR-B-151 final.12.fid
Bis-Cyclopropyl-Tle-Boceprevir-Lact-CN final
181.33
178.97
174.69
171.19
170.59
118.37
60.41
57.38
48.34
40.44
39.18
37.66
36.59
36.04
35.26
34.19
29.93
28.29
27.85
26.52
26.49
26.22
25.03
24.91
12.71
12.58
12.25
11.34
3.53
3.31
f1 (ppm)

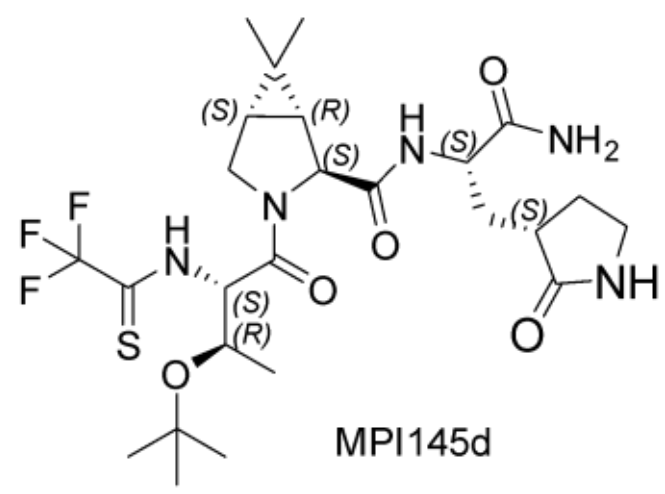

MPI145d

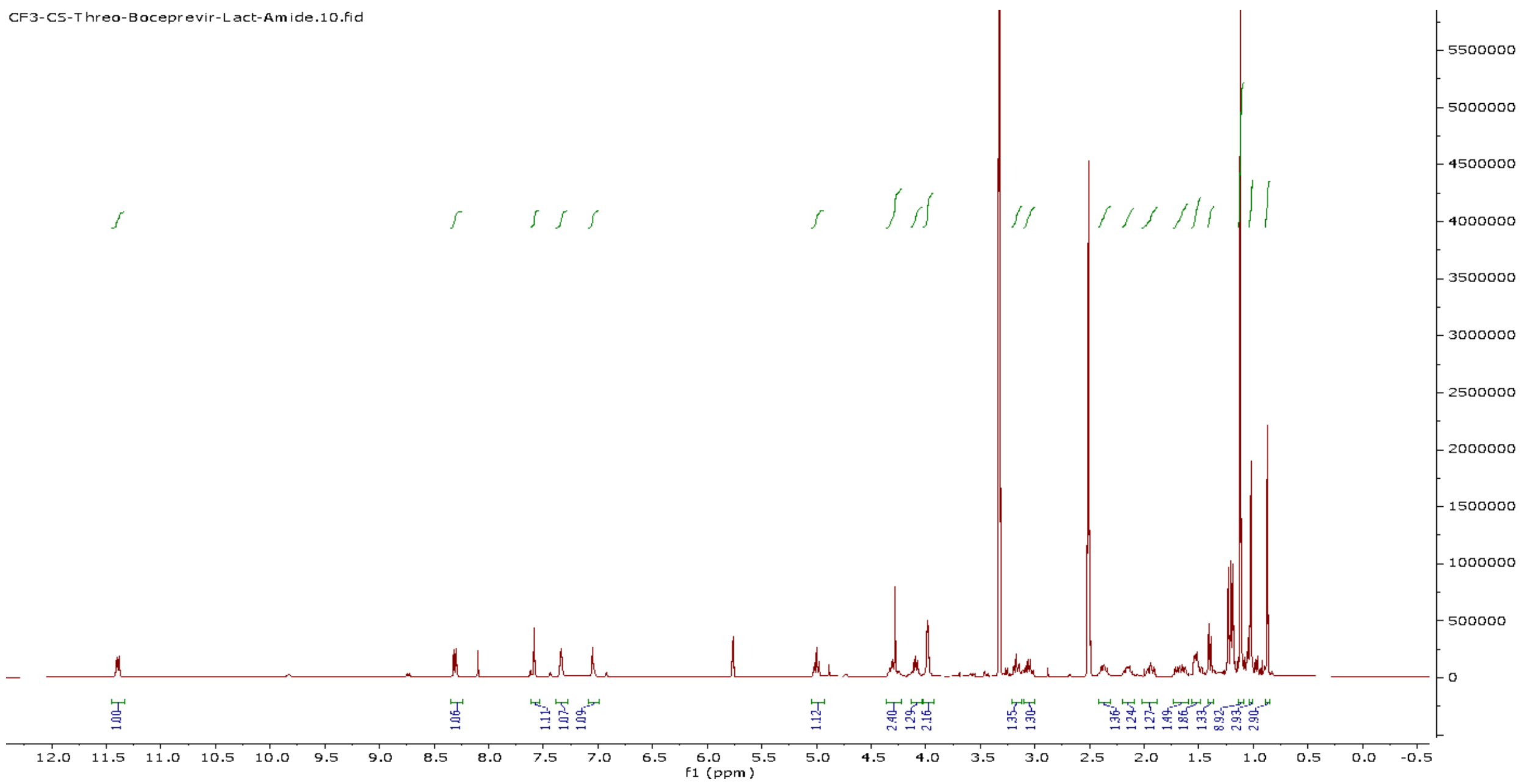

CF3-CS-Threo-Boceprevir-Lact-Amide.10.fid
f1 (ppm)

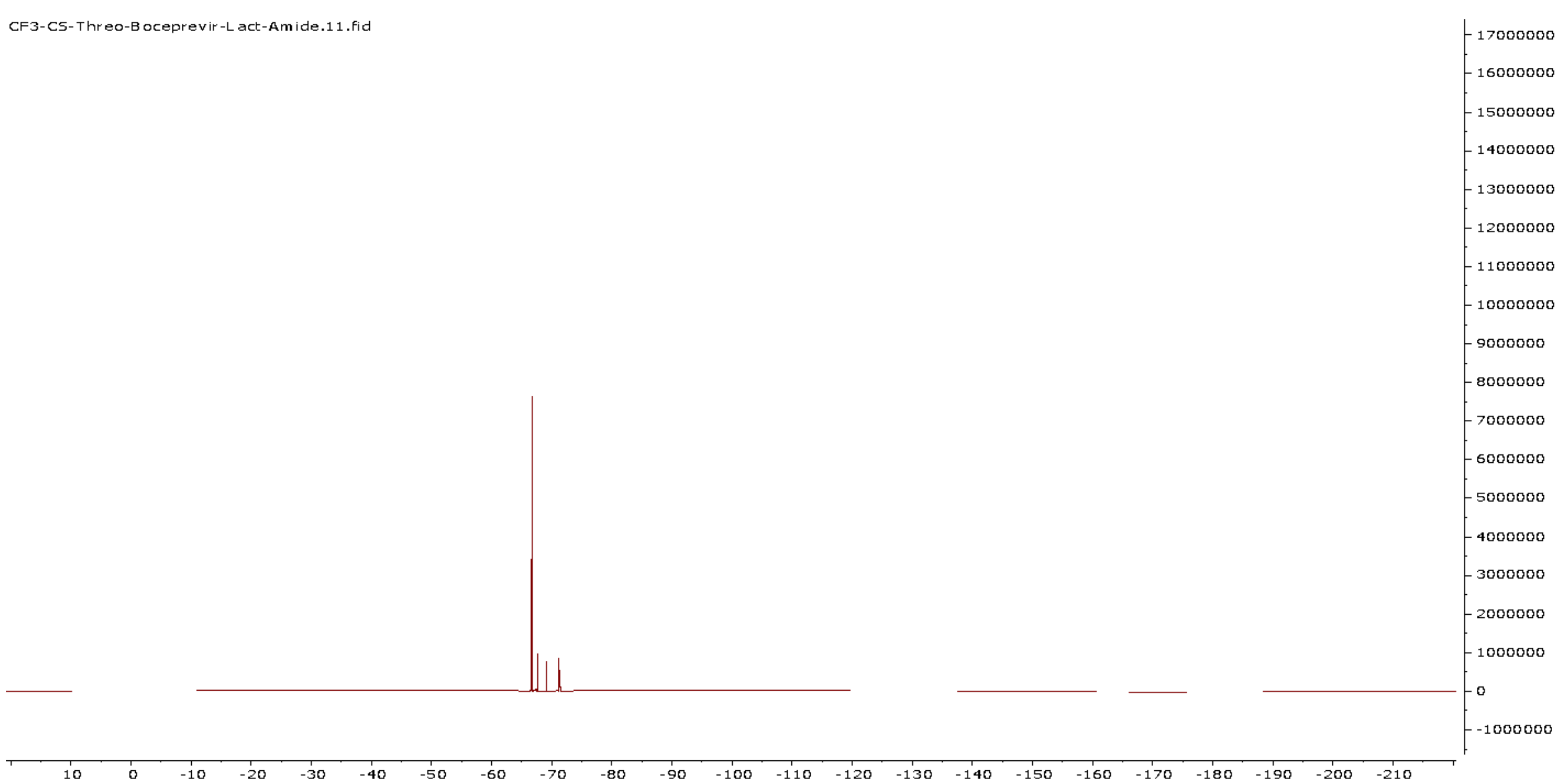

CF3-CS-Threo-Boceprevir-Lact-Amide.11.fid
f1 (ppm)

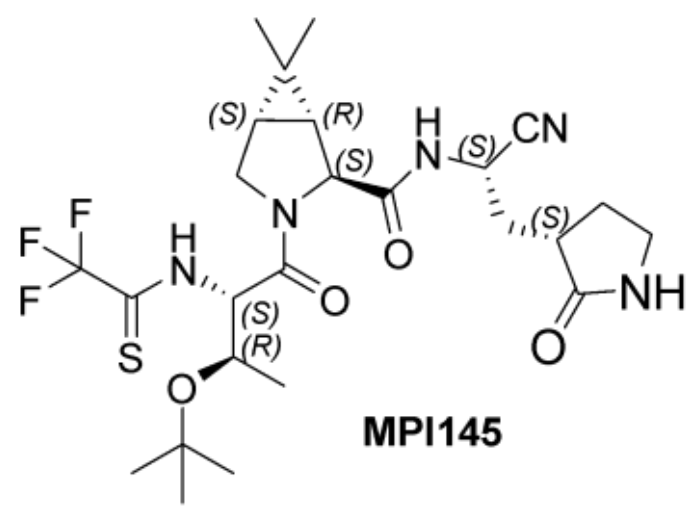

MPI145

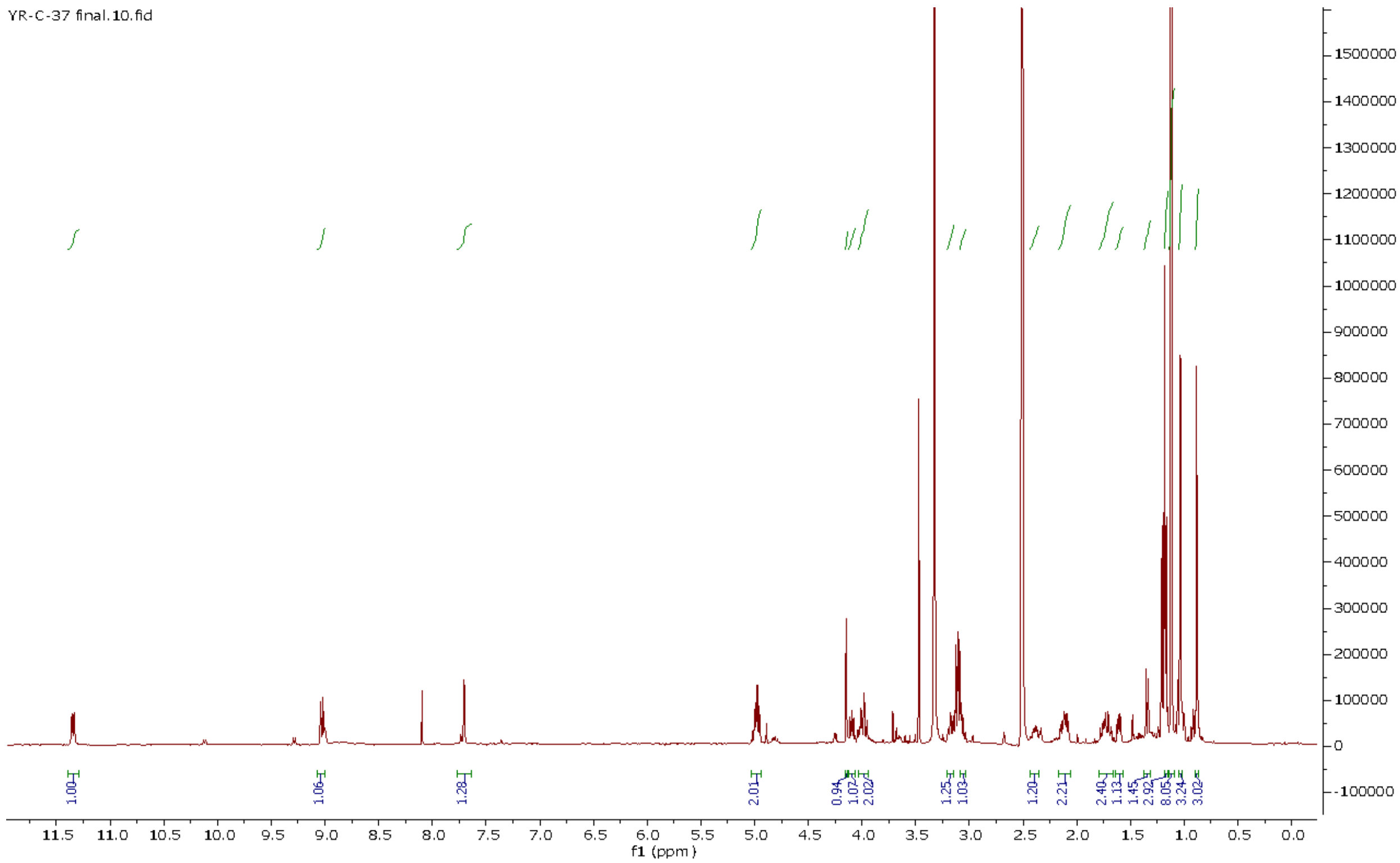

YR-C-37 final.10.fid
1.00
1.06
1.28
2.01
0.94
1.07
2.02
1.25
1.03
1.20
2.21
2.40
1.13
1.45
2.92
8.05
3.24
3.02
f1 (ppm)

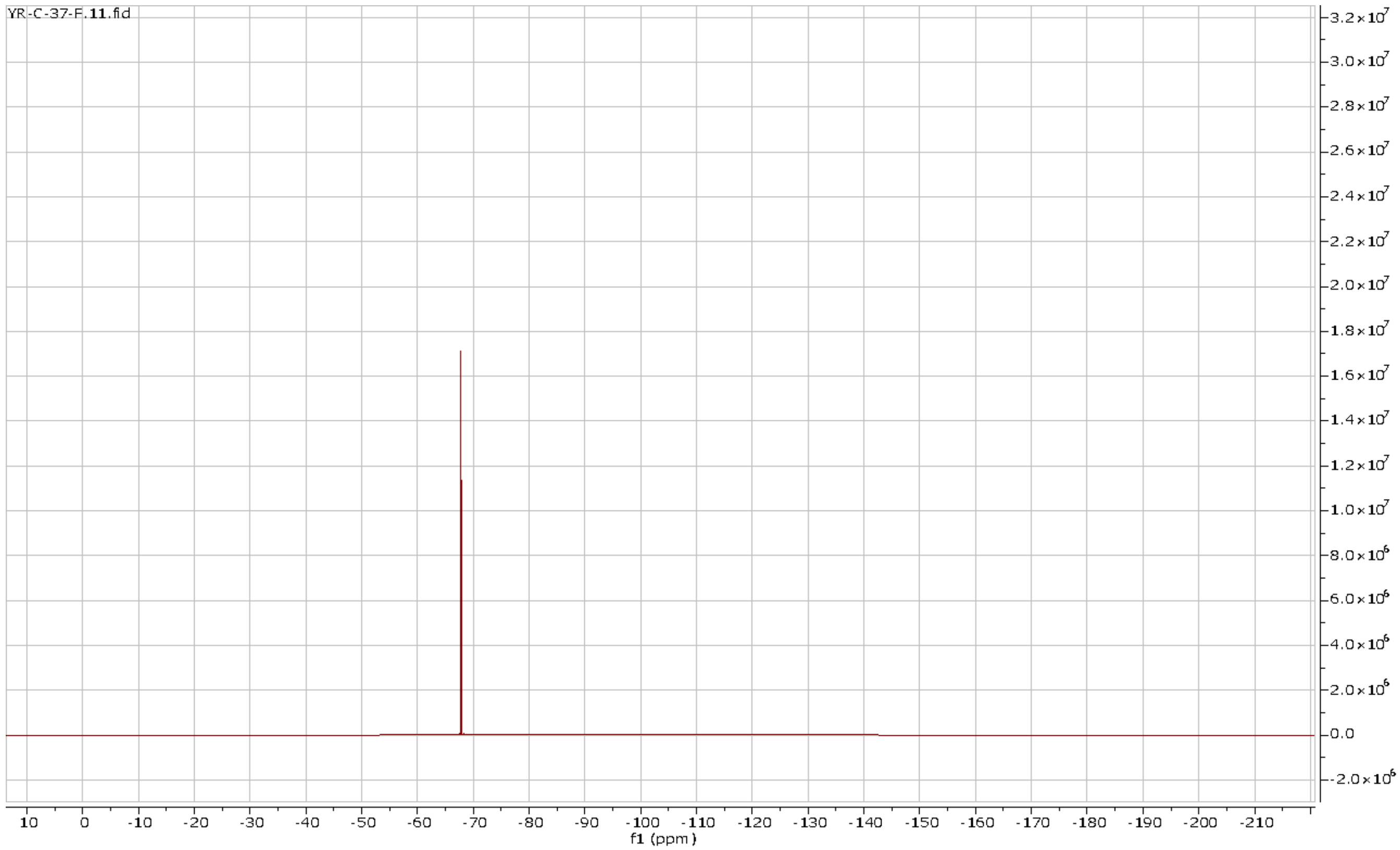

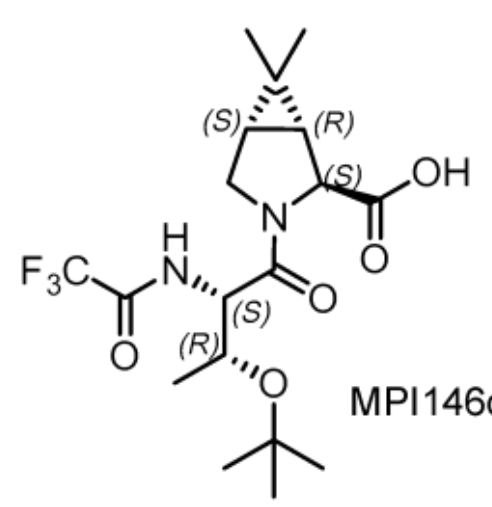

MPI146c

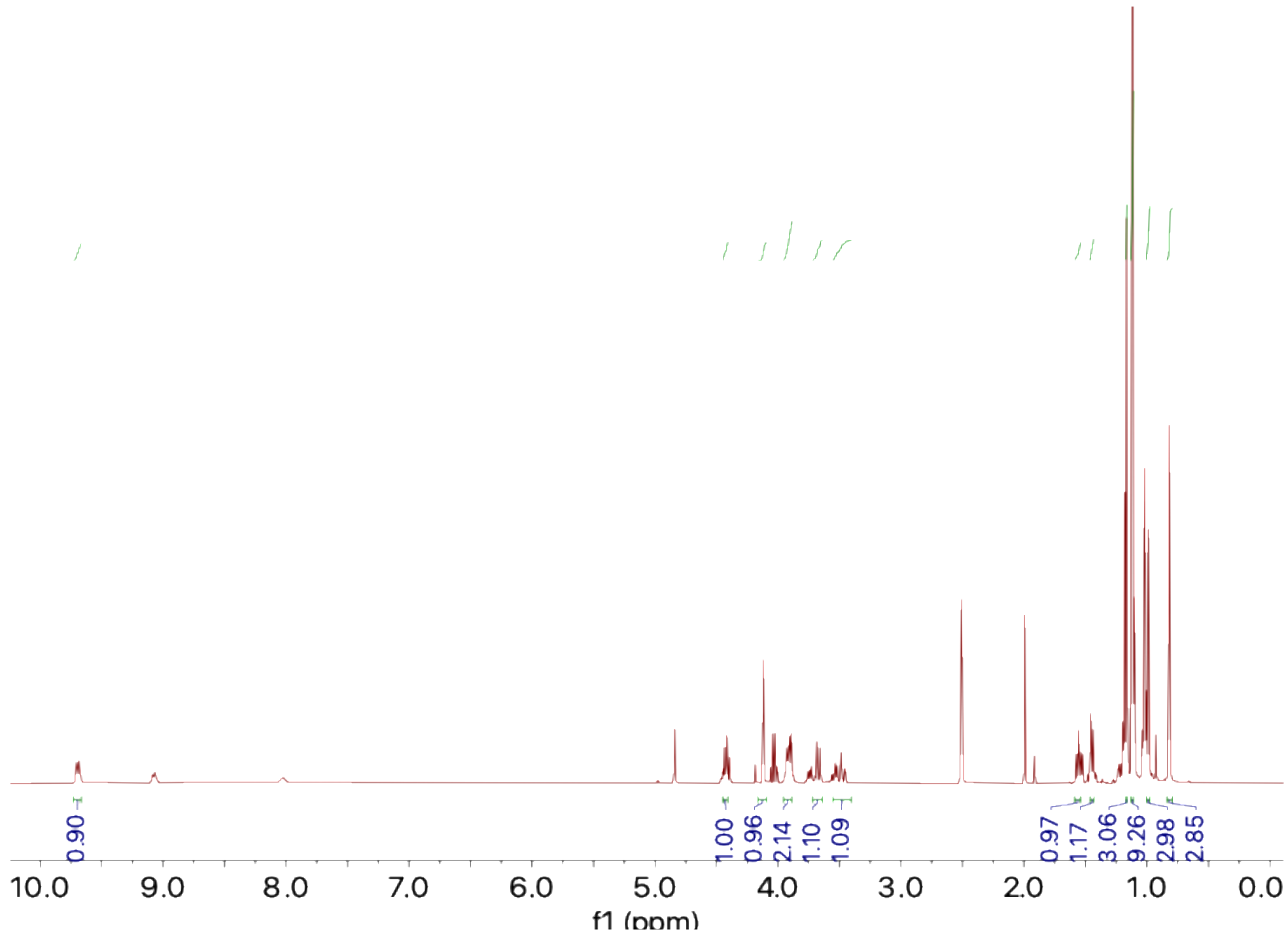
0.90
1.00
0.96
2.14
1.10
1.09
0.97
1.17
3.06
9.26
2.98
2.85
10.0
9.0
8.0
7.0
6.0
5.0
4.0
3.0
2.0
1.0
0.0
f1 (ppm)

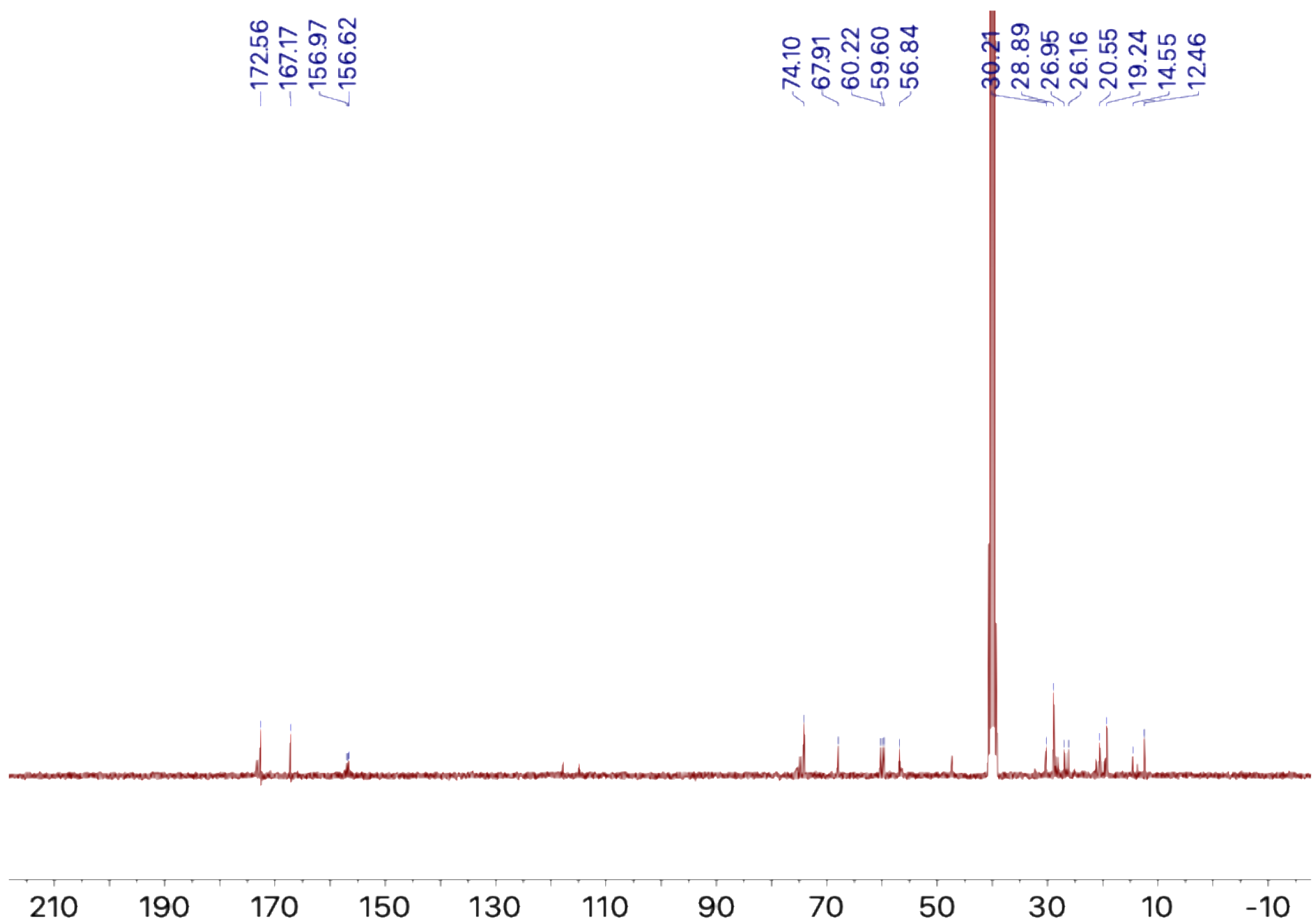
172.56
167.17
156.97
156.62
74.10
67.91
60.22
59.60
56.84
30.21
28.89
26.95
26.16
20.55
19.24
14.55
12.46
210
190
170
150
130
110
90
70
50
30
10
-10

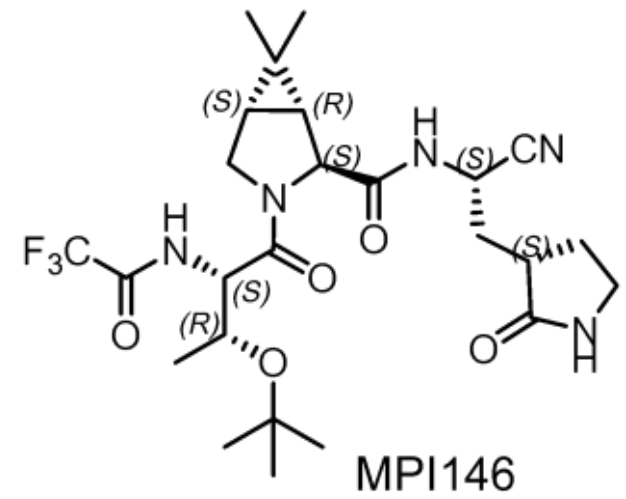

MPI146

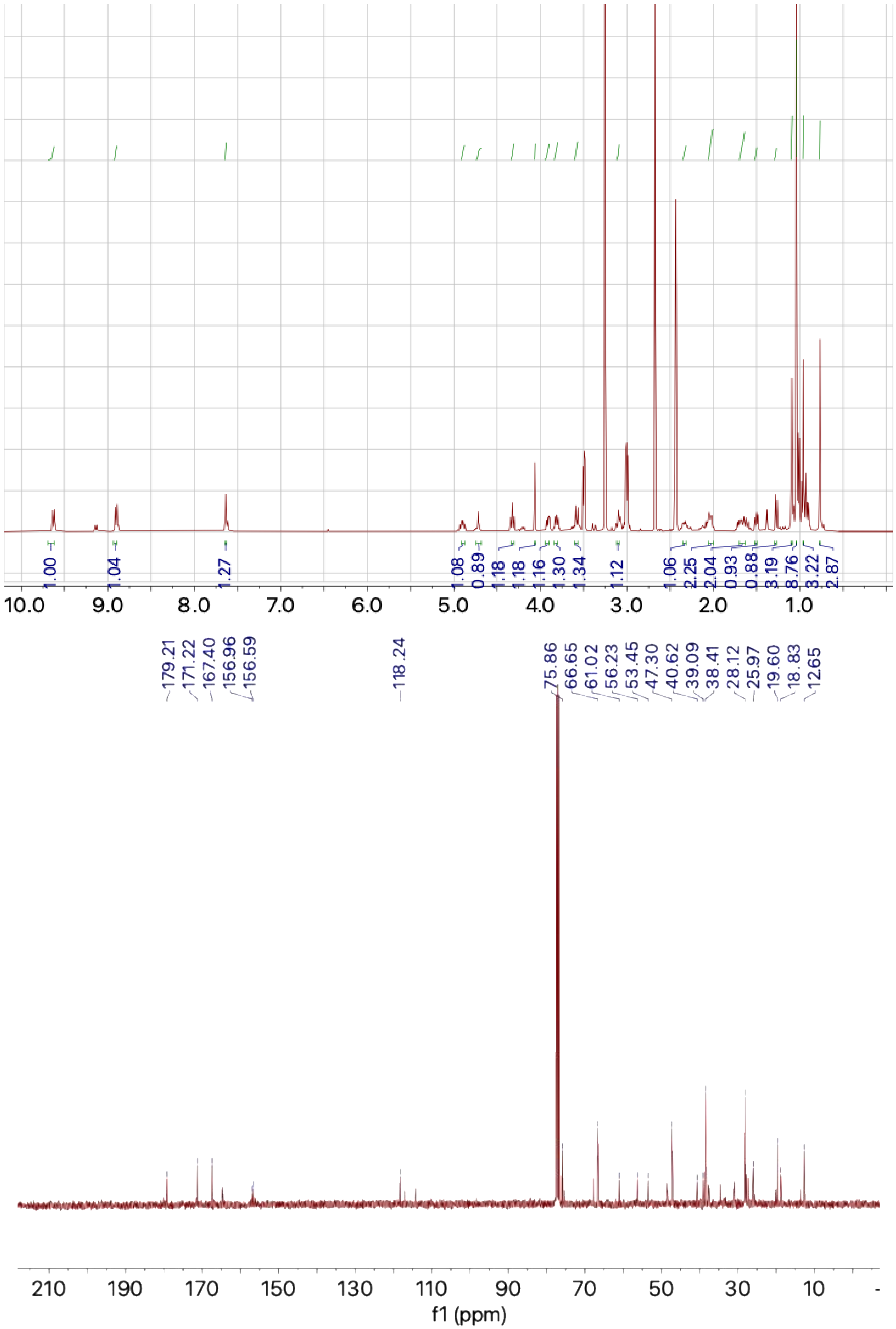

1.00
1.04
1.27
1.08
0.89
1.18
1.18
1.16
1.30
1.34
1.12
1.06
2.25
2.04
0.93
0.88
3.19
8.76
3.22
2.87
10.0
9.0
8.0
7.0
6.0
5.0
4.0
3.0
2.0
1.0
179.21
171.22
167.40
156.96
156.59
118.24
75.86
66.65
61.02
56.23
53.45
47.30
40.62
39.09
38.41
28.12
25.97
19.60
18.83
12.65
210
190
170
150
130
110
90
70
50
30
10
f1 (ppm)

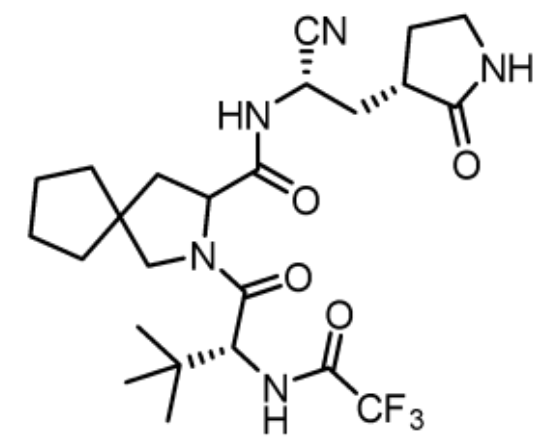

MPI147

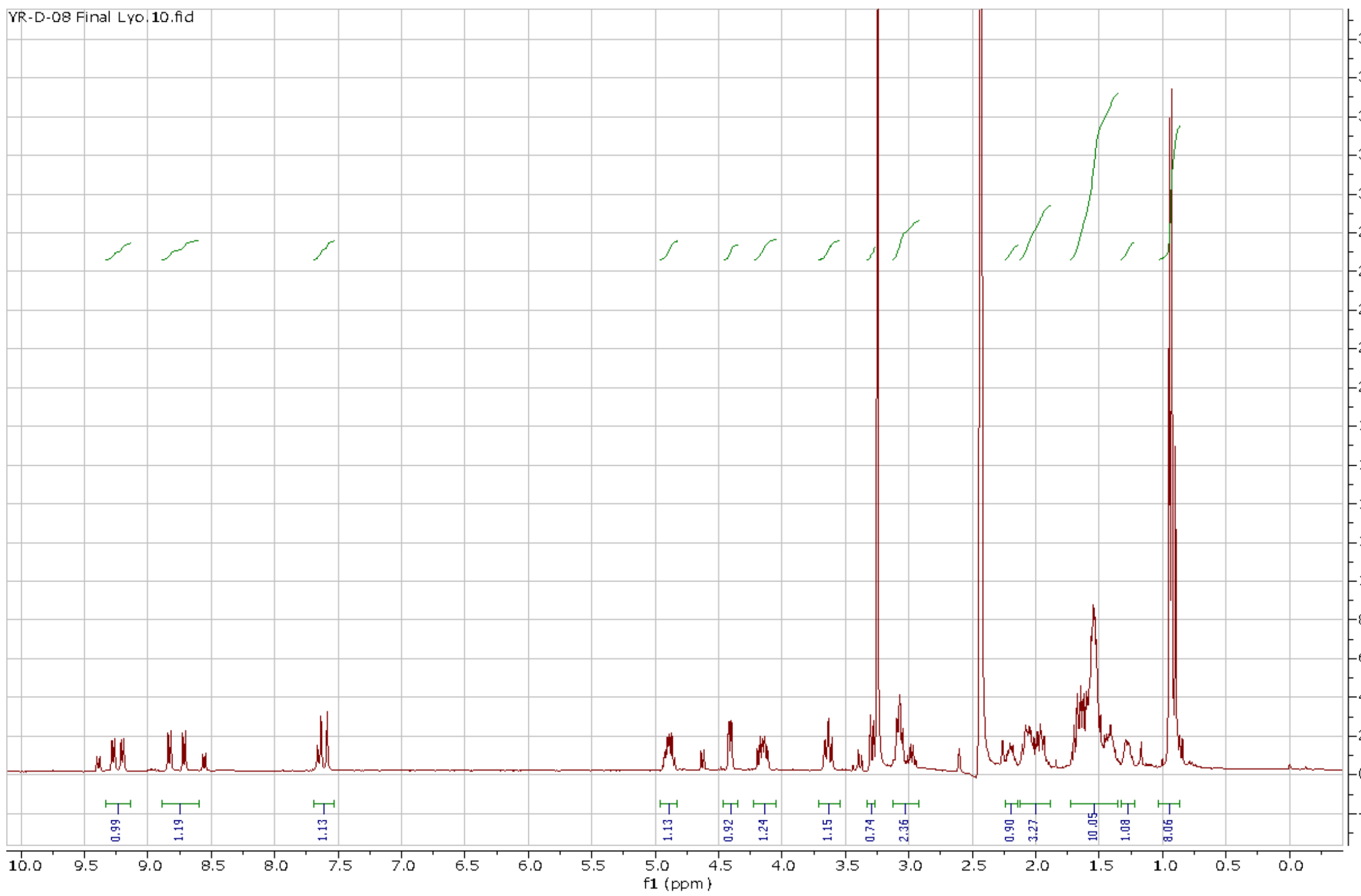

YR-D-08 Final Lyo.10.fid
0.99
1.19
1.13
1.13
0.92
1.24
1.15
0.74
2.36
0.90
3.27
10.05
1.08
8.06
f1 (ppm)

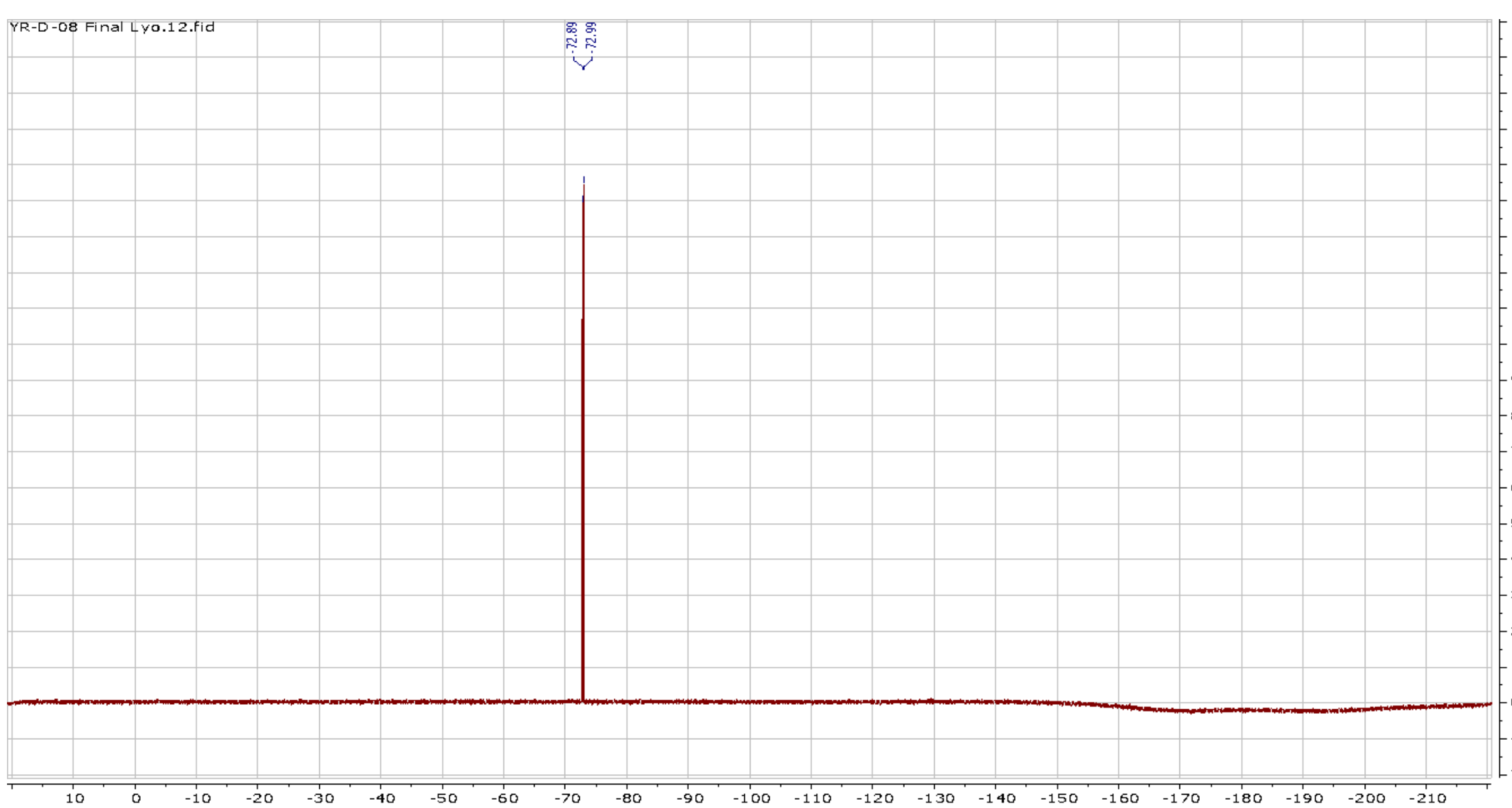

YR-D-08 Final Lyo.12.fid
-72.89
-72.99
f1 (ppm)

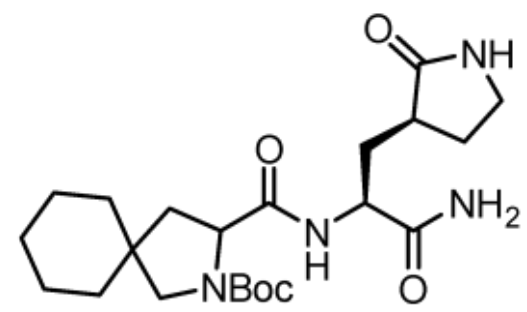

MPI148b

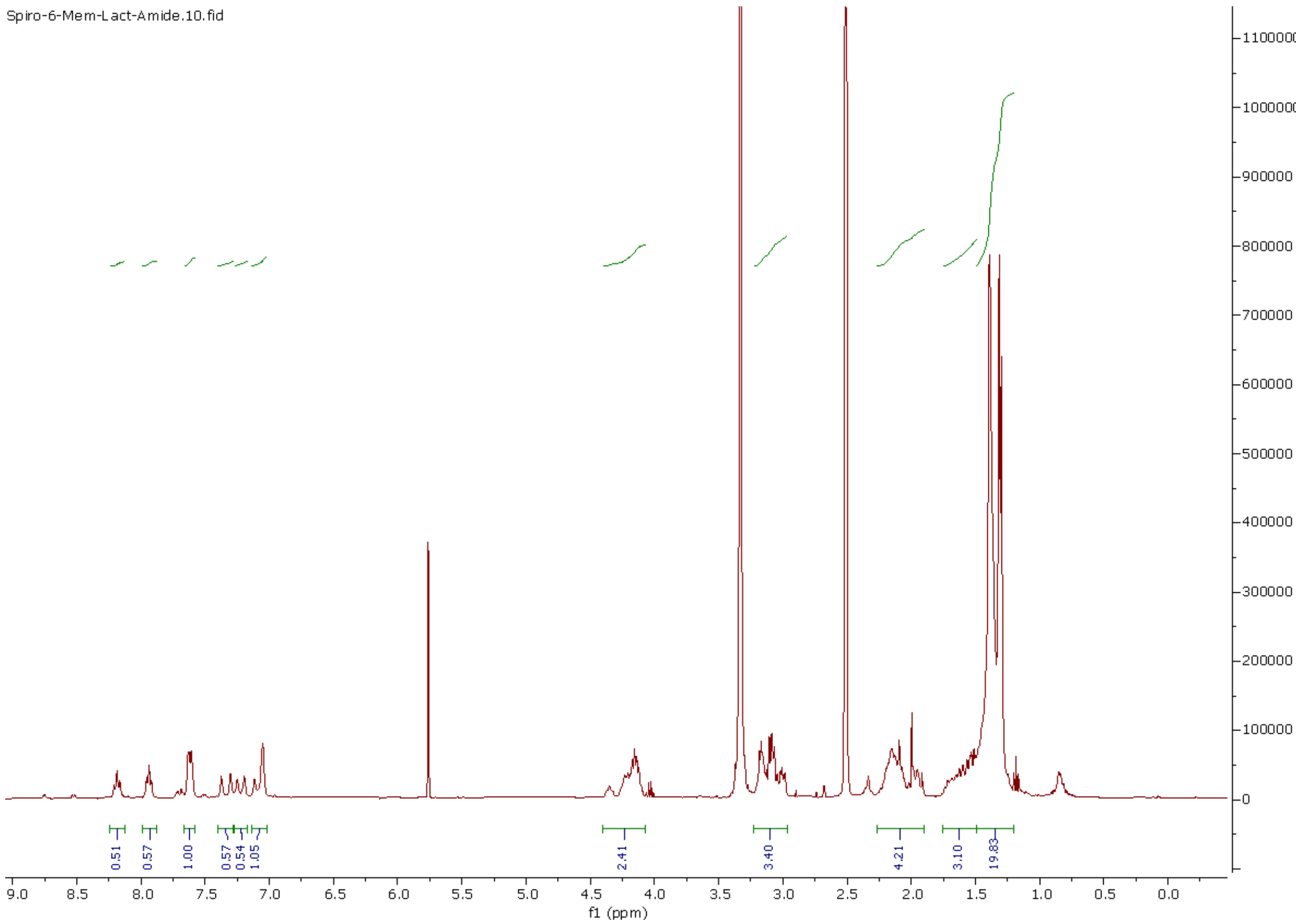

Spiro-6-Mem-Lact-Amide.10.fid
1100000
1000000
900000
800000
700000
600000
500000
400000
300000
200000
100000
0
0.51
0.57
1.00
0.57
0.54
1.05
2.41
3.40
4.21
3.10
19.83
9.0
8.5
8.0
7.5
7.0
6.5
6.0
5.5
5.0
4.5
4.0
3.5
3.0
2.5
2.0
1.5
1.0
0.5
0.0
f1 (ppm)

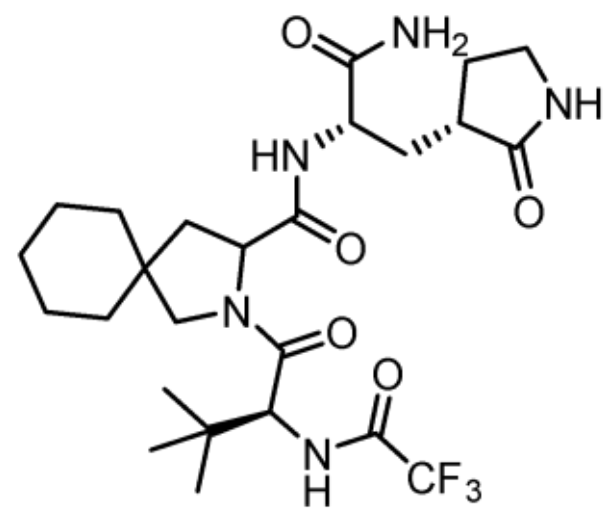

MPI148d

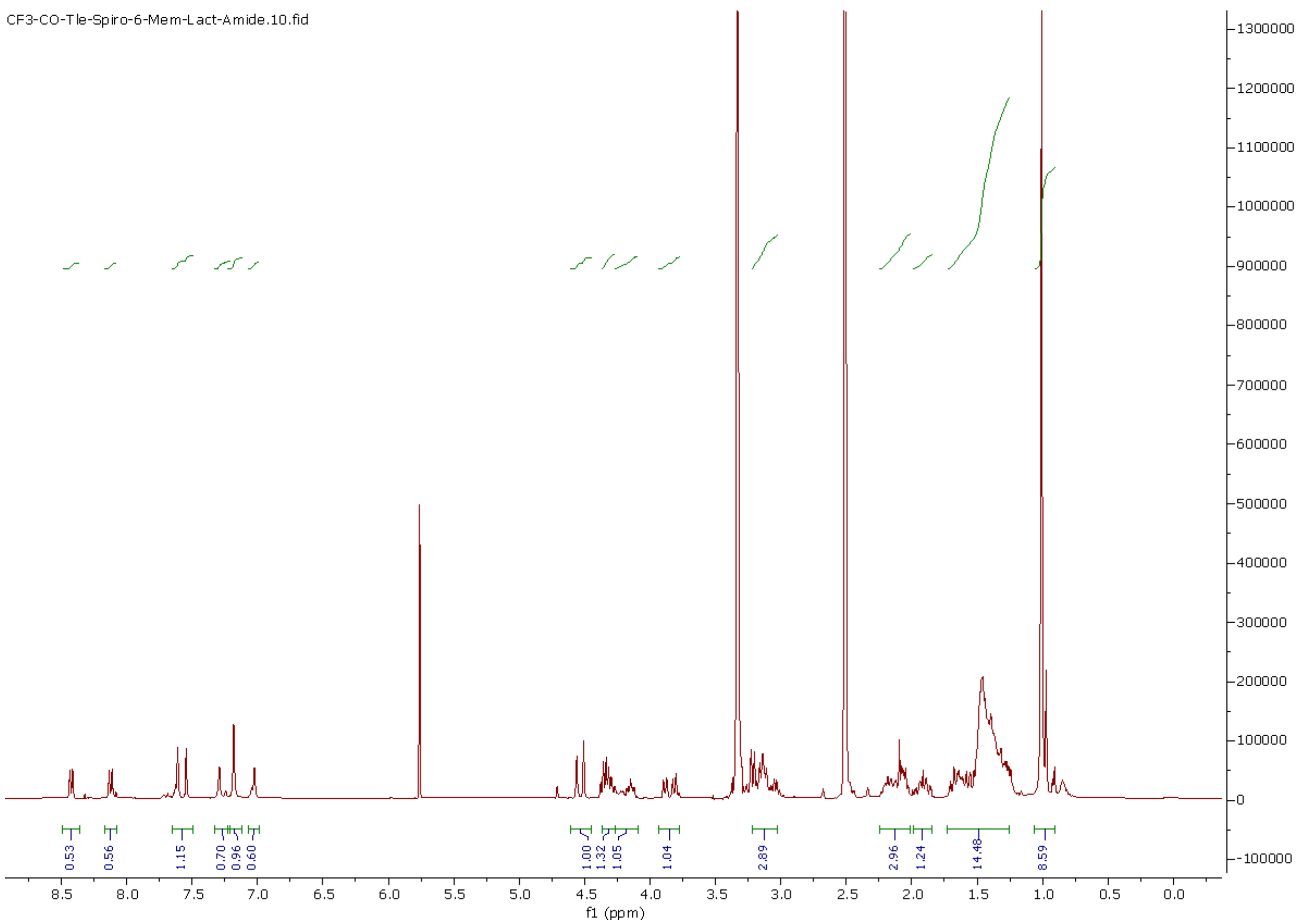
CF3-CO-Tle-Spiro-6-Mem-Lact-Amide.10.fid
0.53
0.56
1.15
0.70
0.96
0.60
1.00
1.32
1.05
1.04
2.89
2.96
1.24
14.48
8.59
f1 (ppm)

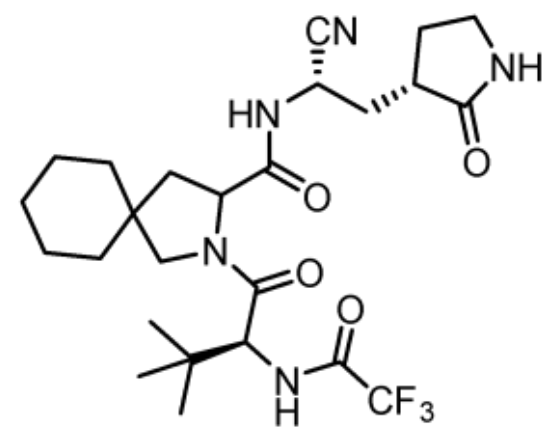

MPI148

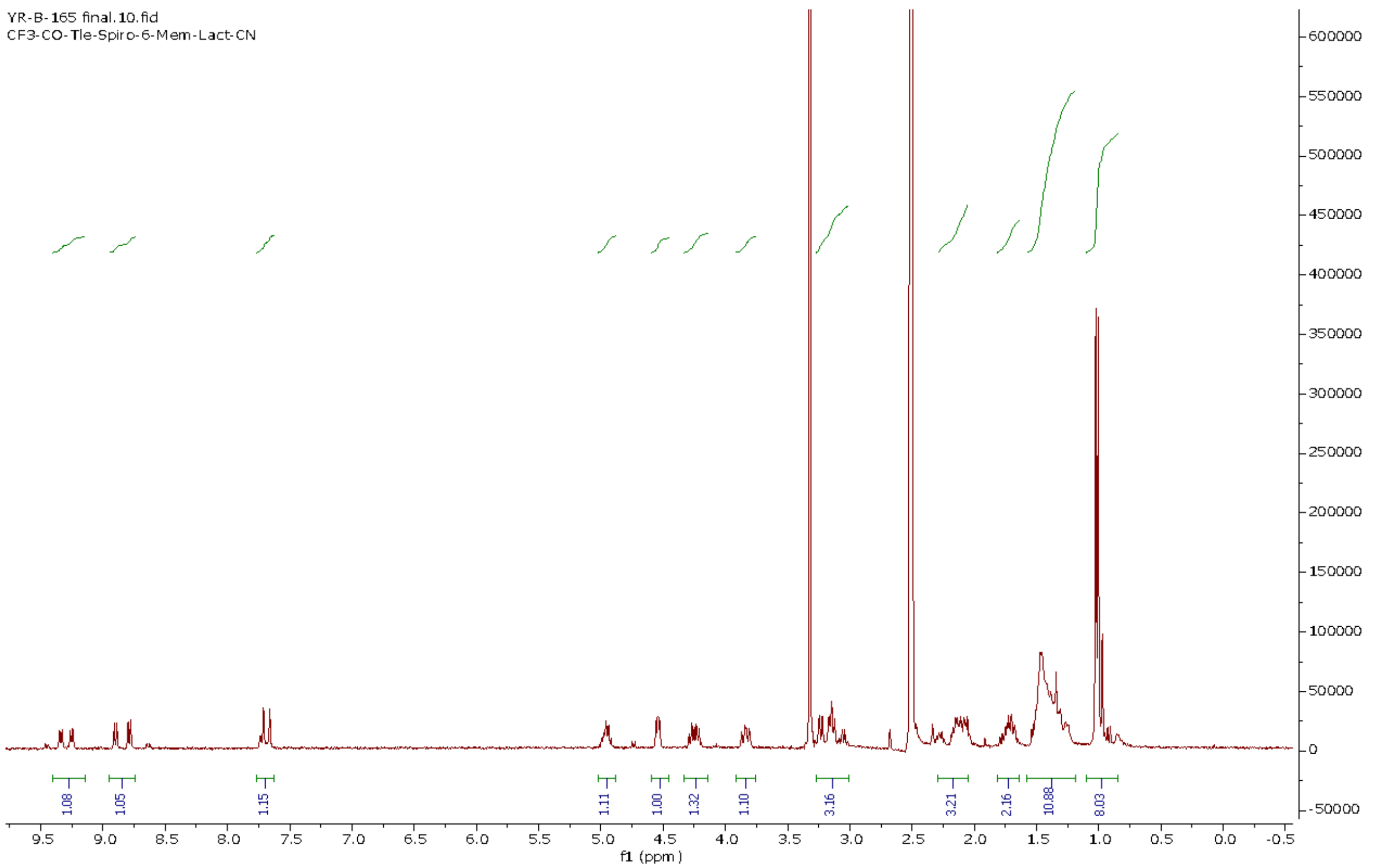
YR-B-165 final.10.fid
CF3-CO-Tle-Spiro-6-Mem-Lact-CN
1.08
1.05
1.15
1.11
1.00
1.32
1.10
3.16
3.21
2.16
10.88
8.03
f1 (ppm)

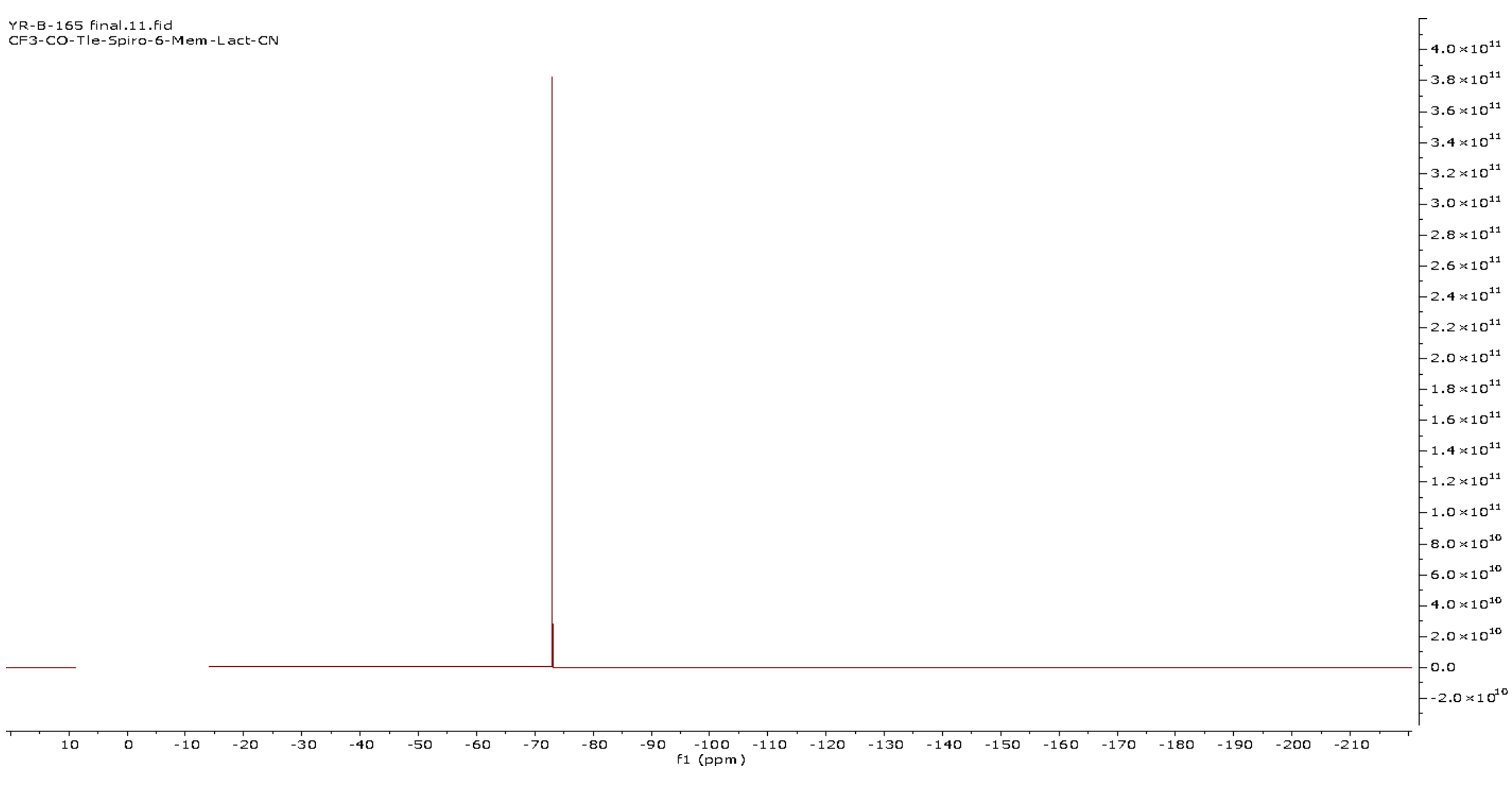
YR-B-165 final.11.fid
CF3-CO-Tle-Spiro-6-Mem-Lact-CN
f1 (ppm)

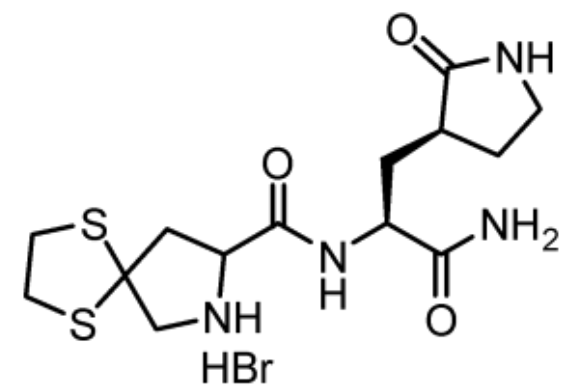

MPI149c

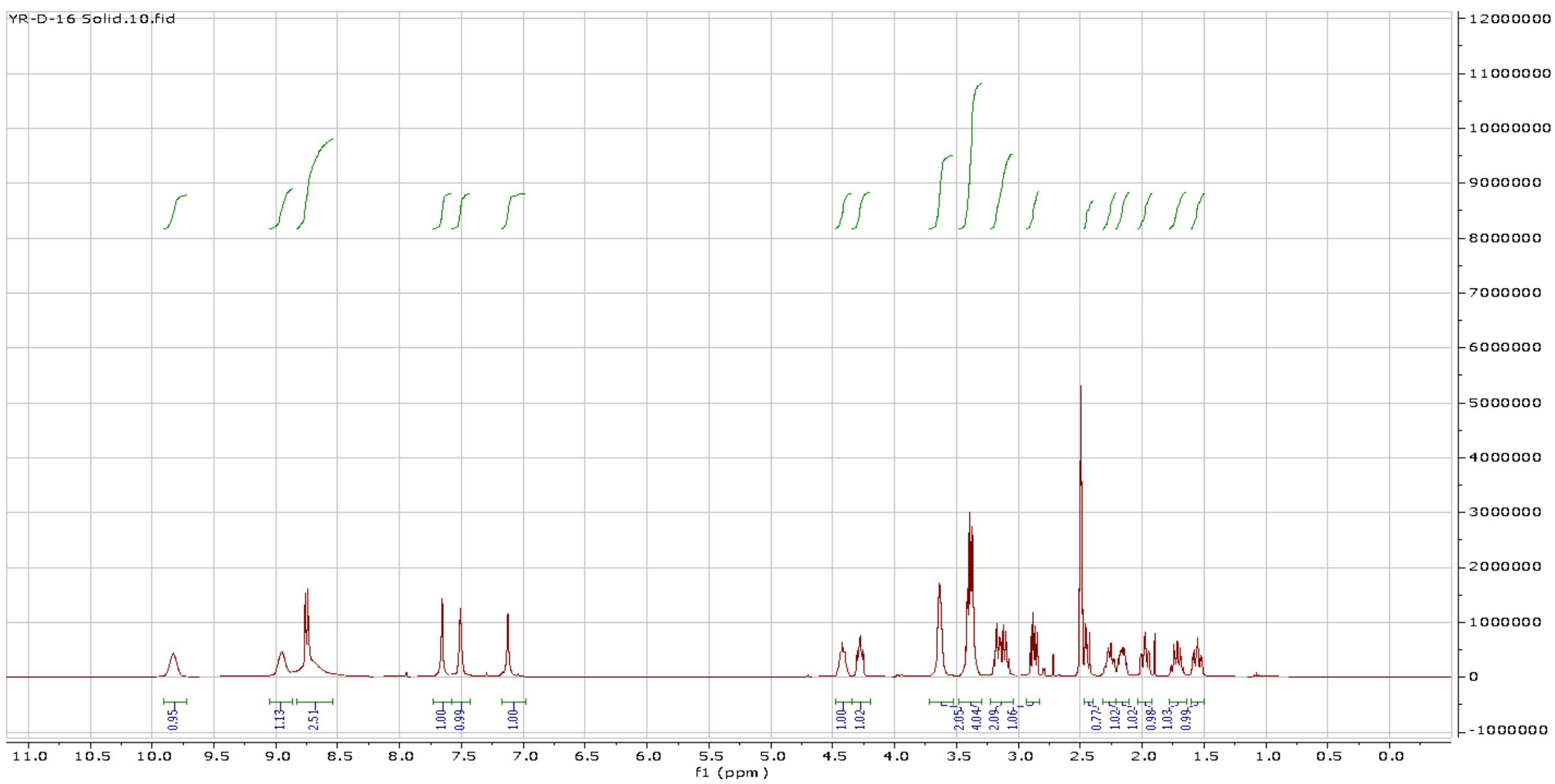

YR-D-16 Solid.10.fid
f1 (ppm)

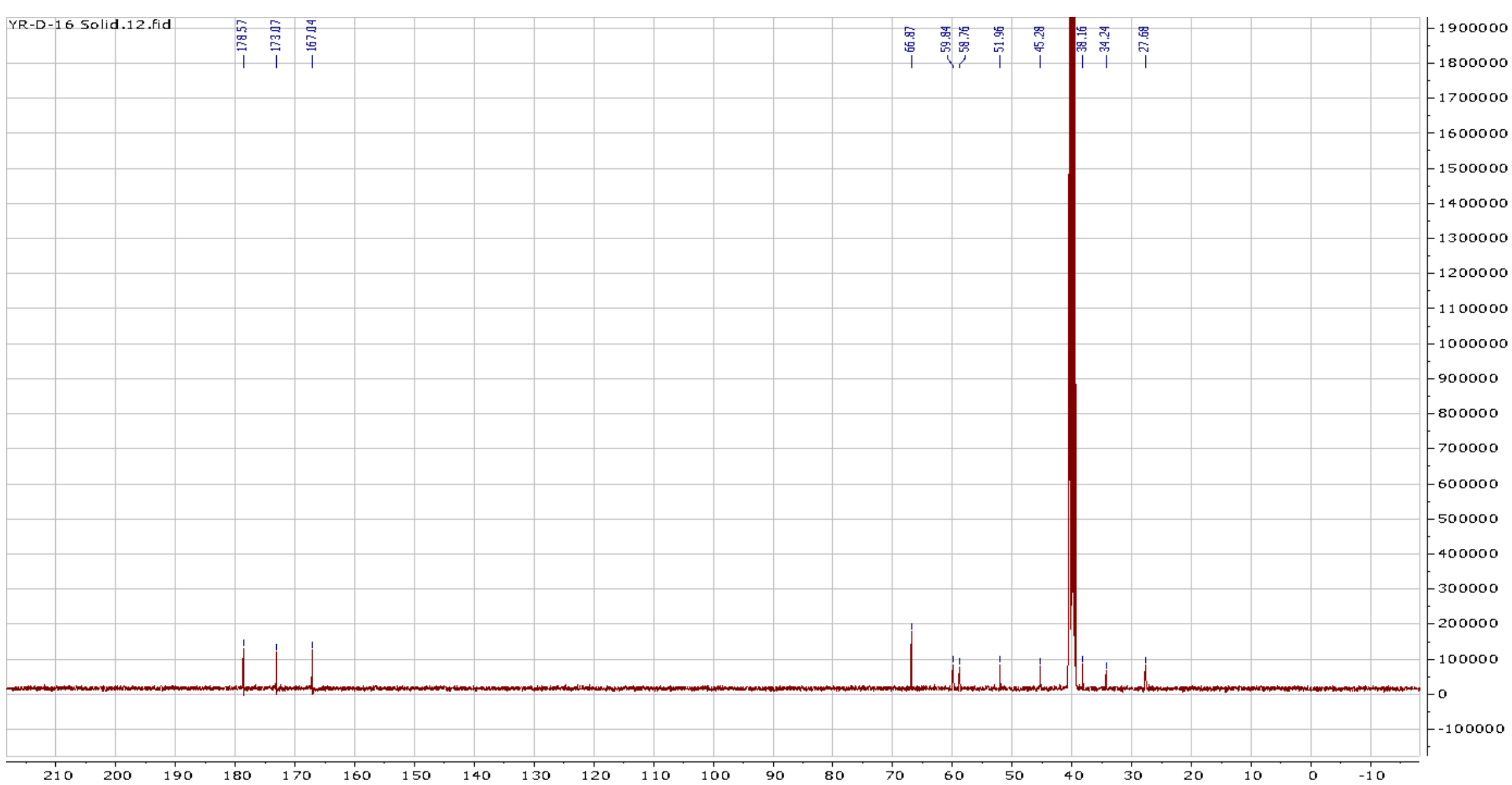

YR-D-16 Solid.12.fid
178.57
173.07
167.04
66.87
59.84
58.76
51.96
45.28
38.16
34.24
27.68
f1 (ppm)

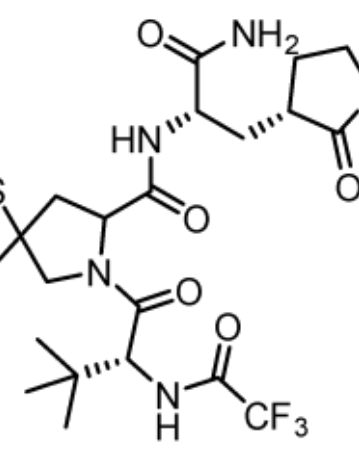

MPI149d

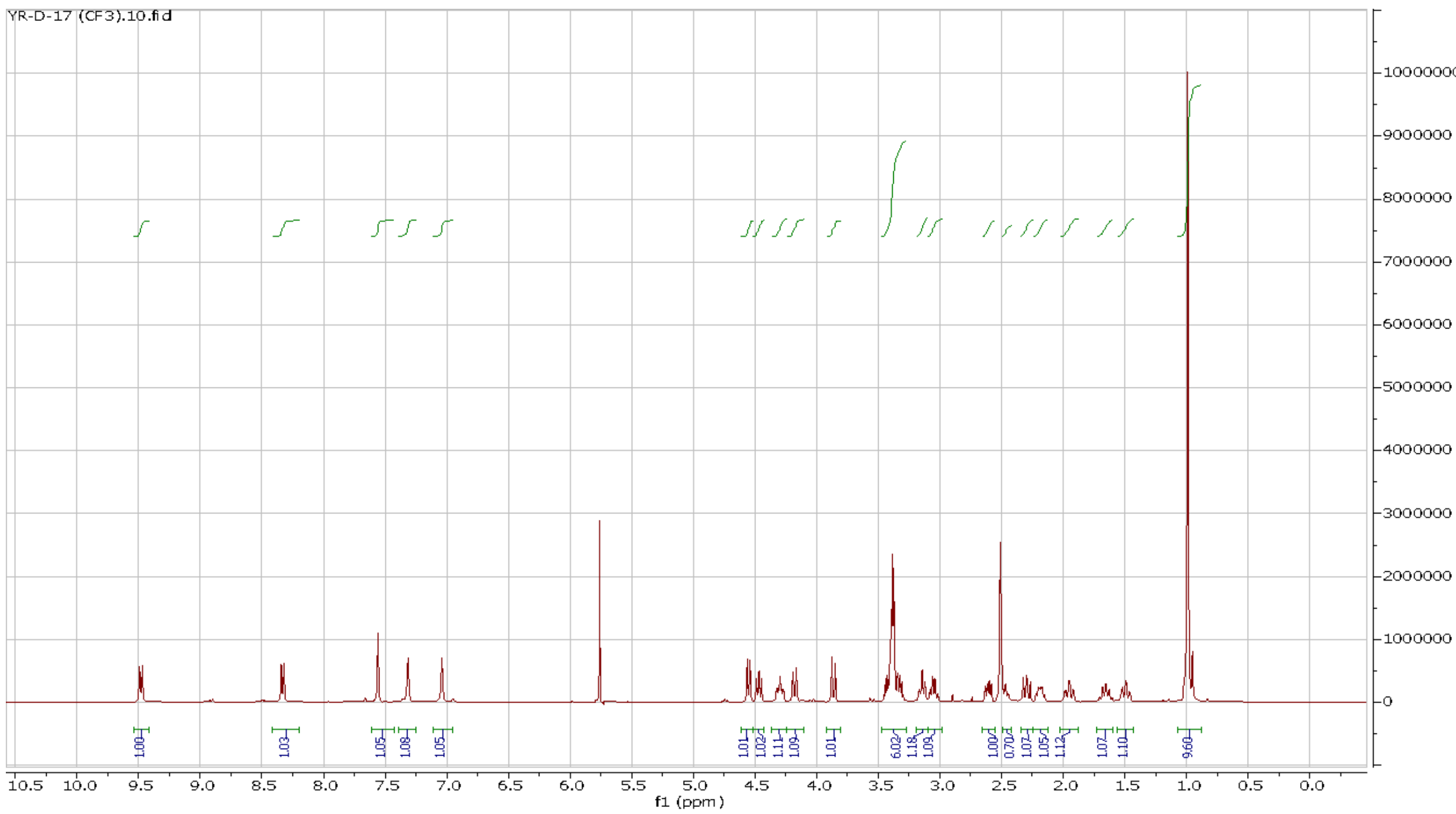

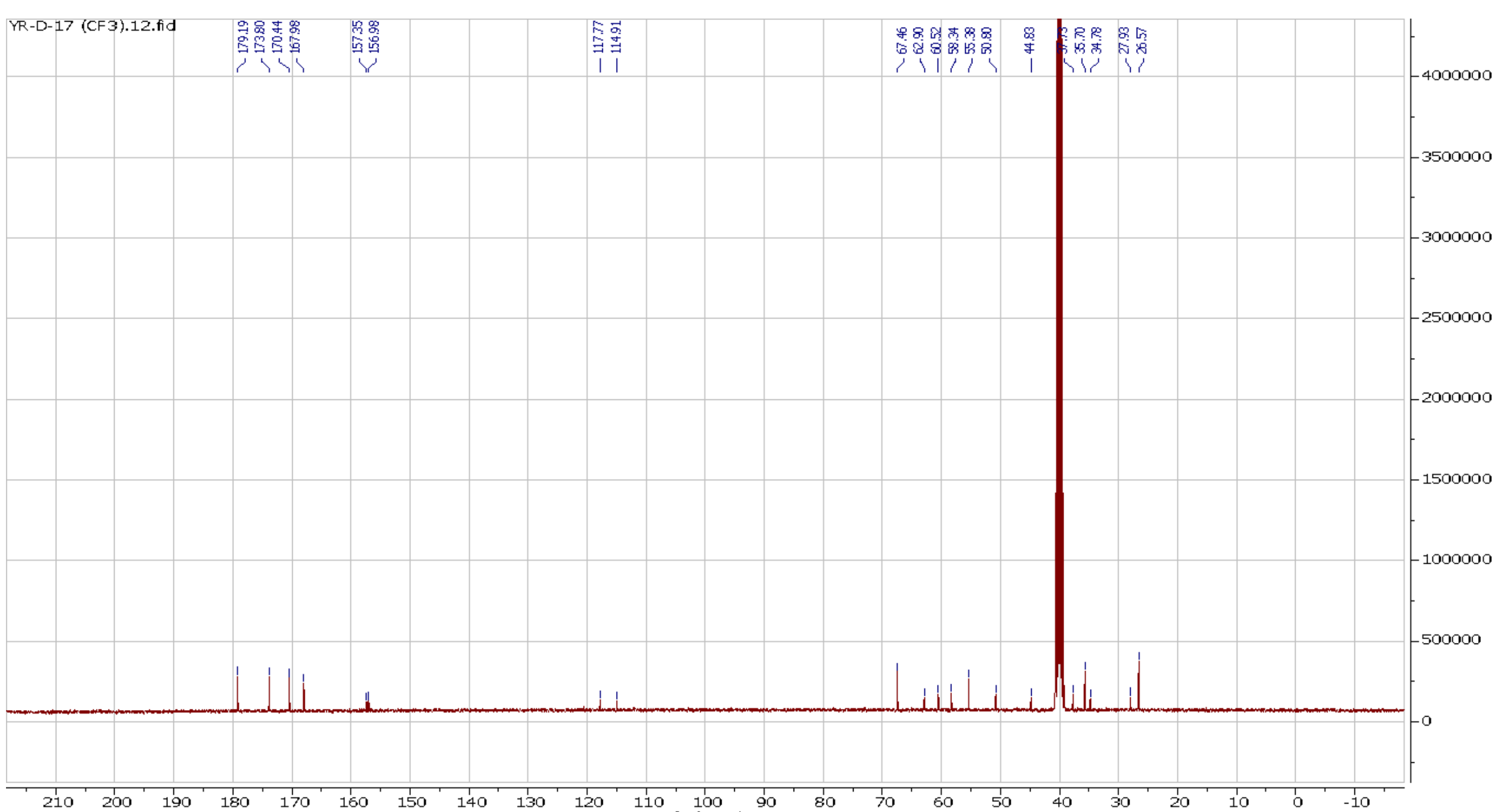

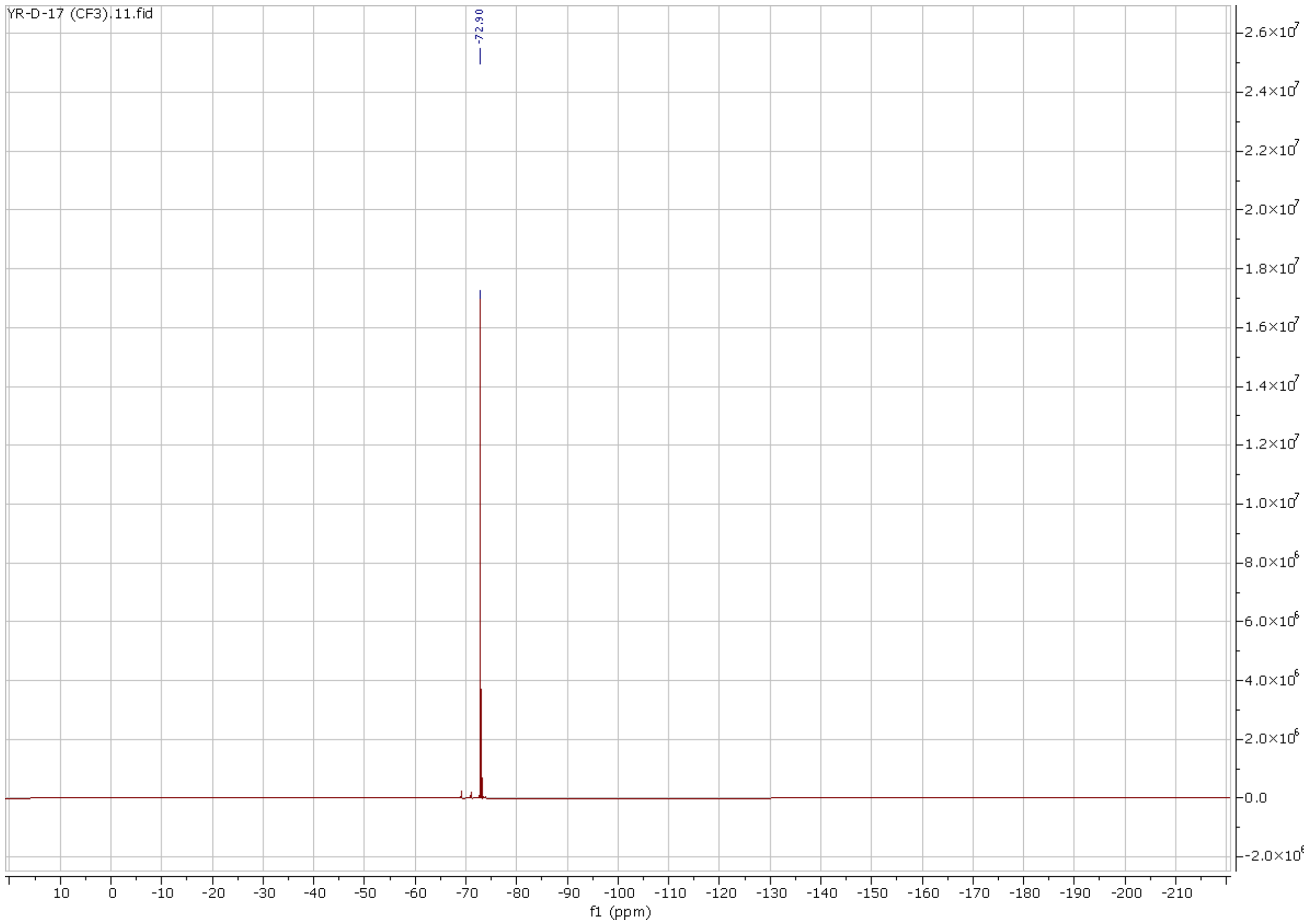
YR-D-17 (CF3).11.fid
-72.90
2.6×10^7
2.4×10^7
2.2×10^7
2.0×10^7
1.8×10^7
1.6×10^7
1.4×10^7
1.2×10^7
1.0×10^7
8.0×10^6
6.0×10^6
4.0×10^6
2.0×10^6
0.0
-2.0×10^6
10 0 -10 -20 -30 -40 -50 -60 -70 -80 -90 -100 -110 -120 -130 -140 -150 -160 -170 -180 -190 -200 -210
f1 (ppm)

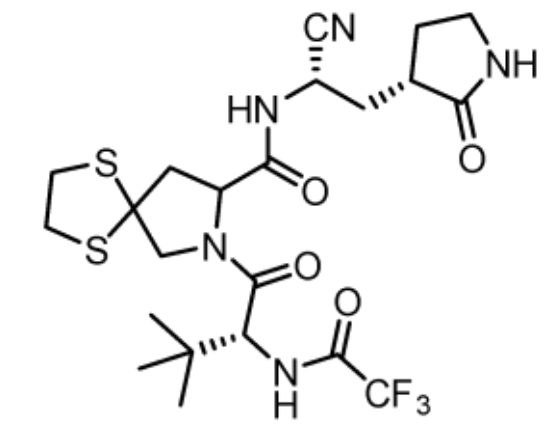

MPI149

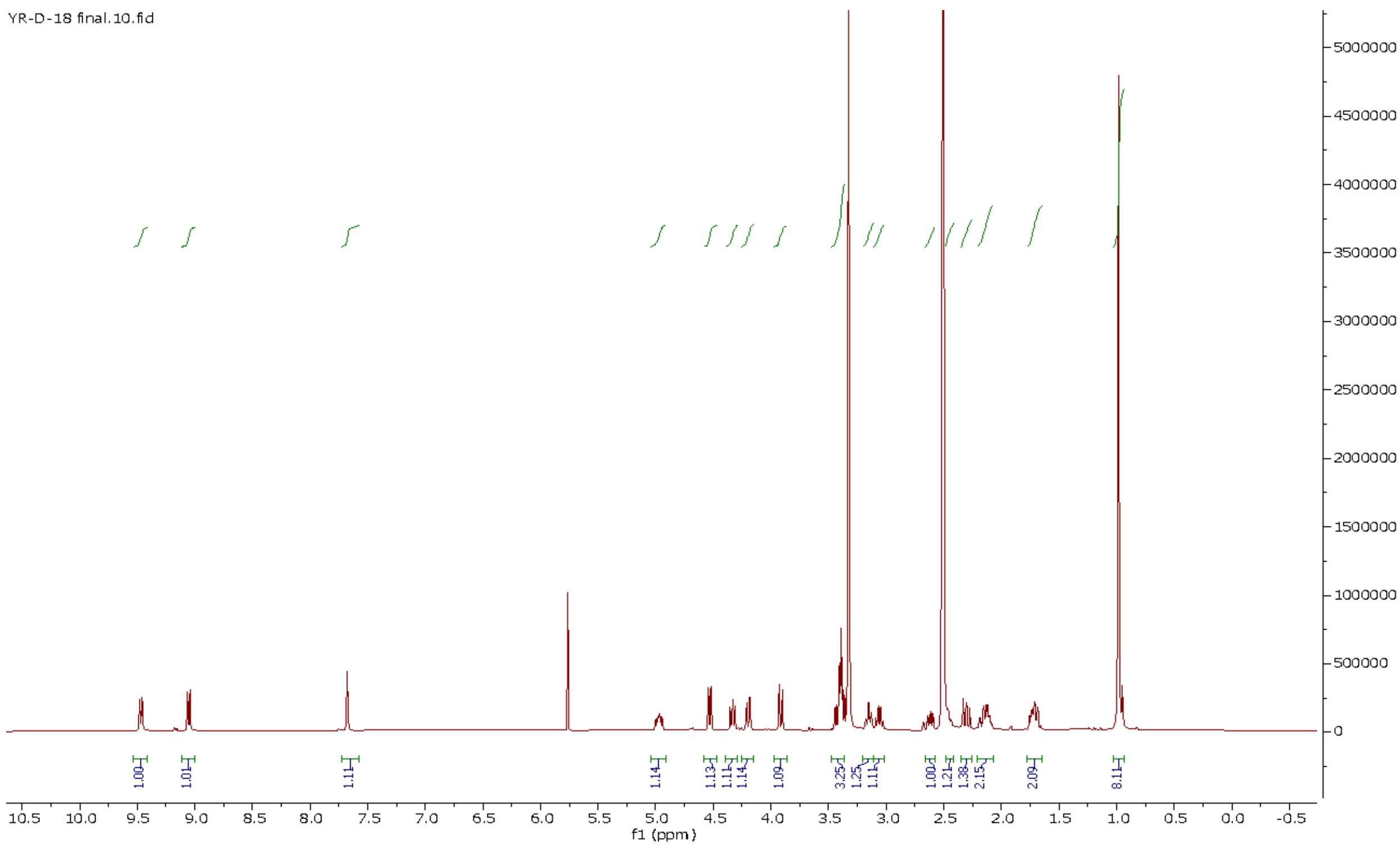

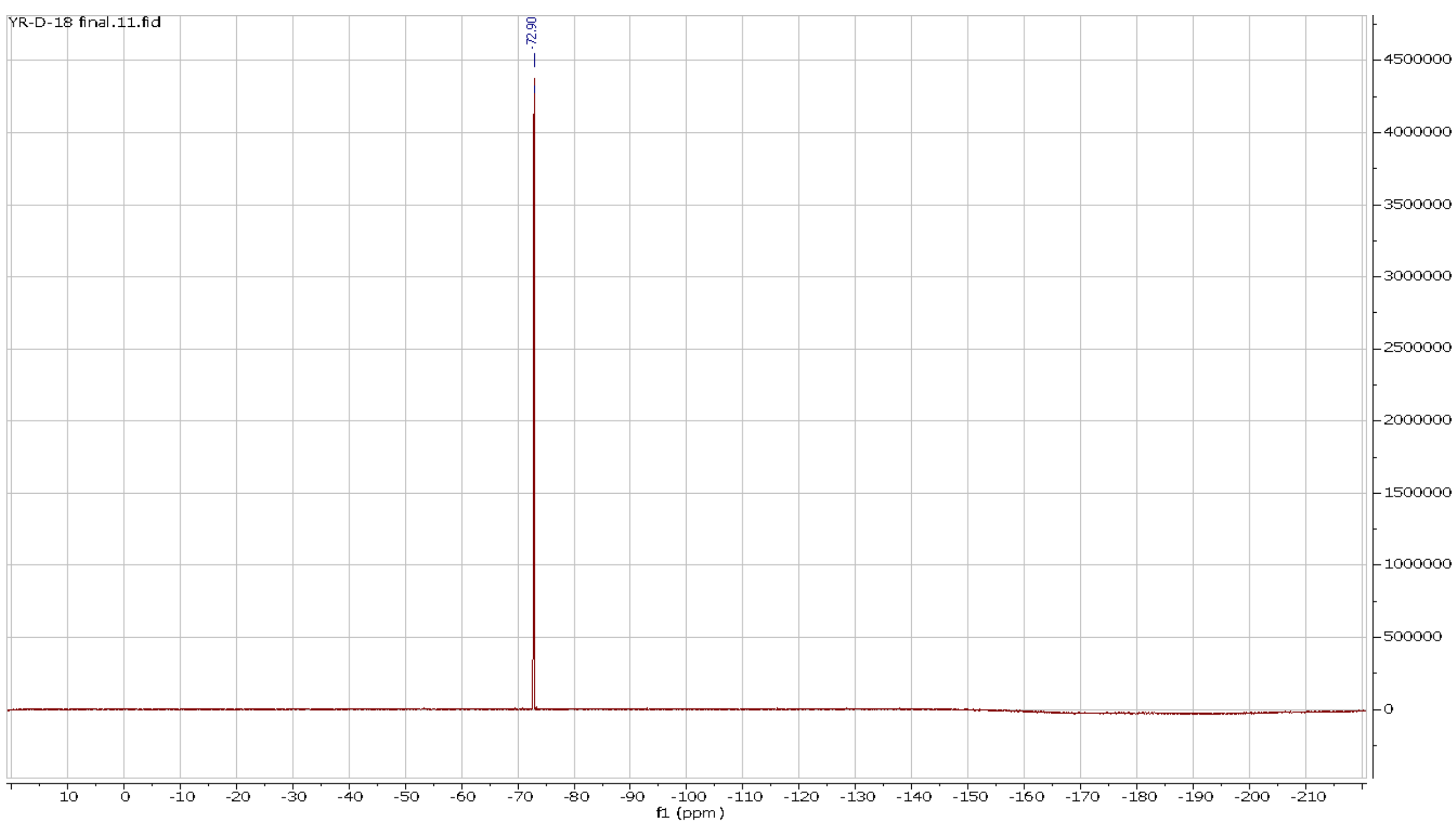

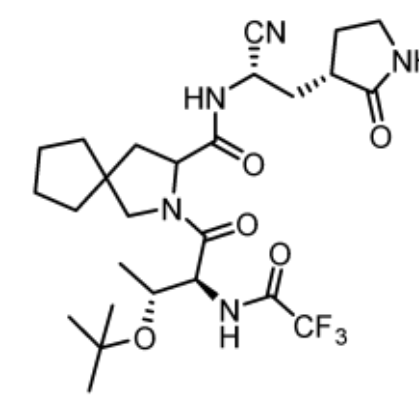
CN
HN
NH
O
N
O
O
O
N
H
CF3

MPI150

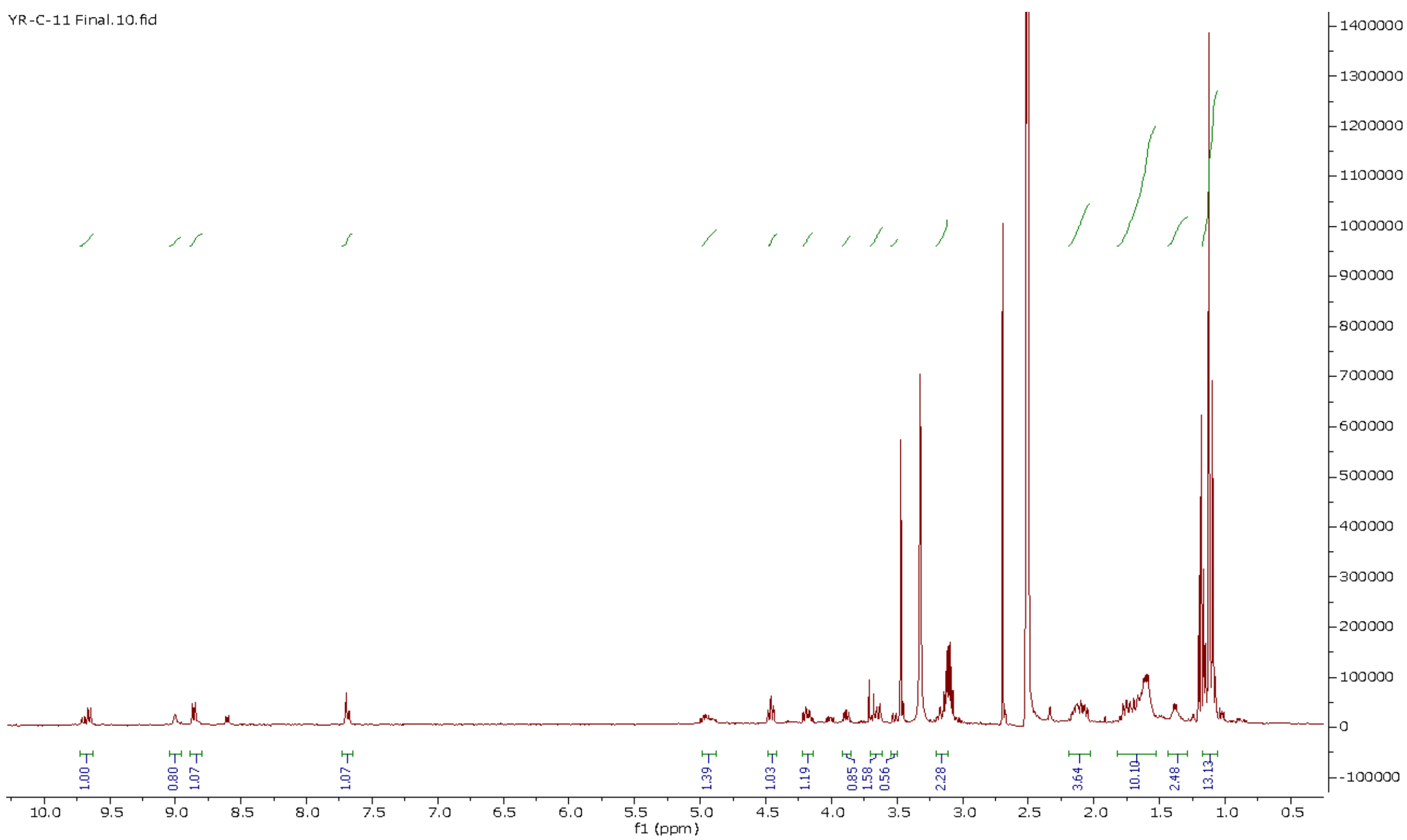
YR-C-11 Final.10.fid
1400000
1300000
1200000
1100000
1000000
900000
800000
700000
600000
500000
400000
300000
200000
100000
0
-100000
1.00
0.80
1.07
1.07
1.39
1.03
1.19
0.85
1.58
0.56
2.28
3.64
10.10
2.48
13.13
10.0
9.5
9.0
8.5
8.0
7.5
7.0
6.5
6.0
5.5
5.0
4.5
4.0
3.5
3.0
2.5
2.0
1.5
1.0
0.5
f1 (ppm)

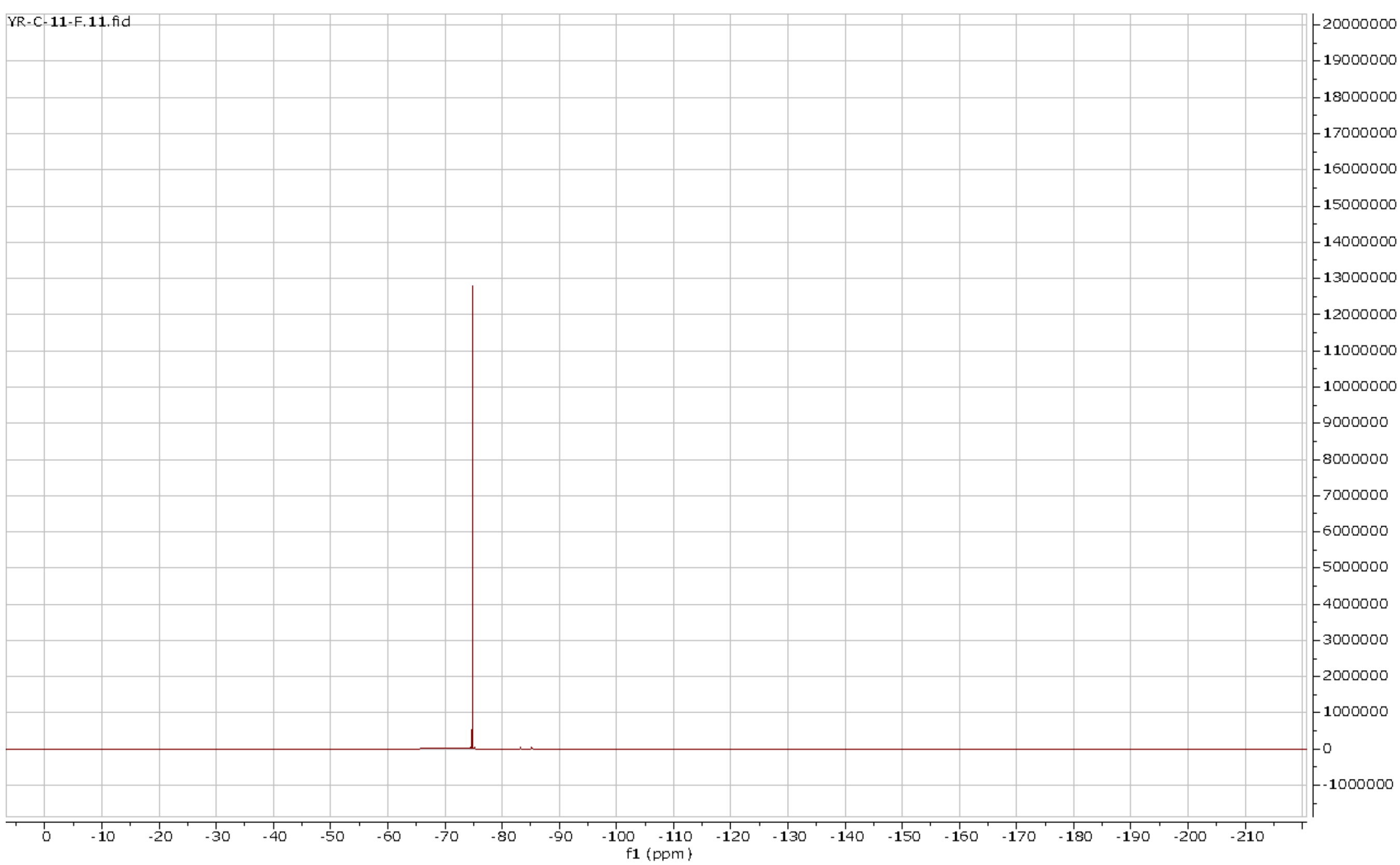
YR-C-11-F.11.fid
20000000
19000000
18000000
17000000
16000000
15000000
14000000
13000000
12000000
11000000
10000000
9000000
8000000
7000000
6000000
5000000
4000000
3000000
2000000
1000000
0
-1000000
0
-10
-20
-30
-40
-50
-60
-70
-80
-90
-100
-110
-120
-130
-140
-150
-160
-170
-180
-190
-200
-210
f1 (ppm)

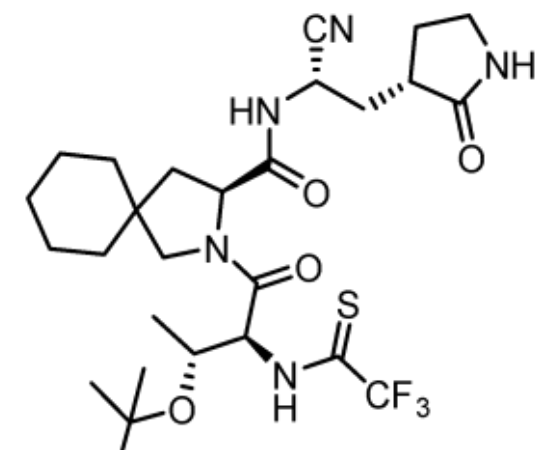

MPI151

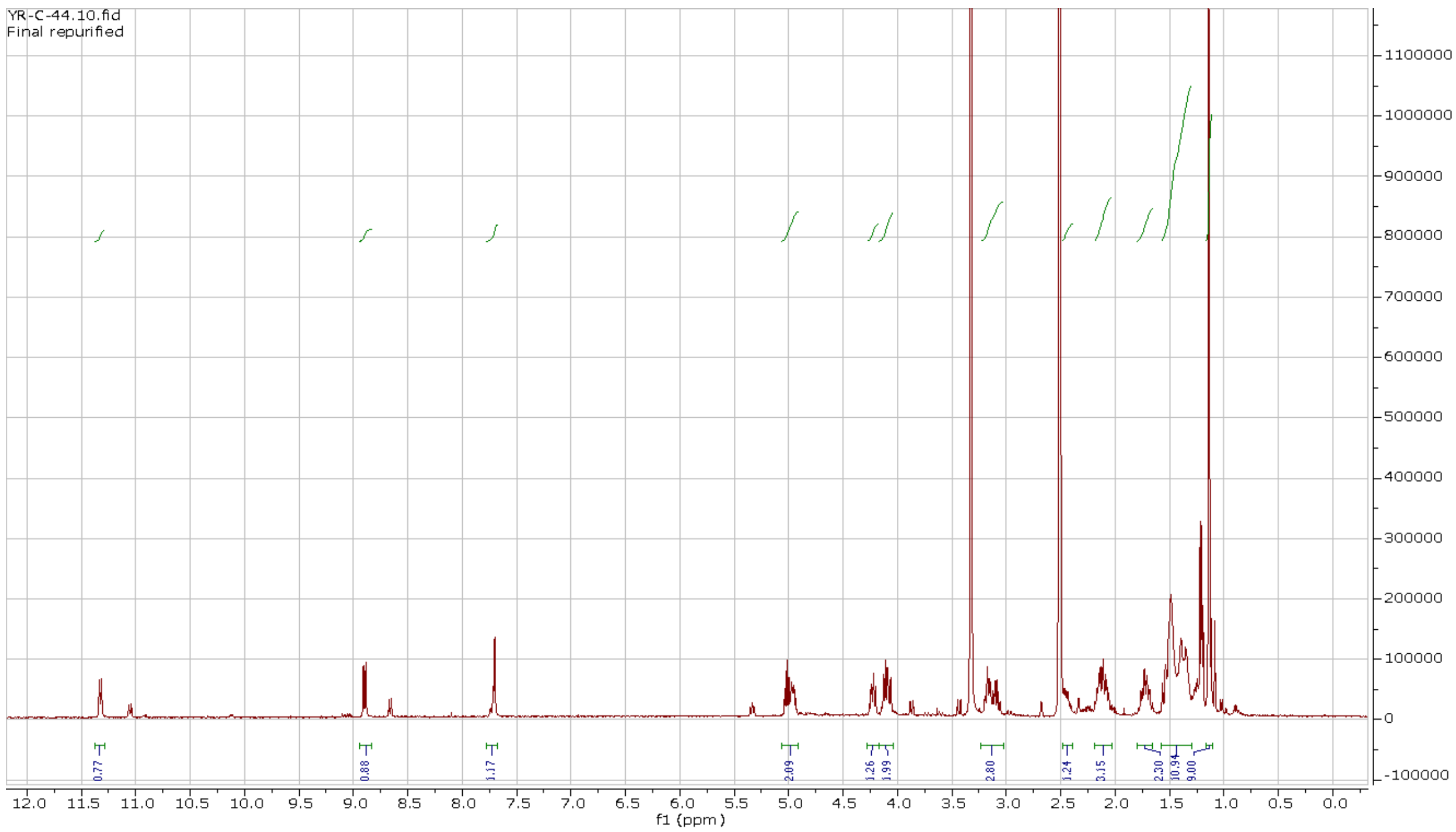

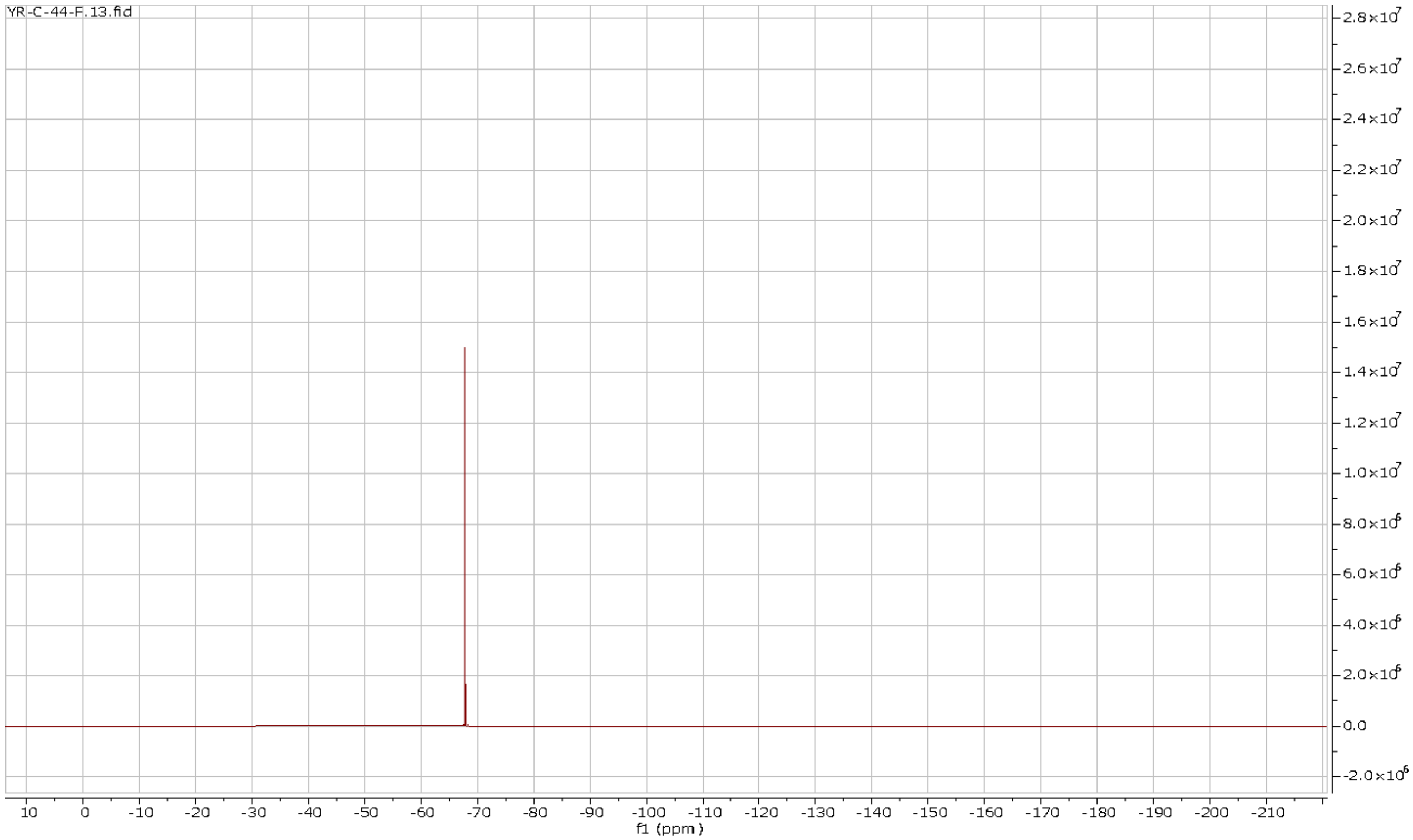
YR-C-44-F.13.fid
2.8×10^7
2.6×10^7
2.4×10^7
2.2×10^7
2.0×10^7
1.8×10^7
1.6×10^7
1.4×10^7
1.2×10^7
1.0×10^7
8.0×10^6
6.0×10^6
4.0×10^6
2.0×10^6
0.0
-2.0×10^6
10 0 -10 -20 -30 -40 -50 -60 -70 -80 -90 -100 -110 -120 -130 -140 -150 -160 -170 -180 -190 -200 -210
f1 (ppm)

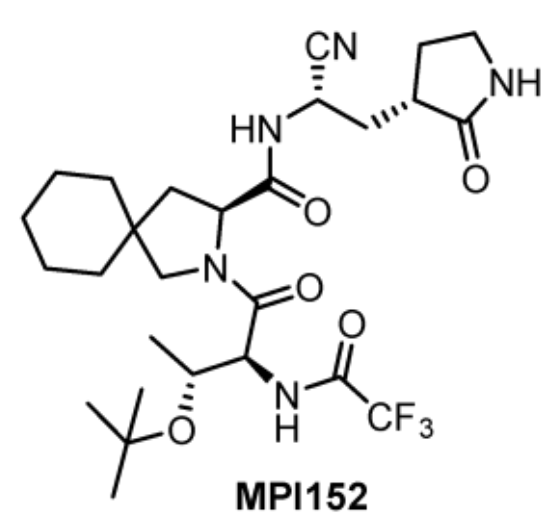
CN
NH
HN
O
O
N
O
O
N
H
CF3
O

MPI152

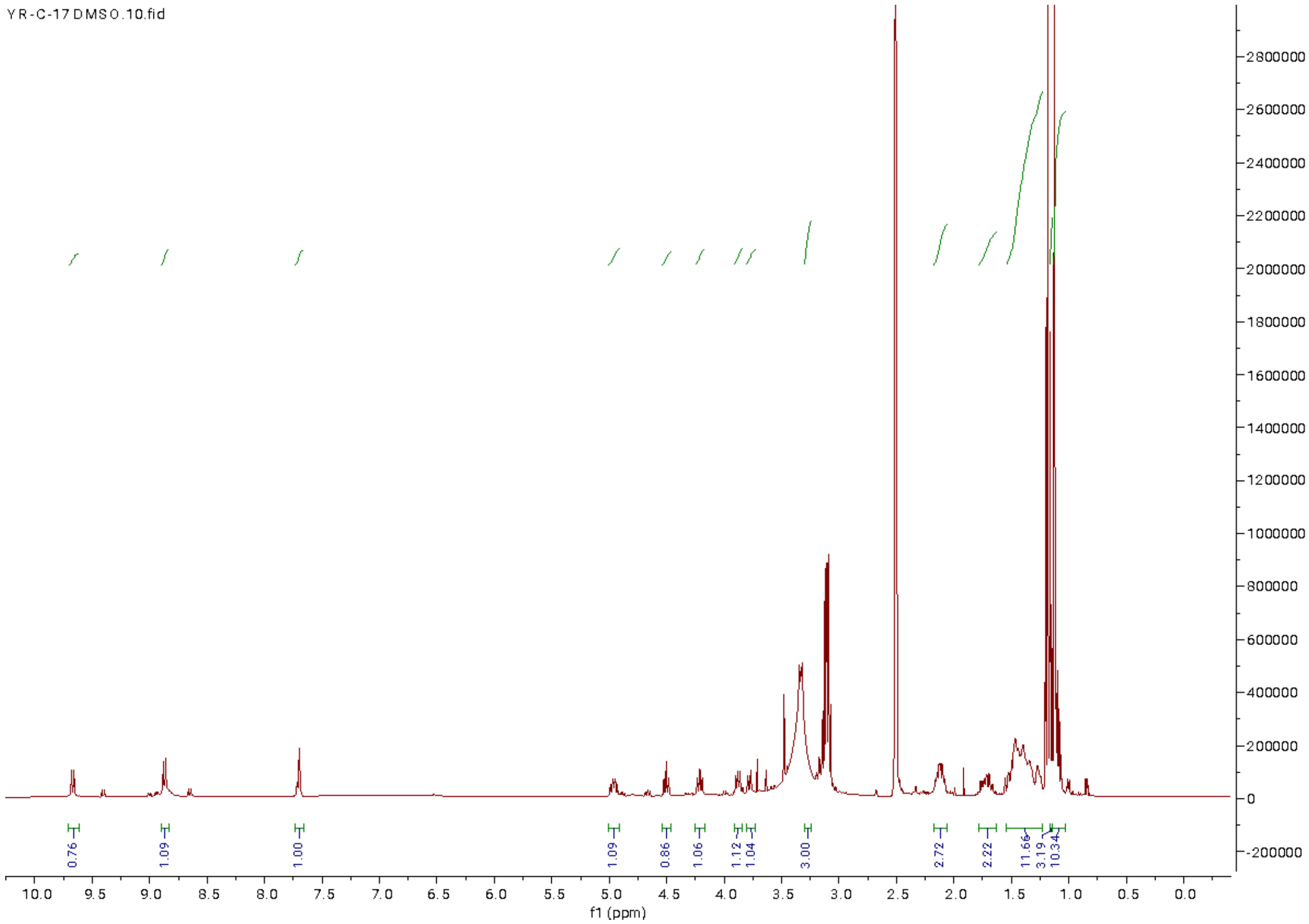

HPLC Purity of Final compound:

MPI-137 data of HPLC purity

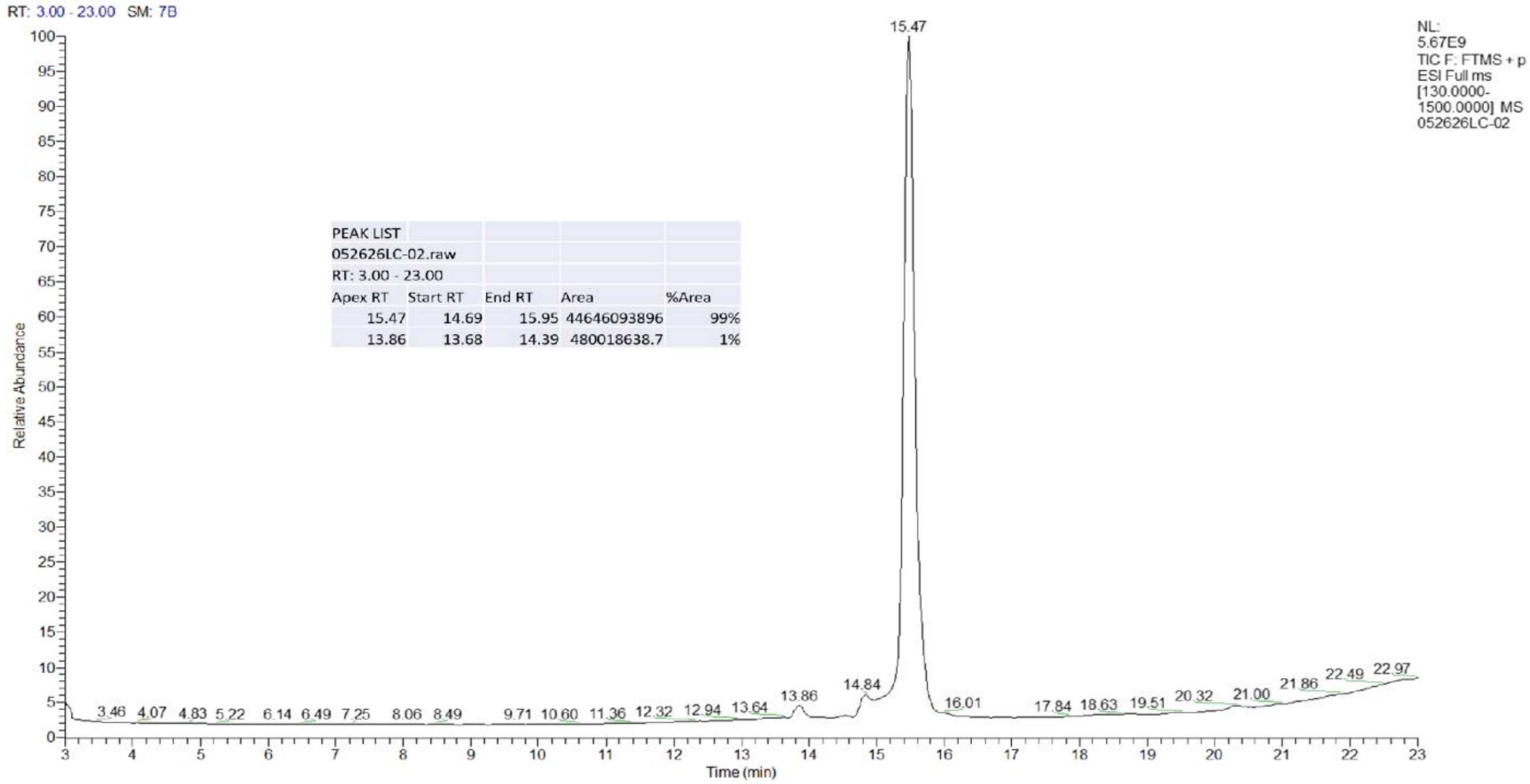

MPI-138 data of HPLC purity

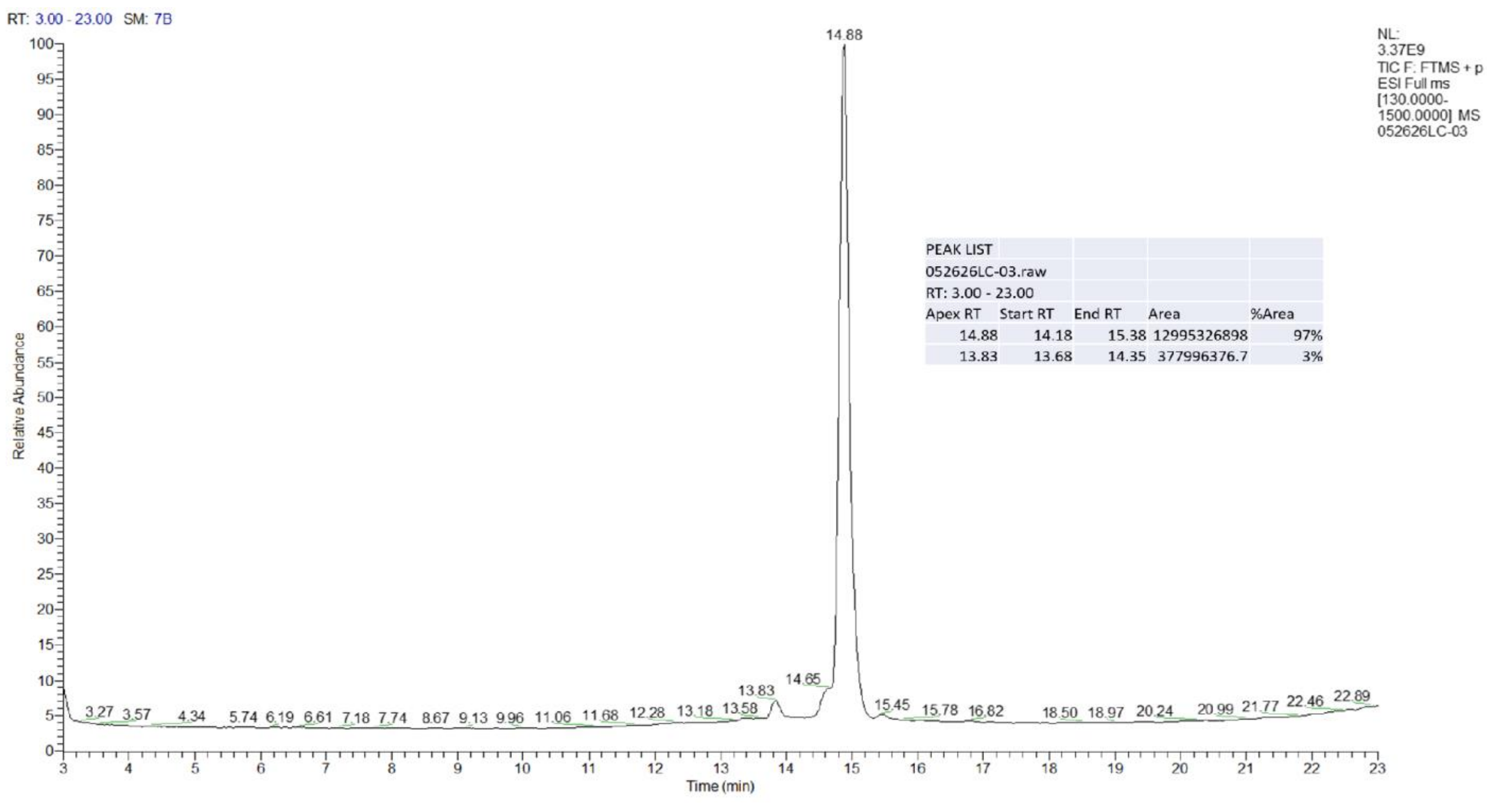

MPI-139 data of HPLC purity

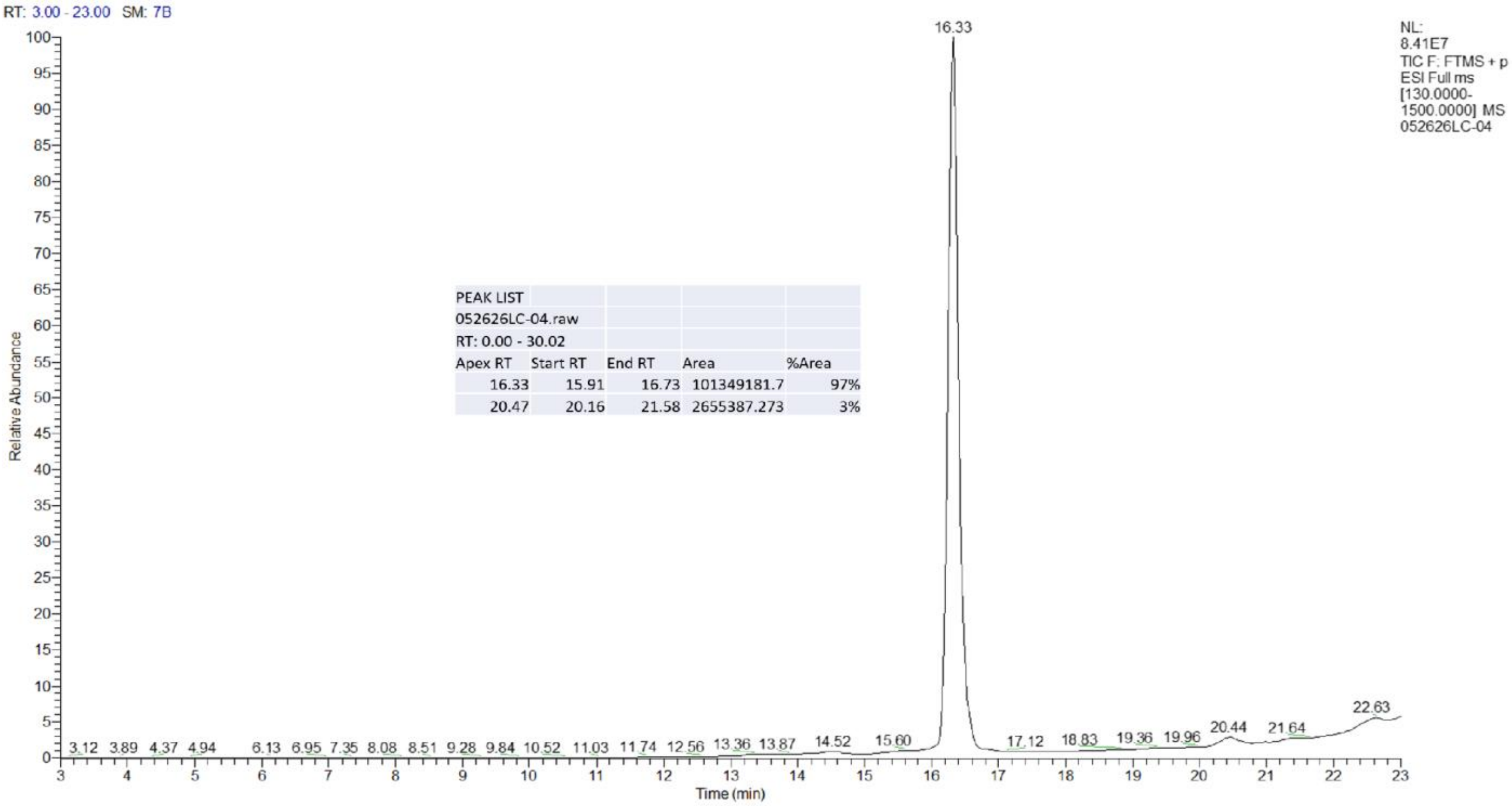

PEAK LIST				
052626LC-04.raw				
RT: 0.00 - 30.02				
Apex RT	Start RT	End RT	Area	%Area
16.33	15.91	16.73	101349181.7	97%
20.47	20.16	21.58	2655387.273	3%

MPI-140 data of HPLC purity

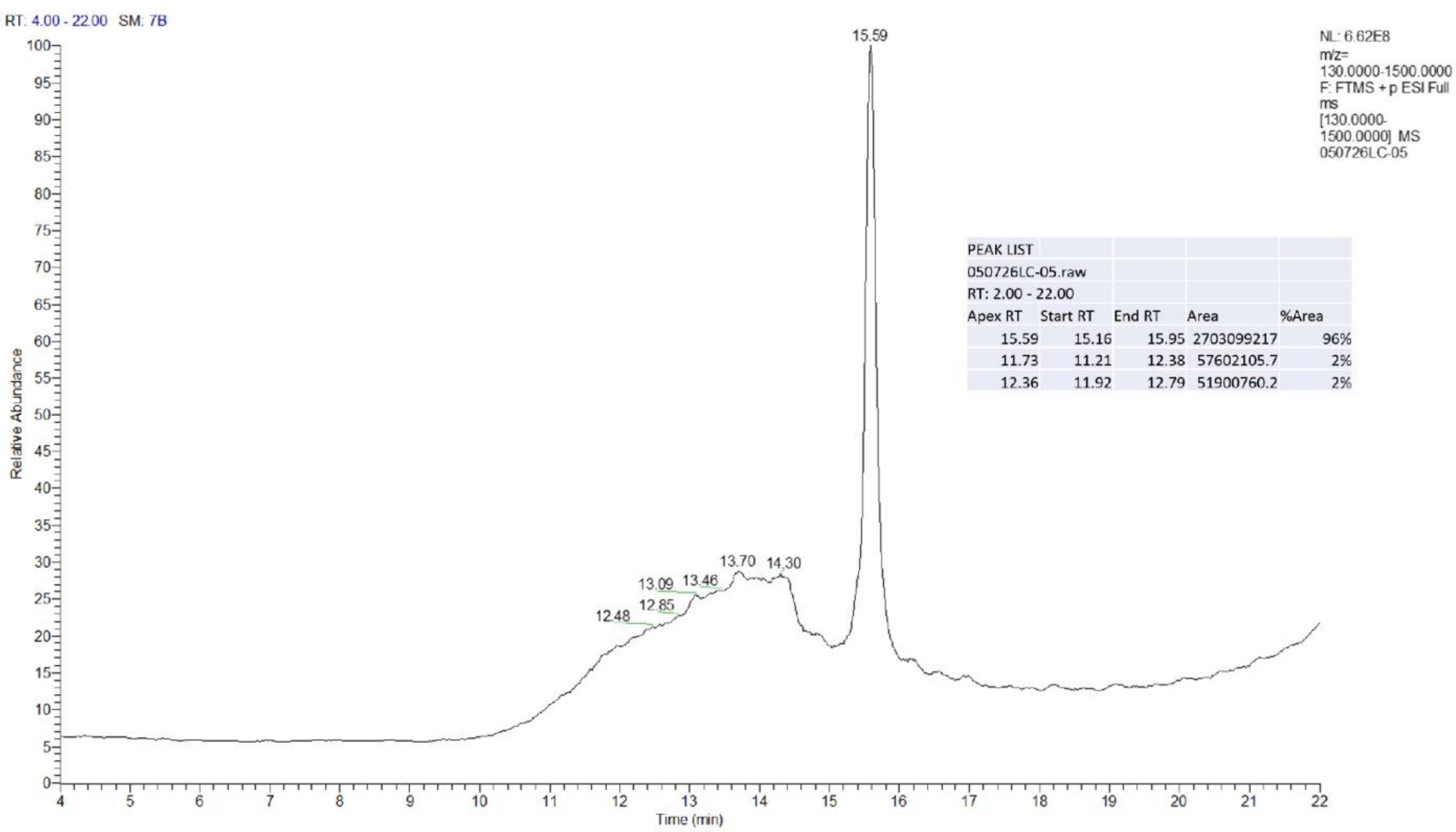

PEAK LIST				
050726LC-05.raw				
RT: 2.00 - 22.00				
Apex RT	Start RT	End RT	Area	%Area
15.59	15.16	15.95	2703099217	96%
11.73	11.21	12.38	57602105.7	2%
12.36	11.92	12.79	51900760.2	2%

MPI-144 data of HPLC purity

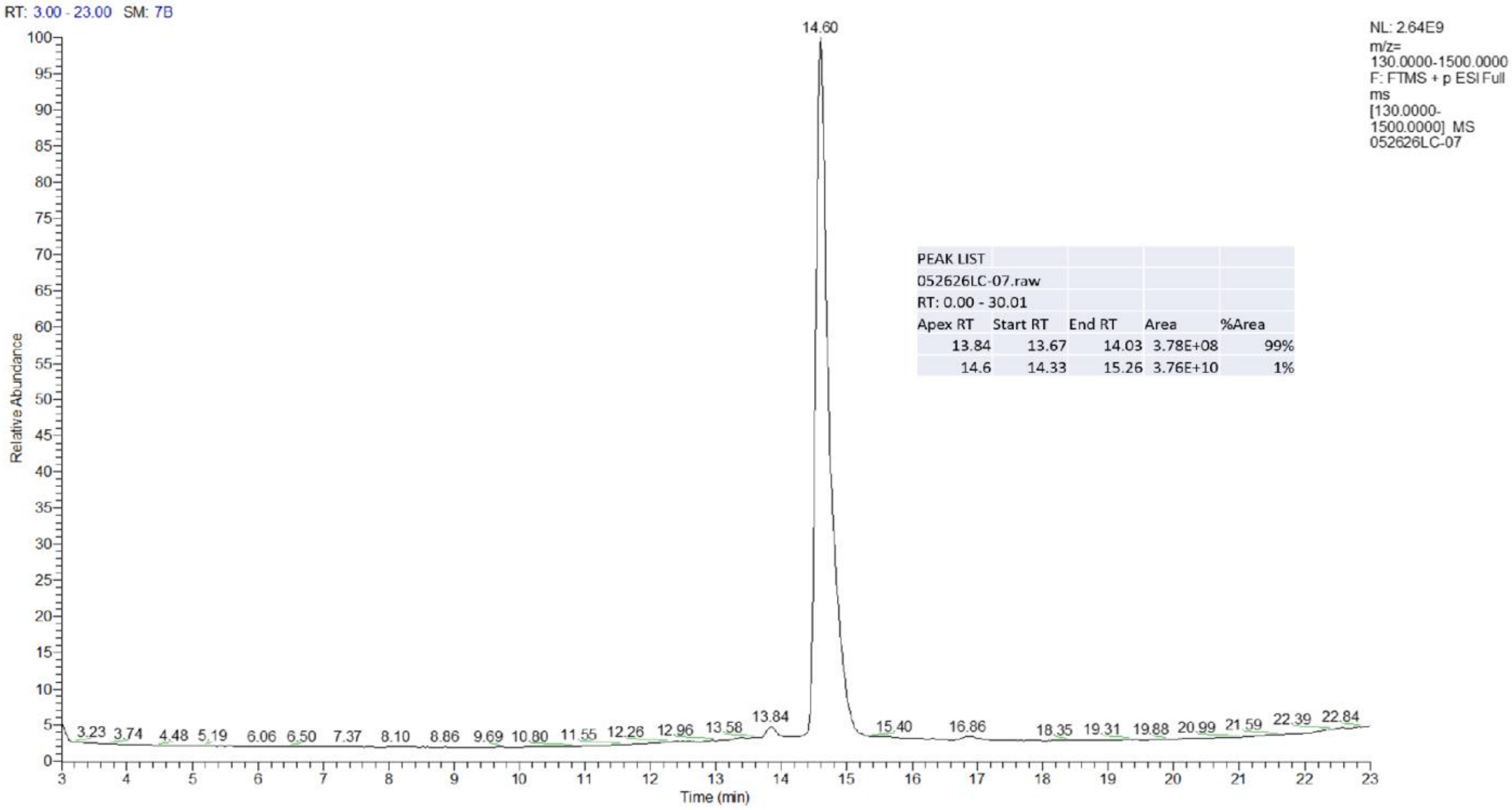

Apex RT	Start RT	End RT	Area	%Area
13.84	13.67	14.03	3.78E+08	99%
14.6	14.33	15.26	3.76E+10	1%

MPI-145 data of HPLC purity

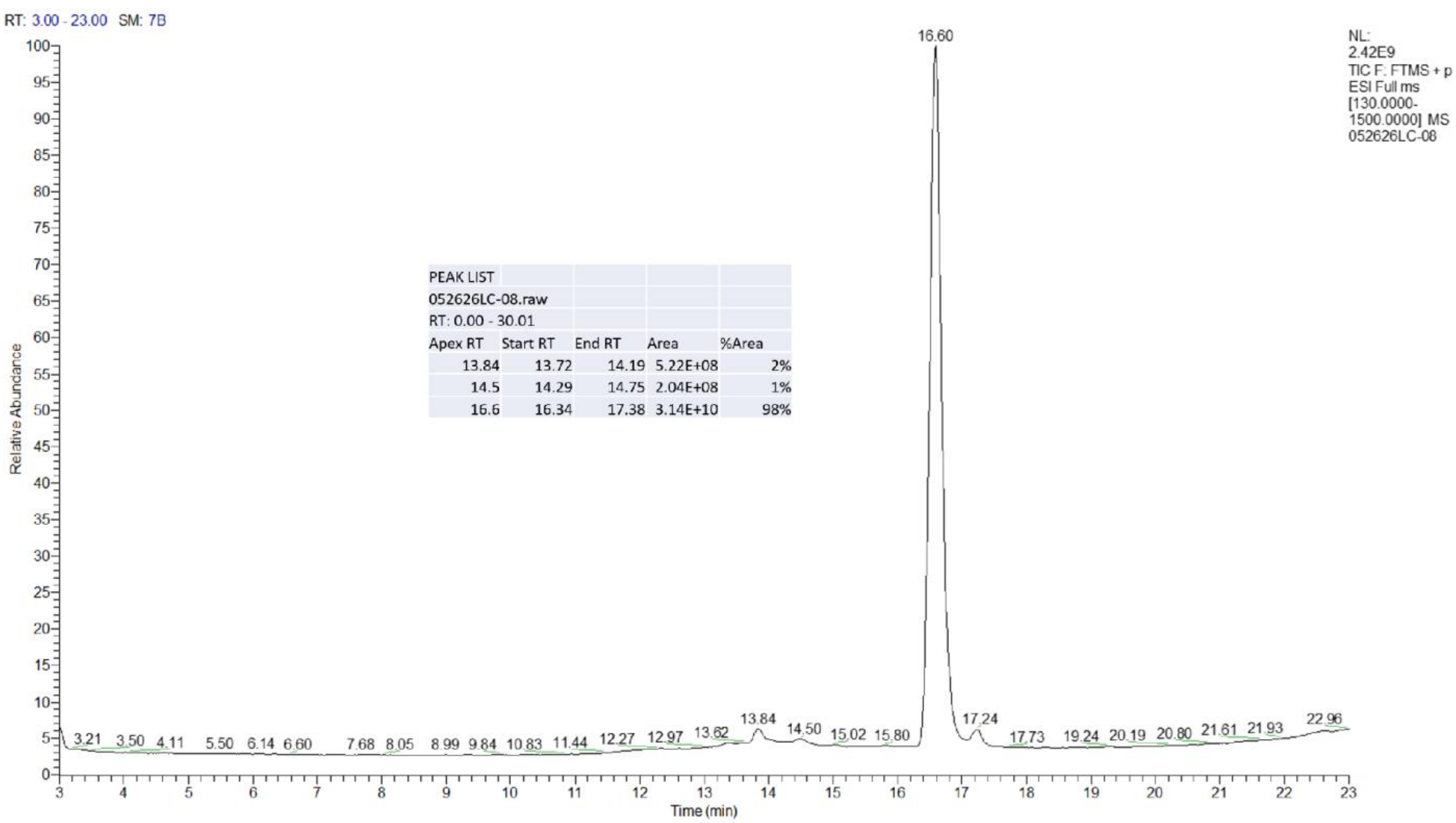

Apex RT	Start RT	End RT	Area	%Area
13.84	13.72	14.19	5.22E+08	2%
14.5	14.29	14.75	2.04E+08	1%
16.6	16.34	17.38	3.14E+10	98%

MPI-146 data of HPLC purity

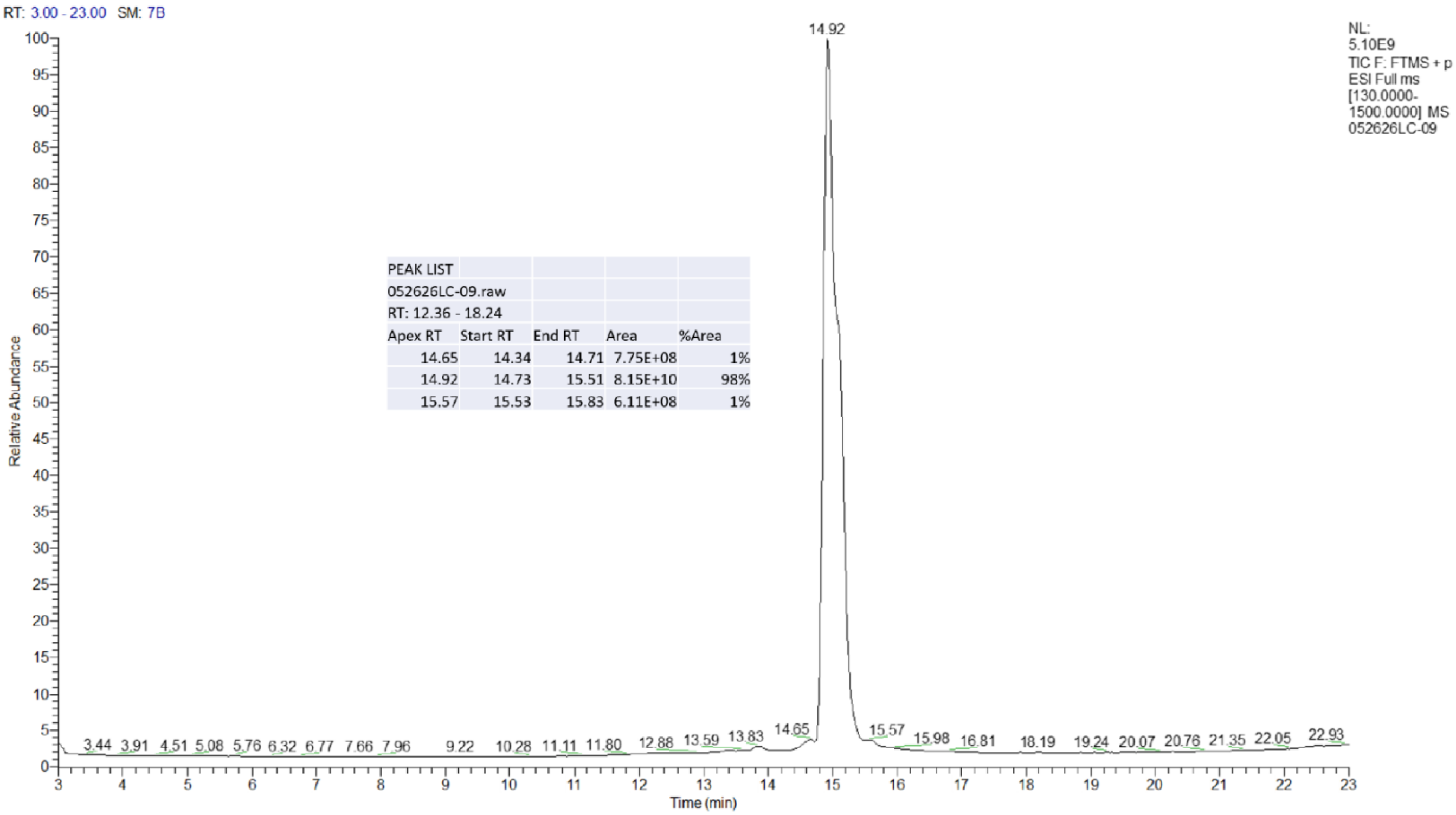

MPI-147 data of HPLC purity (Isomers)

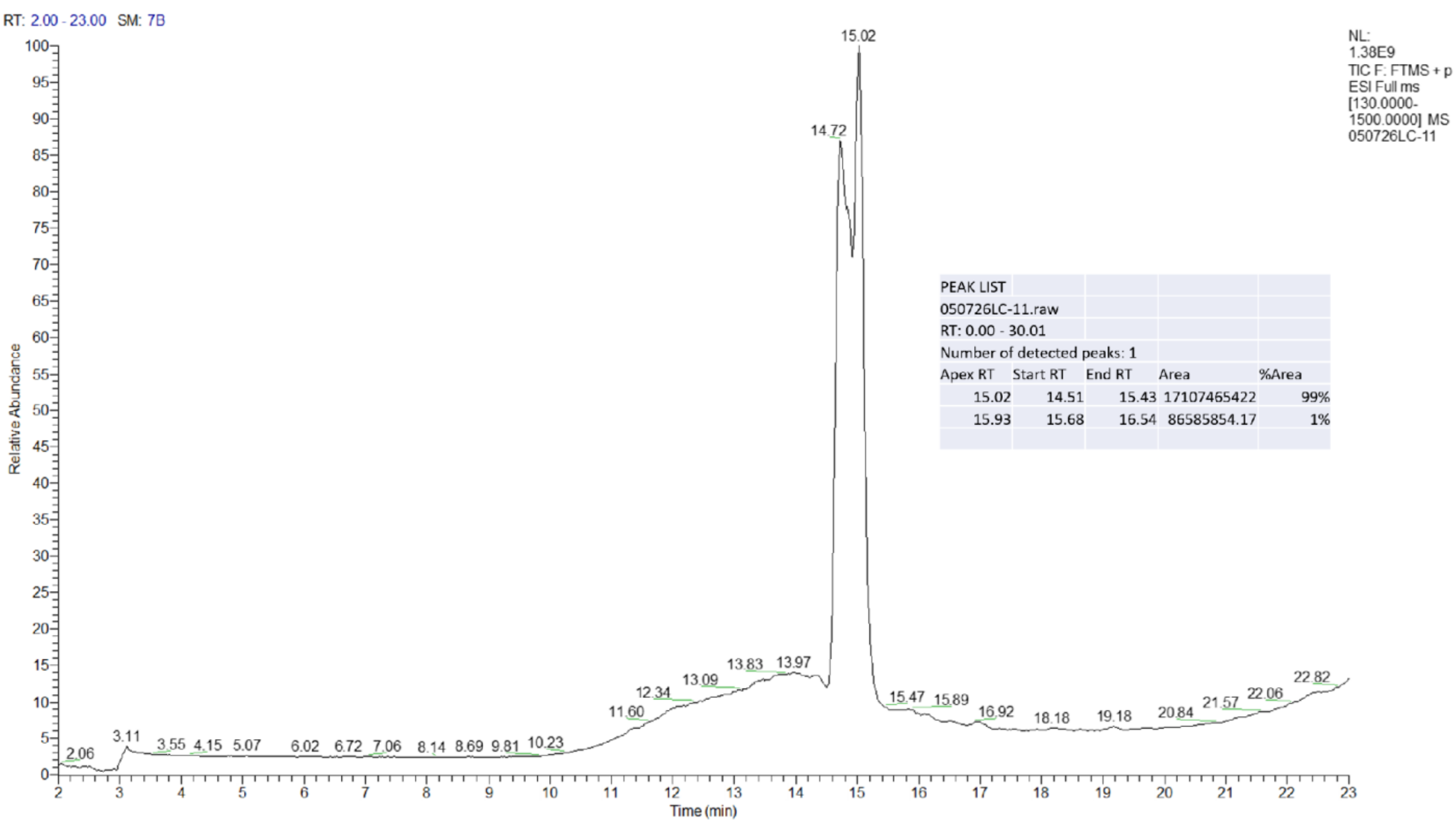

MPI-148 data of HPLC purity

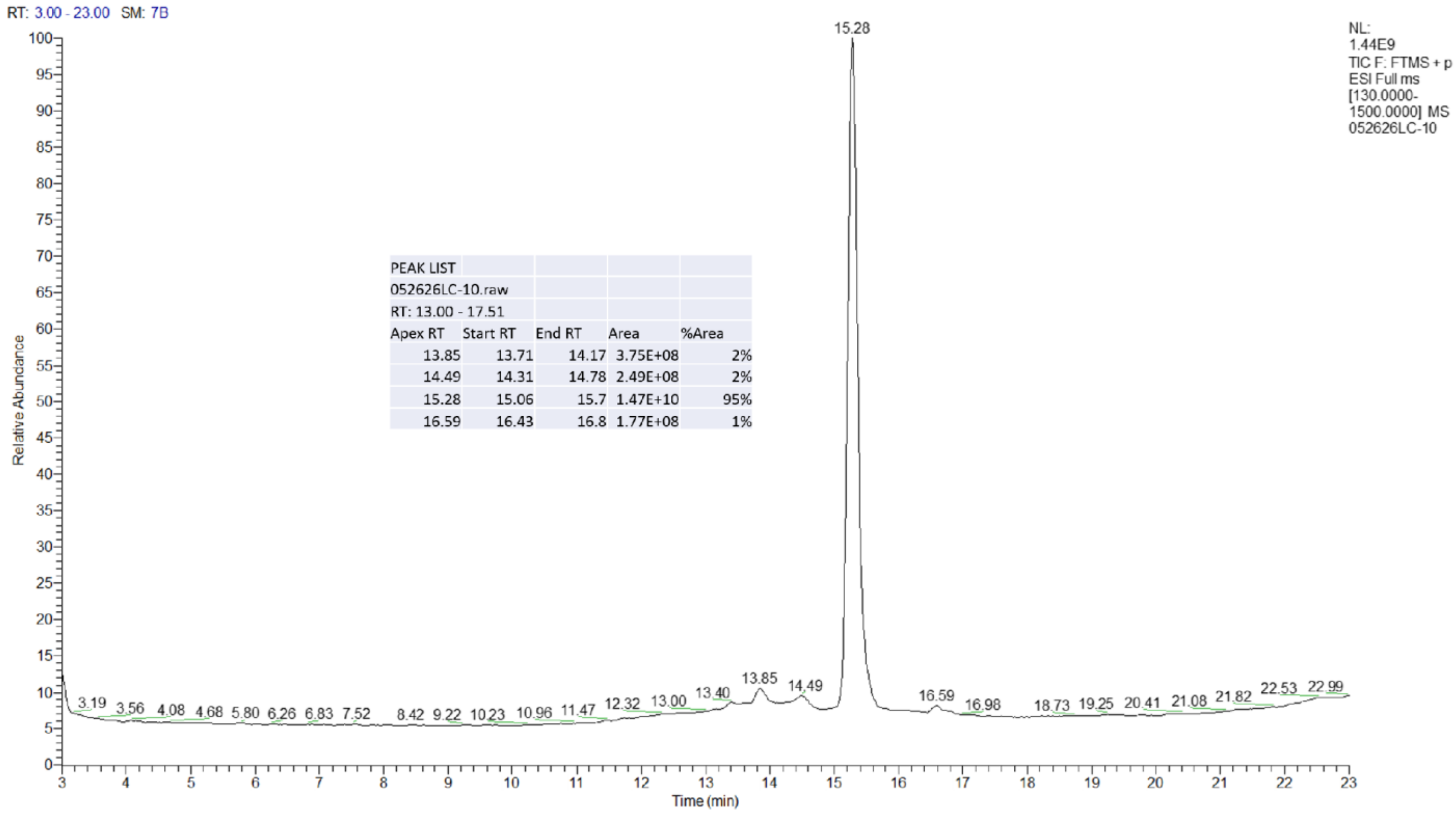

PEAK LIST				
052626LC-10.raw				
RT: 13.00 - 17.51				
Apex RT	Start RT	End RT	Area	%Area
13.85	13.71	14.17	3.75E+08	2%
14.49	14.31	14.78	2.49E+08	2%
15.28	15.06	15.7	1.47E+10	95%
16.59	16.43	16.8	1.77E+08	1%

MPI-149 data of HPLC purity

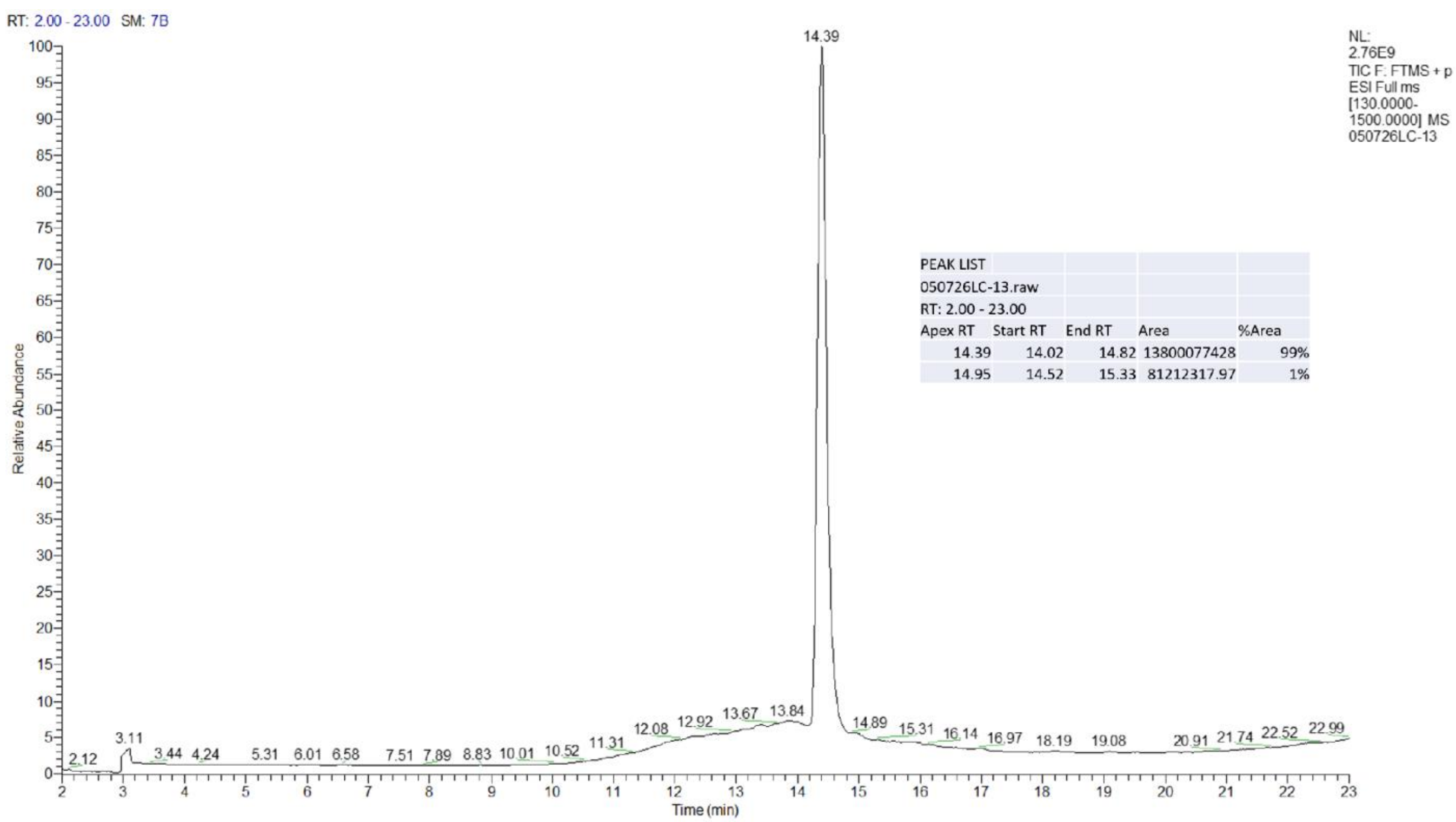

PEAK LIST				
050726LC-13.raw				
RT: 2.00 - 23.00				
Apex RT	Start RT	End RT	Area	%Area
14.39	14.02	14.82	13800077428	99%
14.95	14.52	15.33	81212317.97	1%

MPI-150 data of HPLC purity

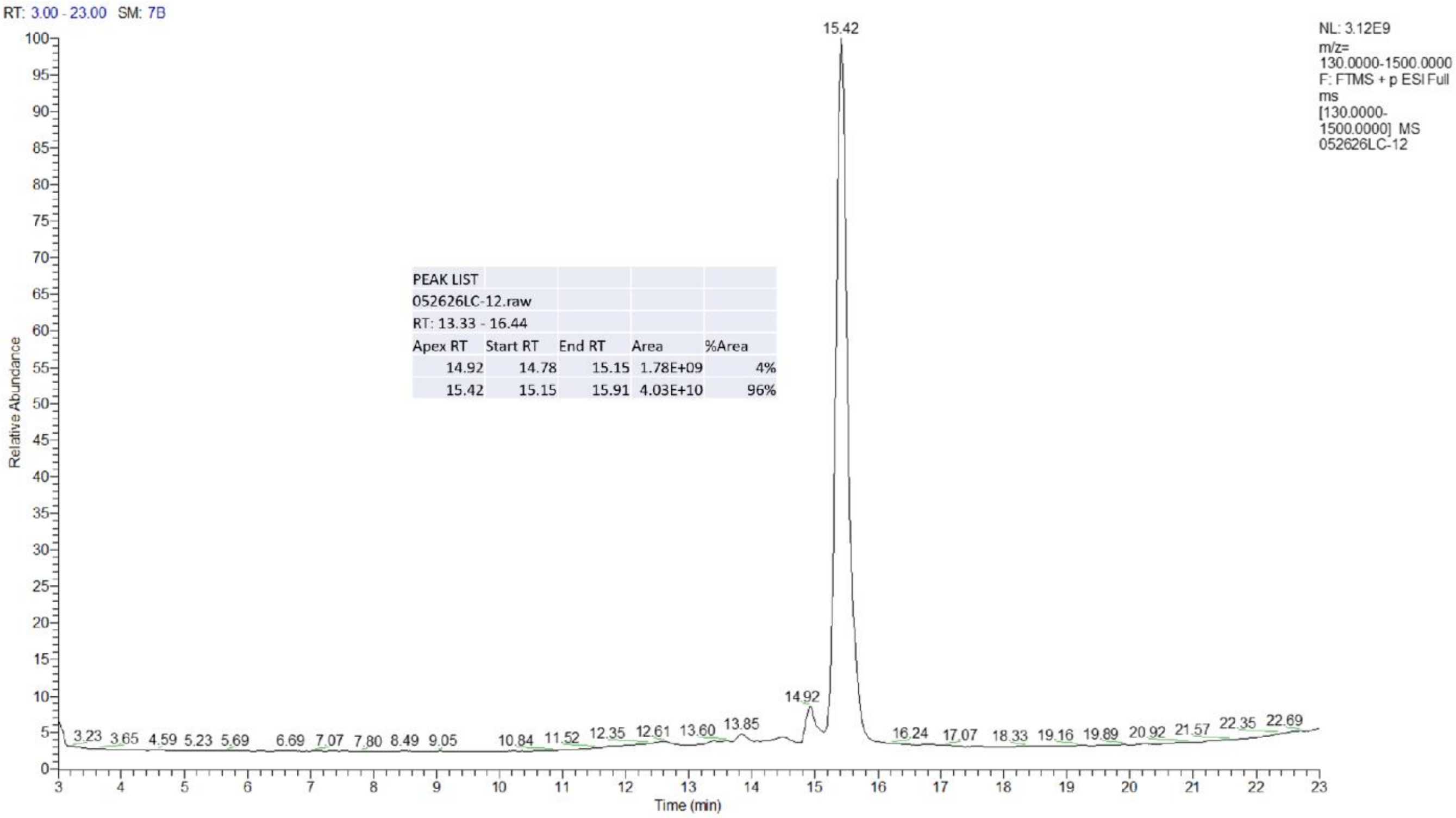

MPI-151 data of HPLC purity (Isomers)

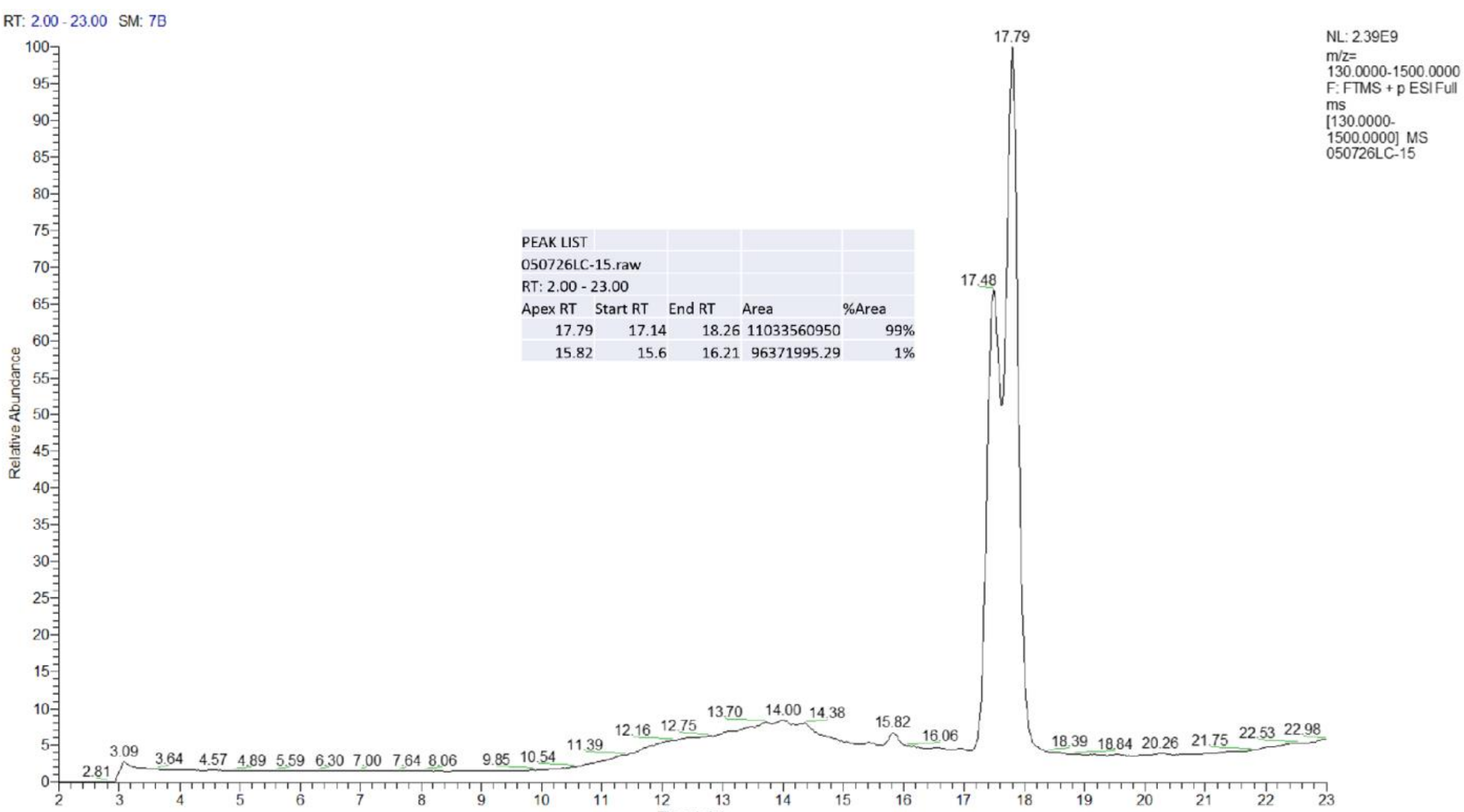

MPI-152 data of HPLC purity

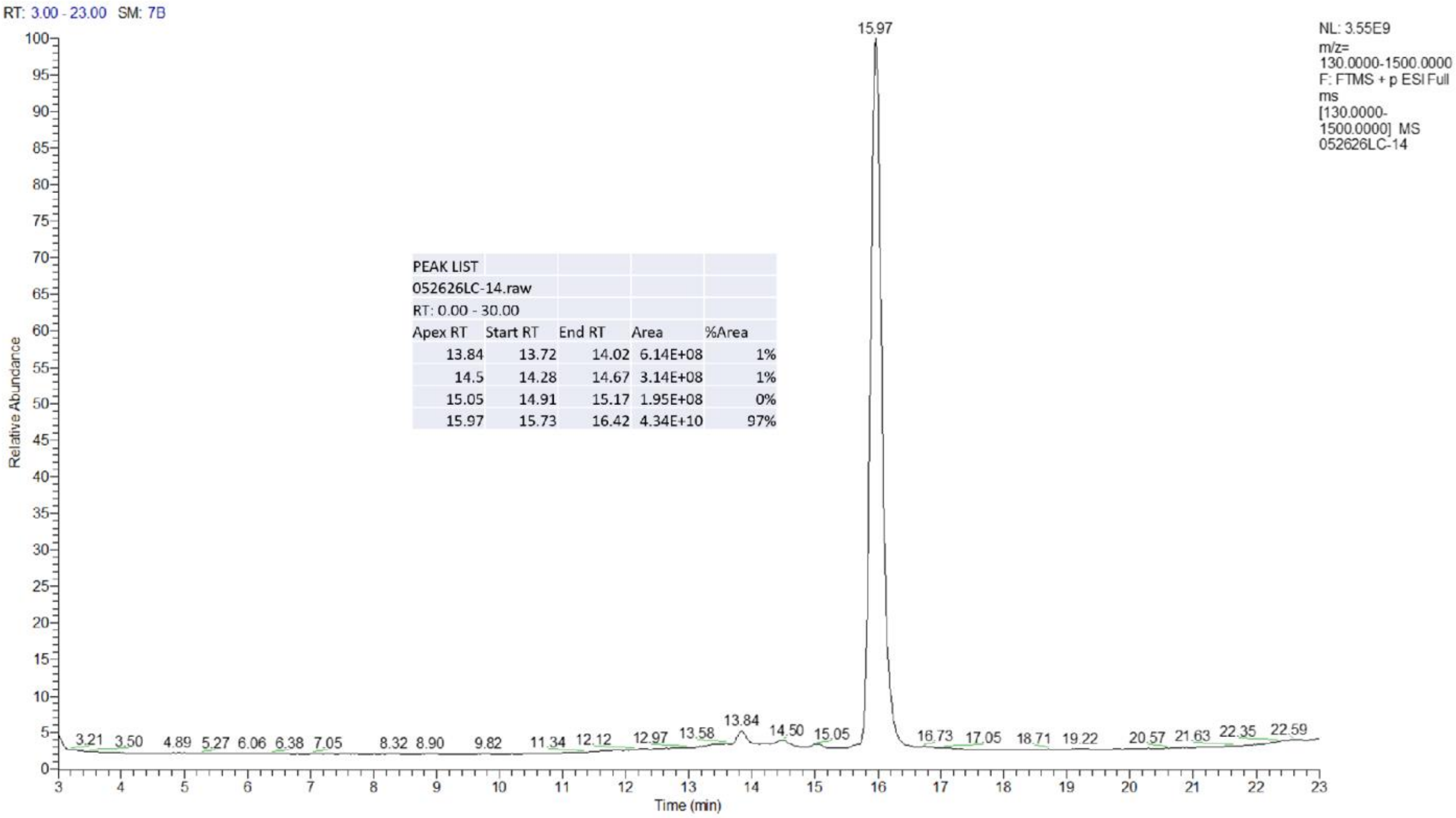

PEAK LIST				
052626LC-14.raw				
RT: 0.00 - 30.00				
Apex RT	Start RT	End RT	Area	%Area
13.84	13.72	14.02	6.14E+08	1%
14.5	14.28	14.67	3.14E+08	1%
15.05	14.91	15.17	1.95E+08	0%
15.97	15.73	16.42	4.34E+10	97%
